# Responses of the Human Gut *Escherichia coli* Population to Pathogen and Antibiotic Disturbances

**DOI:** 10.1128/mSystems.00047-18

**Published:** 2018-07-24

**Authors:** Taylor K. S. Richter, Jane M. Michalski, Luke Zanetti, Sharon M. Tennant, Wilbur H. Chen, David A. Rasko

**Affiliations:** a Department of Microbiology and Immunology, Institute for Genome Sciences, University of Maryland School of Medicine, Baltimore, Maryland, USA; b Center for Vaccine Development, Department of Medicine, University of Maryland School of Medicine, Baltimore, Maryland, USA; Institute for Systems Biology

**Keywords:** *Escherichia coli*, human challenge, resident *E. coli*, variation

## Abstract

Studies of Escherichia coli in the human gastrointestinal tract have focused on pathogens, such as diarrhea-causing enterotoxigenic E. coli (ETEC), while overlooking the resident, nonpathogenic E. coli community. Relatively few genomes of nonpathogenic E. coli strains are available for comparative genomic analysis, and the ecology of these strains is poorly understood. This study examined the diversity and dynamics of resident human gastrointestinal E. coli communities in the face of the ecological challenges presented by pathogen (ETEC) challenge, as well as of antibiotic treatment. Whole-genome sequences obtained from E. coli isolates from before, during, and after ETEC challenge were used in phylogenomic and comparative genomic analyses to examine the diversity of the resident E. coli communities, as well as the dynamics of the challenge strain, H10407, a well-studied ETEC strain (serotype O78:H11) that produces both heat-labile and heat-stable enterotoxins. ETEC failed to become the dominant E. coli clone in two of the six challenge subjects, each of whom exhibited limited or no clinical presentation of diarrhea. The E. coli communities of the remaining four subjects became ETEC dominant during the challenge but reverted to their original, subject-specific populations following antibiotic treatment, suggesting resiliency of the resident E. coli population following major ecological disruptions. This resiliency is likely due in part to the abundance of antibiotic-resistant ST131 E. coli strains in the resident populations. This report provides valuable insights into the potential interactions of members of the gastrointestinal microbiome and its responses to challenge by an external pathogen and by antibiotic exposure.

**IMPORTANCE** Research on human-associated E. coli tends to focus on pathogens, such as enterotoxigenic E. coli (ETEC) strains, which are a leading cause of diarrhea in developing countries. However, the severity of disease caused by these pathogens is thought to be influenced by the microbiome. The nonpathogenic E. coli community that resides in the human gastrointestinal tract may play a role in pathogen colonization and disease severity and may become a reservoir for virulence and antibiotic resistance genes. Our study used whole-genome sequencing of E. coli before, during, and after challenge with an archetype ETEC isolate, H10407, and antibiotic treatment to explore the diversity and resiliency of the resident E. coli population in response to the ecological disturbances caused by pathogen invasion and antibiotic treatment.

## Abstract (precis)

Research on human-associated E. coli tends to focus on pathogens, such as enterotoxigenic E. coli (ETEC) strains, which are a leading cause of diarrhea in developing countries. However, the severity of disease caused by these pathogens is thought to be influenced by the microbiome. The nonpathogenic E. coli community that resides in the human gastrointestinal tract may play a role in pathogen colonization and disease severity and may become a reservoir for virulence and antibiotic resistance genes. Our study used whole-genome sequencing of E. coli before, during, and after challenge with an archetype ETEC isolate, H10407, and antibiotic treatment to explore the diversity and resiliency of the resident E. coli population in response to the ecological disturbances caused by pathogen invasion and antibiotic treatment.

## INTRODUCTION

Escherichia coli isolates are perhaps best known as laboratory workhorses and are also known to be the etiological agents of several gastrointestinal and extraintestinal infections (1, 2). However, this species of bacteria is prevalent in the healthy human gastrointestinal tract, reaching up to 10^9^ CFU per gram in fecal matter (3). While E. coli is thought to play a critical role in the prevention of pathogen colonization (3–5), relatively little is understood about the genomic diversity and ecology of the nonpathogenic isolates that are native to the gastrointestinal tract (3, 6, 7).

With a suite of diseases, including gastrointestinal and urinary tract infections and invasive disease (e.g., meningitis), resulting from E. coli infections, the interest in the pathogenicity of this organism is warranted (1). Enterotoxigenic E. coli (ETEC), one of six generally accepted E. coli diarrheal pathotypes (1, 2), is the causative agent in nearly 80% of cases of traveler’s diarrhea, as well as the leading cause of diarrhea in developing countries, particularly in children under the age of 5 years (8, 9). ETEC comprises E. coli isolates that possess plasmid-encoded enterotoxins, including heat-labile (LT) and/or heat-stable (ST) enterotoxins (1). These enterotoxins increase the amount of cAMP in intestinal epithelial cells, resulting in increased secretion of chloride ions and water from the cell and into the gut lumen, resulting in the observed diarrhea (1, 10). The majority of ETEC strains also encode an identifiable set of host-specific colonization factors (CFs) that enable attachment to the host intestinal cells (1, 11).

Relatively little is known about the genome content of the E. coli bacteria that reside in the human gastrointestinal tract (3, 6, 7). Whole-genome sequencing has been primarily focused on pathogenic or laboratory-derived strains, ignoring much of the potential diversity of the resident, nonpathogenic E. coli isolates (3). Previous studies suggested that the strains of E. coli in the human intestine are diverse (12), having multiple distinct genotypes identified per subject in both nonpathogenic (13–16) and pathogenic (17, 18) E. coli strains. The majority of the studies of resident E. coli have been completed using non-whole-genome assays such as multilocus sequence typing (MLST), multilocus enzyme electrophoresis, and/or serotyping (3). Additionally, most work on nonpathogenic E. coli has focused on isolates from single time points, leaving much to be learned about E. coli genomic diversity within and between human hosts over time. However, these methods do not examine samples at the complete-genome level in longitudinal samples and thus overlook the detailed dynamics of the members of the resident, nonpathogenic E. coli community, especially those within the healthy gut community. This study sought to address a number of these gaps in our understanding of the resident, nonpathogenic E. coli community. We were interested in using whole-genome sequencing to determine the impact of ecological stressors on an E. coli community’s genomic diversity, in particular, those stresses imparted by a closely related pathogen and by an antibiotic to which E. coli is generally sensitive.

A recent ETEC challenge study at the University of Maryland Center for Vaccine Development provided a unique opportunity to investigate the human gastrointestinal E. coli community before, during, and after ETEC challenge and antibiotic treatment. In addition to increasing the available collection of resident E. coli genomes, the genome sequences of multiple E. coli isolates, collected longitudinally, provide insights into the diversity, dynamics, and resiliency of the members of the resident E. coli community in the human gastrointestinal tract. Dogma suggests that during diarrheal infection with ETEC, the pathogen becomes the dominant clone(s) in the gastrointestinal tract (19, 20). Treatment of the host infected with the pathogen, often with antibiotics, is then thought to further disrupt the native gastrointestinal bacteria by reducing the number of susceptible bacteria and encouraging the restructuring of the community (19, 20). However, little is known about the members of the resident E. coli community prior to challenge or about their recovery from antibiotic treatment. Furthermore, variations in the prevalence of virulence and antibiotic resistance genes in this important species are examined in the context of the observed genome variation. Overall, this report serves as a useful starting point for understanding the role of resident, nonpathogenic E. coli in resisting and recovering from incoming pathogens such as ETEC during episodes of traveler’s diarrhea.

## RESULTS

### Clinical results show differential subject responses to challenge.

Details of the challenge study and outcomes have been published previously by McArthur et al. (21). Briefly, there was an observed diarrheal attack rate of 83% (5 of 6) among those receiving the E. coli H10407 challenge. As shown in Table 1, two subjects (008 and 009) had severe diarrhea consisting of cumulative loose stools of >3 liters, one subject (015) had moderate diarrhea with cumulative loose stool of >1 liter but <3 liters, two subjects (001 and 006) had mild diarrhea with stools of >200 ml but <1 liter, and one subject (016) had no symptoms of diarrhea. These classifications of the subjects are used in the remainder of this paper. Additionally, two subjects, 004 and 019, did not receive the challenge strain and had no symptoms of diarrhea. This observed variation in clinical outcome led to questions regarding the role of the resident, nonpathogenic E. coli community in protection against or promotion of ETEC diarrheal diseases.

**TABLE 1:** The diarrheal output volume for each subject during each day of the challenge^a^

Subject ID	Diarrheal output vol (ml) at day postchallenge:									Disease severity
	0	1	2	3	4	5	6	7	Cumulative	
016	0	0	0	0	0	0	0	0	0	None
										
006	0	0	49	99	269	91	89	0	597	Mild
001	0	0	146	109	186	219	0	0	660	Mild
										
015	0	0	0	913	393	60	0	0	1,366	Moderate
										
008	0	1,102	1,141	1,106	258	65	0	0	3,672	Severe
009	0	567	2,043	1,066	159	88	0	0	3,923	Severe

^a^ The subjects are listed in order of increasing disease severity as follows: no diarrhea (None), mild diarrhea (defined as 2 or more loose stools of ≥200 ml within 48 h or a single loose stool of ≥300 ml), moderate diarrhea (cumulative loose stool of ≥1 liter), and severe diarrhea (cumulative loose stool of ≥3 liters). ID, identifier.

### Whole-genome sequencing of E. coli isolates.

E. coli isolates were obtained from extensive plating of the stool and were PCR screened for the presence of the *cfaB* gene, a marker for the E. coli H10407 isolate (the challenge pathogen), which encodes colonization factor antigen I subunit B and is required for virulence (22, 23). The *cfaB* gene was not identified in the isolates from the resident community. Ten colonies per subject time point had genomic DNA extracted, and the extracted DNA was sequenced on the Illumina platform. Where possible, both *cfaB*-positive (*cfaB*^+^) (labeled with "E" in Table S2) and *cfaB-*negative E. coli colonies were selected from each sample to provide insight into the pathogen and resident E. coli populations. The *cfaB*-positive isolates were found in subjects only from day 0 to day 4 and comprised 9.9% of the genomes obtained from subject 001, 58.3% of those obtained from subject 006, 80% of those obtained from subject 008, 100% of those obtained from subject 009, 71% of those obtained from subject 015, and 8.8% of those obtained from subject 016 (Table S2). As predicted, the subjects with moderate and severe diarrhea showed greater proportions of *cfaB*^+^ isolates than the subjects with no or mild diarrhea (see Fig. S1 in the supplemental material).

10.1128/mSystems.00047-18.1 FIG S1  Chart showing the relative proportions of isolates classified as either ETEC or non-ETEC E. coli, with significantly higher proportions of ETEC identified in subjects with moderate to severe diarrhea than in those with no or mild diarrhea (based on *z* score test). Isolates from days 0 to 4 underwent PCR analysis of the *cfaB* gene, with *cfaB*^+^ isolates designated ETEC. Download FIG S1, PDF file, 0.3 MB. Copyright © 2018 Richter et al. 2018 Richter et al. This content is distributed under the terms of the Creative Commons Attribution 4.0 International license.

The assembled resident genomes had an average of 143 contigs (range, 65 to 422), an average size of 5.17 Mb (range, 4.56 to 6.32Mb), and an average of 50.73% G+C content (range, 50.32% to 50.96%). The assembled H10407-like genomes had an average of 255 contigs (range, 201 to 344), an average size of 5.30 Mb (range, 5.08 to 6.12 Mb), and an average of 50.67% G+C content (range, 50.49% to 50.78%). Details for individual genome assemblies are presented in Table S2.

### The members of the E. coli community show a subject-specific response to pathogen challenge and antibiotic treatment.

**FIG 1:**
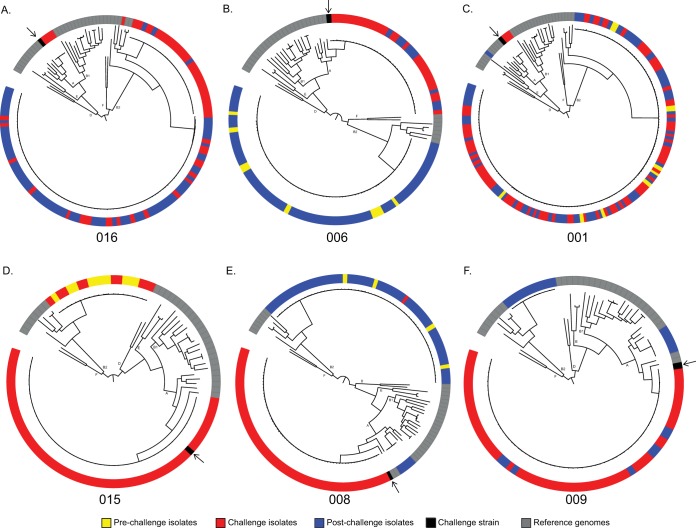
Phylogenetic analysis of isolates by subject. The whole-genome sequences of the isolates from each subject were compared with 32 previously sequenced E. coli and *Shigella* genomes (listed in Table S3) using a single nucleotide polymorphism (SNP)-based approach as previously described (57, 58). SNPs were detected relative to the completed genome sequence of laboratory isolate E. coli UTI89 using the *in silico* Genotyper (ISG) tool (58). A range of 171,581 to 175,765 conserved SNP sites which were present in all of the genomes analyzed were concatenated into a representative sequence for each genome. A maximum-likelihood phylogeny with 100 bootstrap replicates was inferred using RAxML v.7.2.8 (60). Phylogenetic trees of all isolates in each subject are listed in order of increasing disease severity. Isolates collected prechallenge are shown in yellow, isolates collected during challenge in red, isolates collected postchallenge in blue, reference strains in gray, and the challenge strain in black with an arrow.

We used phylogenomic analyses to explore alterations in the dominant E. coli community throughout the course of the challenge study. The inferred phylogenomic trees represent the E. coli genomes from each subject and 32 reference genomes that represent archetype isolates from each of the E. coli pathotypes and *Shigella* species (Fig. 1; see also Table S3). These relationships confirm that the prechallenge E. coli isolates were not closely related to H10407 and that isolates closely related to H10407 were acquired during the challenge period. The resolution of the challenge with antibiotics led to a number of different patterns of genomic relatedness.

### (i) Prechallenge.

Prechallenge, resident communities were not expected to contain ETEC strains, as all volunteers were screened for previous exposure and did not demonstrate any signs of diarrheal illness. Overall, the genomes from the prechallenge isolates were all within phylogroup B2, supporting previous studies that suggested that urinary tract isolates, also from the B2 phylogroup, are present in the gastrointestinal tract of humans which can then be selected for in the urinary tract (24) (Fig. 1, yellow isolates; see also Table S4).

### (ii) Challenge.

During the challenge period, phylogenomics analyses identified H10407-like genomes among isolates from each of the subjects. The E. coli communities in most (4/6) subjects (subjects 006, 008, 009, and 015) became dominated by ETEC H10407-like isolates during the diarrheal phase of the challenge (isolates labeled in red in Fig. 1). This was anticipated, as historical challenge studies performed with E. coli H10407 have resulted in individuals that excrete large volumes of diarrhea with significant CFU of the challenge pathogen (25). The inferred phylogeny confirmed that the E. coli communities in subjects 001 and 016 had only a limited number of H10407-like genomes and never became ETEC H10407 dominant, with the majority of the isolates belonging to the B2 phylogroup, similarly to the resident isolates in the prechallenge community. Among these subjects whose E. coli communities failed to reach ETEC H10407 dominance, subject 016 had no observable diarrhea and subject 001 had mild diarrhea (less than 1-liter total volume).

### (iii) Treatment.

Following the administration of antibiotic treatment, we were interested in (i) the speed with which nonpathogenic E. coli strains regained dominance and (ii) the degree to which the resulting community reflected the previous resident community, given that resident E. coli strains should also be sensitive to ciprofloxacin. All subjects were cleared of ETEC-like isolates within 17 h of the initiation of antibiotic treatment (Fig. 1; see also Table S1), with a return to a resident community reflective of the prechallenge condition. This rapid restoration of the prechallenge, resident E. coli strains was unexpected in that time frame. The genomes from the posttreatment isolates were found to group with the same phylogenomic and pathotype clades as those identified among the prechallenge samples (Fig. 1), with a few exceptions. Subjects 008 and 009 both had isolates with genomes from the day 28 sample that are in phylogroup A along with enteroinvasive E. coli (EIEC) reference isolates (26) (Fig. 1, blue). While these strains were phylogenetically similar to EIEC reference strains, the genomes lacked homologs of typical EIEC and the closely related *Shigella* virulence factors (1) (Table S6). Interestingly, subject 001 had a single isolate genome that grouped most closely with Shigella dysenteriae 197 (phylogroup B1) from the day 21 time point. As before, the virulence profile differed from that of Shigella dysenteriae 197 (Table S6). Subjects at these time points demonstrated no overt clinical symptoms.

10.1128/mSystems.00047-18.3 TABLE S1  Stool sample characteristics and collection details. Download TABLE S1, PDF file, 0.1 MB. Copyright © 2018 Richter et al. 2018 Richter et al. This content is distributed under the terms of the Creative Commons Attribution 4.0 International license.

10.1128/mSystems.00047-18.4 TABLE S2  Genome assembly details. Download TABLE S2, PDF file, 0.2 MB. Copyright © 2018 Richter et al. 2018 Richter et al. This content is distributed under the terms of the Creative Commons Attribution 4.0 International license.

10.1128/mSystems.00047-18.5 TABLE S3  Reference genomes. Download TABLE S3, PDF file, 0.04 MB. Copyright © 2018 Richter et al. 2018 Richter et al. This content is distributed under the terms of the Creative Commons Attribution 4.0 International license.

10.1128/mSystems.00047-18.6 TABLE S4  Details of diversity measurements from each isolate. Download TABLE S4, PDF file, 0.2 MB. Copyright © 2018 Richter et al. 2018 Richter et al. This content is distributed under the terms of the Creative Commons Attribution 4.0 International license.

10.1128/mSystems.00047-18.7 TABLE S5  Virulence factors used in LS-BSR analysis of isolate genomes. Download TABLE S5, PDF file, 0.1 MB. Copyright © 2018 Richter et al. 2018 Richter et al. This content is distributed under the terms of the Creative Commons Attribution 4.0 International license.

10.1128/mSystems.00047-18.8 TABLE S6  Virulence BSR results. Download TABLE S6, PDF file, 0.3 MB. Copyright © 2018 Richter et al. 2018 Richter et al. This content is distributed under the terms of the Creative Commons Attribution 4.0 International license.

Only two subjects (006 and 008) had samples from both the prechallenge and posttreatment periods that became ETEC dominated during the challenge period, making it difficult to make direct comparisons between the E. coli populations present before and after challenge and treatment (Fig. 1B and E; see also Table S1). The clade of non-ETEC genomes observed in these subjects (Fig. 1B and E) showed phylogenomic similarities of the prechallenge and postantibiotic treatment isolate genomes, suggesting that the E. coli members of the community had returned to the original population state following antibiotic treatment in these subjects.

### E. coli community relatedness across subjects.

**FIG 2:**
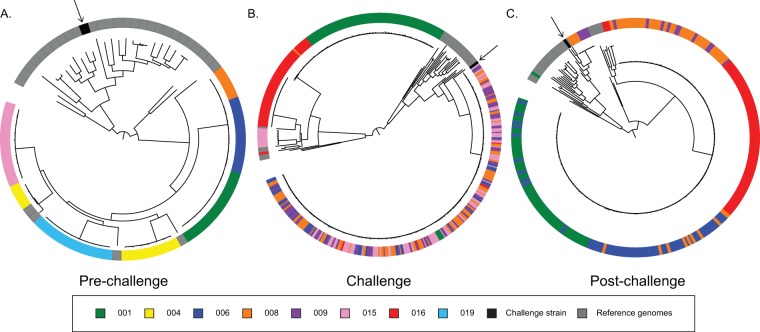
Phylogenetic analysis based on phase of challenge. The whole-genome sequences of the isolates from each subject based on the phase of the challenge were compared with 32 previously sequenced E. coli and *Shigella* genomes listed in Table S3 using a single nucleotide polymorphism (SNP)-based approach as previously described (57, 58). SNPs were detected relative to the completed genome sequence of laboratory isolate E. coli UTI89 using the *in silico* Genotyper (ISG) tool (58). A range of 67,495 to 140,098 conserved SNP sites which were present in all of the genomes analyzed were concatenated into a representative sequence for each genome. A maximum-likelihood phylogeny with 100 bootstrap replicates was inferred using RAxML v.7.2.8 (60). Phylogenetic trees of isolates from the three time periods (prechallenge [A], during challenge [B], and postchallenge [C]) show isolates from subject 001 in green, 006 in blue, 008 in orange, 009 in purple, 015 in pink, 016 in red, 004 in yellow, 019 in light blue, reference strains in gray, and the challenge strain in black with an arrow. Trees for individual time points are presented in Fig. S2.

To investigate the relatedness of isolates across subjects at each period of the challenge study, we used phylogenomic analyses that compared isolate genomes from all subjects within a time point (Fig. 2). The phylogenomic relationships of the isolates from all prechallenge samples revealed that the E. coli isolates formed distinct subject-specific lineages (Fig. 2A). Despite the fact that the resident E. coli strains were phylogenetically closely related across subjects, each subject was found to host subject-specific strains and communities of E. coli.

10.1128/mSystems.00047-18.2 FIG S2  Phylogenetic trees of all subjects at each sample day, with subject 001 data indicated in green, 006 in blue, 008 in orange, 009 in purple, 015 in pink, 016 in red, 004 in yellow, and 019 in light blue, reference strains in gray, and the challenge strain in black. Nodes with bootstrap values of at least 80 are marked with a black circle. Download FIG S2, PDF file, 8.4 MB. Copyright © 2018 Richter et al. 2018 Richter et al. This content is distributed under the terms of the Creative Commons Attribution 4.0 International license.

During the challenge period (days 0 to 4), the isolates from ETEC-dominant subjects lost their subject-specific clustering, with all H10407-like isolates forming a single, indistinguishable clade regardless of the subject of origin (Fig. 2B). These challenge strain genomes cannot be phylogenetically differentiated, suggesting that the challenge isolate genomes remained conserved throughout the challenge, regardless of host. Those subjects whose E. coli communities did not become ETEC dominated (subjects 001 and 016; Fig. 2B) maintained their individual E. coli phylogroup B2 communities throughout the challenge period.

The E. coli isolate genomes largely returned to their pattern of subject-specific clades within phylogroup B2 following treatment with ciprofloxacin (days 4 to 8), with some notable exceptions (Fig. 2C). Of particular interest are the samples from subjects 008 and 009 collected on day 6, which were phylogenomically similar, in contrast to the individual-subject-based groupings seen elsewhere. This occurred again on day 28, the day when both subjects were dominated by phylogroup A strains that were similar but not identical (outlined in Table S8). The posttreatment E. coli isolates from subject 009 were cultivable for only days 6 and 28 postchallenge, so no long-term pattern can be determined (Table S1).

10.1128/mSystems.00047-18.9 TABLE S7  Proportions of resistance genes relative to the number of genomes in each subject during each trial phase. Download TABLE S7, PDF file, 0.04 MB. Copyright © 2018 Richter et al. 2018 Richter et al. This content is distributed under the terms of the Creative Commons Attribution 4.0 International license.

10.1128/mSystems.00047-18.10 TABLE S8  Gene-based differences among the isolates from subjects 008 and 009 at day 28. Download TABLE S8, PDF file, 0.1 MB. Copyright © 2018 Richter et al. 2018 Richter et al. This content is distributed under the terms of the Creative Commons Attribution 4.0 International license.

### Diversity among resident E. coli strains and relevance to disease severity.

Given the observed variations in disease severity, we were interested in the relationship between diversity in the E. coli resident community and disease severity. On the basis of the diversity-stability hypothesis and the notion that the ETEC pathogen would be less likely to establish a niche in a more diverse community (27, 28), we anticipated an inverse correlation between disease severity and the diversity of the E. coli isolates identified. Although the phylogenomic analyses suggest that the resident E. coli isolates within a subject were closely related, the level of diversity within these communities was further explored using *in silico* multilocus sequence typing (MLST), serotyping, and gene content comparisons. As summarized in Table 2 (and detailed in Table S4), E. coli diversity was subject specific and was not fully captured by any single *in silico* analysis method. The most common MLST sequence type was ST131 (with serotype O25:H4), which dominated the population in all subjects except subject 015 (dominated by ST2015) and subjects 004 and 019, neither of whom completed the challenge portion of the study (Table S4).

**TABLE 2:** Summary of the diversity measurements of the resident isolates from each subject, with the subjects ordered for increasing disease severity

Parameter	Diversity value(s) for resident E. coli from subject:					
	016	006	001	015	008	009
No. of phylotypes	1	1	2	1	2	2
No. of MLST sequence types	4	5	6	3	5	2
Serotype	1	1	3	1	3	4
% unique genes compared to reference	21–30	34–48	35–46	21–26	35–48	24–27

The large-scale BLAST score ratio (LS-BSR) assay was used to analyze the gene content variation among the resident E. coli isolates as a measure of diversity among closely related strains of the same species. Resident E. coli strains from all subjects demonstrated variability in gene content differences relative to the E. coli UTI89 isolate, with the range in variability indicating genetic diversity within the community (Table 2). The isolates from subjects 001, 006, and 008 demonstrated a greater degree of genomic diversity (Table 2).

While these data demonstrate that the levels of diversity of the resident E. coli community differed across host subjects, there was no apparent correlation between the detected E. coli diversity and susceptibility to the H10407 pathogen or the observed disease state.

### Impacts of challenge and treatment on virulence and resistance gene profiles.

After observing that the prechallenge E. coli community was restored following treatment, we were interested in the lasting impacts of challenge and treatment on the virulence and resistance potential of the genomes of the resident community. Results from previous studies performed with the challenge strain suggest that H10704 derived from a resident E. coli strain that had acquired plasmid-bound virulence genes, which suggests that the virulence plasmids can be exchanged with the resident E. coli (29). Furthermore, antibiotic use is known to contribute to the acquisition of antibiotic resistance among exposed bacteria (30, 31). We queried the data for any lingering effects of challenge and treatment by examining the canonical virulence and resistance gene profiles of the E. coli communities at each stage of the challenge study.

**FIG 3:**
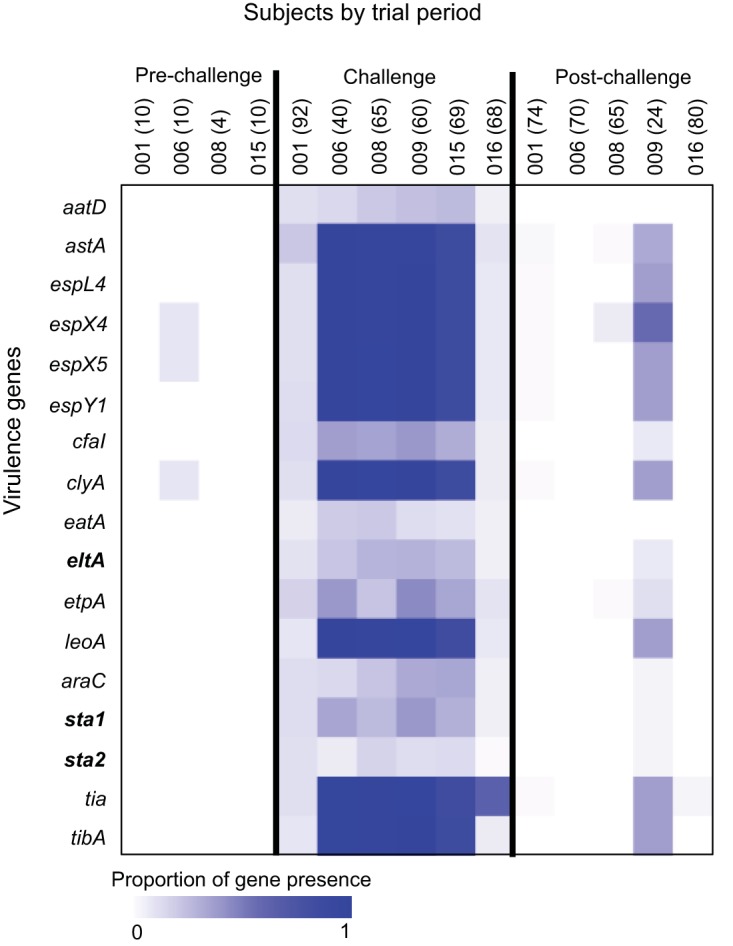
Virulence gene presence and absence. The proportions of isolate genomes containing virulence factors in each subject and time period are presented as a heat map. Gene sequences of known E. coli virulence genes were queried against the isolate genomes using LS-BSR. Genes with a significant sequence match (BSR > 0.8) were deemed present, and the total number of present virulence genes is presented as a proportion of the total number of isolates in that time period (in parentheses). Genes involved in ETEC toxin (LT and ST) production are highlighted in bold. Other listed genes are involved in ETEC adhesion or virulence in other E. coli pathotypes, with details in Table S5. BSR assay results from individual isolates for each investigated virulence factor gene are presented in Table S6.

**FIG 4:**
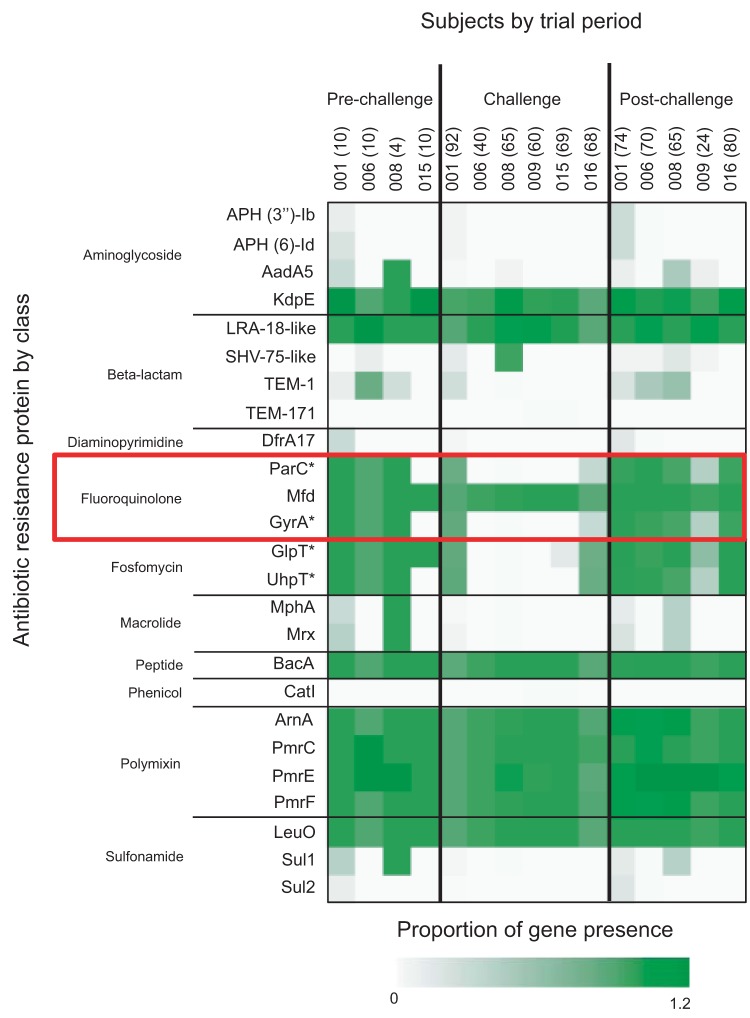
Proportions of antimicrobial resistance genes associated with isolates in each phase of study. The proportions of isolate genomes containing predicted antibiotic resistance in each subject and time period are presented as a heat map. Protein sequences known to confer E. coli antibiotic resistance were queried against the translated isolate genomes using CARD-RGI. The total number of resistant protein sequences present is presented as a proportion of the total number of isolates in that time period (in parentheses), with multiple copies in some isolates. The resistance sequences are listed by the category of antibiotic to which they provide resistance. Those sequences marked with an asterisk (*) provided antibiotic resistance due to a sequence mutant or variation (detailed in Table S7).

Results of comparisons of the gene profiles from prechallenge and posttreatment isolates mirror what was observed in the phylogenetic analyses (Fig. 3 and 4). None of the resident E. coli isolates contained canonical ETEC virulence factors, including heat-labile toxin (LT), heat-stable toxin (ST), or colonization factors (CFs), either before or after E. coli H10407 challenge, indicating that the subjects were not colonized with an ETEC isolate prior to the challenge and that transfer of the ETEC virulence factors to the resident E. coli strains did not appear to occur during the challenge period in any of the subjects. This ETEC virulence gene presence was less pronounced in subjects 001 and 016, for whom ETEC H10407 never became a dominant member of the E. coli population.

Examination of a panel of common antimicrobial resistance genes in the collection of isolates in the study showed that the resident E. coli genomes contained at least three genes or mutations that are known to result in resistance to fluoroquinolones (such as ciprofloxacin), even in the isolates collected prior to challenge and following treatment (Fig. 4). In a functional examination, ciprofloxacin resistance of the resident E. coli isolates demonstrated that pre- and postchallenge isolates from all subjects, where available, could grow on 30 µg/ml of ciprofloxacin. This resistance to fluoroquinolones, most likely due to a mutation in the *gyrA* and *parC* genes, is common among E. coli ST131 isolates (32–35). As ST131 isolates have garnered much attention for harboring drug resistance and virulence genes, we further explored the ST131 isolates from the subjects in this study. The isolates were all ST131 subclone H30R1 clade C (based on *fimH* typing), containing the TEM and SHV-type beta-lactamases but lacking the CTX-M extended-spectrum beta-lactamases (32).

## DISCUSSION

This study examined the E. coli community of the human gastrointestinal tract, using genomics to explore the diversity and dynamics of this prevalent member of the gastrointestinal microbiome, particularly in response to the introduction of a closely related pathogen and treatment with antibiotics. Overall, the majority of the members of each E. coli population appeared to be subject specific, with genomes from any single subject at any time point being more closely related to those from the same subject than to those from other subjects at that or any other time point.

During the challenge study, most subjects became ETEC H10407 dominant, with the ETEC isolates overwhelming the resident, nonpathogenic E. coli strains, leading to the loss of subject-specific communities (Fig. 2). Within 6 to 17 h after the first antibiotic dose, the H10407-like organisms were no longer identifiable by culture or genomics, leaving the previous resident E. coli population to recover its prechallenge population. On the basis of the genomic comparisons, it appears as though the members of the resident E. coli population survive or tolerate the introduction of a known pathogen at high doses in addition to subsequent antibiotic treatment and are able to reestablish dominance within the community. In each case where it was capable of being interrogated, the resident E. coli community reestablished dominance, and additional isolates were identified in two subjects (008 and 009) only at later time points (highlighted in Fig. 2).

Perhaps a key to the resiliency of the resident E. coli population is its resistance to the antibiotic ciprofloxacin. Genome analyses suggest that the resident E. coli genome contains genes and mutations known to confer resistance to several classes of antibiotics, including the fluoroquinolones, such as ciprofloxacin (Fig. 4). The predicted ciprofloxacin resistance was confirmed by growth of prechallenge resident E. coli isolates in the presence of ciprofloxacin. This resistance to the ciprofloxacin treatment was able to allow the resident population to recover following the removal of the antibiotic-sensitive E. coli H10407 challenge strain. The resident populations in most subjects (001, 006, 008, 009, and 016) were dominated by the isolates of ST131. Members of this sequence type are well known for their resistance to antibiotics, particularly fluoroquinolones and beta-lactams, and are common members of resident gut communities throughout the developing world (33, 34, 36, 37). While we are unable to say for certain why the subjects in this study were colonized by ST131, studies have demonstrated high rates of colonization among people who have traveled to countries of endemicity or have stayed in long-term health care facilities and that CTX-M-containing ST131 clones have spread rapidly in the United States, where it is now a dominant lineage (35, 38–41). Furthermore, ST131 is an efficient and prolonged colonizer, known to outcompete other resident strains and predicted to persist in the gastrointestinal tract for years (40, 42, 43), which may also help explain the readiness with which the resident E. coli population recovered following challenge and treatment.

Upon observing the variability in clinical responses to a controlled pathogen input, we were interested in exploring the role of the resident E. coli community in preventing or enhancing the disease severity of an introduced, virulent E. coli strain. While earlier studies on diarrheagenic bacteria, such as the Global Enteric Multicenter Study (GEMS) (8, 9, 25), described the detection of pathogens in the absence of clinical symptoms, they lacked the opportunity to control for a common, known, and quantified pathogenic input such as was included in the design of this challenge study. While we are unable to make sweeping generalizations on the basis of the limited number of subjects, we can conclude that human disease severity is the result of the presence of a bacterium encoding virulence genes in addition to host factors (genetics, immune system status, nutrition status, etc.) and/or the microbiome. Additional studies examining the immune status of these subjects (21) and the gastrointestinal microbiome from this cohort (Richter et al., unpublished results) integrated with this genome-based study will provide a systems view of the host-pathogen interactions. Here we specifically explored the role of the resident E. coli community in ETEC disease severity, with the idea that ETEC would be less likely to find a necessary niche among the members of a more diverse resident E. coli community such as has been described in animal models (44–46).

Differential susceptibilities to ETEC colonization were observed, in that ETEC H10407 did not become a dominant community member in some subjects (001 and 016). Unsurprisingly, diarrhea was either mild or nonexistent in each of those cases (Fig. 1; see also Table 1). On the opposite end of the spectrum, the subjects with the most severe clinical presentation as determined on the basis of diarrheal output (subjects 008 and 009) demonstrated the greatest degree of instability in their E. coli populations, where the dominant E. coli strains shifted between phylogroups B2 and A three times in the course of the study (Fig. 1). While the examined E. coli populations differed between subjects in their degrees of genetic diversity, the diversity of these populations was not correlated with the observed stability and determined neither susceptibility to ETEC colonization nor disease severity (Table 2).

The current study afforded a unique opportunity to longitudinally study both pathogenic and resident E. coli strains in the human gastrointestinal tract over the duration of the challenge study. In addition to providing new genomes for the study of the diversity of nonpathogenic E. coli strains, these data demonstrate the resiliency of the E. coli community in response to extreme ecological disturbances, namely, pathogen and antibiotic introduction. This report serves as a useful starting point for understanding the role of E. coli within the larger bacterial community of the human gastrointestinal tract in comparison to examining only the pathogen in isolation. Understanding these interactions between pathogen and resident nonpathogen will allow us to potentially exploit those nonpathogens as part of a therapy for resisting incoming pathogens such as ETEC during traveler’s diarrhea.

## MATERIALS AND METHODS

### Challenge conditions and stool sample collection.

Fecal samples were obtained from adult volunteers participating in an enterotoxigenic Escherichia coli (ETEC) challenge study performed by the Center for Vaccine Development at the University of Maryland School of Medicine in Baltimore, MD (21). The study was approved by the Institutional Review Board of University of Maryland, Baltimore (UMB). Written informed consent was obtained from healthy adult volunteers 18 to 49 years of age, who were screened for the absence of chronic medical conditions and immunodeficiencies. Participants were excluded if they had received antibiotics in the 2 weeks prior to the study or if they had had previous exposure to ETEC or Vibrio cholerae.

After overnight fasting, subjects ingested 10^8^ CFU of challenge strain E. coli H10407 (29) and were observed in an inpatient research isolation ward, where they were closely monitored for signs of diarrheal illness. As has been previously described (47), each stool was graded as follows: grade 1, firm; grade 2, soft; grade 3, thick liquid; grade 4, opaque watery; grade 5, rice water. All stools of ≥grade 3 were considered loose, and the volume was measured (see Table S1 in the supplemental material). Any individual who developed loose stool was offered an oral rehydration salts (ORS) solution (Jianas Brothers, Kansas City, MO) or intravenous lactated Ringer’s solution at a volume 1.5 times the loose stool volume. Ciprofloxacin was administered (500 mg twice daily for 5 days) to any subject who exceeded 3 liters of cumulative loose stool output or on day 4 postchallenge, whichever occurred first. Individuals were discharged when they were asymptomatic, completed a course of ciprofloxacin therapy, and demonstrated 3 sequential stool cultures (separated by 12 h) that were negative for E. coli.

**FIG 5:**
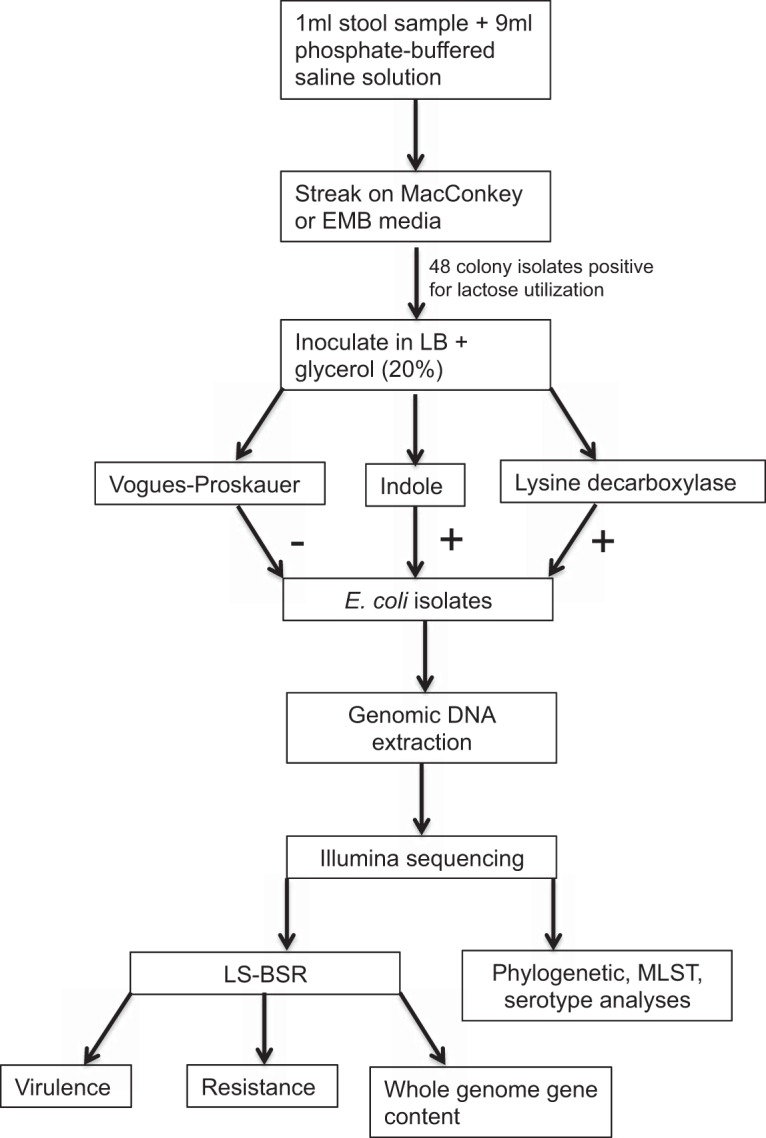
Flow chart of isolate extraction, sequencing, and data analysis.

The sample collection and challenge timeline is shown in Table S1. Subjects also provided fecal samples following discharge from the challenge facility on days 14, 21, and 28 postchallenge. Six subjects (designated 001, 006, 008, 009, 015, and 016) participated in the full challenge study, and an additional two (004 and 019) provided only prechallenge samples. A flow chart of the sample preparation and processing is shown in Fig. 5.

### E. coli colony isolation and pathogen screen.

To isolate E. coli from the stool, samples were streaked for single colonies onto MacConkey or eosin methylene blue (EMB) media. Single colony isolates positive for lactose utilization were picked from each stool sample, inoculated into a 96-well microtiter plate containing Miller’s LB broth (Research Products International), supplemented with 20% glycerol (final), and stored at −80°C as stock isolates.

Three biochemical tests, Voges-Proskauer (VP) (48), lysine decarboxylase (49), and indole (50), were used to confirm the evidence of the presence of enteric bacteria and to differentiate E. coli from other *Enterobacteriaceae* (51). The presumptive E. coli isolates were grown (i) in methyl red-VP (MR-VP) medium (Difco) and tested for acetoin production using Voges-Proskauer reagent (BioMérieux) according to the manufacturer’s instructions, (ii) on decarboxylase medium base (Difco) supplemented with l-lysine (Sigma) to test for l-lysine utilization, and (iii) in L-broth to test for tryptophan utilization using Remel indole reagent (Thermo Scientific) according to the manufacturer’s instructions. Lactose-positive isolates on MacConkey plates that were VP negative, lysine decarboxylase positive, and indole positive were identified as E. coli (Fig. 5).

The isolates were further tested with a PCR assay for the colonization factor antigen gene B (*cfaB*) gene (52, 53) (using forward primer 5′GCTTATTCTCCCGCATCAAAAAC and reverse primer 5′TTACACCGGATGCAGAATATC) from enterotoxigenic E. coli H10407 (29) to distinguish the input pathogen from the resident E. coli strains, which should lack *cfaB*.

### DNA extraction, sequencing, and assembly.

Bacterial genomic DNA was purified from 10 presumptive E. coli isolates from each stool sample using a GenElute bacterial genomic DNA kit (Sigma) according to the instructions of the manufacturer and subjected to whole-genome sequencing. Where possible, both *cfaB*-positive isolates and *cfaB*-negative isolates were analyzed from each stool sample. DNA was sequenced on the Illumina HiSeq platform at the Genome Resource Center at the University of Maryland School of Medicine, Institute for Genome Sciences (http://www.igs.umaryland.edu/resources/grc/). The resulting 100-bp reads were assembled as previously described (54, 55) using the Maryland Super-Read Celera Assembler (MaSuRCa version 2.3.2) (56). Contigs of fewer than 200 bp were excluded from assemblies. Genomes containing greater than 500 contigs (25 genomes total) were excluded from further analysis. The assembly details and corresponding GenBank accession numbers are provided in Table S2.

### Phylogenomic analyses.

Phylogenomic trees were inferred using assembled genomes from this study in addition to 32 E. coli and *Shigella* reference genomes from GenBank (Table S3) (57). Single nucleotide polymorphisms (SNPs) in all genomes were detected relative to the completed genome sequence of ETEC isolate E. coli H10407 using the *in silico* Genotyper (ISG) v.0.12.2 tool (58), which uses MUMmer v.3.22 (59) for SNP detection. These SNP sites were concatenated and used for phylogenetic analysis as previously described (17). A maximum-likelihood phylogeny was generated with RAxML v.7.2.8 (60) using the GTR model of nucleotide substitution with the gamma model of rate heterogeneity and 100 bootstrap replicates and was visualized using FigTree v.1.4.2 (http://tree.bio.ed.ac.uk/software/figtree/).

### Serotype identification.

*In silico* serotype identification was performed on the assembled genomes using the online Center for Genomic Epidemiology SerotypeFinder 1.1 tool (https://cge.cbs.dtu.dk/services/SerotypeFinder/) (61, 62).

### Multilocus sequence typing (MLST).

*In silico* MLST was performed on the assembled genomes using the Achtman E. coli MLST scheme (63). Gene sequences were identified in the isolate genomes using BLASTN, and MLST profiles were determined by querying the PubMLST database (http://pubmlst.org).

### Typing of the *fimH* gene in the ST131 isolates.

*In silico fimH* typing was performed on the assembled genomes from ST131 isolates using the online Center for Genomic Epidemiology FimTyper 1.0 tool (https://cge.cbs.dtu.dk/services/FimTyper/) (64).

### Variation in gene distributions as a measurement of diversity of resident E. coli strains.

The gene contents across all genomes were identified and compared using the large-scale BLAST score ratio (LS-BSR) as previously described (65). Genes with a BSR value of ≥0.80 are considered to be highly conserved and present in the isolate examined. Those genes that were conserved in all genomes were removed from further analyses. The predicted protein function of each gene cluster was determined using an ergatis-based (66) in-house annotation pipeline (67). The data presented as heat maps were generated in MeV (Multi-Experiment Viewer) (68).

Results from LS-BSR assays of the resident E. coli isolates (523 of the 820 total genome isolates, as determined by phylogenomics and *cfaB*-negative PCR) were compared to those from reference strain E. coli UTI89 (a reference strain closely related to the resident isolates) to determine the differences between genomes with respect to the number of genes present or absent. These difference values were divided by the total number of the genes present in the query genome and multiplied by 100 to give the percentage of deviation between the query genome and the reference genome. The variation in these percentages within genomes from a single subject represents the relative level of genetic diversity from within each subject.

### Virulence factor and antibiotic resistance gene identification.

The list of common E. coli virulence factor genes used for interrogation of the study genomes is shown in Table S4. Antibiotic resistance genes were identified in the isolate genomes using the Comprehensive Antibiotic Resistance Database (version 1.1.8; http://arpcard.mcmaster.ca) Resistance Gene Identifier (version 3.2.0) (CARD-RGI) and strict and perfect cutoff values. An average number of genes per time period was calculated by dividing the total number of genes identified in all of the genomes of isolates within a time period but from a single subject by the total number of isolates from that time period. These averages are presented as a heat map generated in MeV (MultiExperiment Viewer) (68).

### Confirmation of ciprofloxacin resistance.

Five isolates from each subject were randomly chosen from day −1 (prechallenge) and day 6 (postchallenge). Following overnight growth on LB agar at 37°C, the colonies were transferred to LB agar containing ciprofloxacin (30 µg/µl) and allowed to grow overnight at 37°C. Positive growth on ciprofloxacin was indicated by the presence of at least 10 colonies following overnight growth.

### Accession number(s).

GenBank accession numbers for the sequences determined in this work are provided in Table S2.
