# Supplementary figures and images for: Responses of the Human Gut Escherichia coli Population to Pathogen and Antibiotic Disturbances
Source: mSystems. 2018 Jul 24;3(4):e00047-18. doi: 10.1128/mSystems.00047-18 (PMC6060285; doi:10.1128/mSystems.00047-18)

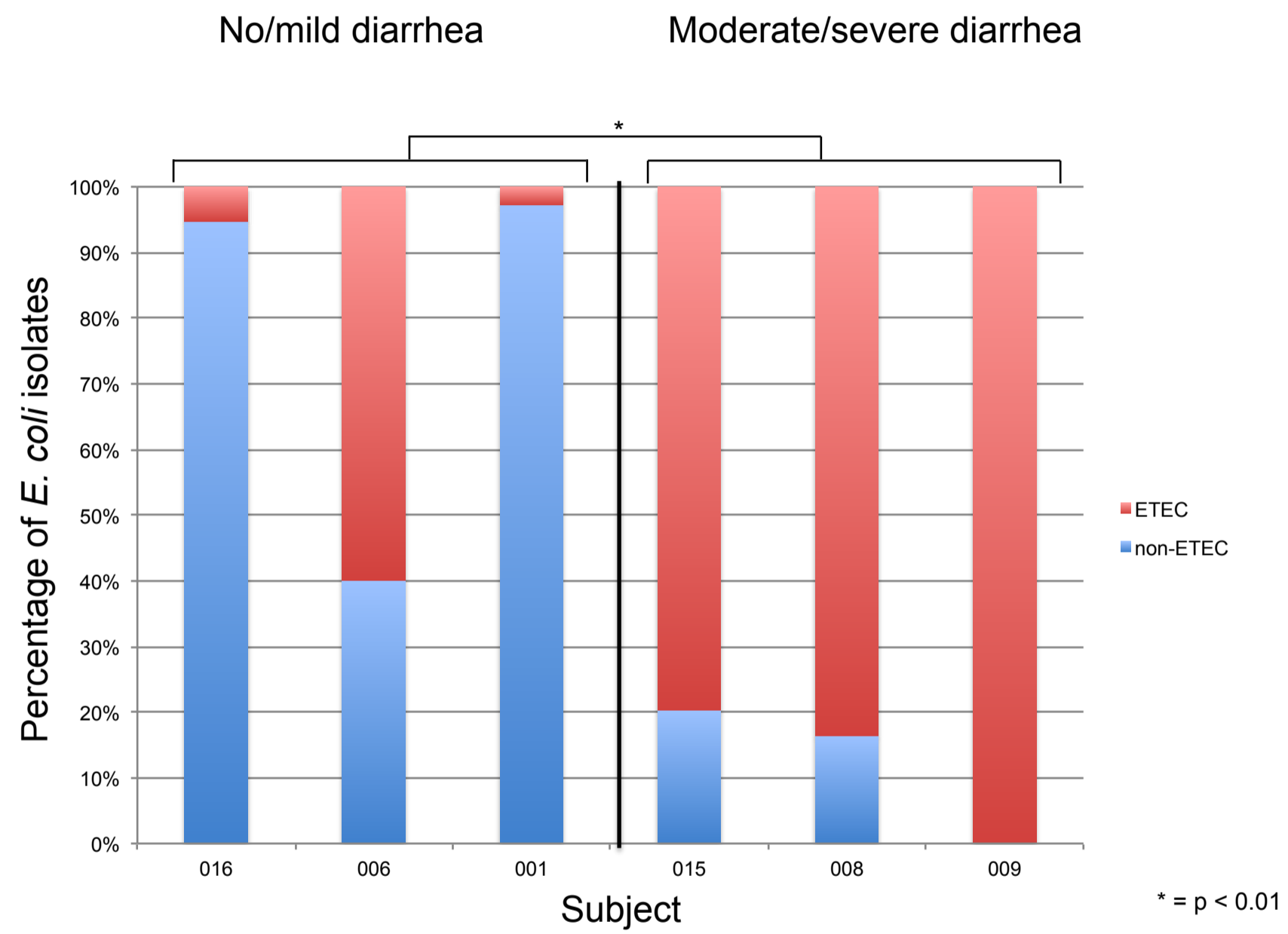

Supplement: FIG S1 [file sys004182251sf1.pdf]

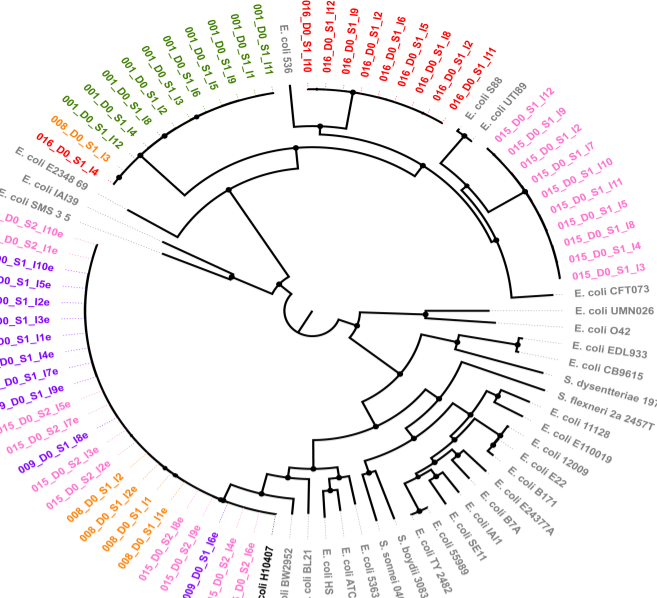

D0

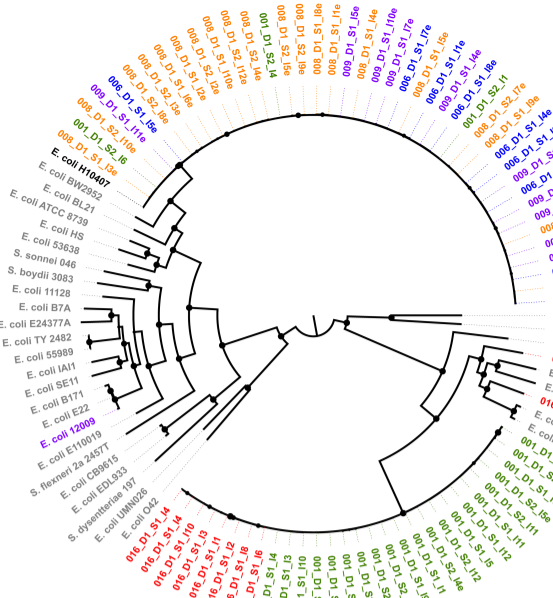

D1

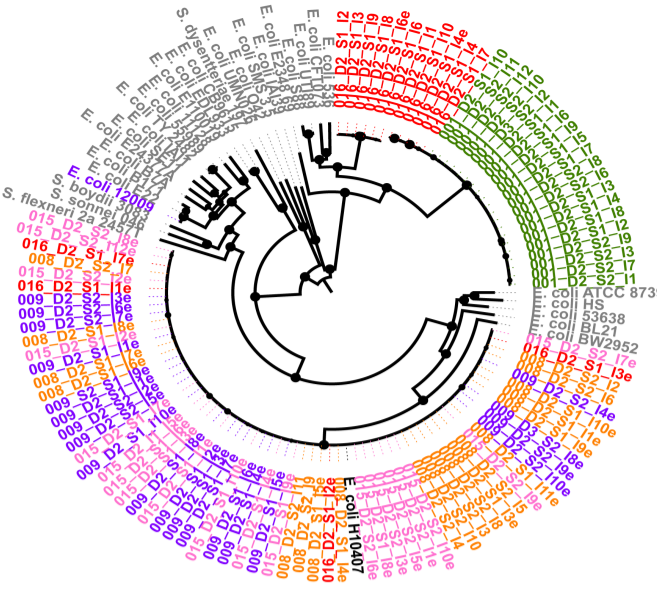

D2

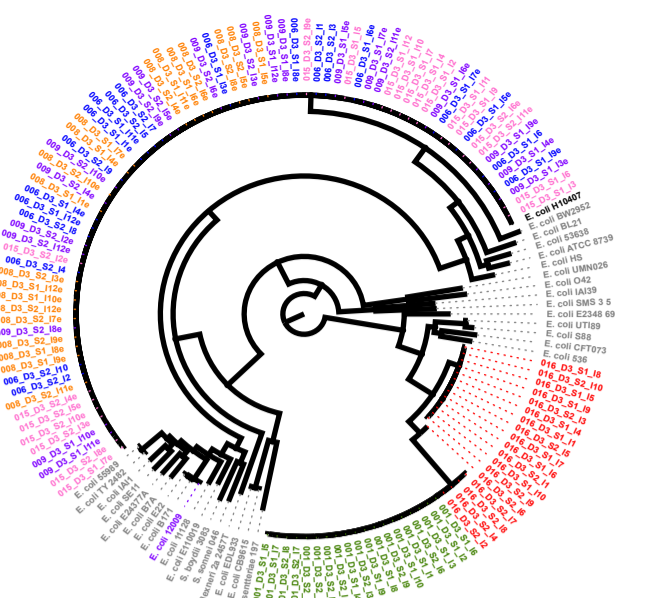

D3

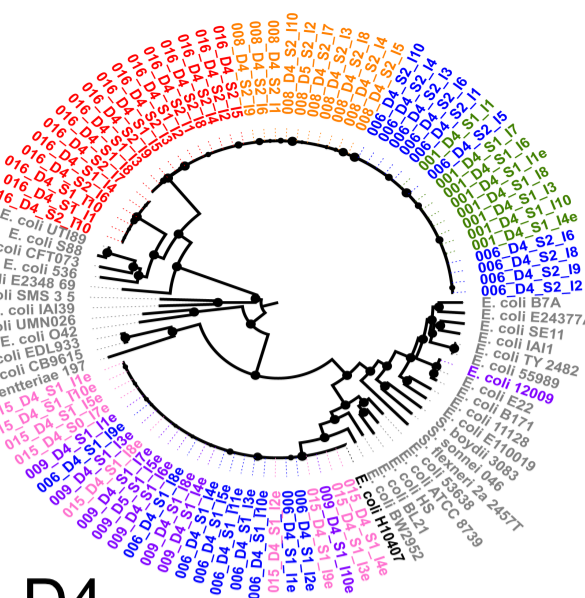

D4

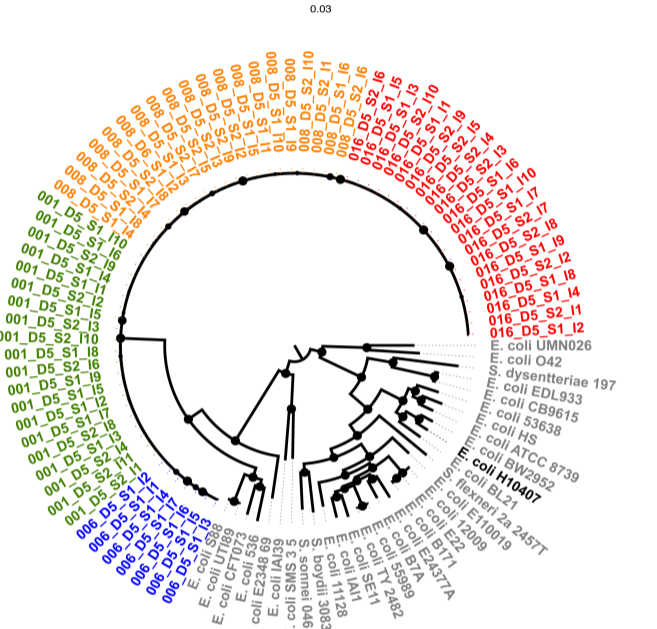

D5

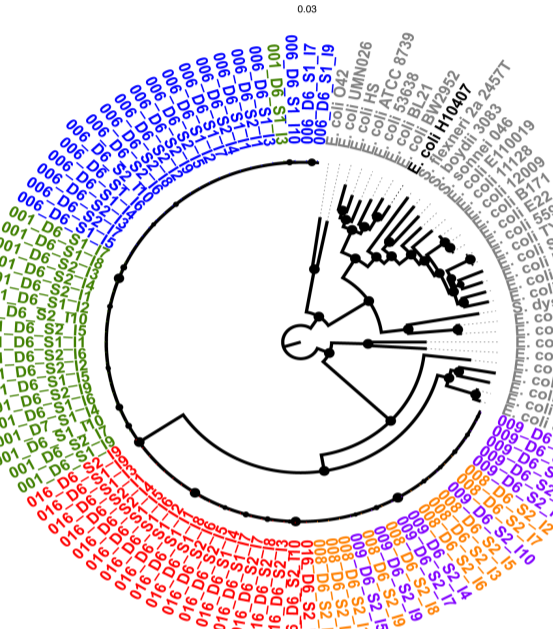

D6

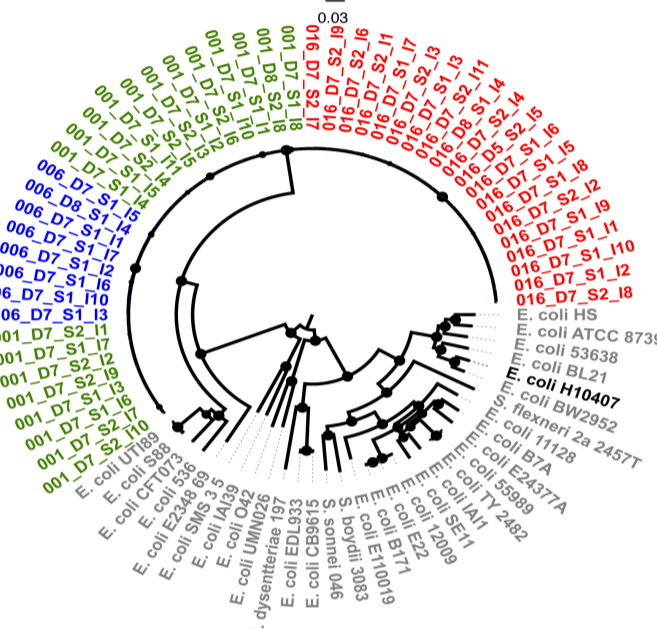

D7

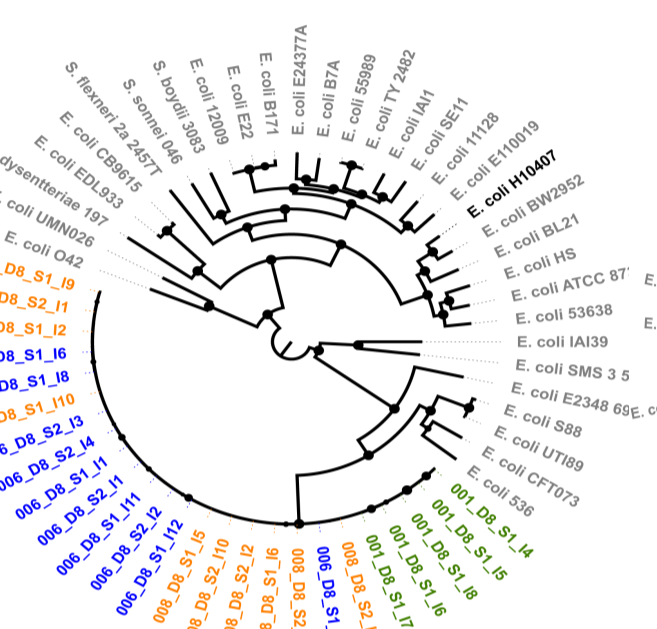

D8

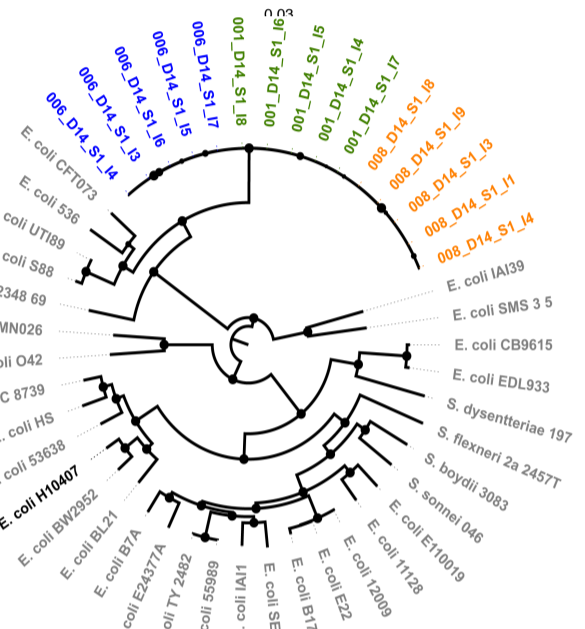

D14

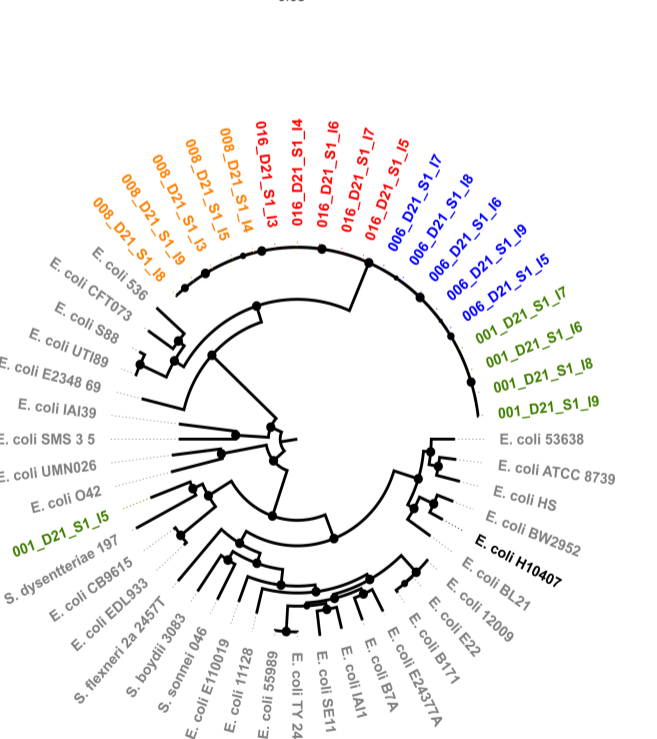

D21

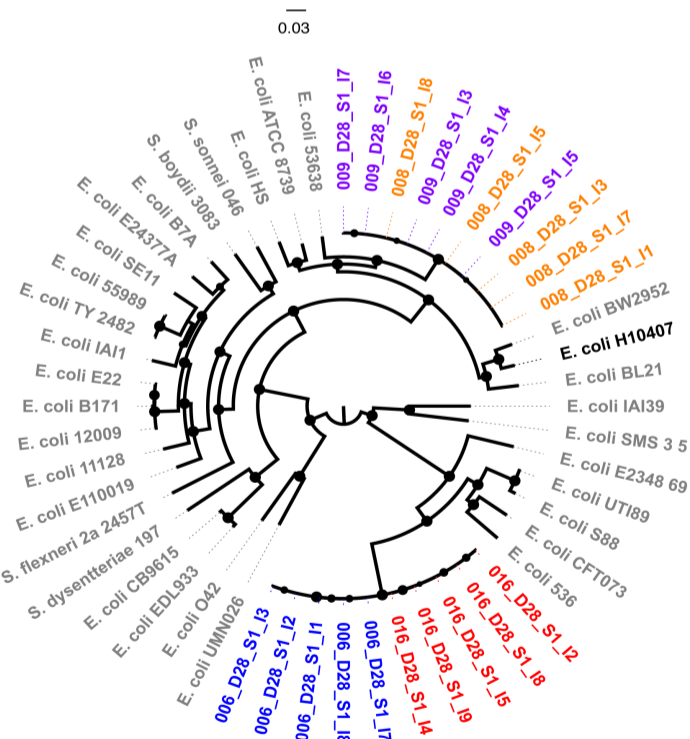

D28

Supplement: FIG S2 [file sys004182251sf2.pdf]
