# Supplementary material for: Responses of the Human Gut Escherichia coli Population to Pathogen and Antibiotic Disturbances
Source: mSystems. 2018 Jul 24;3(4):e00047-18. doi: 10.1128/mSystems.00047-18 (PMC6060285; doi:10.1128/mSystems.00047-18)
Supplement: TABLE S1 [file sys004182251st1.pdf]

Table S1. Stool sample characteristics and collection details

Subject ID	Group	Sample #	Day post challenge	Collection time	Stool grade	Isolation of H10407	Isolates available from sample	Introduction of challenge strain	Time of first antibiotic treatment
001	1	1	-1	17:30	1	pre	yes		
001	1	1	0	17:06	1	neg	yes	09:00 Day 0	
001	1	1	1	08:52	1	pos	yes		
001	1	2	1	17:35	1	neg	yes		
001	1	1	2	07:50	2	pos	yes		
001	1	2	2	10:26	2	neg	yes		
001	1	1	3	09:16	2	neg	yes		
001	1	2	3	16:56	4	neg	yes		
001	1	1	4	06:38	3	neg	yes		09:00 Day 4
001	1	2	4	11:20	4	neg	no		
001	1	1	5	08:06	3	neg	yes		
001	1	2	5	11:41	3	neg	yes		
001	1	1	6	11:02	1	neg	yes		
001	1	2	6	14:28	2	neg	yes		
001	1	1	7	09:37	2	neg	yes		
001	1	2	7	13:21	2	neg	yes		
001	1	1	8	08:12	2	neg	yes		
001	1	1	14	08:15		post	yes		
001	1	1	21	20:30		post	yes		
006	1	1	-1	09:45	1	pre	yes		
006	1	1	0	15:47	1	neg	no	09:00 Day 0	

Home

006	1	1	1	09:53	1	pos	yes	
006	1	1	2	20:50	4	pos	no	
006	1	1	3	14:22	4	pos	yes	
006	1	2	3	15:44	4	pos	yes	
006	1	1	4	11:02	4	pos	yes	09:00 Day 4
006	1	2	4	21:37	3	neg	yes	
006	1	1	5	08:45	3	neg	yes	
006	1	2	5	14:52	4	neg	no	
006	1	1	6	09:32	3	neg	yes	
006	1	2	6	18:08	3	neg	yes	
006	1	1	7	10:20	2	neg	yes	
006	1	1	8	10:12	2	neg	yes	
006	1	2	8	14:14	2	neg	yes	
006	1	1	14	09:05	1		yes	Home
006	1		21	08:00			yes	
006	1		28	08:00			yes	
008	1	1	-1	12:57	1	pre	yes	
008	1	1	0	23:47	1	pos	yes	09:00 Day 0
008	1	1	1	09:03	2	pos	yes	
008	1	2	1	13:11	2	pos	yes	
008	1	1	2	10:54	4	pos	yes	
008	1	2	2	19:10	4	pos	yes	
008	1	1	3	02:57	4		yes	
008	1	2	3	10:40	4	pos	yes	
008	1	3	3	12:10	4	pos	no	12:45 Day 3
008	1	1	4	00:57	4	pos	no	

008	1	2	4	21:08	3	neg	yes	
008	1	1	5	02:29	3	neg	yes	
008	1	2	5	15:04	2	neg	yes	
008	1	1	6	08:34	1	neg	no	
008	1	2	6	18:30	1	neg	yes	
008	1	1	7	06:38	1	neg	no	
008	1	2	7	12:36	1	neg	no	
008	1	1	8	08:04	1	neg	yes	
008	1	2	8	12:44	1	neg	yes	
008	1		14	01:00			yes	Home
008	1		21	01:00			yes	
008	1		28	08:00			yes	
009	1	1	-1	12:43	1		no	
009	1	1	0	19:12	1	pos	yes	09:00 Day 0
009	1	1	1	08:57	3	pos	yes	
009	1	2	1	09:15	3	pos	no	
009	1	1	2	05:01	4	pos	yes	
009	1	2	2	09:47	4	pos	yes	
009	1	1	3	02:57	4	pos	yes	
009	1	2	3	07:47	4	pos	yes	08:00 Day 3
009	1	1	4	00:25	3	pos	yes	
009	1	2	4	13:12	4	neg	no	
009	1	1	5	01:15	3	neg	no	
009	1	2	5	20:58	1	neg	no	
009	1	1	6	08:28	1	neg	no	
009	1	2	6	20:46	1	neg	yes	
009	1	1	7	11:03	1	neg	no	

009	1	2	7	21:24	1	neg	no	
009	1	1	8	11:02	1	neg	no	
009	1		14	21:00	1		no	Home
009	1		21	08:00			no	
009	1		28	10:50			yes	
015	1	1	-1	11:46	1		yes	
015	1	1	0	12:25	2	neg	yes	09:00 Day 0
015	1	2	0	14:12	1	pos	yes	
015	1	1	1	23:57	RS	neg	no	
015	1	1	2	08:35	1	pos	yes	
015	1	2	2	13:14	1	pos	yes	
015	1	1	3	06:58	3	pos	yes	
015	1	2	3	09:02	3	pos	yes	
015	1	1	4	08:47	4	pos	yes	9:00 Day 4
015	1	2	4	17:13	4	neg	no	
015	1	1	5	11:59	RS	neg	no	
015	1	2	5	19:55	3	neg	no	
015	1	1	6	11:41	RS	neg	no	
015	1	2	6	23:56	RS		no	
015	1	1	7	11:56	RS	neg	no	
015	1	2	7	23:44	RS	neg	no	
015	1		21	11:40			no	Home
016	1		-1	13:01	2		no	
016	1	1	0	18:50	1	neg	yes	09:00 Day 0
016	1	1	1	10:02	1	pos	yes	

016	1	1	2	08:28	1	neg	yes	
016	1	1	3	09:26	1	neg	yes	
016	1	2	3	20:24	1	neg	yes	
016	1	1	4	07:05	1	neg	yes	9:00 Day 4
016	1	2	4	11:47	1	neg	yes	
016	1	1	5	09:13	1	neg	yes	
016	1	2	5	18:13	1	neg	yes	
016	1	1	6	09:02	1	neg	yes	
016	1	2	6	21:39	1	neg	yes	
016	1	1	7	08:07	1	neg	yes	
016	1	2	7	13:07	1	neg	yes	
016	1	1	8	08:06	1	neg	no	
016	1		14	06:30			no	Home
016	1		21	07:30			yes	
016	1		28	06:30			yes	
019	2	1	-1	15:38	1		yes	
004	2	1	-1	20:00	1		yes	

RS= rectal swab, no fecal sample available
