# Supplementary material for: Responses of the Human Gut Escherichia coli Population to Pathogen and Antibiotic Disturbances
Source: mSystems. 2018 Jul 24;3(4):e00047-18. doi: 10.1128/mSystems.00047-18 (PMC6060285; doi:10.1128/mSystems.00047-18)
Supplement: TABLE S2 [file sys004182251st2.pdf]

Table S2: Genome assembly details							
Subject	Day	Sample	Isolate	Number of contigs	GC	Total length	Accession
001	-1	1	2	107	50.87	5047540	SRR4423707
001	-1	1	3	82	50.77	4992862	SRR4423702
001	-1	1	4	92	50.78	4981792	SRR4423701
001	-1	1	5	99	50.77	5007773	SRR4423679
001	-1	1	6	109	50.79	4980990	SRR4423680
001	-1	1	7	117	50.81	5016995	SRR4423677
001	-1	1	8	113	50.81	5022209	SRR4423678
001	-1	1	10	125	50.77	4978882	SRR4423710
001	-1	1	11	108	50.82	5284405	SRR4423709
001	-1	1	12	96	50.80	4997954	SRR4423708
001	0	1	1	126	50.82	5103585	SRR4423658
001	0	1	2	113	50.79	5073923	SRR4423655
001	0	1	3	94	50.79	5060517	SRR4423654
001	0	1	4	107	50.80	5077392	SRR4423651
001	0	1	5	116	50.79	5081548	SRR4423650
001	0	1	6	119	50.79	5087683	SRR4423620
001	0	1	8	106	50.80	5074270	SRR4423621
001	0	1	9	124	50.80	5081203	SRR4423632
001	0	1	11	169	50.74	5133050	SRR4423653
001	0	1	12	157	50.80	4969874	SRR4423652
001	1	1	1	127	50.81	5034426	SRR4783543
001	1	1	2	125	50.77	5061674	SRR4423601
001	1	1	3	94	50.79	4977135	SRR4423602
001	1	1	4	116	50.80	5045864	SRR4423619
001	1	1	5	155	50.82	5019488	SRR4423618
001	1	1	7	101	50.77	4988945	SRR4423617
001	1	1	9	98	50.82	5003607	SRR4783542
001	1	1	10	148	50.83	5034590	SRR4423635
001	1	1	11	128	50.81	5029021	SRR4423636
001	1	1	12	106	50.78	5014668	SRR4423637
001	1	2	1	286	50.66	5260245	SRR4423657
001	1	2	3	110	50.78	5040027	SRR4423594
001	1	2	4	235	50.68	5275216	SRR4423592
001	1	2	6	233	50.66	5355265	SRR4423596
001	1	2	8	97	50.76	5011316	SRR4423600
001	1	2	9	99	50.80	5356233	SRR4423582
001	1	2	11	101	50.82	5041539	SRR4423613
001	1	2	12	110	50.82	5069291	SRR4423612
001	1	2	1E	101	50.79	5028817	SRR4423611
001	1	2	2E	110	50.81	5017063	SRR4423593

001	1	2	3E	118	50.77	5010497	SRR4423591
001	1	2	4E	108	50.77	4926943	SRR4423597
001	1	2	5E	124	50.77	5025669	SRR4423595
001	1	2	6E	96	50.82	4974524	SRR4423599
001	2	1	1	114	50.78	5098471	SRR4423583
001	2	1	2	103	50.79	5018024	SRR4423585
001	2	1	3	102	50.75	4692454	SRR4423588
001	2	1	4	99	50.77	4990361	SRR4423587
001	2	1	5	92	50.79	4980442	SRR4423590
001	2	1	6	89	50.76	5018244	SRR4423589
001	2	1	7	98	50.79	5020482	SRR4423575
001	2	1	8	91	50.79	4978681	SRR4423576
001	2	1	9	98	50.76	5056634	SRR4423577
001	2	1	10	81	50.79	5008876	SRR4423586
001	2	2	1	110	50.79	4985906	SRR4423572
001	2	2	2	82	50.79	4970042	SRR4423570
001	2	2	3	106	50.80	5024609	SRR4423694
001	2	2	6	110	50.79	5025988	SRR4423693
001	2	2	7	96	50.78	4992568	SRR4423692
001	2	2	8	110	50.79	4980585	SRR4423691
001	2	2	9	107	50.78	5188040	SRR4423698
001	2	2	10	110	50.78	4932331	SRR4423573
001	2	2	11	109	50.69	5271950	SRR4423574
001	2	2	12	220	50.78	5110993	SRR4423569
001	3	1	1	113	50.78	5009229	SRR4423695
001	3	1	2	107	50.76	4995633	SRR4423699
001	3	1	3	104	50.80	5035074	SRR4783541
001	3	1	4	100	50.77	4974960	SRR4423644
001	3	1	5	112	50.79	5015898	SRR4423645
001	3	1	6	111	50.79	5010327	SRR4423646
001	3	1	7	93	50.79	5008408	SRR4423647
001	3	1	8	121	50.78	5047644	SRR4423640
001	3	1	9	136	50.78	4921606	SRR4423641
001	3	1	10	108	50.78	5003291	SRR4423700
001	3	2	1	97	50.81	5034631	SRR4423638
001	3	2	2	116	50.76	5041725	SRR4423666
001	3	2	3	89	50.78	4983875	SRR4423665
001	3	2	4	101	50.78	4874029	SRR4423664
001	3	2	5	106	50.77	5014079	SRR4423663
001	3	2	6	81	50.81	5007277	SRR4423670
001	3	2	7	113	50.89	5581819	SRR4423669
001	3	2	8	100	50.79	5017369	SRR4423668
001	3	2	9	114	50.80	4999832	SRR4423667

001	4	1	1	269	50.63	5236079	SRR4423607
001	4	1	3	98	50.77	5071311	SRR4423608
001	4	1	6	94	50.77	5047252	SRR4423625
001	4	1	7	106	50.79	5002438	SRR4423627
001	4	1	8	173	50.77	5101092	SRR4423629
001	4	1	10	91	50.79	5056566	SRR4423604
001	4	1	1E	167	50.75	5078911	SRR4423605
001	4	1	4E	337	50.62	5235542	SRR4423615
001	5	1	1	90	50.81	5021014	SRR4435823
001	5	1	2	128	50.73	5062524	SRR4435685
001	5	1	3	119	50.82	5199291	SRR4435686
001	5	1	4	109	50.75	5137232	SRR4435810
001	5	1	5	157	50.83	5431896	SRR4435813
001	5	1	6	109	50.77	5094125	SRR4435788
001	5	1	7	97	50.80	5089997	SRR4435822
001	5	1	8	90	50.78	4958774	SRR4435713
001	5	1	9	99	50.85	5058031	SRR4435714
001	5	1	10	122	50.80	5080867	SRR4435799
001	5	2	1	165	50.72	5099685	SRR4435793
001	5	2	2	105	50.81	5091208	SRR4435792
001	5	2	3	103	50.78	5070675	SRR4435789
001	5	2	4	111	50.79	5083322	SRR4435790
001	5	2	5	128	50.79	5115642	SRR4435797
001	5	2	6	113	50.78	5096215	SRR4435798
001	5	2	8	99	50.79	5081079	SRR4435803
001	5	2	9	108	50.78	5095764	SRR4435802
001	5	2	10	94	50.79	5084534	SRR4435794
001	5	2	11	95	50.78	5064332	SRR4435791
001	6	1	1	107	50.78	5016596	SRR4435807
001	6	1	2	104	50.78	4983128	SRR4435805
001	6	1	3	98	50.83	5003059	SRR4435804
001	6	1	4	91	50.83	4989038	SRR4435809
001	6	1	5	120	50.82	5039968	SRR4435808
001	6	1	6	88	50.72	5083844	SRR4435818
001	6	1	7	102	50.81	4991506	SRR4435819
001	6	1	8	105	50.80	5025412	SRR4435820
001	6	1	9	122	50.82	4930554	SRR4435821
001	6	1	10	118	50.79	5020244	SRR4435806
001	6	2	1	110	50.77	5004809	SRR4435816
001	6	2	2	105	50.81	4982522	SRR4435811
001	6	2	3	113	50.85	5132127	SRR4435812
001	6	2	4	127	50.81	5352043	SRR4435691
001	6	2	5	103	50.79	4944333	SRR4435690

001	6	2	6	105	50.81	5865624	SRR4435777
001	6	2	7	111	50.78	5012016	SRR4435692
001	6	2	8	120	50.81	5075122	SRR4435695
001	6	2	9	105	50.75	5048502	SRR4435694
001	6	2	10	107	50.77	5041066	SRR4435817
001	7	1	1	95	50.80	4977332	SRR4435688
001	7	1	2	129	50.81	5002137	SRR4435722
001	7	1	3	115	50.77	5107102	SRR4435719
001	7	1	4	102	50.80	5012699	SRR4435720
001	7	1	5	123	50.79	5042464	SRR4435725
001	7	1	6	106	50.76	5020047	SRR4435726
001	7	1	7	122	50.81	5038251	SRR4435723
001	7	1	8	124	50.80	5034681	SRR4435724
001	7	1	10	112	50.76	4772210	SRR4435687
001	7	1	11	96	50.87	5566452	SRR4435721
001	7	2	1	99	50.81	4988977	SRR4435744
001	7	2	2	141	50.82	5020155	SRR4435742
001	7	2	3	118	50.77	5022621	SRR4435741
001	7	2	4	240	50.64	5090134	SRR4435740
001	7	2	5	101	50.82	4991732	SRR4435739
001	7	2	6	129	50.82	5034044	SRR4435738
001	7	2	7	93	50.82	4987629	SRR4435737
001	7	2	8	119	50.80	5034210	SRR4435746
001	7	2	9	95	50.83	4995084	SRR4435745
001	7	2	10	107	50.78	5007825	SRR4435743
001	8	1	4	216	50.84	5069478	SRR4423690
001	8	1	5	312	50.86	5073752	SRR4423689
001	8	1	6	148	50.83	5035043	SRR4423688
001	8	1	7	123	50.78	5028139	SRR4423687
001	8	1	8	114	50.80	4995047	SRR4423686
001	14	1	4	229	50.82	5058417	SRR4423683
001	14	1	5	111	50.75	5027548	SRR4423682
001	14	1	6	239	50.83	5121845	SRR4423681
001	14	1	7	216	50.82	5105872	SRR4423706
001	14	1	8	101	50.76	4999549	SRR4423705
001	21	1	5	212	50.32	5504819	SRR4423673
001	21	1	6	134	50.82	5043873	SRR4423674
001	21	1	7	93	50.80	5021146	SRR4423671
001	21	1	8	202	50.83	5023594	SRR4423672
001	21	1	9	99	50.81	5017845	SRR4423603
004	-1	1	1	216	50.67	5214014	SRR4435770
004	-1	1	2	119	50.71	5270357	SRR4435772
004	-1	1	3	141	50.65	5227402	SRR4435773

004	-1	1	4	203	50.65	5207030	SRR4435774
004	-1	1	5	132	50.67	5305236	SRR4435775
004	-1	1	6	137	50.63	5432801	SRR4435776
004	-1	1	7	126	50.65	5309569	SRR4435754
004	-1	1	8	90	50.72	5321506	SRR4435753
004	-1	1	9	160	50.58	5544121	SRR4435756
004	-1	1	10	128	50.69	5344276	SRR4435771
006	-1	1	2	127	50.75	5131561	SRR4435780
006	-1	1	3	129	50.76	5145805	SRR4435779
006	-1	1	5	115	50.79	6114236	SRR4435757
006	-1	1	6	132	50.76	5150015	SRR4435758
006	-1	1	7	121	50.75	5138398	SRR4435759
006	-1	1	8	115	50.72	5175955	SRR4435760
006	-1	1	10	110	50.75	5104089	SRR4435701
006	-1	1	11	119	50.77	5160520	SRR4435782
006	-1	1	12	133	50.79	5311746	SRR4435781
006	1	1	1E	262	50.70	5249632	SRR4435711
006	1	1	2E	264	50.61	5264944	SRR4435708
006	1	1	3E	282	50.68	5282433	SRR4435824
006	1	1	4E	261	50.66	5295955	SRR4435706
006	1	1	5E	251	50.63	5261643	SRR4435707
006	1	1	6E	236	50.71	5254735	SRR4435704
006	1	1	7E	257	50.69	5254535	SRR4435705
006	1	1	8E	273	50.63	5269019	SRR4435715
006	1	1	9E	256	50.64	5278531	SRR4435716
006	3	1	11E	252	50.64	5269711	SRR4444851
006	3	1	12E	244	50.71	5277000	SRR4444852
006	3	1	1E	223	50.72	5224351	SRR4444849
006	3	1	3E	254	50.71	5318275	SRR4444850
006	3	1	4E	269	50.67	5442166	SRR4783546
006	3	1	5E	252	50.64	5308249	SRR4783545
006	3	1	6E	227	50.63	5113692	SRR4783544
006	3	1	7E	230	50.68	5225332	SRR4444843
006	3	1	8E	246	50.65	5254363	SRR4783539
006	3	1	9E	242	50.69	5219207	SRR4444844
006	3	2	1	245	50.67	5171210	SRR4444757
006	3	2	2	265	50.68	5311126	SRR4444759
006	3	2	3	314	50.65	5281151	SRR4444760
006	3	2	4	269	50.71	5263933	SRR4444761
006	3	2	5	254	50.67	5272399	SRR4444762
006	3	2	6	257	50.64	5239437	SRR4444753
006	3	2	7	252	50.67	5256098	SRR4444754
006	3	2	8	250	50.65	5260818	SRR4444834

006	3	2	9	273	50.71	5363908	SRR4444833
006	3	2	10	245	50.68	5436923	SRR4444758
006	4	1	10E	308	50.69	5279343	SRR4444838
006	4	1	11E	239	50.69	5159548	SRR4444837
006	4	1	1E	218	50.62	5225290	SRR4444840
006	4	1	2E	218	50.65	5191510	SRR4444839
006	4	1	3E	241	50.65	5232211	SRR4444842
006	4	1	4E	253	50.59	5219694	SRR4444841
006	4	1	5E	258	50.68	5266134	SRR4444855
006	4	1	8E	253	50.62	5295239	SRR4444853
006	4	1	9E	256	50.65	5306002	SRR4444854
006	4	2	1	79	50.80	4980328	SRR4444857
006	4	2	2	119	50.80	5023624	SRR4444861
006	4	2	3	97	50.77	4986336	SRR4783538
006	4	2	4	106	50.74	4607010	SRR4444862
006	4	2	5	104	50.78	5014570	SRR4444798
006	4	2	6	109	50.80	4882959	SRR4444797
006	4	2	7	105	50.80	4983018	SRR4444796
006	4	2	8	110	50.77	5007114	SRR4444795
006	4	2	9	95	50.84	5538696	SRR4444802
006	4	2	10	83	50.79	4987928	SRR4444858
006	5	1	1	121	50.72	5117575	SRR4444800
006	5	1	2	125	50.76	5111394	SRR4444799
006	5	1	3	98	50.84	5024527	SRR4444794
006	5	1	4	100	50.79	5104305	SRR4444793
006	5	1	5	113	50.79	5124052	SRR4444817
006	5	1	6	112	50.75	5122152	SRR4444818
006	5	1	7	121	50.70	5175686	SRR4444819
006	6	1	1	115	50.72	5002790	SRR4444815
006	6	1	2	119	50.77	5020329	SRR4444821
006	6	1	3	89	50.76	5027272	SRR4444822
006	6	1	4	112	50.69	5089050	SRR4444780
006	6	1	5	133	50.67	5080523	SRR4444779
006	6	1	6	143	50.72	5158204	SRR4444782
006	6	1	7	116	50.75	5054882	SRR4444781
006	6	1	8	113	50.82	5004900	SRR4444776
006	6	1	9	108	50.76	5082190	SRR4444775
006	6	1	10	130	50.78	5166928	SRR4444816
006	6	2	1	126	50.80	5049331	SRR4444774
006	6	2	2	115	50.76	5063274	SRR4444791
006	6	2	3	111	50.80	4991807	SRR4444792
006	6	2	4	109	50.70	5077541	SRR4444789
006	6	2	5	130	50.77	5056036	SRR4444790

006	6	2	6	120	50.69	5107246	SRR4444787
006	6	2	7	129	50.69	5080572	SRR4444788
006	6	2	8	122	50.72	5152874	SRR4444785
006	6	2	9	108	50.79	4951826	SRR4444786
006	6	2	10	129	50.83	5021974	SRR4444773
006	7	1	1	125	50.74	5420299	SRR4444870
006	7	1	2	100	50.96	5635306	SRR4444868
006	7	1	3	110	50.84	5024815	SRR4444867
006	7	1	4	103	50.83	5018814	SRR4444866
006	7	1	5	140	50.79	5093911	SRR4444865
006	7	1	6	97	50.75	5043078	SRR4444864
006	7	1	7	96	50.81	5048997	SRR4444863
006	7	1	8	131	50.77	5085481	SRR4444872
006	7	1	9	113	50.78	5078538	SRR4444871
006	7	1	10	110	50.77	5061006	SRR4444869
006	8	1	1	124	50.77	5039635	SRR4435749
006	8	1	2	106	50.81	5007716	SRR4435748
006	8	1	6	110	50.78	5255821	SRR4435747
006	8	1	11	148	50.81	5032614	SRR4435752
006	8	1	12	112	50.82	5154810	SRR4435751
006	8	2	1	119	50.82	4997606	SRR4435712
006	8	2	2	106	50.75	5068326	SRR4435702
006	8	2	3	114	50.76	5070325	SRR4435703
006	8	2	4	126	50.78	5257898	SRR4435700
006	8	2	8	105	50.67	5378358	SRR4435783
006	14	1	3	115	50.81	5051994	SRR4435699
006	14	1	4	121	50.74	5258421	SRR4435689
006	14	1	5	103	50.86	5059156	SRR4435693
006	14	1	6	100	50.80	5323505	SRR4435787
006	14	1	7	95	50.82	5047421	SRR4435786
006	21	1	5	108	50.70	5032529	SRR4435763
006	21	1	6	151	50.65	5377487	SRR4435764
006	21	1	7	132	50.76	5300372	SRR4435765
006	21	1	8	116	50.73	5072643	SRR4435766
006	21	1	9	109	50.81	4990262	SRR4435732
006	28	1	1	102	50.80	5009830	SRR4783540
006	28	1	2	124	50.65	5136856	SRR4783547
006	28	1	3	120	50.80	5039350	SRR4435727
006	28	1	7	125	50.65	5173998	SRR4435730
006	28	1	8	149	50.60	5002050	SRR4435729
008	-1	1	1	149	50.74	5251377	SRR4444803
008	-1	1	2	150	50.71	5213916	SRR4444809
008	-1	1	3	148	50.78	5246002	SRR4444810

008	-1	1	4	179	50.84	5249091	SRR4444807
008	-1	1	5	230	50.65	5213259	SRR4444808
008	-1	1	6	170	50.84	5127248	SRR4444811
008	-1	1	7	184	50.80	5203103	SRR4444812
008	-1	1	8	177	50.76	5332031	SRR4444768
008	-1	1	9	160	50.81	5151829	SRR4444767
008	-1	1	12	144	50.77	5107813	SRR4444804
008	0	1	1	224	50.74	5276186	SRR4444878
008	0	1	2	225	50.68	5217135	SRR4444880
008	0	1	3	172	50.75	5305993	SRR4444874
008	0	1	1E	217	50.78	5298399	SRR4444879
008	0	1	2E	220	50.71	5382909	SRR4444873
008	1	1	10E	252	50.62	5255085	SRR4451613
008	1	1	1E	253	50.56	5456905	SRR4451606
008	1	1	2E	240	50.68	5247848	SRR4451607
008	1	1	3E	226	50.67	5260926	SRR4451598
008	1	1	4E	239	50.66	5173162	SRR4451599
008	1	1	5E	249	50.71	5270501	SRR4451600
008	1	1	6E	246	50.57	5085598	SRR4451601
008	1	1	7E	251	50.70	5251805	SRR4451602
008	1	1	8E	244	50.70	5259585	SRR4451603
008	1	1	9E	244	50.69	5257043	SRR4451604
008	1	2	10E	209	50.62	5236642	SRR4451597
008	1	2	11E	235	50.66	5308552	SRR4451549
008	1	2	12E	234	50.62	5159310	SRR4451548
008	1	2	2E	293	50.62	5293940	SRR4451551
008	1	2	3E	253	50.58	5291219	SRR4451550
008	1	2	4E	250	50.67	5246787	SRR4451553
008	1	2	5E	212	50.62	5206556	SRR4451552
008	1	2	7E	233	50.61	5224602	SRR4451555
008	1	2	8E	230	50.68	5194479	SRR4451554
008	1	2	9E	241	50.69	5284879	SRR4451557
008	2	1	10E	273	50.70	5310753	SRR4451571
008	2	1	11E	261	50.69	5446149	SRR4451568
008	2	1	1E	275	50.64	5154734	SRR4451569
008	2	1	3E	215	50.72	5203559	SRR4451574
008	2	1	4E	257	50.71	5150789	SRR4451575
008	2	1	5E	247	50.71	5260354	SRR4451572
008	2	1	6E	261	50.67	5243752	SRR4451573
008	2	1	7E	276	50.65	5296113	SRR4451576
008	2	1	8E	266	50.55	5337973	SRR4451577
008	2	1	9E	266	50.65	5316642	SRR4451661
008	2	2	2	297	50.68	5303809	SRR4451664

008	2	2	3	276	50.69	5101182	SRR4451663
008	2	2	4	284	50.66	5274972	SRR4451662
008	2	2	5	305	50.66	5087116	SRR4451657
008	2	2	6	325	50.60	5284356	SRR4451656
008	2	2	7	301	50.65	5250116	SRR4451532
008	2	2	8	255	50.61	5088677	SRR4451533
008	2	2	9	212	50.61	5182197	SRR4451534
008	2	2	10	296	50.64	5249606	SRR4451658
008	2	2	11	250	50.67	5231540	SRR4451665
008	3	1	10E	243	50.72	5282126	SRR4451529
008	3	1	11E	230	50.70	5268129	SRR4451530
008	3	1	12E	228	50.74	5270697	SRR4451531
008	3	1	1E	220	50.65	5313520	SRR4451536
008	3	1	4E	240	50.66	5292489	SRR4451537
008	3	1	5E	223	50.72	5243481	SRR4451633
008	3	1	6E	207	50.65	5233344	SRR4451632
008	3	1	7E	232	50.68	5276232	SRR4451635
008	3	1	8E	231	50.69	5230006	SRR4451634
008	3	1	9E	244	50.69	5320868	SRR4451629
008	3	2	10E	270	50.53	5271441	SRR4451627
008	3	2	11E	218	50.66	5214615	SRR4451626
008	3	2	12E	221	50.67	5215121	SRR4451654
008	3	2	3E	226	50.55	5640994	SRR4451655
008	3	2	4E	225	50.69	5248889	SRR4451652
008	3	2	5E	269	50.74	5441452	SRR4451653
008	3	2	6E	259	50.70	5396341	SRR4451650
008	3	2	7E	239	50.68	5231616	SRR4451651
008	3	2	8E	240	50.65	5279252	SRR4451648
008	3	2	9E	217	50.67	5198152	SRR4451649
008	4	2	1	180	50.75	5304696	SRR4451620
008	4	2	2	190	50.72	5246328	SRR4451618
008	4	2	3	208	50.67	4568911	SRR4451617
008	4	2	4	201	50.70	5328651	SRR4451616
008	4	2	5	175	50.71	5311292	SRR4451625
008	4	2	6	192	50.74	5296385	SRR4451624
008	4	2	7	188	50.71	5273750	SRR4451636
008	4	2	8	172	50.70	5292819	SRR4451637
008	4	2	9	184	50.75	5272170	SRR4451638
008	4	2	10	200	50.71	5285368	SRR4451619
008	5	1	1	164	50.72	5197692	SRR4451641
008	5	1	2	155	50.86	5168873	SRR4451643
008	5	1	3	155	50.59	5266064	SRR4451644
008	5	1	4	163	50.72	5205558	SRR4451645

008	5	1	5	204	50.68	5270694	SRR4451559
008	5	1	6	161	50.75	5163460	SRR4451558
008	5	1	7	198	50.70	5203013	SRR4451561
008	5	1	8	149	50.74	5249592	SRR4451560
008	5	1	9	195	50.82	5120950	SRR4451563
008	5	1	10	193	50.72	5198071	SRR4451642
008	5	2	1	196	50.70	5155974	SRR4451564
008	5	2	2	181	50.77	5009294	SRR4451566
008	5	2	3	187	50.73	5292054	SRR4451540
008	5	2	4	198	50.82	5056878	SRR4451541
008	5	2	5	166	50.67	5259654	SRR4451538
008	5	2	6	154	50.74	5233042	SRR4451539
008	5	2	7	143	50.70	5188530	SRR4451544
008	5	2	8	147	50.72	5231323	SRR4451545
008	5	2	9	194	50.81	5122791	SRR4451542
008	5	2	10	175	50.83	5082776	SRR4451567
008	6	2	2	159	50.83	5151248	SRR4451588
008	6	2	3	145	50.78	5098286	SRR4451595
008	6	2	4	133	50.81	5116914	SRR4451594
008	6	2	5	150	50.83	5953152	SRR4451593
008	6	2	6	158	50.81	5092675	SRR4451592
008	6	2	7	145	50.81	5103094	SRR4451587
008	6	2	8	192	50.75	5646345	SRR4451586
008	6	2	9	187	50.82	5115593	SRR4451582
008	6	2	10	158	50.82	4918745	SRR4451590
008	6	2	11	151	50.81	5321553	SRR4451589
008	8	1	2	103	50.80	5070890	SRR4444745
008	8	1	5	326	50.58	5589259	SRR4444746
008	8	1	6	129	50.80	5067230	SRR4444747
008	8	1	9	116	50.66	5139696	SRR4444748
008	8	1	10	149	50.86	5037129	SRR4444744
008	8	2	1	123	50.84	5363008	SRR4444751
008	8	2	2	145	50.72	5102126	SRR4444824
008	8	2	3	156	50.80	5112238	SRR4444823
008	8	2	6	130	50.77	5106813	SRR4444826
008	8	2	10	132	50.69	5133225	SRR4444752
008	14	1	1	242	50.83	5127551	SRR4444827
008	14	1	3	181	50.78	5116728	SRR4444830
008	14	1	4	190	50.81	5092202	SRR4444829
008	14	1	8	195	50.75	5177563	SRR4444832
008	14	1	9	188	50.78	5137907	SRR4444831
008	21	1	3	188	50.91	5740540	SRR4444772
008	21	1	4	162	50.76	5359209	SRR4444771

008	21	1	5	194	50.73	5284218	SRR4444770
008	21	1	8	207	50.71	5256377	SRR4444769
008	21	1	9	162	50.69	5380622	SRR4444764
008	28	1	1	109	50.71	4899373	SRR4451610
008	28	1	3	125	50.69	4927609	SRR4451611
008	28	1	5	113	50.72	4905794	SRR4451608
008	28	1	7	104	50.69	4879361	SRR4451609
008	28	1	8	106	50.72	4908116	SRR4451614
009	0	1	10E	286	50.65	5397464	SRR4481811
009	0	1	1E	341	50.65	5400671	SRR4481806
009	0	1	2E	281	50.61	5304959	SRR4481805
009	0	1	3E	272	50.66	5397908	SRR4481754
009	0	1	4E	231	50.70	5234852	SRR4481753
009	0	1	5E	291	50.65	5388603	SRR4481752
009	0	1	6E	262	50.53	5317516	SRR4481751
009	0	1	7E	262	50.67	5299887	SRR4481750
009	0	1	8E	308	50.66	5362953	SRR4481749
009	0	1	9E	259	50.65	5267702	SRR4481748
009	1	1	10E	260	50.68	5261973	SRR4481723
009	1	1	11E	243	50.63	5268719	SRR4481724
009	1	1	12E	255	50.67	5249199	SRR4481727
009	1	1	2E	261	50.67	5283553	SRR4481728
009	1	1	3E	217	50.71	5238510	SRR4481698
009	1	1	4E	253	50.70	5288090	SRR4481697
009	1	1	5E	237	50.71	5339805	SRR4481700
009	1	1	6E	237	50.64	5225931	SRR4481699
009	1	1	7E	255	50.63	5259267	SRR4481702
009	1	1	9E	259	50.69	5391695	SRR4481701
009	2	1	10E	228	50.78	5220564	SRR4481689
009	2	1	1E	241	50.74	5329232	SRR4481690
009	2	1	2E	224	50.72	5287696	SRR4481691
009	2	1	3E	228	50.73	5520295	SRR4481692
009	2	1	4E	215	50.70	5274487	SRR4481685
009	2	1	5E	236	50.70	5360015	SRR4481686
009	2	1	6E	261	50.70	5366981	SRR4481687
009	2	1	7E	251	50.69	5311422	SRR4481688
009	2	1	8E	239	50.72	5278055	SRR4481693
009	2	1	9E	248	50.72	5372489	SRR4481694
009	2	2	10E	241	50.67	5267735	SRR4481818
009	2	2	1E	242	50.67	5300119	SRR4481817
009	2	2	2E	239	50.67	5329073	SRR4481824
009	2	2	3E	255	50.61	5385383	SRR4481823
009	2	2	4E	258	50.67	5454340	SRR4481822

009	2	2	5E	248	50.67	5190212	SRR4481821
009	2	2	6E	251	50.58	5405447	SRR4481816
009	2	2	7E	228	50.68	5302475	SRR4481815
009	2	2	8E	222	50.51	5428977	SRR4481803
009	2	2	9E	245	50.65	5263177	SRR4481804
009	3	1	10E	229	50.66	5243846	SRR4481799
009	3	1	11E	220	50.68	5139452	SRR4481800
009	3	1	12E	305	50.70	5285113	SRR4481797
009	3	1	3E	239	50.72	5244452	SRR4481798
009	3	1	4E	212	50.70	5219660	SRR4481795
009	3	1	5E	226	50.67	5333388	SRR4481796
009	3	1	6E	220	50.66	5215561	SRR4481792
009	3	1	7E	217	50.73	5285150	SRR4481791
009	3	1	8E	235	50.69	5120181	SRR4481794
009	3	1	9E	273	50.69	5277919	SRR4481793
009	3	2	10E	256	50.69	5298532	SRR4481788
009	3	2	11E	258	50.66	5208632	SRR4481787
009	3	2	12E	255	50.69	5288460	SRR4481790
009	3	2	2E	252	50.65	5306637	SRR4481789
009	3	2	3E	258	50.68	5332385	SRR4481786
009	3	2	4E	221	50.54	5219808	SRR4481785
009	3	2	5E	249	50.62	5270233	SRR4481695
009	3	2	6E	239	50.68	5292478	SRR4481696
009	3	2	8E	265	50.68	5343292	SRR4481759
009	3	2	9E	250	50.70	5350160	SRR4481760
009	4	1	10E	237	50.68	5279981	SRR4481763
009	4	1	1E	253	50.65	5335892	SRR4481764
009	4	1	3E	246	50.69	5371494	SRR4481707
009	4	1	4E	233	50.69	5377063	SRR4481736
009	4	1	5E	231	50.64	5277972	SRR4481735
009	4	1	6E	229	50.66	5228504	SRR4481734
009	4	1	7E	224	50.71	5237233	SRR4481755
009	4	1	8E	239	50.66	5285464	SRR4481732
009	4	1	9E	228	50.69	5594737	SRR4481731
009	6	2	3	140	50.81	5137668	SRR4481758
009	6	2	4	171	50.80	5132774	SRR4481782
009	6	2	5	147	50.83	5132047	SRR4481781
009	6	2	6	158	50.80	5079005	SRR4481780
009	6	2	7	151	50.82	5080662	SRR4481779
009	6	2	8	140	50.80	5061560	SRR4481778
009	6	2	9	155	50.79	5099234	SRR4481777
009	6	2	10	145	50.84	5077118	SRR4481771
009	6	2	11	129	50.81	5062975	SRR4481772

009	6	2	12	279	50.72	5201196	SRR4481757
009	28	1	3	127	50.71	4912149	SRR4481745
009	28	1	4	115	50.72	4905759	SRR4481721
009	28	1	5	124	50.69	4896702	SRR4481722
009	28	1	6	114	50.72	4924876	SRR4481719
009	28	1	7	122	50.80	4718817	SRR4481720
015	-1	1	1	124	50.51	5254478	SRR4481715
015	-1	1	2	111	50.50	5239323	SRR4481714
015	-1	1	3	136	50.48	5287608	SRR4481711
015	-1	1	4	103	50.46	4982803	SRR4481712
015	-1	1	6	122	50.50	5287517	SRR4481709
015	-1	1	7	88	50.52	5247680	SRR4481710
015	-1	1	8	102	50.51	5221006	SRR4481742
015	-1	1	9	135	50.52	5284751	SRR4481741
015	-1	1	10	114	50.53	5411753	SRR4481716
015	-1	1	12	117	50.52	5223895	SRR4481713
015	0	1	2	98	50.51	5245535	SRR4481733
015	0	1	3	123	50.54	5435171	SRR4481682
015	0	1	4	104	50.44	5412129	SRR4481683
015	0	1	5	84	50.49	5635695	SRR4481684
015	0	1	7	102	50.49	5837824	SRR4783449
015	0	1	8	95	50.52	5424979	SRR4783450
015	0	1	9	99	50.42	5357878	SRR4783447
015	0	1	10	100	50.49	5081452	SRR4481740
015	0	1	11	80	50.50	5214197	SRR4481739
015	0	1	12	118	50.44	5316913	SRR4481756
015	0	2	10E	282	50.65	5291804	SRR4783454
015	0	2	1E	239	50.68	5276995	SRR4783451
015	0	2	2E	294	50.67	5298717	SRR4783452
015	0	2	3E	232	50.64	5299700	SRR4783455
015	0	2	4E	237	50.61	5180052	SRR4783456
015	0	2	5E	248	50.62	5302542	SRR4783457
015	0	2	6E	239	50.68	5168897	SRR4783458
015	0	2	7E	267	50.63	5279800	SRR4783459
015	0	2	8E	257	50.64	5194234	SRR4783588
015	0	2	9E	246	50.70	5284335	SRR4783460
015	2	1	10E	260	50.72	5311797	SRR4783463
015	2	1	1E	212	50.72	5304381	SRR4783464
015	2	1	2E	250	50.70	5281386	SRR4783465
015	2	1	3E	224	50.74	5331587	SRR4783466
015	2	1	4E	243	50.68	5310702	SRR4783400
015	2	1	5E	219	50.66	5265777	SRR4783399
015	2	1	6E	212	50.73	5282697	SRR4783402

015	2	1	7E	208	50.69	5265426	SRR4783401
015	2	1	8E	237	50.69	5252229	SRR4783404
015	2	1	9E	256	50.73	5430735	SRR4783403
015	2	2	10E	260	50.67	5314097	SRR4783398
015	2	2	12E	253	50.66	5294799	SRR4783397
015	2	2	1E	290	50.70	5306436	SRR4783419
015	2	2	2E	332	50.70	5424095	SRR4783420
015	2	2	3E	320	50.71	5454757	SRR4783417
015	2	2	5E	271	50.67	5368719	SRR4783418
015	2	2	6E	285	50.67	5333328	SRR4783423
015	2	2	7E	305	50.72	5345454	SRR4783424
015	2	2	8E	310	50.76	5546884	SRR4783421
015	2	2	9E	291	50.71	5337623	SRR4783422
015	3	1	2	302	50.67	5352730	SRR4783377
015	3	1	3	344	50.61	5380134	SRR4783384
015	3	1	4	323	50.69	5347336	SRR4783383
015	3	1	5	340	50.73	5334528	SRR4783382
015	3	1	6	302	50.70	5312245	SRR4783381
015	3	1	7	239	50.71	5262054	SRR4783386
015	3	1	9	214	50.69	5186129	SRR4783385
015	3	1	10	235	50.65	5270874	SRR4783380
015	3	1	11	220	50.70	5271589	SRR4783379
015	3	1	12	294	50.64	5343365	SRR4783378
015	3	2	10E	290	50.66	5389719	SRR4783395
015	3	2	11E	285	50.71	5393520	SRR4783396
015	3	2	2E	291	50.68	5368981	SRR4783389
015	3	2	3E	286	50.62	5169063	SRR4783390
015	3	2	4E	262	50.65	5319317	SRR4783391
015	3	2	5E	298	50.62	5396671	SRR4783392
015	3	2	6E	298	50.72	5381240	SRR4783387
015	3	2	7E	288	50.71	5310152	SRR4783388
015	3	2	8E	273	50.70	5381374	SRR4783486
015	3	2	9E	278	50.68	5345843	SRR4783493
015	4	1	10E	317	50.54	5872189	SRR4783482
015	4	1	1E	289	50.73	5440534	SRR4783489
015	4	1	2E	271	50.69	5336112	SRR4783484
015	4	1	3E	295	50.63	5290874	SRR4783483
015	4	1	4E	258	50.60	5341784	SRR4783500
015	4	1	5E	303	50.71	5609994	SRR4783499
015	4	1	7E	266	50.71	5358537	SRR4783507
015	4	1	8E	271	50.73	5342978	SRR4783508
015	4	1	9E	260	50.66	5305955	SRR4783505
016	0	1	2	173	50.51	5286817	SRR4783476

016	0	1	3	175	50.52	5251854	SRR4783477
016	0	1	4	160	50.77	5367125	SRR4783478
016	0	1	5	189	50.51	5296459	SRR4783479
016	0	1	6	167	50.53	5278961	SRR4783480
016	0	1	8	211	50.56	5414002	SRR4783467
016	0	1	9	189	50.58	5302992	SRR4783468
016	0	1	10	140	50.53	5254091	SRR4783473
016	0	1	11	198	50.55	5306052	SRR4783474
016	0	1	12	196	50.51	5284710	SRR4783475
016	1	1	1	183	50.76	5299135	SRR4783435
016	1	1	2	158	50.76	5494672	SRR4783430
016	1	1	3	185	50.73	5376830	SRR4783427
016	1	1	4	168	50.74	5219156	SRR4783428
016	1	1	5	181	50.68	5292016	SRR4783433
016	1	1	6	167	50.72	5202497	SRR4783434
016	1	1	7	244	50.66	5259005	SRR4783431
016	1	1	8	153	50.80	5239390	SRR4783432
016	1	1	9	165	50.48	5246330	SRR4783425
016	1	1	10	139	50.75	5194976	SRR4783429
016	2	1	1	157	50.67	5333745	SRR4783491
016	2	1	2	203	50.49	5317470	SRR4783496
016	2	1	3	184	50.54	5492720	SRR4783494
016	2	1	4	169	50.69	5375482	SRR4783498
016	2	1	6	191	50.52	5411492	SRR4783471
016	2	1	7	169	50.76	4929106	SRR4783568
016	2	1	8	174	50.50	5247438	SRR4783570
016	2	1	9	209	50.51	5320118	SRR4783664
016	2	1	10	137	50.71	5265486	SRR4783490
016	2	1	1E	330	50.62	5347753	SRR4783481
016	2	1	2E	329	50.49	6121027	SRR4783487
016	2	1	3E	321	50.66	5406970	SRR4783485
016	2	1	4E	153	50.75	5240601	SRR4783497
016	2	1	6E	196	50.49	5222936	SRR4783472
016	2	1	7E	293	50.68	5299765	SRR4783571
016	3	1	1	167	50.53	5226705	SRR4783563
016	3	1	4	149	50.54	5221424	SRR4783659
016	3	1	5	166	50.52	5262836	SRR4783584
016	3	1	6	149	50.54	4843741	SRR4783662
016	3	1	7	159	50.55	5251745	SRR4783661
016	3	1	8	142	50.52	5247079	SRR4783656
016	3	1	9	158	50.50	5255394	SRR4783655
016	3	1	10	149	50.51	5229206	SRR4783562
016	3	2	1	176	50.54	5295499	SRR4783591

016	3	2	2	172	50.57	5319487	SRR4783556
016	3	2	3	156	50.54	5274384	SRR4783557
016	3	2	4	186	50.71	5228136	SRR4783558
016	3	2	5	207	50.54	5337817	SRR4783559
016	3	2	7	252	50.63	5082133	SRR4783553
016	3	2	8	151	50.72	5319983	SRR4783554
016	3	2	9	164	50.53	5309182	SRR4783555
016	3	2	10	149	50.58	5253339	SRR4783653
016	4	1	1	155	50.50	5235381	SRR4783532
016	4	1	2	401	50.60	5510728	SRR4783530
016	4	1	3	143	50.75	5286327	SRR4783529
016	4	1	4	154	50.53	5246525	SRR4783536
016	4	1	6	290	50.61	5570580	SRR4783534
016	4	1	8	422	50.54	5474343	SRR4783528
016	4	1	10	168	50.55	5300365	SRR4783531
016	4	2	1	158	50.77	5770227	SRR4783595
016	4	2	2	200	50.69	5492396	SRR4783537
016	4	2	3	175	50.74	5327521	SRR4783599
016	4	2	4	176	50.78	5254085	SRR4783551
016	4	2	5	148	50.74	5307921	SRR4783597
016	4	2	6	183	50.53	5322218	SRR4783598
016	4	2	7	182	50.61	5449672	SRR4783601
016	4	2	8	174	50.70	5398025	SRR4783602
016	4	2	9	149	50.70	5269022	SRR4783575
016	4	2	10	157	50.50	5293288	SRR4783593
016	5	1	1	192	50.71	5291140	SRR4783576
016	5	1	2	192	50.71	5272117	SRR4783578
016	5	1	3	215	50.75	5257263	SRR4783592
016	5	1	4	192	50.68	5504606	SRR4783580
016	5	1	5	216	50.77	5294020	SRR4783600
016	5	1	6	221	50.74	5259496	SRR4783594
016	5	1	7	207	50.71	5292173	SRR4783604
016	5	1	8	195	50.71	5315481	SRR4783605
016	5	1	9	212	50.74	5276219	SRR4783606
016	5	1	10	181	50.71	5244611	SRR4783579
016	5	2	1	155	50.75	5237219	SRR4783564
016	5	2	2	210	50.75	5254214	SRR4783619
016	5	2	3	203	50.75	5220994	SRR4783620
016	5	2	4	166	50.70	5331946	SRR4783621
016	5	2	5	170	50.72	5345269	SRR4783658
016	5	2	6	219	50.69	5349033	SRR4783616
016	5	2	7	192	50.73	5295339	SRR4783615
016	5	2	8	192	50.73	5295338	SRR4783590

016	5	2	9	176	50.67	5432653	SRR4783663
016	5	2	10	212	50.70	5387478	SRR4783618
016	6	1	1	163	50.67	5414481	SRR4783610
016	6	1	2	178	50.77	5366276	SRR4783608
016	6	1	3	198	50.87	5996067	SRR4783525
016	6	1	4	166	50.74	5329648	SRR4783526
016	6	1	5	191	50.82	6084265	SRR4783523
016	6	1	6	181	50.83	6323598	SRR4783524
016	6	1	7	139	50.75	5241734	SRR4783513
016	6	1	8	179	50.70	5660726	SRR4783521
016	6	1	9	163	50.82	5636365	SRR4783511
016	6	1	10	139	50.70	5329332	SRR4783633
016	6	2	1	222	50.73	5273839	SRR4783519
016	6	2	2	180	50.72	5375356	SRR4783642
016	6	2	3	186	50.75	5270827	SRR4783641
016	6	2	4	188	50.75	5241213	SRR4783644
016	6	2	5	195	50.74	5240094	SRR4783643
016	6	2	6	215	50.72	5292798	SRR4783549
016	6	2	7	272	50.83	5951055	SRR4783637
016	6	2	8	215	50.73	5458436	SRR4783640
016	6	2	9	208	50.75	5287765	SRR4783639
016	6	2	10	191	50.70	5274302	SRR4783520
016	7	1	1	159	50.73	5227739	SRR4783654
016	7	1	2	159	50.74	5323616	SRR4783614
016	7	1	3	157	50.74	5199890	SRR4783652
016	7	1	4	140	50.74	5240464	SRR4783636
016	7	1	5	184	50.74	5376878	SRR4783613
016	7	1	6	187	50.66	5288431	SRR4783634
016	7	1	7	191	50.77	5280832	SRR4783635
016	7	1	8	175	50.71	5240525	SRR4783632
016	7	1	9	200	50.77	5833263	SRR4783609
016	7	1	10	194	50.71	5387597	SRR4783617
016	7	2	1	200	50.73	5265983	SRR4783631
016	7	2	2	185	50.70	5290978	SRR4783625
016	7	2	3	175	50.72	5256804	SRR4783624
016	7	2	4	189	50.72	5306994	SRR4783627
016	7	2	5	174	50.76	5258174	SRR4783626
016	7	2	6	167	50.74	5246001	SRR4783623
016	7	2	7	180	50.73	5269053	SRR4783622
016	7	2	8	175	50.77	5183558	SRR4783645
016	7	2	9	184	50.75	5227068	SRR4783646
016	7	2	11	184	50.71	5407760	SRR4783630
016	21	1	3	156	50.64	5326347	SRR4783587

016	21	1	4	169	50.76	5259263	SRR4783586
016	21	1	5	144	50.72	5278587	SRR4783441
016	21	1	6	180	50.67	5372505	SRR4783408
016	21	1	7	172	50.77	5226276	SRR4783407
016	28	1	2	157	50.83	5839569	SRR4783410
016	28	1	3	156	50.79	5267937	SRR4783409
016	28	1	4	179	50.78	6008467	SRR4783444
016	28	1	8	174	50.72	5373658	SRR4783443
016	28	1	9	172	50.74	5311225	SRR4783414
019	-1	1	1	115	50.63	5355629	SRR4783573
019	-1	1	2	93	50.57	4903481	SRR4783585
019	-1	1	3	201	50.61	4867312	SRR4783548
019	-1	1	4	106	50.63	5003110	SRR4783638
019	-1	1	5	99	50.60	4939470	SRR4783518
019	-1	1	6	98	50.59	4943246	SRR4783517
019	-1	1	7	65	50.58	4924163	SRR4783516
019	-1	1	8	104	50.56	4960142	SRR4783515
019	-1	1	9	96	50.55	4867809	SRR4783514
019	-1	1	12	112	50.59	5001069	SRR4783651

All	Average	178.96	50.71	5213359.44	
	Maximum	422.00	50.96	6323598.00	
	Minimum	65.00	50.32	4568911.00	
