# Supplementary material for: Responses of the Human Gut Escherichia coli Population to Pathogen and Antibiotic Disturbances
Source: mSystems. 2018 Jul 24;3(4):e00047-18. doi: 10.1128/mSystems.00047-18 (PMC6060285; doi:10.1128/mSystems.00047-18)
Supplement: TABLE S3 [file sys004182251st3.pdf]

Table S3. Reference Genomes

Strain name	Pathotype	Phylogroup	Accession
BL21	lab adapted	A	NC_012947
BW2952	lab adapted	A	NC_012759
SE11	fecal isolate	B1	NC_011415
IAI1	fecal isolate	B1	NC_011741
SMS_3_5	other	F	CP000970
HS	commensal	A	NC_009800
ATCC 8739	lab adapted	A	NC_010468
536	ExPEC	B2	NC_008253
S88	ExPEC	B2	NC_011742
UTI89	ExPEC	B2	NC_007946
CFT073	ExPEC	F	AE014075
IA139	ExPEC	D	NC_011750
UMN026	ExPEC	D	NC_011751
53638	EIEC	A	NZ_AAKB00000000
55989	EAEC	B1	NC_011748
EDL933	EHEC	E	NC_002655
11368	EHEC	B1	NC_013361
11128	EHEC	B1	NC_013364
12009	EHEC	B1	NC_013353
E2348/69	EPEC	B2	NC_011601
B171	EPEC	B1	AAJX00000000
E110019	AEEC	B1	AAJW00000000
H10407	ETEC	A	FN649414
E24377A	ETEC	B1	NC_009801
B7A	ETEC	B1	NZ_AAJT02000000
TY-2482	EAEC	B1	AFOG01000000
042	EAEC	D	FN554766
CB9615	EPEC	E	NC_013941
S. flexneri 2a 2457T	Shigella	B1	NC_004741
S. boydii 3083	Shigella	B1	NC_010658
S. sonnei 046	Shigella	B1	NC_007384
S. dysenteriae 197	Shigella	*E*	NC_007606
