# Supplementary material for: Responses of the Human Gut Escherichia coli Population to Pathogen and Antibiotic Disturbances
Source: mSystems. 2018 Jul 24;3(4):e00047-18. doi: 10.1128/mSystems.00047-18 (PMC6060285; doi:10.1128/mSystems.00047-18)
Supplement: TABLE S4 [file sys004182251st4.pdf]

Table S4: Details of diversity measurements of each isolate

Subject	Day	Sample	Isolate	Resident or ETEC-like	ST	Serotype	Phylotype
001	-1	1	2	Resident	131	O25:H4	B2
001	-1	1	3	Resident	131	O25:H4	B2
001	-1	1	4	Resident	131	O25:H4	B2
001	-1	1	5	Resident	131	O25:H4	B2
001	-1	1	6	Resident	131	O25:H4	B2
001	-1	1	7	Resident	131	O25:H4	B2
001	-1	1	8	Resident	131	O25:H4	B2
001	-1	1	10	Resident	131	O25:H4	B2
001	-1	1	11	Resident	131	O25:H4	B2
001	-1	1	12	Resident	131	O25:H4	B2
001	0	1	1	Resident	131	O25:H4	B2
001	0	1	2	Resident	131	O25:H4	B2
001	0	1	3	Resident	NM	O25:H4	B2
001	0	1	4	Resident	131	O25:H4	B2
001	0	1	5	Resident	131	O25:H4	B2
001	0	1	6	Resident	131	O25:H4	B2
001	0	1	8	Resident	131	O25:H4	B2
001	0	1	9	Resident	131	O25:H4	B2
001	0	1	11	Resident	131	O25:H4	B2
001	0	1	12	Resident	131	O25:H4	B2
001	1	1	1	Resident	131	O25:H4	B2
001	1	1	2	Resident	131	O25:H4	B2
001	1	1	3	Resident	131	O25:H4	B2
001	1	1	4	Resident	131	O25:H4	B2
001	1	1	5	Resident	131	O25:H4	B2
001	1	1	7	Resident	131	O25:H4	B2
001	1	1	9	Resident	131	O25:H4	B2
001	1	1	10	Resident	131	O25:H4	B2
001	1	1	11	Resident	131	O25:H4	B2
001	1	1	12	Resident	131	O25:H4	B2
001	1	2	1	ETEC-like	48	O78:H11	A
001	1	2	3	Resident	131	O25:H4	B2
001	1	2	4	ETEC-like	48	O78:H11	A
001	1	2	6	ETEC-like	48	O78:H11	A
001	1	2	8	Resident	131	O25:H4	B2
001	1	2	9	Resident	131	O25:H4	B2
001	1	2	11	Resident	131	O25:H4	B2
001	1	2	12	Resident	131	O25:H4	B2
001	1	2	1E	Resident	131	O25:H4	B2
001	1	2	2E	Resident	131	O25:H4	B2

001	1	2	3E	Resident	131	O25:H4	B2
001	1	2	4E	Resident	131	O25:H4	B2
001	1	2	5E	Resident	131	O25:H4	B2
001	1	2	6E	Resident	131	O25:H4	B2
001	2	1	1	Resident	131	O25:H4	B2
001	2	1	2	Resident	131	O25:H4	B2
001	2	1	3	Resident	131	O25:H4	B2
001	2	1	4	Resident	131	O25:H4	B2
001	2	1	5	Resident	131	O25:H4	B2
001	2	1	6	Resident	131	O25:H4	B2
001	2	1	7	Resident	131	O25:H4	B2
001	2	1	8	Resident	131	O25:H4	B2
001	2	1	9	Resident	131	O25:H4	B2
001	2	1	10	Resident	131	O25:H4	B2
001	2	2	1	Resident	131	O25:H4	B2
001	2	2	2	Resident	131	O25:H4	B2
001	2	2	3	Resident	131	O25:H4	B2
001	2	2	6	Resident	131	O25:H4	B2
001	2	2	7	Resident	131	O25:H4	B2
001	2	2	8	Resident	131	O25:H4	B2
001	2	2	9	Resident	131	O25:H4	B2
001	2	2	10	Resident	131	O25:H4	B2
001	2	2	11	Resident	131	O25:H4	B2
001	2	2	12	Resident	131	O25:H4	B2
001	3	1	1	Resident	131	O25:H4	B2
001	3	1	2	Resident	131	O25:H4	B2
001	3	1	3	Resident	131	O25:H4	B2
001	3	1	4	Resident	131	O25:H4	B2
001	3	1	5	Resident	131	O25:H4	B2
001	3	1	6	Resident	131	O25:H4	B2
001	3	1	7	Resident	131	O25:H4	B2
001	3	1	8	Resident	2666	O25:H4	B2
001	3	1	9	Resident	131	O25:H4	B2
001	3	1	10	Resident	131	O25:H4	B2
001	3	2	1	Resident	131	O25:H4	B2
001	3	2	2	Resident	131	O25:H4	B2
001	3	2	3	Resident	131	O25:H4	B2
001	3	2	4	Resident	131	O25:H4	B2
001	3	2	5	Resident	131	O25:H4	B2
001	3	2	6	Resident	131	O25:H4	B2
001	3	2	7	Resident	131	O25:H4	B2
001	3	2	8	Resident	131	O25:H4	B2
001	3	2	9	Resident	131	O25:H4	B2

001	4	1	1	Resident	131	O25:H4	B2
001	4	1	3	Resident	131	O25:H4	B2
001	4	1	6	Resident	131	O25:H4	B2
001	4	1	7	Resident	131	O25:H4	B2
001	4	1	8	Resident	131	O25:H4	B2
001	4	1	10	Resident	131	O25:H4	B2
001	4	1	1E	Resident	131	O25:H4	B2
001	4	1	4E	Resident	131	O25:H4	B2
001	5	1	1	Resident	131	O25:H4	B2
001	5	1	2	Resident	131	O25:H4	B2
001	5	1	3	Resident	131	O25:H4	B2
001	5	1	4	Resident	131	O25:H4	B2
001	5	1	5	Resident	131	O25:H4	B2
001	5	1	6	Resident	131	O25:H4	B2
001	5	1	7	Resident	131	O25:H4	B2
001	5	1	8	Resident	131	O25:H4	B2
001	5	1	9	Resident	131	O25:H4	B2
001	5	1	10	Resident	131	O25:H4	B2
001	5	2	1	Resident	131	O25:H4	B2
001	5	2	2	Resident	131	O25:H4	B2
001	5	2	3	Resident	131	O25:H4	B2
001	5	2	4	Resident	131	O25:H4	B2
001	5	2	5	Resident	131	O25:H4	B2
001	5	2	6	Resident	131	O25:H4	B2
001	5	2	8	Resident	131	O25:H4	B2
001	5	2	9	Resident	131	O25:H4	B2
001	5	2	10	Resident	131	O25:H4	B2
001	5	2	11	Resident	131	O25:H4	B2
001	6	1	1	Resident	131	O25:H4	B2
001	6	1	2	Resident	131	O25:H4	B2
001	6	1	3	Resident	131	O25:H4	B2
001	6	1	4	Resident	131	O25:H4	B2
001	6	1	5	Resident	131	O25:H4	B2
001	6	1	6	Resident	131	O25:H4	B2
001	6	1	7	Resident	131	O25:H4	B2
001	6	1	8	Resident	131	O25:H4	B2
001	6	1	9	Resident	131	O25:H4	B2
001	6	1	10	Resident	131	O25:H4	B2
001	6	2	1	Resident	131	O25:H4	B2
001	6	2	2	Resident	131	O25:H4	B2
001	6	2	3	Resident	131	ONT:H4	B2
001	6	2	4	Resident	131	O25:H4	B2
001	6	2	5	Resident	5603	O25:H4	B2

001	6	2	6	Resident	131	O25:H4	B2
001	6	2	7	Resident	131	O25:H4	B2
001	6	2	9	Resident	131	O25:H4	B2
001	6	2	10	Resident	131	O25:H4	B2
001	7	1	1	Resident	131	O25:H4	B2
001	7	1	2	Resident	131	O25:H4	B2
001	7	1	3	Resident	NM	O25:H4	B2
001	7	1	4	Resident	131	O25:H4	B2
001	7	1	5	Resident	131	O25:H4	B2
001	7	1	6	Resident	131	O25:H4	B2
001	7	1	7	Resident	131	O25:H4	B2
001	7	1	8	Resident	131	O25:H4	B2
001	7	1	10	Resident	NM	O25:H4	B2
001	7	1	11	Resident	131	O25:H4	B2
001	7	2	1	Resident	131	O25:H4	B2
001	7	2	2	Resident	131	O25:H4	B2
001	7	2	3	Resident	131	O25:H4	B2
001	7	2	4	Resident	131	O25:H4	B2
001	7	2	5	Resident	131	O25:H4	B2
001	7	2	6	Resident	131	O25:H4	B2
001	7	2	7	Resident	131	O25:H4	B2
001	7	2	8	Resident	131	O25:H4	B2
001	7	2	9	Resident	131	O25:H4	B2
001	7	2	10	Resident	131	O25:H4	B2
001	8	1	4	Resident	131	O25:H4	B2
001	8	1	5	Resident	131	O25:H4	B2
001	8	1	6	Resident	131	O25:H4	B2
001	8	1	7	Resident	131	O25:H4	B2
001	8	1	8	Resident	131	O25:H4	B2
001	14	1	4	Resident	131	O25:H4	B2
001	14	1	5	Resident	131	O25:H4	B2
001	14	1	6	Resident	131	O25:H4	B2
001	14	1	7	Resident	131	O25:H4	B2
001	14	1	8	Resident	131	O25:H4	B2
001	21	1	5	Resident	543	O169:H9	E
001	21	1	6	Resident	131	O25:H4	B2
001	21	1	7	Resident	131	O25:H4	B2
001	21	1	8	Resident	131	O25:H4	B2
001	21	1	9	Resident	131	O25:H4	B2
004	-1	1	1	Resident	95	O18:H7	B2
004	-1	1	2	Resident	404	O75:H5	B2
004	-1	1	3	Resident	404	O75:H5	B2
004	-1	1	4	Resident	95	O18:H7	B2

004	-1	1	5	Resident	404	O75:H5	B2
004	-1	1	6	Resident	404	O75:H5	B2
004	-1	1	7	Resident	404	O75:H5	B2
004	-1	1	8	Resident	404	O75:H5	B2
004	-1	1	9	Resident	95	O18:H7	B2
004	-1	1	10	Resident	404	O75:H5	B2
006	-1	1	2	Resident	NM	O25:H4	B2
006	-1	1	3	Resident	131	O25:H4	B2
006	-1	1	5	Resident	131	O25:H4	B2
006	-1	1	6	Resident	131	O25:H4	B2
006	-1	1	7	Resident	131	O25:H4	B2
006	-1	1	8	Resident	131	O25:H4	B2
006	-1	1	10	Resident	131	O25:H4	B2
006	-1	1	11	Resident	131	O25:H4	B2
006	-1	1	12	Resident	131	O25:H4	B2
006	1	1	1E	ETEC-like	48	O78:H11	A
006	1	1	2E	ETEC-like	48	O78:H11	A
006	1	1	3E	ETEC-like	48	O78:H11	A
006	1	1	4E	ETEC-like	48	O78:H11	A
006	1	1	5E	ETEC-like	48	O78:H11	A
006	1	1	6E	ETEC-like	48	O78:H11	A
006	1	1	7E	ETEC-like	48	O78:H11	A
006	1	1	8E	ETEC-like	48	O78:H11	A
006	1	1	9E	ETEC-like	48	O78:H11	A
006	3	1	1E	ETEC-like	48	O78:H11	A
006	3	1	3E	ETEC-like	48	O78:H11	A
006	3	1	4E	ETEC-like	48	O78:H11	A
006	3	1	5E	ETEC-like	48	O78:H11	A
006	3	1	6E	ETEC-like	5909	O78:H11	A
006	3	1	7E	ETEC-like	48	O78:H11	A
006	3	1	8E	ETEC-like	48	O78:H11	A
006	3	1	9E	ETEC-like	48	O78:H11	A
006	3	1	11E	ETEC-like	48	O78:H11	A
006	3	1	12E	ETEC-like	48	O78:H11	A
006	3	2	1	ETEC-like	48	O78:H11	A
006	3	2	2	ETEC-like	48	O78:H11	A
006	3	2	3	ETEC-like	48	O78:H11	A
006	3	2	4	ETEC-like	48	O78:H11	A
006	3	2	5	ETEC-like	48	O78:H11	A
006	3	2	6	ETEC-like	48	O78:H11	A
006	3	2	7	ETEC-like	48	O78:H11	A
006	3	2	8	ETEC-like	48	O78:H11	A
006	3	2	9	ETEC-like	48	O78:H11	A

006	3	2	10	ETEC-like	48	O78:H11	A
006	4	1	1E	ETEC-like	48	O78:H11	A
006	4	1	2E	ETEC-like	48	O78:H11	A
006	4	1	3E	ETEC-like	48	O78:H11	A
006	4	1	4E	ETEC-like	48	O78:H11	A
006	4	1	5E	ETEC-like	48	O78:H11	A
006	4	1	8E	ETEC-like	48	O78:H11	A
006	4	1	9E	ETEC-like	48	O78:H11	A
006	4	1	10E	ETEC-like	48	O78:H11	A
006	4	1	11E	ETEC-like	48	O78:H11	A
006	4	2	1	Resident	131	O25:H4	B2
006	4	2	2	Resident	131	O25:H4	B2
006	4	2	3	Resident	131	O25:H4	B2
006	4	2	4	Resident	NM	O25:H4	B2
006	4	2	5	Resident	131	O25:H4	B2
006	4	2	6	Resident	NM	O25:H4	B2
006	4	2	7	Resident	131	O25:H4	B2
006	4	2	8	Resident	131	O25:H4	B2
006	4	2	9	Resident	131	O25:H4	B2
006	4	2	10	Resident	131	O25:H4	B2
006	5	1	1	Resident	131	O25:H4	B2
006	5	1	2	Resident	131	O25:H4	B2
006	5	1	3	Resident	131	O25:H4	B2
006	5	1	4	Resident	131	O25:H4	B2
006	5	1	5	Resident	131	O25:H4	B2
006	5	1	6	Resident	131	O25:H4	B2
006	5	1	7	Resident	131	O25:H4	B2
006	6	1	1	Resident	131	O25:H4	B2
006	6	1	2	Resident	131	O25:H4	B2
006	6	1	3	Resident	131	O25:H4	B2
006	6	1	4	Resident	131	O25:H4	B2
006	6	1	5	Resident	131	O25:H4	B2
006	6	1	6	Resident	131	O25:H4	B2
006	6	1	7	Resident	131	O25:H4	B2
006	6	1	8	Resident	131	O25:H4	B2
006	6	1	9	Resident	131	O25:H4	B2
006	6	1	10	Resident	131	O25:H4	B2
006	6	2	1	Resident	131	O25:H4	B2
006	6	2	2	Resident	131	O25:H4	B2
006	6	2	3	Resident	131	O25:H4	B2
006	6	2	4	Resident	131	O25:H4	B2
006	6	2	5	Resident	131	O25:H4	B2
006	6	2	6	Resident	131	O25:H4	B2

006	6	2	7	Resident	131	O25:H4	B2
006	6	2	8	Resident	131	O25:H4	B2
006	6	2	9	Resident	131	O25:H4	B2
006	6	2	10	Resident	131	O25:H4	B2
006	7	1	1	Resident	131	O25:H4	B2
006	7	1	2	Resident	131	O25:H4	B2
006	7	1	3	Resident	131	O25:H4	B2
006	7	1	4	Resident	131	O25:H4	B2
006	7	1	5	Resident	131	O25:H4	B2
006	7	1	6	Resident	131	O25:H4	B2
006	7	1	7	Resident	131	O25:H4	B2
006	7	1	10	Resident	131	O25:H4	B2
006	8	1	1	Resident	131	O25:H4	B2
006	8	1	2	Resident	131	O25:H4	B2
006	8	1	6	Resident	131	O25:H4	B2
006	8	1	11	Resident	131	O25:H4	B2
006	8	1	12	Resident	131	O25:H4	B2
006	8	2	1	Resident	131	O25:H4	B2
006	8	2	2	Resident	131	O25:H4	B2
006	8	2	3	Resident	131	O25:H4	B2
006	8	2	4	Resident	131	O25:H4	B2
006	8	2	8	Resident	131	O25:H4	B2
006	14	1	3	Resident	131	O25:H4	B2
006	14	1	4	Resident	131	O25:H4	B2
006	14	1	5	Resident	131	O25:H4	B2
006	14	1	6	Resident	131	O25:H4	B2
006	14	1	7	Resident	131	O25:H4	B2
006	21	1	5	Resident	131	O25:H4	B2
006	21	1	6	Resident	131	O25:H4	B2
006	21	1	7	Resident	131	O25:H4	B2
006	21	1	8	Resident	131	O25:H4	B2
006	21	1	9	Resident	131	O25:H4	B2
006	28	1	1	Resident	131	O25:H4	B2
006	28	1	2	Resident	131	O25:H4	B2
006	28	1	3	Resident	131	O25:H4	B2
006	28	1	7	Resident	131	O25:H4	B2
006	28	1	8	Resident	131	O25:H4	B2
008	-1	1	6	Resident	131	O25:H4	B2
008	-1	1	7	Resident	131	O25:H4	B2
008	-1	1	8	Resident	131	O25:H4	B2
008	-1	1	9	Resident	131	O25:H4	B2
008	0	1	1	ETEC-like	48	O78:H11	A
008	0	1	2	ETEC-like	48	O78:H11	A

008	0	1	3	Resident	131	O25:H4	B2
008	0	1	1E	ETEC-like	48	O78:H11	A
008	0	1	2E	ETEC-like	48	O78:H11	A
008	1	1	1E	ETEC-like	48	O78:H11	A
008	1	1	2E	ETEC-like	48	O78:H11	A
008	1	1	3E	ETEC-like	48	O78:H11	A
008	1	1	4E	ETEC-like	48	O78:H11	A
008	1	1	5E	ETEC-like	48	O78:H11	A
008	1	1	6E	ETEC-like	48	O78:H11	A
008	1	1	7E	ETEC-like	48	O78:H11	A
008	1	1	8E	ETEC-like	5909	O78:H11	A
008	1	1	9E	ETEC-like	48	O78:H11	A
008	1	1	10E	ETEC-like	48	O78:H11	A
008	1	2	2E	ETEC-like	48	O78:H11	A
008	1	2	3E	ETEC-like	48	O78:H11	A
008	1	2	4E	ETEC-like	48	O78:H11	A
008	1	2	5E	ETEC-like	48	O78:H11	A
008	1	2	7E	ETEC-like	48	O78:H11	A
008	1	2	8E	ETEC-like	48	O78:H11	A
008	1	2	9E	ETEC-like	48	O78:H11	A
008	1	2	10E	ETEC-like	48	O78:H11	A
008	1	2	11E	ETEC-like	48	O78:H11	A
008	1	2	12E	ETEC-like	48	O78:H11	A
008	2	1	1E	ETEC-like	48	O78:H11	A
008	2	1	3E	ETEC-like	48	O78:H11	A
008	2	1	4E	ETEC-like	48	O78:H11	A
008	2	1	5E	ETEC-like	48	O78:H11	A
008	2	1	6E	ETEC-like	48	O78:H11	A
008	2	1	7E	ETEC-like	48	O78:H11	A
008	2	1	8E	ETEC-like	5909	O78:H11	A
008	2	1	9E	ETEC-like	48	O78:H11	A
008	2	1	10E	ETEC-like	48	O78:H11	A
008	2	1	11E	ETEC-like	48	O78:H11	A
008	2	2	2	ETEC-like	48	O78:H11	A
008	2	2	3	ETEC-like	NM	O78:H11	A
008	2	2	4	ETEC-like	48	O78:H11	A
008	2	2	5	ETEC-like	48	O78:H11	A
008	2	2	6	ETEC-like	48	O78:H11	A
008	2	2	7	ETEC-like	48	O78:H11	A
008	2	2	8	ETEC-like	48	O78:HNT	A
008	2	2	9	ETEC-like	48	O78:H11	A
008	2	2	10	ETEC-like	48	O78:H11	A
008	2	2	11	ETEC-like	48	O78:H11	A

008	3	1	1E	ETEC-like	48	O78:H11	A
008	3	1	4E	ETEC-like	48	O78:H11	A
008	3	1	5E	ETEC-like	48	O78:H11	A
008	3	1	6E	ETEC-like	48	O78:H11	A
008	3	1	7E	ETEC-like	48	O78:H11	A
008	3	1	8E	ETEC-like	48	O78:H11	A
008	3	1	9E	ETEC-like	48	O78:H11	A
008	3	1	10E	ETEC-like	48	O78:H11	A
008	3	1	11E	ETEC-like	48	O78:H11	A
008	3	1	12E	ETEC-like	48	O78:H11	A
008	3	2	3E	ETEC-like	48	O78:H11	A
008	3	2	4E	ETEC-like	48	O78:H11	A
008	3	2	5E	ETEC-like	48	O78:H11	A
008	3	2	6E	ETEC-like	48	O78:H11	A
008	3	2	7E	ETEC-like	48	O78:H11	A
008	3	2	8E	ETEC-like	48	O78:H11	A
008	3	2	9E	ETEC-like	48	O78:H11	A
008	3	2	10E	ETEC-like	48	O78:H11	A
008	3	2	11E	ETEC-like	48	O78:H11	A
008	3	2	12E	ETEC-like	48	O78:H11	A
008	4	2	1	Resident	131	O25:H4	B2
008	4	2	2	Resident	131	O25:H4	B2
008	4	2	3	Resident	131	O25:H4	B2
008	4	2	4	Resident	131	O25:H4	B2
008	4	2	5	Resident	131	O25:H4	B2
008	4	2	6	Resident	131	O25:H4	B2
008	4	2	7	Resident	131	O25:H4	B2
008	4	2	8	Resident	131	O25:H4	B2
008	4	2	9	Resident	131	O25:H4	B2
008	4	2	10	Resident	2666	O25:H4	B2
008	5	1	1	Resident	131	O25:H4	B2
008	5	1	2	Resident	131	O25:H4	B2
008	5	1	3	Resident	131	O25:H4	B2
008	5	1	4	Resident	131	O25:H4	B2
008	5	1	5	Resident	131	O25:H4	B2
008	5	1	6	Resident	131	O25:H4	B2
008	5	1	7	Resident	131	O25:H4	B2
008	5	1	8	Resident	131	O25:H4	B2
008	5	1	9	Resident	131	O25:H4	B2
008	5	1	10	Resident	131	O25:H4	B2
008	5	2	1	Resident	NM	O25:H4	B2
008	5	2	2	Resident	131	O25:H4	B2
008	5	2	3	Resident	131	O25:H4	B2

008	5	2	4	Resident	131	O25:H4	B2
008	5	2	5	Resident	131	O25:H4	B2
008	5	2	6	Resident	131	O25:H4	B2
008	5	2	7	Resident	131	O25:H4	B2
008	5	2	8	Resident	131	O25:H4	B2
008	5	2	9	Resident	131	O25:H4	B2
008	5	2	10	Resident	131	O25:H4	B2
008	6	2	2	Resident	131	O25:H4	B2
008	6	2	3	Resident	131	O25:H4	B2
008	6	2	4	Resident	131	O25:H4	B2
008	6	2	5	Resident	131	O25:H4	B2
008	6	2	6	Resident	131	O25:H4	B2
008	6	2	7	Resident	131	O25:H4	B2
008	6	2	8	Resident	131	O25:H4	B2
008	6	2	9	Resident	131	O25:H4	B2
008	6	2	10	Resident	131	O25:H4	B2
008	6	2	11	Resident	131	O25:H4	B2
008	8	1	2	Resident	131	O25:H4	B2
008	8	1	5	Resident	131	O25:H4	B2
008	8	1	6	Resident	131	O25:H4	B2
008	8	1	9	Resident	131	O25:H4	B2
008	8	1	10	Resident	131	O25:HNT	B2
008	8	2	1	Resident	131	O25:H4	B2
008	8	2	2	Resident	131	O25:H4	B2
008	8	2	3	Resident	131	O25:H4	B2
008	8	2	6	Resident	131	O25:H4	B2
008	8	2	10	Resident	131	O25:H4	B2
008	14	1	1	Resident	131	O25:H4	B2
008	14	1	3	Resident	131	O25:H4	B2
008	14	1	4	Resident	131	O25:H4	B2
008	14	1	8	Resident	131	O25:H4	B2
008	14	1	9	Resident	131	O25:H4	B2
008	21	1	3	Resident	131	O25:H4	B2
008	21	1	4	Resident	131	O25:H4	B2
008	21	1	5	Resident	131	O25:H4	B2
008	21	1	8	Resident	131	O25:H4	B2
008	21	1	9	Resident	131	O25:H4	B2
008	28	1	1	Resident	176	O13/O135:H30	A
008	28	1	3	Resident	176	O13/O135:H30	A
008	28	1	5	Resident	176	O13/O135:H30	A
008	28	1	7	Resident	176	O13/O135:H30	A
008	28	1	8	Resident	NM	O13/O135:H30	A
009	0	1	1E	ETEC-like	48	O78:H11	A

009	0	1	2E	ETEC-like	48	O78:H11	A
009	0	1	3E	ETEC-like	48	O78:H11	A
009	0	1	4E	ETEC-like	48	O78:H11	A
009	0	1	5E	ETEC-like	48	O78:H11	A
009	0	1	6E	ETEC-like	48	O78:H11	A
009	0	1	7E	ETEC-like	48	O78:H11	A
009	0	1	8E	ETEC-like	48	O78:H11	A
009	0	1	9E	ETEC-like	48	O78:H11	A
009	0	1	10E	ETEC-like	48	O78:H11	A
009	1	1	2E	ETEC-like	48	O78:H11	A
009	1	1	3E	ETEC-like	48	O78:H11	A
009	1	1	4E	ETEC-like	48	O78:H11	A
009	1	1	5E	ETEC-like	48	O78:H11	A
009	1	1	6E	ETEC-like	48	O78:H11	A
009	1	1	7E	ETEC-like	48	ONT:H11	A
009	1	1	9E	ETEC-like	48	O78:H11	A
009	1	1	10E	ETEC-like	48	O78:H11	A
009	1	1	11E	ETEC-like	48	O78:H11	A
009	1	1	12E	ETEC-like	48	O78:H11	A
009	2	1	1E	ETEC-like	48	O78:H11	A
009	2	1	2E	ETEC-like	48	O78:H11	A
009	2	1	3E	ETEC-like	48	O78:H11	A
009	2	1	4E	ETEC-like	48	O78:H11	A
009	2	1	5E	ETEC-like	48	O78:H11	A
009	2	1	6E	ETEC-like	48	O78:H11	A
009	2	1	7E	ETEC-like	48	O78:H11	A
009	2	1	8E	ETEC-like	48	O78:H11	A
009	2	1	9E	ETEC-like	48	O78:H11	A
009	2	1	10E	ETEC-like	48	O78:H11	A
009	2	2	1E	ETEC-like	48	O78:H11	A
009	2	2	2E	ETEC-like	48	O78:H11	A
009	2	2	3E	ETEC-like	48	O78:H11	A
009	2	2	4E	ETEC-like	48	O78:H11	A
009	2	2	5E	ETEC-like	48	O78:H11	A
009	2	2	6E	ETEC-like	48	O78:H11	A
009	2	2	7E	ETEC-like	48	O78:H11	A
009	2	2	8E	ETEC-like	48	O78:H11	A
009	2	2	9E	ETEC-like	48	O78:H11	A
009	2	2	10E	ETEC-like	48	O78:H11	A
009	3	1	3E	ETEC-like	48	O78:H11	A
009	3	1	4E	ETEC-like	48	O78:H11	A
009	3	1	5E	ETEC-like	48	O78:H11	A
009	3	1	6E	ETEC-like	48	O78:H11	A

009	3	1	7E	ETEC-like	48	O78:H11	A
009	3	1	8E	ETEC-like	48	O78:H11	A
009	3	1	9E	ETEC-like	48	O78:H11	A
009	3	1	10E	ETEC-like	48	O78:H11	A
009	3	1	11E	ETEC-like	48	O78:H11	A
009	3	1	12E	ETEC-like	48	O78:H11	A
009	3	2	2E	ETEC-like	48	O78:H11	A
009	3	2	3E	ETEC-like	48	O78:H11	A
009	3	2	4E	ETEC-like	NM	O78:H11	A
009	3	2	5E	ETEC-like	48	O78:H11	A
009	3	2	6E	ETEC-like	48	O78:H11	A
009	3	2	8E	ETEC-like	48	O78:H11	A
009	3	2	9E	ETEC-like	48	O78:H11	A
009	3	2	10E	ETEC-like	48	O78:H11	A
009	3	2	11E	ETEC-like	48	O78:H11	A
009	3	2	12E	ETEC-like	48	O78:H11	A
009	4	1	1E	ETEC-like	48	O78:H11	A
009	4	1	3E	ETEC-like	48	O78:H11	A
009	4	1	4E	ETEC-like	48	O78:H11	A
009	4	1	5E	ETEC-like	48	O78:H11	A
009	4	1	6E	ETEC-like	48	O78:H11	A
009	4	1	7E	ETEC-like	48	O78:H11	A
009	4	1	8E	ETEC-like	48	O78:H11	A
009	6	2	3	Resident	131	O25:H4	B2
009	6	2	4	Resident	131	O25:H4	B2
009	6	2	5	Resident	131	O25:H4	B2
009	6	2	6	Resident	131	O25:H4	B2
009	6	2	7	Resident	131	O25:H4	B2
009	6	2	8	Resident	131	O25:H4	B2
009	6	2	9	Resident	131	O25:H4	B2
009	6	2	10	Resident	131	O25:H4	B2
009	6	2	11	Resident	131	O25:H4	B2
009	6	2	12	Resident	131	O25:H4	B2
009	28	1	3	Resident	176	O13/O135:H30	A
009	28	1	4	Resident	176	O13/O135:H30	A
009	28	1	5	Resident	176	O13/O135:H30	A
009	28	1	6	Resident	176	O13/O135:H30	A
009	28	1	7	Resident	176	O13/O135:HNT	A
015	-1	1	1	Resident	2015	O50/O2:H14	B2
015	-1	1	2	Resident	2015	O50/O2:H14	B2
015	-1	1	3	Resident	2015	O50/O2:H14	B2
015	-1	1	4	Resident	NM	O50/O2:H14	B2
015	-1	1	6	Resident	2015	O50/O2:H14	B2

015	-1	1	7	Resident	2015	O50/O2:H14	B2
015	-1	1	8	Resident	2015	O50/O2:H14	B2
015	-1	1	9	Resident	2015	O50/O2:H14	B2
015	-1	1	10	Resident	2015	O50/O2:H14	B2
015	-1	1	12	Resident	2015	O50/O2:H14	B2
015	0	1	2	Resident	2015	O50/O2:H14	B2
015	0	1	3	Resident	2015	O50/O2:H14	B2
015	0	1	4	Resident	2015	O50/O2:H14	B2
015	0	1	5	Resident	2015	O50/O2:H14	B2
015	0	1	7	Resident	2015	O50/O2:H14	B2
015	0	1	8	Resident	NM	O50/O2:H14	B2
015	0	1	9	Resident	2015	O50/O2:H14	B2
015	0	1	10	Resident	NM	O50/O2:H14	B2
015	0	1	11	Resident	2015	O50/O2:H14	B2
015	0	1	12	Resident	2015	O50/O2:H14	B2
015	0	2	1E	ETEC-like	48	O78:H11	A
015	0	2	2E	ETEC-like	48	O78:H11	A
015	0	2	3E	ETEC-like	48	O78:H11	A
015	0	2	4E	ETEC-like	48	O78:H11	A
015	0	2	5E	ETEC-like	48	O78:H11	A
015	0	2	6E	ETEC-like	48	O78:H11	A
015	0	2	7E	ETEC-like	48	O78:H11	A
015	0	2	8E	ETEC-like	48	O78:H11	A
015	0	2	9E	ETEC-like	48	O78:H11	A
015	0	2	10E	ETEC-like	48	O78:H11	A
015	2	1	1E	ETEC-like	48	O78:H11	A
015	2	1	2E	ETEC-like	48	O78:H11	A
015	2	1	3E	ETEC-like	48	O78:H11	A
015	2	1	4E	ETEC-like	48	O78:H11	A
015	2	1	5E	ETEC-like	48	O78:H11	A
015	2	1	6E	ETEC-like	48	O78:H11	A
015	2	1	7E	ETEC-like	48	O78:H11	A
015	2	1	8E	ETEC-like	48	O78:H11	A
015	2	1	9E	ETEC-like	48	O78:H11	A
015	2	1	10E	ETEC-like	48	O78:H11	A
015	2	2	1E	ETEC-like	48	O78:H11	A
015	2	2	2E	ETEC-like	48	O78:H11	A
015	2	2	3E	ETEC-like	48	O78:H11	A
015	2	2	5E	ETEC-like	48	O78:H11	A
015	2	2	6E	ETEC-like	48	O78:H11	A
015	2	2	7E	ETEC-like	48	O78:H11	A
015	2	2	8E	ETEC-like	48	O78:H11	A
015	2	2	9E	ETEC-like	48	O78:H11	A

015	2	2	10E	ETEC-like	48	O78:H11	A
015	2	2	12E	ETEC-like	48	O78:H11	A
015	3	1	2	ETEC-like	48	O78:H11	A
015	3	1	3	ETEC-like	48	O78:H11	A
015	3	1	4	ETEC-like	48	O78:H11	A
015	3	1	5	ETEC-like	48	O78:H11	A
015	3	1	6	ETEC-like	48	O78:H11	A
015	3	1	7	ETEC-like	48	O78:H11	A
015	3	1	9	ETEC-like	48	O78:H11	A
015	3	1	10	ETEC-like	48	O78:H11	A
015	3	1	11	ETEC-like	48	O78:H11	A
015	3	1	12	ETEC-like	48	O78:H11	A
015	3	2	2E	ETEC-like	48	O78:H11	A
015	3	2	3E	ETEC-like	48	O78:H11	A
015	3	2	4E	ETEC-like	48	O78:H11	A
015	3	2	5E	ETEC-like	48	O78:H11	A
015	3	2	6E	ETEC-like	48	O78:H11	A
015	3	2	7E	ETEC-like	48	O78:H11	A
015	3	2	8E	ETEC-like	48	O78:H11	A
015	3	2	9E	ETEC-like	48	O78:H11	A
015	3	2	10E	ETEC-like	48	O78:H11	A
015	3	2	11E	ETEC-like	48	O78:H11	A
015	4	1	1E	ETEC-like	48	O78:H11	A
015	4	1	2E	ETEC-like	48	O78:H11	A
015	4	1	3E	ETEC-like	48	O78:H11	A
015	4	1	4E	ETEC-like	48	O78:H11	A
015	4	1	5E	ETEC-like	48	O78:H11	A
015	4	1	7E	ETEC-like	48	O78:H11	A
015	4	1	8E	ETEC-like	48	O78:H11	A
015	4	1	9E	ETEC-like	48	O78:H11	A
015	4	1	10E	ETEC-like	48	O78:H11	A
016	0	1	2	Resident	682	O75:H5	B2
016	0	1	3	Resident	682	O75:H5	B2
016	0	1	4	Resident	131	O75:H4	B2
016	0	1	5	Resident	682	O75:H5	B2
016	0	1	6	Resident	682	O75:H5	B2
016	0	1	8	Resident	682	O75:H5	B2
016	0	1	9	Resident	682	O75:H5	B2
016	0	1	10	Resident	682	O75:H5	B2
016	0	1	11	Resident	682	O75:H5	B2
016	0	1	12	Resident	682	O75:H5	B2
016	1	1	1	Resident	131	O25:H4	B2
016	1	1	2	Resident	131	O25:H4	B2

016	1	1	3	Resident	131	O25:H4	B2
016	1	1	4	Resident	131	O25:H4	B2
016	1	1	5	Resident	131	O25:H4	B2
016	1	1	6	Resident	131	O25:H4	B2
016	1	1	7	Resident	144	O16:H6	B2
016	1	1	8	Resident	131	O25:H4	B2
016	1	1	9	Resident	682	O75:H5	B2
016	1	1	10	Resident	131	O25:H4	B2
016	2	1	1	Resident	131	O25:H4	B2
016	2	1	2	Resident	682	O75:H5	B2
016	2	1	3	Resident	682	O75:H5	B2
016	2	1	4	Resident	131	O25:H4	B2
016	2	1	6	Resident	682	O75:H5	B2
016	2	1	7	Resident	131	O25:H4	B2
016	2	1	8	Resident	682	O75:H5	B2
016	2	1	9	Resident	682	O75:H5	B2
016	2	1	10	Resident	131	O25:H4	B2
016	2	1	1E	ETEC-like	48	O78:H11	A
016	2	1	2E	ETEC-like	48	O78:H11	A
016	2	1	3E	ETEC-like	48	O78:H11	A
016	2	1	4E	Resident	131	O25:H4	B2
016	2	1	6E	Resident	682	O75:H5	B2
016	2	1	7E	ETEC-like	48	O78:H11	A
016	3	1	1	Resident	682	O75:H5	B2
016	3	1	4	Resident	682	O75:H5	B2
016	3	1	5	Resident	682	O75:H5	B2
016	3	1	6	Resident	682	O75:H5	B2
016	3	1	7	Resident	682	O75:H5	B2
016	3	1	8	Resident	682	O75:H5	B2
016	3	1	9	Resident	682	O75:H5	B2
016	3	1	10	Resident	682	O75:H5	B2
016	3	2	1	Resident	682	O75:H5	B2
016	3	2	2	Resident	131	O25:H4	B2
016	3	2	3	Resident	682	O75:H5	B2
016	3	2	4	Resident	131	O25:H4	B2
016	3	2	5	Resident	682	O75:H5	B2
016	3	2	7	Resident	131	ONT:H4	B2
016	3	2	8	Resident	131	O25:H4	B2
016	3	2	9	Resident	682	O75:H5	B2
016	3	2	10	Resident	682	O75:H5	B2
016	4	1	1	Resident	682	O75:H5	B2
016	4	1	2	Resident	131	O75:H4	B2
016	4	1	3	Resident	131	O25:H4	B2

016	4	1	4	Resident	682	O75:H5	B2
016	4	1	6	Resident	131	O25:H4	B2
016	4	1	8	Resident	NM	O25:H4	B2
016	4	1	10	Resident	682	O75:H5	B2
016	4	2	1	Resident	131	O25:H4	B2
016	4	2	2	Resident	131	O25:H4	B2
016	4	2	3	Resident	131	O25:H4	B2
016	4	2	4	Resident	131	O25:H4	B2
016	4	2	5	Resident	131	O25:H4	B2
016	4	2	6	Resident	682	O75:H5	B2
016	4	2	7	Resident	682	O75:H5	B2
016	4	2	8	Resident	131	O25:H4	B2
016	4	2	9	Resident	131	O25:H4	B2
016	4	2	10	Resident	682	O75:H5	B2
016	5	1	1	Resident	131	O25:H4	B2
016	5	1	2	Resident	131	O25:H4	B2
016	5	1	3	Resident	131	O25:H4	B2
016	5	1	4	Resident	131	O25:H4	B2
016	5	1	5	Resident	131	O25:H4	B2
016	5	1	6	Resident	131	O25:H4	B2
016	5	1	7	Resident	131	O25:H4	B2
016	5	1	8	Resident	131	O25:H4	B2
016	5	1	9	Resident	131	O25:H4	B2
016	5	1	10	Resident	131	O25:H4	B2
016	5	2	1	Resident	131	O25:H4	B2
016	5	2	2	Resident	131	O25:H4	B2
016	5	2	3	Resident	131	O25:H4	B2
016	5	2	4	Resident	131	O25:H4	B2
016	5	2	5	Resident	131	O25:H4	B2
016	5	2	6	Resident	131	O25:H4	B2
016	5	2	7	Resident	131	O25:H4	B2
016	5	2	8	Resident	131	O25:H4	B2
016	5	2	9	Resident	131	O25:H4	B2
016	5	2	10	Resident	131	O25:H4	B2
016	6	1	1	Resident	131	O25:H4	B2
016	6	1	2	Resident	131	O25:H4	B2
016	6	1	3	Resident	131	O25:H4	B2
016	6	1	4	Resident	131	O25:H4	B2
016	6	1	5	Resident	131	O25:H4	B2
016	6	1	6	Resident	131	O25:H4	B2
016	6	1	7	Resident	131	O25:H4	B2
016	6	1	8	Resident	131	O25:H4	B2
016	6	1	9	Resident	131	O25:H4	B2

016	6	1	10	Resident	131	O25:H4	B2
016	6	2	1	Resident	131	O25:H4	B2
016	6	2	2	Resident	131	O25:H4	B2
016	6	2	3	Resident	131	O25:H4	B2
016	6	2	4	Resident	131	O25:H4	B2
016	6	2	5	Resident	131	O25:H4	B2
016	6	2	6	Resident	131	O25:H4	B2
016	6	2	7	Resident	131	O25:H4	B2
016	6	2	8	Resident	131	O25:H4	B2
016	6	2	9	Resident	131	O25:H4	B2
016	6	2	10	Resident	131	O25:H4	B2
016	7	1	1	Resident	131	O25:H4	B2
016	7	1	2	Resident	131	O25:H4	B2
016	7	1	3	Resident	131	O25:H4	B2
016	7	1	4	Resident	131	O25:H4	B2
016	7	1	5	Resident	131	O25:H4	B2
016	7	1	6	Resident	131	O25:H4	B2
016	7	1	7	Resident	131	O25:H4	B2
016	7	1	8	Resident	131	O25:H4	B2
016	7	1	9	Resident	131	O25:H4	B2
016	7	1	10	Resident	131	O25:H4	B2
016	7	2	1	Resident	131	O25:H4	B2
016	7	2	2	Resident	131	O25:H4	B2
016	7	2	3	Resident	131	O25:H4	B2
016	7	2	4	Resident	131	O25:H4	B2
016	7	2	5	Resident	131	O25:H4	B2
016	7	2	6	Resident	131	O25:H4	B2
016	7	2	7	Resident	131	O25:H4	B2
016	7	2	8	Resident	131	O25:H4	B2
016	7	2	9	Resident	131	O25:H4	B2
016	7	2	11	Resident	131	O25:H4	B2
016	21	1	3	Resident	131	O25:H4	B2
016	21	1	4	Resident	131	O25:H4	B2
016	21	1	5	Resident	131	O25:H4	B2
016	21	1	6	Resident	131	O25:H4	B2
016	21	1	7	Resident	131	O25:H4	B2
016	28	1	2	Resident	131	O25:H4	B2
016	28	1	3	Resident	131	O25:H4	B2
016	28	1	4	Resident	131	O25:H4	B2
016	28	1	8	Resident	131	O25:H4	B2
016	28	1	9	Resident	131	O25:H4	B2
019	-1	1	1	Resident	420	O46/O134:H31	B2
019	-1	1	2	Resident	420	O46/O134:H31	B2

019	-1	1	3	Resident	420	O46/O134:H31	B2
019	-1	1	4	Resident	420	O46/O134:H31	B2
019	-1	1	5	Resident	420	O46/O134:H31	B2
019	-1	1	6	Resident	420	O46/O134:H31	B2
019	-1	1	7	Resident	420	O46/O134:H31	B2
019	-1	1	8	Resident	420	O46/O134:H31	B2
019	-1	1	9	Resident	420	O46/O134:H31	B2
019	-1	1	12	Resident	420	O46/O134:H31	B2
