# Supplementary material for: Responses of the Human Gut Escherichia coli Population to Pathogen and Antibiotic Disturbances
Source: mSystems. 2018 Jul 24;3(4):e00047-18. doi: 10.1128/mSystems.00047-18 (PMC6060285; doi:10.1128/mSystems.00047-18)
Supplement: TABLE S5 [file sys004182251st5.pdf]

Table S5: Virulence factors used in LS-BSR analysis of isolate genomes

Gene name	Pathotype	Annotation	Accession
2845650_185_3	ETEC	CS6 fimbrial subunit	DQ538390.1
987P	ETEC	fimbral protein 987P	U50547.1
aafA_042	EAEC	major fimbrial subunit of aggregative adherence fimbria II AafA [Escherichia coli 042]	AAB82330.1
aatA_55989	EAEC	AatA outermembrane protein [Escherichia coli 55989]	YP_002415663.1
aatB_55989	EAEC	AatB [Escherichia coli 55989]	YP_002415662.1
aatC_55989	EAEC	AatC ATB binding protein of ABC transporter [Escherichia coli 55989]	YP_002415661.1
aatD	EAEC	part of the aat-encoded secretion system, membrane associated	NC_008460.1
aggR	EAEC	AAF (Aggregative Adherence Fimbriae) /I regulator protein [Escherichia coli]	CAA79242.1
aidA-I	ETEC	(Adhesin involved diffuse adhesin) diffuse adherence adhesin [Escherichia coli]	ADH10230.1
araC_regulator	ETEC	AraC family transcriptional regulator [Escherichia coli E24377A]	YP_001451499.1
astA	EAEC	heat-stable enterotoxin 1 [Escherichia coli]	AAA20885.1
B7A_eatA	ETEC	type V autotransporter family of secretion systems (can cross inner and outer membranes of G- bac)	NC_007635.1
BCE001_MS16_197_2_aka_TW11786	ETEC	fimbral family protein	NC_000913.3
bfpA_E2348_69	EPEC	major pilin structural unit bundlin [Escherichia coli O127:H6 str. E2348/69]	YP_002332157.1
bfpB_E2348_69	EPEC	secretin lipoprotein [Escherichia coli O127:H6 str. E2348/69]	YP_002332159.1
boa	Salmonella	Protease from Salmonella bongori	AY876285
cah	STEC	putative adhesin (calcium binding antigen 43 homolog) [Escherichia coli O157:H7 str. EDL933]	AAG55356.1
cfai	ETEC	adhesin/fimbrial subunit B	NC_014234.1
Cif0.81_B171	EPEC	T3SS secreted effector Cif-homolog [Escherichia coli O111:H-]	BAG66370.1
clpV_corr_T6SSaspV_B171	EPEC	123991-130757 Escherichia coli B171 gcontig_1112554187332 whole genome shotgun sequence	AAJX02000009.1
clpV_T6SSmetV_B171	EPEC	338385-341030 Escherichia coli B171 gcontig_1112554187834, whole genome shotgun sequence	194426143

clpV_T6SSpheU_042	EAEC	EC042_4530		284919779
Cluster6061_4	ETEC	fimbral protein precursor	AY009096.1	
clyA	ETEC	hemolysin, pore-forming cytotoxic protein	AF240780.1	
cs1	ETEC	csoA colonization factor (adhesion)	LN870269.1	
CS1_E24377A	ETEC	csoA colonization factor (adhesion)	CP000800.1	
cs12	ETEC	fimbria major subunit protein precursor	AY009096.1	
cs12_like	ETEC	fimbria major subunit protein precursor	AJ488511.1	
cs13	ETEC	pilin protein	X71971.1	
cs14	ETEC	fimbrial protein	AY283611.1	
cs15	ETEC	adhesin protein	X64623.1	
cs17	ETEC	fimbrial subunit	AY515609.1	
cs18	ETEC	fimbrial protein	AF335469.1	
cs19	ETEC	major fimbrial subunit	AY288101.1	
cs2	ETEC	fimbrial subunit	Z47800.1	
cs20	ETEC	fimbria major subunit protein	AF438155.1	
cs21	ETEC	pilus structural subunit	LN870270.2	
cs22	ETEC	adhesin protein	AF145205.1	
cs26	ETEC	fimbrial protein	HQ203050.1	
cs27a	ETEC	fimbrial protein	HQ203047.1	
cs27b	ETEC	fimbrial protein	HQ203048.1	
cs28a	ETEC	fimbrial protein	HQ203049.1	
cs28b	ETEC	fimbrial protein	HQ203046.1	
cs3	ETEC	cstA colonization factor (adhesion)	X16944.1	
cs4	ETEC	fimbrial protein	AY281092.1	
cs5	ETEC	fimbral subunit	AJ224079.2	
cs6	ETEC	fimbral subunit	U04844.1	
cs7	ETEC	fimbria major subunit protein	AY009095.1	
cs8	ETEC	pilus assembly protein; CFA/III	EU107088.1	
eaaA	various	serine proteiase autotransporter of Enterobacteraceae (SPATE)	AAF63237.1	
eae_E2348_69	EPEC	EAE_ECO27 RecName: Full=Intimin; AltName: Full=Attaching and effacing	P19809.2	

		protein; Short=Eae protein	
eaeH	ETEC	attaching and effacing protein	DQ109813.1
eatA	ETEC	type V autotransporter family of secretion systems (can cross inner and outer membranes of G- bac) [Escherichia coli ETEC H10407]	AAO17297.1
ehaA	STEC	autotransporter	CP012802.1
ehaB	STEC	putative structural/membrane protein	CP012802.1
ehaC	STEC	adhesin	CP012802.1
ehaD	STEC	putative ATP-binding component of a transport system	CP012802.1
ehaJ	STEC	AidA autotransporter-like protein [Escherichia coli O127:H6 str. E2348/69]	CAS10252.1
ehxA_pO157_EDL933	EHEC	hemolysin toxin protein [Escherichia coli O157:H7 str. EDL933]	AAC70116.1
eltA_E24377A	ETEC	heat-labile enterotoxin A [Escherichia coli E24377A]	YP_001451390.1
epeA	AEEC	Autotransporter protease [Escherichia coli]	AAL18821.1
esaR	AEEC	transcriptional regulator	NZ_CYFC01000013
escV_E2348_69	EPEC	translocator EscV [Escherichia coli O127:H6 str. E2348/69]	YP_002331411.1
EspB_E2348_69	EPEC	translocon EspB [Escherichia coli O127:H6 str. E2348/69]	CAS11482.1
EspB_Sakai	EHEC	ECs4554 EspB [Escherichia coli O157:H7 str. Sakai]	NP_312581.1
EspB3.10_B171	EPEC	translocon EspB [Escherichia coli O111:H-]	BAG66729.1
espC	AEEC	enterotoxin EspC [Escherichia coli]	AAG37043.1
EspF_E2348_69	EPEC	LEE-encoded effector EspF [Escherichia coli O127:H6 str. E2348/69]	CAS11478.1
EspF1_Sakai	EHEC	ECs4550 EspF [Escherichia coli O157:H7 str. Sakai]	NP_312577.1
EspF3.10_B171	EPEC	LEE-encoded effector EspF [Escherichia coli O111:H-]	BAG66725.1
EspG_E2348_69	EPEC	LEE-encoded effector EspG [Escherichia coli O127:H6 str. E2348/69]	CAS11518.1
EspG_Sakai	EHEC	ECs4590 EspG [Escherichia coli O157:H7 str. Sakai]	NP_312617.1
EspG2_E2348_69	EPEC	T3SS secreted effector EspG homolog [Escherichia coli O127:H6 str. E2348/69]	CAS10464.1
EspG3.10_B171	EPEC	LEE-encoded effector EspG [Escherichia coli O111:H-]	BAG66764.1
EspH_E2348_69	EPEC	LEE-encoded effector EspH [Escherichia coli O127:H6 str. E2348/69]	CAS11492.1
EspH_Sakai	EHEC	hypothetical protein ECs4564 [Escherichia coli O157:H7 str. Sakai]	NP_312591.1
EspH3.10_B171	EPEC	LEE-encoded effector EspH [Escherichia coli O111:H-]	BAG66739.1
espI	AEEC	hypothetical protein [Escherichia coli]	CAC39288.1

EspJ_E2348_69	EPEC	T3SS secreted effector EspJ homolog [Escherichia coli O127:H6 str. E2348/69]	CAS08271.1
EspJ_Sakai	EHEC	hypothetical protein ECs2714 [Escherichia coli O157:H7 str. Sakai]	NP_310741.1
EspK_Sakai	EHEC	hypothetical protein ECs1568 [Escherichia coli O157:H7 str. Sakai]	NP_309595.1
EspL_E2348_69	EPEC	T3SS secreted effector EspL homolog [Escherichia coli O127:H6 str. E2348/69]	CAS10778.1
EspL1_Sakai	EHEC	hypothetical protein ECs2427 [Escherichia coli]	ACI72529.1
EspL2_Sakai	EHEC	ECs3855 enterotoxin [Escherichia coli O157:H7 str. Sakai]	NP_311882.1
EspL4_Sakai	EHEC	ECs4935 regulator of acetyl CoA synthetase [Escherichia coli O157:H7 str. Sakai]	NP_312962.1
EspL4.36_B171	EPEC	T3SS secreted effector EspL-homolog [Escherichia coli O111:H-]	BAG66854.1
EspM1_Sakai	EHEC	ECs1825 bfpT-regulated chaperone-like protein [Escherichia coli O157:H7 str. Sakai]	NP_309852.1
EspM1.18_B171	EPEC	T3SS secreted effector EspM-homolog [Escherichia coli O111:H-]	BAG66387.1
EspM2_Sakai	EHEC	ECs3485 chaperone-like protein [Escherichia coli O157:H7 str. Sakai]	NP_311512.1
EspM3.10_B171	EPEC	T3SS secreted effector EspM-homolog [Escherichia coli O111:H-]	BAG66724.1
EspN_Sakai	EHEC	hypothetical protein ECs1561 [Escherichia coli O157:H7 str. Sakai]	NP_309588.1
EspN2.21_B171	EPEC	T3SS secreted effector EspN-homolog [Escherichia coli O111:H-]	BAG66592.1
EspO1_1_Sakai	EHEC	hypothetical protein ECs1567 [Escherichia coli O157:H7 str. Sakai]	NP_309594.1
EspO1_2_Sakai	EHEC	ECs1821 hypothetical protein [Escherichia coli O157:H7 str. Sakai]	NP_309848.1
espP	AEEC	serine protease/autotransporter	NP_052685.1
EspR1_Sakai	EHEC	ECs2073 hypothetical protein [Escherichia coli]	ACI83685.1
EspR3_Sakai	EHEC	ECs2672 hypothetical protein [Escherichia coli O157:H7 str. Sakai]	BAB36095.1
EspR4_Sakai	EHEC	ECs2674 hypothetical protein [Escherichia coli O157:H7 str. Sakai]	BAB36097.1
espT	EPEC	espT protein [Escherichia coli]	CAX32481.1
espV_DEC11D	EPEC	putative T3SS effector protein EspV [Escherichia coli DEC11D]	ZP_13848083.1
EspW_Sakai	EHEC	hypothetical protein ECs3487 [Escherichia coli O157:H7 str. Sakai]	NP_311514.1
EspW1.18_B171	EPEC	T3SS secreted effector EspW-homolog [Escherichia coli O111:H-]	BAG66385.1
EspX1_Sakai	EHEC	ECs0025 hypothetical protein [Escherichia coli O157:H7 str. Sakai]	BAB33448.1
EspX2_Sakai	EHEC	ECs0876 hypothetical protein [Escherichia coli O157:H7 str. Sakai]	BAB34299.1
EspX4_Sakai	EHEC	hypothetical protein ECs5021 [Escherichia coli O157:H7 str. Sakai]	NP_313048.1
EspX5_Sakai	EHEC	hypothetical protein ECs5048 [Escherichia coli O157:H7 str. Sakai]	NP_313075.1
EspX6_Sakai	EHEC	hypothetical protein ECs5295 [Escherichia coli O157:H7 str. Sakai]	NP_313322.1

EspX7_Sakai	EHEC	ECs1560 secreted effector protein [Escherichia coli O157:H7 str. Sakai]	NP_309587.1
EspY1_Sakai	EHEC	ECs0061 hypothetical protein [Escherichia coli O157:H7 str. Sakai]	BAB33484.1
EspY2_Sakai	EHEC	ECs0073 hypothetical protein [Escherichia coli O157:H7 str. Sakai]	BAB33496.1
EspY3_Sakai	EHEC	ECs0472 hypothetical protein [Escherichia coli O157:H7 str. Sakai]	BAB33895.1
EspY4_Sakai	EHEC	hypothetical protein ECs4653 [Escherichia coli O157:H7 str. Sakai]	NP_312680.1
EspZ_E2348_69	EPEC	LEE-encoded effector EspZ [Escherichia coli O127:H6 str. E2348/69]	CAS11499.1
EspZ_Sakai	EHEC	ECs4571 SepZ [Escherichia coli O157:H7 str. Sakai]	NP_312598.1
EspZ3.10_B171	EPEC	LEE-encoded effector EspZ [Escherichia coli O111:H-]	BAG66746.1
etpA	ETEC	involved in heme utilization or adhesion	NG_036219
F17A	ETEC	fimbrial subunit precursor	AF055306.1
F41	ETEC	fimbrial protein	X14354.1
fyuA	ETEC	pesticin receptor	Z38064.1
hcp	ETEC	putative cell envelope biogenesis	AF044503.1
icsA	EIEC/ Shigella	nucleation of actin filaments	AF336792.1
ipaA	EIEC/ Shigella	type III effector	NC_002698.1
ipgD	EIEC/ Shigella	type III effector	L04309.1
irp2	ETEC	yersiniabactin biosynthetic protein	CP012633.1
K88_ab	ETEC	fimbrial protein	M29374.1
K88_ac	ETEC	fimbrial protein	M29375.1
K99	ETEC	fimbrial protein	M35282.1
leoA	ETEC	GTP binding protein	AF170971.1
Map_B171	EPEC	LEE-encoded effector Map (mitochondrial associated protein) [Escherichia coli O111:H-]	BAG66737.1
Map_E2348_69	EPEC	LEE-encoded effector Map (mitochondrial associated protein) [Escherichia coli O127:H6 str. E2348/69]	CAS11490.1
Map_Sakai	EHEC	LEE-encoded effector Map (mitochondrial associated protein) [Escherichia coli O157:H7 str. Sakai]	NP_312589.1
New_CF_type_	ETEC	putative colonization factor	AY513487.1

pfc071

NleA_E2348_69	EPEC	T3SS secreted effector NleA/EspI homolog [Escherichia coli O127:H6 str. E2348/69]	CAS08990.1
NleA_Sakai	EHEC	hypothetical protein ECs1812 [Escherichia coli O157:H7 str. Sakai]	NP_309839.1
NleA0.81_B171	EPEC	T3SS secreted effector NleA-homolog [Escherichia coli O111:H-]	BAG66372.1
NleB0.81_B171	EPEC	T3SS secreted effector NleB-homolog [Escherichia coli O111:H-]	BAG66368.1
NleB1_E2348_69	EPEC	T3SS effector-like protein NleB homolog [Escherichia coli O127:H6 str. E2348/69]	CAS08589.1
NleB1_Sakai	EHEC	hypothetical protein ECs3857 [Escherichia coli O157:H7 str. Sakai]	NP_311884.1
NleB2_1_Sakai	EHEC	ECs0846 hypothetical protein [Escherichia coli O157:H7 str. Sakai]	BAB34269.1
NleB2_E2348_69	EPEC	T3SS secreted effector NleB homolog [Escherichia coli O127:H6 str. E2348/69]	CAS10779.1
NleC_E2348_69	EPEC	T3SS secreted effector NleC homolog [Escherichia coli O127:H6 str. E2348/69]	CAS08590.1
NleC_Sakai	EHEC	ECs0847 hypothetical protein [Escherichia coli O157:H7 str. Sakai]	BAB34270.1
NleD_E2348_69	EPEC	T3SS secreted effector NleD homolog [Escherichia coli O127:H6 str. E2348/69]	CAS08592.1
NleD_Sakai	EHEC	ECs0850 hypothetical protein [Escherichia coli O157:H7 str. Sakai]	BAB34273.1
NleE_Sakai	EHEC	hypothetical protein ECs3858 [Escherichia coli O157:H7 str. Sakai]	NP_311885.1
NleE1_E2348_69	EPEC	T3SS secreted effector NleE homolog with internal deletion of 56 aa [Escherichia coli O127:H6 str. E2348/69]	CAS08628.1
NleE2_E2348_69	EPEC	T3SS secreted effector NleE homolog [Escherichia coli O127:H6 str. E2348/69]	CAS10780.1
NleE4.36_B171	EPEC	T3SS secreted effector NleE-homolog [Escherichia coli O111:H-]	BAG66853.1
NleF_E2348_69	EPEC	T3SS secreted effector NleF homolog [Escherichia coli O127:H6 str. E2348/69]	CAS08993.1
NleF_Sakai	EHEC	hypothetical protein ECs1815 [Escherichia coli O157:H7 str. Sakai]	NP_309842.1
NleF2.04_B171	EPEC	T3SS secreted effector NleF-homolog [Escherichia coli O111:H-]	BAG66565.1
NleG_E2348_69	EPEC	T3SS secreted effector NleI/NleG homolog [Escherichia coli O127:H6 str. E2348/69]	CAS08588.1
NleG_Sakai	EHEC	hypothetical protein ECs1824 [Escherichia coli O157:H7 str. Sakai]	NP_309851.1
NleG0.81_B171	EPEC	T3SS secreted effector NleG-homolog [Escherichia coli O111:H-]	BAG66371.1
NleG1.18_2_B171	EPEC	T3SS secreted effector NleG-homolog [Escherichia coli O111:H-]	BAG66386.1
NleG1.18_B171	EPEC	T3SS secreted effector NleG-homolog [Escherichia coli O111:H-]	BAG66384.1
NleG2_2_Sakai	EHEC	ECs1994 hypothetical protein [Escherichia coli O157:H7 str. Sakai]	NP_310021.1

NleG2_3_Sakai	EHEC	hypothetical protein ECs2156 [Escherichia coli O157:H7 str. Sakai]	NP_310183.1
NleG2.21_B171	EPEC	T3SS secreted effector NleG-homolog [Escherichia coli O111:H-]	BAG66593.1
NleG3.10_B171	EPEC	T3SS secreted effector NleG-homolog [Escherichia coli O111:H-]	BAG66723.1
NleG4.26_B171	EPEC	T3SS secreted effector NleG-homolog [Escherichia coli O111:H-]	BAG66806.1
NleG5_1_Sakai	EHEC	ECs1996 hypothetical protein [Escherichia coli O157:H7 str. Sakai]	NP_310023.1
NleG5_2_Sakai	EHEC	ECs2154 hypothetical protein [Escherichia coli O157:H7 str. Sakai]	NP_310181.1
NleG6_1_Sakai	EHEC	ECs1995 hypothetical protein [Escherichia coli O157:H7 str. Sakai]	BAB35418.1
NleG6_2_Sakai	EHEC	hypothetical protein ECs2155 [Escherichia coli O157:H7 str. Sakai]	NP_310182.1
NleG8_2_Sakai	EHEC	hypothetical protein ECs3486 [Escherichia coli O157:H7 str. Sakai]	NP_311513.1
NleH0.81_B171	EPEC	T3SS secreted effector NleH-homolog [Escherichia coli O111:H-]	BAG66369.1
NleH1_1_Sakai	EHEC	ECs0848 hypothetical protein [Escherichia coli O157:H7 str. Sakai]	BAB34271.1
NleH1_2_Sakai	EHEC	ECs1814 hypothetical protein ECs1814 [Escherichia coli O157:H7 str. Sakai]	NP_309841.1
NleH1_E2348_69	EPEC	T3SS secreted effector NleH homolog [Escherichia coli O127:H6 str. E2348/69]	CAS08992.1
NleH2_E2348_69	EPEC	T3SS secreted effector NleH homolog [Escherichia coli O127:H6 str. E2348/69]	CAS08266.1
OspB4.26_B171	EPEC	T3SS secreted effector OspB-homolog [Escherichia coli O111:H-]	BAG66805.1
P0299538_5_araC	ETEC	transcriptional regulator	CP000797.1
P0302293_4_174_31	ETEC	fimbrial subunit	AY513487.1
papG_CFT073	UPEC	PapG protein (adhesin) [Escherichia coli CFT073]	NP_755458.1
peaR	ETEC	AraC family transcriptional regulator (plasmid encoded) [Escherichia coli E24377A]	CP000797.1
perA_E2348	EPEC	transcriptional activator, plasmid encoded regulator	AB523681.1
pet	EAEC	serine protease pet autotransporter/enterotoxin [Escherichia coli 042]	AAC26634.1
PFC071	ETEC	major fimbrial subunit	AY513487.1
pic	many pathotypes	Pic serine protease precursor [Escherichia coli 042]	AAD23953.1
regulator	ETEC	regulatory protein [Escherichia coli]	AAA24419.1
sab	STEC	adhesin [Escherichia coli]	YP_308797.1
sat_CFT073	UPEC	AE016766_155 secreted auto transpoter toxin [Escherichia coli CFT073]	AAN82067.1
sepA	Shigella	SepA, extracellular serine protease of the IgA1 protease family, secreted by a	AAL72309.1

		C-terminal autotransporter domain [Shigella flexneri 2a str. 301]	
ShET1	EIEC/ Shigella	enterotoxin of Shigella fleneri 2a	DM147453.1
sigA	Shigella	serine protease [Shigella flexneri 2a str. 301]	AAN44449.1
sigA_Sflex2A	Shigella	serine protease [Shigella flexneri 2a str. 301]	NP_708742.1
sta1	ETEC	heat-stable enterotoxin [Escherichia coli] STa1	AAA23990.1
sta2	ETEC	heat-stable enterotoxin [Escherichia coli] STa2	M25607.1
stx1A_EDL933	EHEC	shiga-like toxin 1 subunit A encoded within prophage CP-933V [Escherichia coli O157:H7 str. EDL933]	NP_288673.1
stx1B_EDL933	EHEC	shiga-like toxin 1 subunit B encoded within prophage CP-933V [Escherichia coli O157:H7 str. EDL933]	NP_288672.1
stx2A_EDL933	EHEC	shiga-like toxin II A subunit encoded by bacteriophage BP-933W [Escherichia coli O157:H7 str. EDL933]	NP_286976.1
stx2B_EDL933	EHEC	shiga-like toxin II B subunit encoded by bacteriophage BP-933W [Escherichia coli O157:H7 str. EDL933]	NP_286977.1
TccP_Sakai	EHEC	ECs2715 EspF-like protein [Escherichia coli O157:H7 str. Sakai]	NP_310742.1
TccP1.03_B171	EPEC	T3SS secreted effector, TccP2 [Escherichia coli O111:H-]	BAG66379.1
tia	ETEC	adhesin/invasin determinant	U20318.1
tibA	ETEC	Adhesin/invasin tibA precursor (Glycoprotein tibA) [Escherichia coli ETEC H10407]	CBJ01643.1
tibA_homolog	ETEC	Adhesin/invasin tibA precursor (Glycoprotein tibA) [Escherichia coli ETEC H10407]	AF109215.1
Tir_B171	EPEC	translocated intimin receptor Tir [Escherichia coli O111:H-]	BAG66736.1
Tir_E2348_69	EPEC	translocated intimin receptor Tir [Escherichia coli O127:H6 str. E2348/69]	CAS11489.1
Tir_Sakai	EHEC	translocated intimin receptor [Escherichia coli O157:H7 str. Sakai]	NP_312588.1
tsh	many pathotypes	temperature-sensitive hemagglutinin tsh autotransporter	AAA24698.1
vat	many pathotypes	AE016756_57 Haemoglobin protease [Escherichia coli CFT073]	AAN78874.1
VirA	EIEC/ Shigella	type III effector	AY206441.1

yghJ	many pathotypes	putative lipoprotein/accessory colonization factor	CP007594.1
