# Supplementary material for: Responses of the Human Gut Escherichia coli Population to Pathogen and Antibiotic Disturbances
Source: mSystems. 2018 Jul 24;3(4):e00047-18. doi: 10.1128/mSystems.00047-18 (PMC6060285; doi:10.1128/mSystems.00047-18)
Supplement: TABLE S6 [file sys004182251st6.pdf]

Table S6: Virulence BSR results

Subect	Day	Sample	Isolate	aatD	astA_EAEC	cah	cfai	clpV_corr_T6SSaspV_B171_EPEC	clpV_T6SSmetV_B171_EPEC	clyA	CS1_E24377A	eaaA	eaeH	eatA	ehaA	ehaB	ehaC	ehaD	eltA_E24377A_ETEC	espI	EspL1_Sakai	EspL4_Sakai	EspR1_Sakai	EspR3_Sakai	EspR4_Sakai	EspX1_Sakai	EspX2_Sakai	EspX4_Sakai	EspX5_Sakai	EspX6_Sakai	EspY1_Sakai	EspY2_Sakai	EspY4_Sakai	etpA	fyuA	hcp	irp2	leoA	P0299538_maybe	papG_CFT073_UPEC	sat	ST_ETEC	STP	tia	tibA	tibA_homolog	vat	yghJ
001	-1	1	10	0	0.33	0.72	0.1	0.38	0.97	0.35	0	0.43	0.92	0.3	0.11	0.6	0.83	0.06	0	0.96	0.28	0.04	0	0	0	0	0	0	0	0	0	0	0	0	1	0	1	0.03	0.29	0	0.99	0.19	0.19	0	0.15	0.15	0.37	0.02
001	-1	1	11	0	0.33	0.72	0.1	0.38	0.97	0.35	0	0.43	0.92	0.3	0.11	0.6	0.84	0.06	0	0.96	0.28	0.04	0	0	0	0	0	0	0	0	0	0	0	0	1	0	1	0.03	0.29	0	0.99	0.19	0.19	0	0.15	0.15	0.37	0.02
001	-1	1	12	0	0.33	0.72	0.1	0.38	0.97	0.35	0	0.43	0.92	0.3	0.11	0.6	0.84	0.06	0	0.96	0.28	0.04	0	0	0	0	0	0	0	0	0	0	0	0	1	0	1	0.03	0.29	0	0.99	0.19	0.19	0	0.15	0.15	0.37	0.02
001	-1	1	2	0	0.33	0.72	0.1	0.38	0.97	0.35	0	0.43	0.92	0.3	0.11	0.6	0.83	0.06	0	0.96	0.28	0.04	0	0	0	0	0	0	0	0	0	0	0	0	1	0	1	0.03	0.29	0	0.99	0.19	0.19	0	0.15	0.15	0.37	0.02
001	-1	1	3	0	0.33	0.72	0.1	0.38	0.97	0.35	0	0.43	0.92	0.3	0.11	0.6	0.84	0.06	0	0.96	0.28	0.04	0	0	0	0	0	0	0	0	0	0	0	0	1	0	1	0.03	0.29	0	0.99	0.19	0.19	0	0.15	0.15	0.37	0.02
001	-1	1	4	0	0.33	0.72	0.1	0.38	0.97	0.35	0	0.43	0.92	0.3	0.11	0.6	0.84	0.06	0	0.96	0.28	0.04	0	0	0	0	0	0	0	0	0	0	0	0	1	0	1	0.03	0.29	0	0.99	0.19	0.19	0	0.15	0.15	0.37	0.02
001	-1	1	5	0	0.33	0.72	0.1	0.38	0.97	0.35	0	0.43	0.92	0.3	0.11	0.6	0.83	0.06	0	0.96	0.28	0.04	0	0	0	0	0	0	0	0	0	0	0	0	1	0	1	0.03	0.29	0	0.99	0.19	0.19	0	0.15	0.15	0.37	0.02
001	-1	1	6	0	0.33	0.72	0.1	0.38	0.97	0.35	0	0.43	0.92	0.3	0.1	0.6	0.62	0.06	0	0.96	0.28	0.04	0	0	0	0	0	0	0	0	0	0	0	0	1	0	1	0.03	0.29	0	0.99	0.19	0.19	0	0.15	0.15	0.37	0.02
001	-1	1	7	0	0.33	0.72	0.1	0.38	0.97	0.35	0	0.43	0.92	0.3	0.11	0.6	0.83	0.06	0	0.96	0.28	0.04	0	0	0	0	0	0	0	0	0	0	0	0	1	0	1	0.03	0.29	0	0.99	0.19	0.19	0	0.15	0.15	0.37	0.02
001	-1	1	8	0	0.33	0.72	0.1	0.38	0.97	0.35	0	0.43	0.92	0.3	0.11	0.6	0.84	0.06	0	0.96	0.28	0.04	0	0	0	0	0	0	0	0	0	0	0	0	1	0	1	0.03	0.29	0	0.99	0.19	0.19	0	0.15	0.15	0.37	0.02
001	0	1	11	0	0.33	0.72	0.1	0.38	0.97	0.35	0	0.43	0.92	0.3	0.11	0.6	0.84	0.06	0	0.96	0.28	0.04	0	0	0	0	0	0	0	0	0	0	0	0	1	0	1	0.03	0.29	0	0.99	0.19	0.19	0	0.15	0.15	0.37	0.02
001	0	1	12	0	0.33	0.72	0.1	0.38	0.97	0.35	0	0.43	0.92	0.3	0.11	0.6	0.84	0.06	0	0.96	0.28	0.04	0	0	0	0	0	0	0	0	0	0	0	0	1	0	1	0.03	0.29	0	0.99	0.19	0.19	0	0.15	0.15	0.37	0.02
001	0	1	1	0	0.33	0.72	0.1	0.38	0.97	0.35	0	0.43	0.92	0.3	0.11	0.6	0.84	0.06	0	0.96	0.28	0.04	0	0	0	0	0	0	0	0	0	0	0	0	1	0	1	0.03	0.29	0	0.99	0.19	0.19	0	0.15	0.15	0.37	0.02
001	0	1	2	0	0.33	0.72	0.1	0.38	0.97	0.35	0	0.43	0.92	0.3	0.11	0.6	0.84	0.06	0	0.96	0.28	0.04	0	0	0	0	0	0	0	0	0	0	0	0	1	0	1	0.03	0.29	0	0.99	0.19	0.19	0	0.15	0.15	0.37	0.02
001	0	1	3	0	0.33	0.72	0.1	0.38	0.97	0.35	0	0.43	0.92	0.3	0.11	0.6	0.84	0.06	0	0.96	0.28	0.04	0	0	0	0	0	0	0	0	0	0	0	0	1	0	1	0.03	0.29	0	0.99	0.19	0.19	0	0.15	0.15	0.37	0.02
001	0	1	4	0	0.33	0.72	0.1	0.38	0.97	0.35	0	0.43	0.92	0.3	0.11	0.6	0.84	0.06	0	0.96	0.28	0.04	0	0	0	0	0	0	0	0	0	0	0	0	1	0	1	0.03	0.29	0	0.99	0.19	0.19	0	0.15	0.15	0.37	0.02
001	0	1	5	0	0.33	0.72	0.1	0.38	0.97	0.35	0	0.43	0.92	0.3	0.11	0.6	0.83	0.06	0	0.96	0.28	0.04	0	0	0	0	0	0	0	0	0	0	0	0	1	0	1	0.03	0.29	0	0.99	0.19	0.19	0	0.15	0.15	0.37	0.02
001	0	1	6	0	0.33	0.72	0.1	0.38	0.97	0.35	0	0.43	0.92	0.3	0.11	0.6	0.83	0.06	0	0.96	0.28	0.04	0	0	0	0	0	0	0	0	0	0	0	0	1	0	1	0.03	0.29	0	0.99	0.19	0.19	0	0.15	0.15	0.37	0.02
001	0	1	8	0	0.33	0.72	0.1	0.38	0.97	0.35	0	0.43	0.92	0.3	0.11	0.6	0.83	0.06	0	0.96	0.28	0.04	0	0	0	0	0	0	0	0	0	0	0	0	1	0	1	0.03	0.29	0	0.99	0.19	0.19	0	0.15	0.15	0.37	0.02
001	0	1	9	0	0.33	0.72	0.1	0.38	0.97	0.35	0	0.43	0.92	0.3	0.11	0.6	0.84	0.06	0	0.96	0.28	0.04	0	0	0	0	0	0	0	0	0	0	0	0	1	0	1	0.03	0.29	0	0.99	0.19	0.19	0	0.15	0.15	0.37	0.02
001	1	1	10	0	0.33	0.72	0.1	0.38	0.97	0.35	0	0.43	0.92	0.3	0.11	0.6	0.84	0.06	0	0.21	0.28	0.04	0	0	0	0	0	0	0	0	0	0	0	0	1	0	1	0.03	0.29	0	0.99	0.19	0.19	0	0.15	0.15	0.37	0.02
001	1	1	11	0	0.98	0.72	0.1	0.38	0.97	0.35	0	0.43	0.92	0.3	0.11	0.6	0.84	0.06	0	0.96	0.28	0.04	0	0	0	0	0	0	0	0	0	0	0	0.82	1	0	1	0.03	0.29	0	0.99	0.19	0.19	0	0.15	0.15	0.37	0.02
001	1	1	12	0	0.33	0.72	0.1	0.38	0.97	0.35	0	0.43	0.92	0.3	0.11	0.6	0.84	0.06	0	0.96	0.28	0.04	0	0	0	0	0	0	0	0	0	0	0	0	1	0	1	0.03	0.29	0	0.99	0.19	0.19	0	0.15	0.15	0.37	0.02
001	1	1	1	0	0.33	0.72	0.1	0.38	0.97	0.35	0	0.43	0.92	0.3	0.11	0.6	0.84	0.06	0	0.96	0.28	0.04	0	0	0	0	0	0	0	0	0	0	0	0	1	0	1	0	0.29	0	0.99	0.19	0.19	0	0.15	0.15	0.37	0.02
001	1	1	2	0	0.33	0.72	0.1	0.38	0.97	0.35	0	0.43	0.92	0.3	0.11	0.6	0.84	0.06	0	0.96	0.28	0.04	0	0	0	0	0	0	0	0	0	0	0	0.8	1	0	1	0.03	0.29	0	0.99	0.19	0.19	0	0.15	0.15	0.37	0.02
001	1	1	3	0	0.33	0.72	0.1	0.38	0.97	0.35	0	0.43	0.92	0.3	0.11	0.6	0.84	0.06	0	0.96	0.28	0.04	0	0	0	0	0	0	0	0	0	0	0	0	1	0	1	0.03	0.29	0	0.99	0.19	0.19	0	0.15	0.15	0.37	0.02
001	1	1	4	0	0.33	0.72	0.1	0.38	0.97	0.35	0	0.43	0.92	0.3	0.11	0.6	0.83	0.06	0	0.96	0.28	0.04	0	0	0	0	0	0	0	0	0	0	0	0	1	0	1	0.03	0.29	0	0.99	0.19	0.19	0	0.15	0.15	0.37	0.02
001	1	1	5	0	0.33	0.72	0.1	0.38	0.97	0.35	0	0.43	0.92	0.3	0.11	0.6	0.84	0.06	0	0.96	0.28	0.04	0	0	0	0	0	0	0	0	0	0	0	0	1	0	1	0.03	0.29	0	0.99	0.19	0.19	0	0.15	0.15	0.37	0.02
001	1	1	7	0	0.33	0.72	0.1	0.38	0.97	0.35	0	0.43	0.92	0.3	0.11	0.6	0.84	0.06	0	0.96	0.28	0.04	0	0	0	0	0	0	0	0	0	0	0	0	1	0	1	0.03	0.29	0	0.99	0.19	0.19	0	0.15	0.15	0.37	0.02
001	1	1	9	0	0.33	0.72	0.1	0.38	0.97	0.35	0	0.43	0.92	0.3	0.11	0.6	0.84	0.06	0	0.96	0.28	0.04	0	0	0	0	0	0	0	0	0	0	0	0	1	0	1	0.03	0.29	0	0.99	0.19	0.19	0	0.15	0.15	0.37	0.02
001	1	2	11	0	0.33	0.72	0.1	0.38	0.97	0.35	0	0.43	0.92	0.3	0.11	0.6	0.84	0.06	0	0.96	0.28	0.04	0	0	0	0	0	0	0	0	0	0	0	0	1	0	1	0.03	0.29	0	0.99	0.19	0.19	0	0.15	0.15	0.37	0.02
001	1	2	12	0	0.33	0.72	0.1	0.38	0.97	0.35	0	0.43	0.92	0.3	0.11	0.6	0.84	0.06	0	0.96	0.28	0.04	0	0	0	0	0	0	0	0	0	0	0	0	1	0	1	0.03	0.29	0	0.99	0.19	0.19	0	0.15	0.15	0.37	0.02
001	1	2	1E	0	0.33	0.72	0.1	0.38	0.97	0.35	0	0.43	0.92	0.3	0.11	0.6	0.83	0.06	0	0.96	0.28	0.04	0	0	0	0	0	0	0	0	0	0	0	0	1	0	1	0.03	0.29	0	0.99	0.19	0.19	0	0.15	0.15	0.37	0.02
001	1	2	1	0	0.98	0.58	0	0.27	0.32	1	0	0.04	1	0.04	0.27	0.59	0.96	0.96	0	0.21	0.74	0.96	0.47	0	0	0.74	0	0.94	0.95	0.05	0.82	0.24	0	0.59	1	0	1	1	0.2	0	0.04	0.65	1	1	1	1	0.04	1
001	1	2	2E	0	0.33	0.72	0.1	0.38	0.97	0.35	0	0.43	0.92	0.3	0.11	0.6	0.84	0.06	0	0.96	0.28	0.04	0	0	0	0	0	0	0	0	0	0	0	0	1	0	1	0.03	0.29	0	0.99	0.19	0.19	0	0.15	0.15	0.37	0.02
001	1	2	2	0	0.98	0.72	0.1	0.38	0.97	1	0	0.43	1	0.3	0.22	0.6	0.93	0.96	0	0.96	0.74	0.96	0.47	0	0	0.74	0	0.94	0.95	0.05	0.82	0.24	0	0	1	0	1	1	0.81	0	0.99	0.19	0	1	1	1	0.37	1
001	1	2	3E	0	0.33	0.72	0.1	0.38	0.97	0.35	0	0.43	0.92	0.3	0.11	0.6	0.83	0.06	0	0.96	0.28	0.04	0	0	0	0	0	0	0	0	0	0	0	0	1	0	1	0.03	0.29	0	0.99	0.19	0.19	0	0.15	0.15	0.37	0.02
001	1	2	3	0	0.33	0.72	0.1	0.38	0.97	0.35	0	0.43	0.92	0.3	0.11	0.6	0.84	0.06	0	0.96	0.28	0.04	0	0	0	0	0	0	0	0	0	0	0	0	1	0	1	0.03	0.29	0	0.99	0.19	0.19	0	0.15	0.15	0.37	0.02
001	1	2	4E	0	0.33	0.72	0.1	0.38	0.97	0.35	0	0.43	0.92	0.3	0.11	0.6	0.84	0.06	0	0.96	0.28	0.04	0	0	0	0	0	0	0	0	0	0	0	0	1	0	1	0.03	0.29	0	0.67	0.19	0.19	0	0.15	0.15	0.37	0.02
001	1	2	4	0	0.98	0.78	0	0.27	0.32	1	0	0.04	1	0.04	0.22	0.59	0.96	0.96	0	0.21	0.74	0.96	0.47	0	0	0.74	0	0.94	0.95	0.05	0.82	0.24	0	0	1	0	1	1	0.81	0	0.04	0.91	0.68	1	1	1	0.04	1
001	1	2	5E	0	0.33	0.72	0.1	0.38	0.97	0.35	0	0.43	0.92	0.3	0.11	0.6	0.84	0.06	0	0.96	0.28	0.04	0	0	0	0	0	0	0	0	0	0	0	0	1	0	1	0.03	0.29	0	0.99	0.19	0.19	0	0.15	0.15	0.37	0.02
001	1	2	5	0.97	0.98	0.72	0.99	0.38	0.97	1	0	0.43	0.92	1	0.22	0.6	0.85	0.96	1	0.96	0.74	0.96	0.47	0	0	0.74	0	0.94	0.95	0.05	0.82	0.24	0	1	1	0	1	0.75	0.81	0	0.99	0.91	1	1	0.38	0.38	0.39	0.93
001	1	2	6E	0	0.33	0.72	0.1	0.38	0.97	0.35	0	0.43	0.92	0.3	0.11	0.6	0.84	0.06	0	0.96	0.28	0.04	0	0	0	0	0	0	0	0	0	0	0	0	1	0	1	0.03	0.29	0	0.99	0.19	0.19	0	0.15	0.15	0.37	0.02
001	1	2	6	0.53	0.98	0.78	0.99	0.27	0.32	1	0	0.04	1	0.04	0.22	0.59	0.96	0.96	1	0.21	0.74	0.96	0.47	0	0	0.74	0	0.94	0.95	0.05	0.82	0.24	0	0.41	1	0	1	1	0.81	0	0.04	0.91	0.68	1	1	1	0.04	1
001	1	2	8	0	0.33	0.72	0.1	0.38	0.97	0.35	0	0.43	0.92	0.3	0.11	0.6	0.84	0.06	0	0.96	0.28	0.04	0	0	0	0	0	0	0	0	0	0	0	0	1	0	1	0.03	0.29	0	0.99	0.19	0.19	0	0.15	0.15	0.37	0.02
001	1	2	9	0	0	0.72	0.1	0.38	0.97	0.35	0	0.43	0.92	0.3	0.11	0.6	0.84	0.06	0	0.96	0.28	0.04	0	0	0	0	0	0	0	0	0	0	0	0	1	0	1	0.03	0.29	0	0.99	0.19	0	0	0.15	0.15	0.37	0.02
001	2	1	10	0	0.33	0.72	0.1	0.38	0.97	0.35	0	0.43	0.92	0.3	0.11	0.6	0.84	0.06	0	0.96	0.28	0.04	0	0	0	0	0	0	0	0	0	0	0	0	1	0	1	0.03	0.29	0	0.99	0.19	0.19	0	0.15	0.15	0.37	0.02
001	2	1	1	0	0.33	0.72	0.1	0.38	0.97	0.35	0	0.43	0.92	0.3	0.11	0.6	0.84	0.06	0	0.96	0.28	0.04	0	0	0	0	0	0	0	0	0	0	0	0	1	0	1	0.03	0.29	0	0.99	0.19	0.19	0	0.15	0.15	0.37	0.02
001	2	1	2	0	0.33	0.72	0.1	0.38	0.97	0.35	0	0.43	0.92	0.3	0.11	0.6	0.84	0.06	0	0.96	0.28	0.04	0	0	0	0	0	0	0	0	0	0	0	0	1	0	1	0.03	0.29	0	0.99	0.19	0.19	0	0.15	0.15	0.37	0.02
001	2	1	3	0	0.33	0.72	0.1	0.38	0.97	0.35	0	0.43	0.92	0.3	0.06	0.6	0.11	0.06	0	0.96	0.28	0.04	0	0	0	0	0	0	0	0	0	0	0	0	1	0	1	0.03	0.29	0	0.99	0	0.19	0	0.15	0.15	0.37	0.02
001	2	1	4	0	0.33	0.72	0.1	0.38	0.97	0.35	0	0.43	0.92	0.3	0.11	0.6	0.84	0.06	0	0.96	0.28	0.04	0	0	0	0	0	0	0	0	0	0	0	0	1	0	1	0	0.29	0	0.99	0.19	0.19	0	0.15	0.15	0.37	0.02
001	2	1	5	0	0.33	0.72	0.1	0.38	0.97	0.35	0	0.43	0.92	0.3	0.11	0.6	0.83	0.06	0	0.96	0.28	0.04	0	0	0	0	0	0	0	0	0	0	0	0	1	0	1	0.03	0.29	0	0.99	0.19	0.19	0	0.15	0.15	0.37	0.02
001	2	1	6	0	0.33	0.72	0.1	0.38	0.97	0.35	0	0.43	0.92	0.3	0.11	0.6	0.83	0.06	0	0.96	0.28	0.04	0	0	0	0	0	0	0	0	0	0	0	0	1	0	1	0.03	0.29	0	0.99	0.19	0.19	0	0.15	0.15	0.37	0.02
001	2	1	7	0	0.33	0.72	0.1	0.38	0.97	0.35	0	0.43	0.92	0.3	0.11	0.6	0.83	0.06	0	0.96	0.28	0.04	0	0	0	0	0	0	0	0	0	0	0	0	1	0	1	0.03	0.29	0	0.99	0.19	0.19	0	0.15	0.15	0.37	0.02
001	2	1	8	0	0.33	0.72	0.1	0.38	0.97	0.35	0	0.43	0.92	0.3	0.11	0.6	0.84	0.06	0	0.96	0.28	0.04	0	0	0	0	0	0	0	0	0	0	0	0	1	0	1	0.03	0.29	0	0.99	0.19	0.19	0	0.15	0.15	0.37	0.02
001	2	1	9	0	0.33	0.72	0	0.38	0.97	0.35	0	0.43	0.92	0.3	0.11	0.6	0.84	0.06	0	0.96	0.28	0.04	0	0	0	0	0	0	0	0	0	0	0	0	1	0	1	0.03	0.29	0	0.99	0.19	0.19	0	0.15	0.15	0.37	0.02
001	2	2	10	0	0.33	0.72	0.1	0.38	0.97	0.35	0	0.43	0.92	0.3	0.11	0.6	0.84	0.06	0	0.96	0.28	0.04	0	0	0	0	0	0	0	0	0	0	0	0	1	0	1	0.03	0.29	0	0.99	0.19	0.19	0	0.15	0.15	0.37	0.02

001	2	2 11	0	0.33	0.72	0.1	0.38	0.97	0.35	0	0.33	0.92	0.29	0.11	0.6	0.84	0.06	0	0.96	0.28	0.04	0	0	0	0	0	0	0	0	0	0	0	0	1	0	1	0.03	0.29	0	0.99	0.19	0.19	0	0.15	0.15	0.29	0.02
001	2	2 12	0	0.33	0.72	0.1	0.38	0.97	0.35	0	0.43	0.92	0.3	0.11	0.6	0.84	0.06	0	0.96	0.28	0.04	0	0	0	0	0	0	0	0	0	0	0	0	1	0	1	0.03	0.29	0	0.99	0.19	0.19	0	0.15	0.15	0.37	0.02
001	2	2 1	0	0.33	0.72	0.1	0.38	0.97	0.35	0	0.43	0.92	0.3	0.11	0.6	0.84	0.06	0	0.96	0.28	0.04	0	0	0	0	0	0	0	0	0	0	0	0	1	0	1	0.03	0.29	0	0.99	0.19	0.19	0	0.15	0.15	0.37	0.02
001	2	2 2	0	0.33	0.72	0.1	0.38	0.97	0.35	0	0.43	0.92	0.3	0.11	0.6	0.84	0.06	0	0.96	0.28	0.04	0	0	0	0	0	0	0	0	0	0	0	0	1	0	1	0.03	0.29	0	0.99	0.19	0.19	0	0.15	0.15	0.37	0.02
001	2	2 3	0	0.33	0.72	0.1	0.38	0.97	0.35	0	0.43	0.92	0.3	0.11	0.6	0.84	0.06	0	0.96	0.28	0.04	0	0	0	0	0	0	0	0	0	0	0	0	1	0	1	0.03	0.29	0	0.99	0.19	0.19	0	0.15	0.15	0.37	0.02
001	2	2 6	0	0.33	0.72	0.1	0.38	0.97	0.35	0	0.43	0.92	0.3	0.11	0.6	0.83	0.06	0	0.96	0.28	0.04	0	0	0	0	0	0	0	0	0	0	0	0	1	0	1	0.03	0.29	0	0.99	0.19	0.19	0	0.15	0.15	0.37	0.02
001	2	2 7	0	0.33	0.72	0.1	0.38	0.97	0.35	0	0.43	0.92	0.3	0.11	0.6	0.84	0.06	0	0.96	0.28	0.04	0	0	0	0	0	0	0	0	0	0	0	0	1	0	1	0.03	0.29	0	0.99	0.19	0.19	0	0.15	0.15	0.37	0.02
001	2	2 8	0	0.33	0.72	0.1	0.38	0.97	0.35	0	0.43	0.92	0.3	0.11	0.6	0.84	0.06	0	0.96	0.28	0.04	0	0	0	0	0	0	0	0	0	0	0	0.65	1	0	1	0.03	0.29	0	0.99	0.19	0.19	0	0.15	0.15	0.37	0.02
001	2	2 9	0	0.33	0.72	0.1	0.38	0.97	0.35	0	0.43	0.92	0.3	0.11	0.6	0.84	0.06	0	0.96	0.28	0.04	0	0	0	0	0	0	0	0	0	0	0	0	1	0	1	0.03	0.29	0	0.99	0.19	0.19	0	0.15	0.15	0.37	0.02
001	3	1 10	0	0.33	0.72	0.1	0.38	0.97	0.35	0	0.43	0.92	0.3	0.11	0.6	0.84	0.06	0	0.96	0.28	0.04	0	0	0	0	0	0	0	0	0	0	0	0	1	0	1	0.03	0.29	0	0.99	0.19	0.19	0	0.15	0.15	0.37	0.02
001	3	1 1	0	0.33	0.72	0.1	0.38	0.97	0.35	0	0.43	0.92	0.3	0.11	0.6	0.84	0.06	0	0.96	0.28	0.04	0	0	0	0	0	0	0	0	0	0	0	0	1	0	1	0.03	0.29	0	0.99	0.19	0.19	0	0.15	0.15	0.37	0.02
001	3	1 2	0	0.33	0.72	0.1	0.38	0.97	0.35	0	0.43	0.92	0.3	0.11	0.6	0.84	0.06	0	0.96	0.28	0.04	0	0	0	0	0	0	0	0	0	0	0	0	1	0	1	0.03	0.29	0	0.99	0.19	0.19	0	0.15	0.15	0.37	0.02
001	3	1 3	0	0.33	0.72	0.1	0.38	0.97	0.35	0	0.43	0.92	0.3	0.11	0.6	0.84	0.06	0	0.96	0.28	0.04	0	0	0	0	0	0	0	0	0	0	0	0	1	0	1	0.03	0.29	0	0.99	0.19	0.19	0	0.15	0.15	0.37	0.02
001	3	1 4	0	0.33	0.72	0.1	0.38	0.97	0.35	0	0.43	0.92	0.3	0.11	0.6	0.84	0.06	0	0.96	0.28	0.04	0	0	0	0	0	0	0	0	0	0	0	0	1	0	1	0.03	0.29	0	0.99	0.19	0.19	0	0.15	0.15	0.37	0.02
001	3	1 5	0	0.33	0.72	0.1	0.38	0.97	0.35	0	0.43	0.92	0.3	0.11	0.6	0.84	0.06	0	0.96	0.28	0.04	0	0	0	0	0	0	0	0	0	0	0	0	1	0	1	0.03	0.29	0	0.99	0.19	0.19	0	0.15	0.15	0.37	0.02
001	3	1 6	0	0.33	0.72	0.1	0.38	0.97	0.35	0	0.43	0.92	0.3	0.11	0.6	0.84	0.06	0	0.96	0.28	0.04	0	0	0	0	0	0	0	0	0	0	0	0	1	0	1	0.03	0.29	0	0.99	0.19	0.19	0	0.15	0.15	0.37	0.02
001	3	1 7	0	0.33	0.72	0.1	0.38	0.97	0.35	0	0.43	0.92	0.3	0.11	0.6	0.84	0.06	0	0.96	0.28	0.04	0	0	0	0	0	0	0	0	0	0	0	0	1	0	1	0.03	0.29	0	0.99	0.19	0.19	0	0.15	0.15	0.37	0.02
001	3	1 8	0	0.33	0.72	0.1	0.38	0.97	0.35	0	0.43	0.92	0.3	0.11	0.6	0.84	0.06	0	0.96	0.27	0.04	0	0	0	0	0	0	0	0	0	0	0	0	1	0	1	0.03	0.29	0	0.99	0.19	0.19	0	0.15	0.15	0.37	0.02
001	3	1 9	0.37	0.98	0.72	0.1	0.38	0.97	0.35	0	0.43	0.92	0.3	0.11	0.6	0.84	0.06	0.72	0.96	0.28	0.04	0	0	0	0	0	0	0	0	0	0	0	0.72	1	0	1	0.03	0.29	0	0.99	0.19	0.19	0	0.15	0.15	0.37	0.02
001	3	2 12	0.94	0.98	0.72	0.99	0.38	0.97	0.35	0	0.43	0.92	0.56	0.11	0.6	0.84	0.07	1	0.96	0.28	0.21	0.34	0	0	0.19	0	0.55	0.04	0.02	0	0	0	0.82	1	0	1	0.26	0.81	0	0.99	0.73	1	0	0.15	0.15	0.37	0.11
001	3	2 1	0	0.33	0.72	0.1	0.38	0.97	0.35	0	0.43	0.92	0.3	0.11	0.6	0.84	0.06	0	0.96	0.28	0.04	0	0	0	0	0	0	0	0	0	0	0	0	1	0	1	0.03	0.29	0	0.99	0.19	0.19	0	0.15	0.15	0.37	0.02
001	3	2 2	0	0.33	0.72	0.1	0.38	0.97	0.35	0	0.43	0.92	0.3	0.11	0.6	0.84	0.06	0	0.96	0.28	0.04	0	0	0	0	0	0	0	0	0	0	0	0	1	0	1	0.03	0.29	0	0.99	0.19	0.19	0	0.15	0.15	0.37	0.02
001	3	2 3	0	0.33	0.72	0.1	0.38	0.97	0.35	0	0.43	0.92	0.3	0.11	0.6	0.83	0.06	0	0.96	0.28	0.04	0	0	0	0	0	0	0	0	0	0	0	0	1	0	1	0.03	0.29	0	0.99	0.19	0.19	0	0.15	0.15	0.37	0.02
001	3	2 4	0	0.33	0.72	0.1	0.38	0.97	0.35	0	0.43	0.92	0.3	0.11	0.6	0.84	0.06	0	0.96	0.28	0.04	0	0	0	0	0	0	0	0	0	0	0	0	1	0	1	0.03	0.29	0	0.99	0.19	0.19	0	0.15	0.15	0.37	0.02
001	3	2 5	0	0.33	0.72	0.1	0.38	0.97	0.35	0	0.43	0.92	0.3	0.11	0.6	0.84	0.06	0	0.96	0.28	0.04	0	0	0	0	0	0	0	0	0	0	0	0	1	0	1	0.03	0.29	0	0.99	0.19	0.19	0	0.15	0.15	0.37	0.02
001	3	2 6	0	0.33	0.72	0.1	0.38	0.97	0.35	0	0.43	0.92	0.3	0.11	0.6	0.83	0.06	0	0.96	0.28	0.04	0	0	0	0	0	0	0	0	0	0	0	0	1	0	1	0.03	0.29	0	0.99	0.19	0.19	0	0.15	0.15	0.37	0.02
001	3	2 7	0	0	0.72	0.1	0.38	0.97	0.35	0	0.43	0.92	0.3	0.11	0.6	0.84	0.06	0	0.96	0.28	0.04	0	0	0	0	0	0	0	0	0	0	0	0	1	0	1	0.03	0.29	0	0.99	0.19	0	0	0.15	0.15	0.37	0.02
001	3	2 8	0	0.33	0.72	0.1	0.38	0.97	0.35	0	0.43	0.92	0.3	0.11	0.6	0.84	0.06	0	0.96	0.28	0.04	0	0	0	0	0	0	0	0	0	0	0	0.7	1	0	1	0.03	0.29	0	0.99	0.19	0.19	0	0.15	0.15	0.37	0.02
001	3	2 9	0	0.78	0.72	0.1	0.38	0.97	0.35	0	0.43	0.92	0.3	0.11	0.6	0.84	0.06	0	0.96	0.28	0.04	0	0	0	0	0	0	0	0	0	0	0	0.82	1	0	1	0.03	0.29	0	0.99	0.3	0.47	0	0.15	0.15	0.37	0.02
001	4	1 10	0	0.33	0.72	0.1	0.38	0.97	0.35	0	0.43	0.92	0.3	0.11	0.6	0.83	0.06	0	0.96	0.28	0.04	0	0	0	0	0	0	0	0	0	0	0	0	1	0	1	0.03	0.29	0	0.99	0.19	0.19	0	0.15	0.15	0.37	0.02
001	4	1 1E	0	0.98	0.61	0.1	0.38	0.97	0.35	0	0.43	0.92	0.3	0.11	0.6	0.84	0.06	0	0.96	0.28	0.04	0	0	0	0	0	0	0	0	0	0	0	0.82	1	0	1	0.03	0.47	0	0.99	0.65	1	0	0.15	0.15	0.37	0.02
001	4	1 1	0.97	0.98	0.72	0.99	0.38	0.97	0.35	0	0.43	0.92	0.78	0.11	0.6	0.84	0.06	1	0.96	0.28	0.14	0	0	0	0	0	0	0	0	0	0	0	0.82	1	0	1	0.03	0.81	0	0.99	0.91	1	0	0.15	0.15	0.37	0.02
001	4	1 2E	0	0.98	0.6	0.99	0.38	0.97	1	0	0.43	0.72	0.3	0.22	0.58	0.96	0.96	0	0.96	0.74	0.96	0.47	0	0	0.74	0	0.94	0.95	0.05	0.82	0.24	0	0	1	0	1	1	0.47	0	0.99	0.19	0	1	1	1	0.37	1
001	4	1 2	0.97	0.98	0.8	0.99	0.38	0.97	1	0	0.43	0.73	1	0.22	0.59	0.87	0.96	1	0.96	0.74	0.96	0.47	0	0	0.74	0	0.94	0.95	0.05	0.82	0.24	0	1	1	0	1	1	0.81	0	0.99	0.91	1	1	1	1	0.39	1
001	4	1 3E	0.97	0.98	0.85	0.99	0.38	0.97	1	0	0.04	0.92	0.04	0.22	0.6	0.95	0.96	0	0.96	0.74	0.96	0.47	0	0	0.74	0	0.94	0.95	0.05	0.82	0.24	0	1	1	0	0.19	0.03	0.47	0	0.04	0.91	0.68	0	1	1	0.04	1
001	4	1 3	0	0.33	0.72	0.1	0.38	0.97	0.35	0	0.43	0.92	0.3	0.11	0.6	0.83	0.06	0	0.96	0.28	0.04	0	0	0	0	0	0	0	0	0	0	0	0	1	0	1	0.03	0.29	0	0.99	0.19	0.19	0	0.15	0.15	0.37	0.02
001	4	1 4E	0.97	0.98	0.72	0.99	0.38	0.97	0.35	0	0.43	0.92	0.61	0.1	0.6	0.69	0.06	0.61	0.96	0.28	0.1	0	0	0	0	0	0.18	0	0	0	0	0	0.82	1	0	1	0.03	0.81	0	0.99	0.91	1	0	0.15	0.15	0.37	0.02
001	4	1 4	0.97	0.98	0.72	0.99	0.38	0.97	1	0	0.31	0.95	1	0.22	0.59	0.96	0.96	1	0.21	0.74	0.96	0.47	0	0	0.74	0	0.94	0.95	0.05	0.82	0.24	0	1	1	0	1	1	0.81	0	0.99	0.91	1	1	0.63	0.63	0.39	1
001	4	1 5E	0.97	0.98	0.72	0.99	0.38	0.97	0.49	0	0.43	0.65	1	0.11	0.6	0.84	0.15	1	0.96	0.23	0.41	0.34	0	0	0.74	0	0.76	0.74	0.05	0.65	0.23	0	1	1	0	1	0.31	0.47	0	0.99	0.91	1	0.8	0.15	0.15	0.39	0.12
001	4	1 5	0.97	0.98	0.72	0.99	0.38	0.97	1	0	0.43	0.88	1	0.22	0.6	0.96	0.96	1	0.21	0.74	0.96	0.47	0	0	0.74	0	0.94	0.95	0.05	0.82	0.24	0	1	1	0	1	1	0.81	0	0.99	0.91	1	1	0.98	0.98	0.39	0.54
001	4	1 6E	0.97	0.98	0.31	0.99	0.38	0.97	1	0	0.31	1	1	0.22	0.6	0.74	0.96	1	0.96	0.74	0.96	0.47	0	0	0.74	0	0.94	0.95	0.05	0.82	0.24	0	1	1	0	1	1	0.81	0	0.99	0.91	0.68	1	1	1	0.39	1
001	4	1 6	0	0.33	0.72	0.1	0.38	0.97	0.35	0	0.43	0.92	0.3	0.11	0.6	0.84	0.06	0	0.96	0.28	0.04	0	0	0	0	0	0	0	0	0	0	0	0	1	0	1	0.03	0.29	0	0.99	0.19	0.19	0	0.15	0.15	0.37	0.02
001	4	1 7	0	0.33	0.72	0.1	0.38	0.97	0.35	0	0.43	0.92	0.3	0.11	0.6	0.83	0.06	0	0.96	0.28	0.04	0	0	0	0	0	0	0	0	0	0	0	0	1	0	1	0.03	0.29	0	0.99	0.19	0.19	0	0.15	0.15	0.37	0.02
001	4	1 8	0	0.98	0.72	0.1	0.38	0.97	0.35	0	0.43	0.92	0.29	0.11	0.6	0.84	0.06	0	0.96	0.28	0.04	0	0	0	0	0	0	0	0	0	0	0	0.82	1	0	1	0.03	0.29	0	0.99	0.41	0.76	0	0.15	0.15	0.37	0.02
001	4	1 9	0.97	0.98	0.72	0.99	0.38	0.97	0.35	0	0.43	0.83	1	0.11	0.6	0.83	0.24	1	0.96	0.26	0.73	0.46	0	0	0.42	0	0.77	0.52	0.03	0.82	0.24	0	1	1	0	1	0.47	0.81	0	0.99	0.91	1	0.72	0.16	0.16	0.39	0.23
001	5	1 10	0	0.33	0.72	0.1	0.38	0.97	0.35	0	0.43	0.22	0.3	0.11	0.6	0.84	0.06	0	0.96	0.28	0.04	0	0	0	0	0	0	0	0	0	0	0	0	1	0	1	0.03	0.29	0	0.99	0.19	0.19	0	0.15	0.15	0.37	0.02
001	5	1 1	0	0.33	0.72	0.1	0.38	0.97	0.35	0	0.3	0.92	0.29	0.11	0.6	0.84	0.06	0	0.96	0.28	0.04	0	0	0	0	0	0	0	0	0	0	0	0	1	0	1	0.03	0.29	0	0.99	0.19	0.19	0	0.15	0.15	0.29	0.02
001	5	1 2	0	0.33	0.72	0.1	0.38	0.97	0.35	0	0.43	0.92	0.3	0.11	0.6	0.83	0.06	0	0.96	0.28	0.04	0	0	0	0	0	0	0	0	0	0	0	0	1	0	1	0.03	0.29	0	0.99	0.19	0.19	0	0.15	0.15	0.37	0.02
001	5	1 3	0	0.98	0.72	0.1	0.38	0.97	0.35	0	0.43	0.92	0.3	0.11	0.6	0.84	0.06	0	0.96	0.28	0.04	0	0	0	0	0	0	0	0	0	0	0	0.41	1	0	1	0.03	0.29	0	0.99	0.19	0.19	0	0.15	0.15	0.37	0.02
001	5	1 4	0	0.33	0.72	0.1	0.38	0.97	0.35	0	0.43	0.92	0.3	0.11	0.6	0.83	0.06	0	0.96	0.28	0.04	0	0	0	0	0	0	0	0	0	0	0	0	1	0	1	0.03	0.29	0	0.99	0.19	0.19	0	0.15	0.15	0.37	0.02
001	5	1 5	0	0	0.72	0.1	0.38	0.97	0.35	0	0.43	0.92	0.3	0.11	0.6	0.84	0.06	0	0.96	0.28	0.04	0	0	0	0	0	0	0	0	0	0	0	0	1	0	1	0.03	0.29	0	0.99	0.19	0	0	0.15	0.15	0.37	0.02
001	5	1 6	0	0.33	0.72	0.1	0.38	0.97	0.35	0	0.43	0.92	0.3	0.11	0.6	0.84	0.06	0	0.96	0.28	0.04	0	0	0	0	0	0	0	0	0	0	0	0	1	0	1	0.03	0.29	0	0.99	0.19	0.19	0	0.15	0.15	0.37	0.02
001	5	1 7	0	0.33	0.72	0.1	0.38	0.97	0.35	0	0.43	0.92	0.3	0.11	0.6	0.83	0.06	0	0.96	0.28	0.04	0	0	0	0	0	0	0	0	0	0	0	0	1	0	1	0.03	0.29	0	0.99	0.39	0.59	0	0.15	0.15	0.37	0.02
001	5	1 8	0	0.33	0.72	0.1	0.38	0.97	0.35	0	0.43	0.92	0.3	0.11	0.6	0.83	0.06	0	0.96	0.28	0.04	0	0	0	0	0	0	0	0	0	0	0	0	1	0	1	0.03	0.29	0	0.99	0.19	0.19	0	0.15	0.15	0.37	0.02
001	5	1 9	0	0.33	0.72	0.1	0.38	0.97	0.35	0	0.43	0.92	0.3	0.11	0.6	0.83	0.06	0	0.96	0.28	0.04	0	0	0	0	0	0	0	0	0	0	0	0	1	0	1	0.03	0.29	0	0.99	0.19	0.19	0	0.15	0.15	0.37	0.02
001	5	2 10	0	0.33	0.72	0.1	0.38	0.97	0.35	0	0.43	0.92	0.3	0.11	0.6	0.83	0.06	0	0.96	0.28	0.04	0	0	0	0	0	0	0	0	0	0	0	0	1	0	1	0.03	0.29	0	0.99	0.19	0.19	0	0.15	0.15	0.37	0.02
001	5	2 11	0	0.33	0.72	0.1	0.38	0.97	0.35	0	0.43	0.92	0.3	0.11	0.6	0.84	0.06	0	0.96	0.28	0.04	0	0	0	0	0	0	0	0	0	0	0	0	1	0	1	0.03	0.29	0	0.99	0.19	0.19	0	0.15	0.15	0.37	0.02
001	5	2 1	0	0.33	0.72	0.1	0.38	0.97	0.35	0	0.43	0.92	0.3	0.11	0.6	0.84	0.06	0	0.96	0.28	0.04	0	0	0	0	0	0	0	0	0	0	0	0	1	0	1	0.03	0.29	0	0.99	0.19	0.19	0	0.15	0.15	0.37	0.02
001	5	2 2	0	0.33	0.72	0.1	0.38	0.97	0.35	0	0.43	0.92	0.3	0.11	0.6	0.84	0.06	0	0.96	0.28	0.04	0	0	0	0	0	0	0	0	0	0	0	0	1	0	1	0.03	0.29	0	0.99	0.19	0.19	0	0.15	0.15	0.37	0.02
001	5	2 3	0	0.33	0.72	0.1	0.34	0.73	0.35	0	0.43	0.92	0.3	0.11	0.6	0.84	0.06	0	0.96	0.28	0.04	0	0	0	0	0	0	0	0	0	0	0	0	1	0	1	0.03	0.29	0	0.99	0.19	0.19	0	0.15	0.15	0.37	0.02
001	5	2 4	0	0.33	0.72	0.1	0.38	0.97	0.35	0	0.43	0.92	0.3	0.11	0.6	0.83	0.06	0	0.96	0.28	0.04	0	0	0	0	0	0	0	0	0	0	0	0	1	0	1	0.03	0.29	0	0.99	0.19	0.19	0	0.15	0.15	0.37	0.02
001	5	2 5	0	0.33	0.72	0.1	0.38	0.97	0.35	0	0.43	0.92	0.3	0.11	0.6	0.83	0.06	0	0.96	0.28	0.04	0	0	0	0	0	0	0	0	0	0	0	0	1	0	1	0.03	0.29	0	0.99	0.19	0.19	0	0.15	0.15	0.37	0.02
001	5	2 6	0	0.33	0.72	0.1	0.38	0.97	0.35	0	0.43	0.92	0.3	0.11	0.6	0.83	0.06	0	0.96	0.28	0.04	0	0	0	0	0	0	0	0	0	0	0	0	1	0	1	0.03	0.29	0	0.99	0.19	0.19	0	0.15	0.15	0.37	0.02
001	5	2 8	0	0.33	0.72	0.1	0.38	0.97	0.35	0	0.43	0.92	0.3	0.11	0.6	0.84	0.06	0	0.96	0.28	0.04	0	0	0	0	0	0	0	0	0	0	0	0	1	0	1	0.03	0.29	0	0.99	0.19	0.19	0	0.15	0.15	0.37	0.02
001	5	2 9	0	0.33	0.72	0.1	0.38	0.97	0.35	0	0.43	0.92	0.3	0.11	0.6	0.84	0.06	0	0.96	0.28	0.04	0	0	0	0	0	0	0	0	0	0	0	0	1	0	1	0.03	0.29	0	0.99	0.19	0.19	0	0.15	0.15	0.37	0.02
001	6	1 10	0	0.33	0.72	0.1	0.38	0.97	0.35	0	0.43	0.92	0.3	0.11	0.6	0.83	0.06	0	0.96	0.28	0.04	0	0	0	0	0	0	0	0	0	0	0	0	1	0	1	0.03	0.29	0	0.99	0.19	0.19	0	0.15	0.15	0.37	0.02
001	6	1 1	0	0.33	0.72	0.1	0.38	0.97	0.35	0	0.43	0.92	0.3	0.11	0.6	0.84	0.06	0	0.96	0.28	0.04	0	0	0	0	0	0	0	0	0	0	0	0	1	0	1	0.03	0.29	0	0.99	0.19	0.19	0	0.15	0.15	0.37	0.02
001	6	1 2	0	0.33	0.72	0.1	0.38	0.97	0.35	0	0.43	0.92	0.3	0.11	0.6	0.84	0.06	0	0.96	0.28	0.04	0	0	0	0	0	0	0	0	0	0	0	0	1	0	1	0.03	0.29	0	0.99	0.19	0.19	0	0.15	0.15	0.37	0.02
001	6	1 3	0	0.33	0.72	0.1	0.38	0.97	0.35	0	0.43	0.92	0.3	0.11	0.6	0.83	0.06	0	0.96	0.28	0.04	0	0	0	0	0	0	0	0	0	0	0	0	1	0	1	0.03	0.29	0	0.99	0.19	0.19	0	0.15	0.15	0.37	0.02
001	6	1 4	0	0.33	0.72	0.1	0.38	0.97	0.35	0	0.43	0.92	0.3	0.11	0.6	0.84	0.06	0	0.96	0.28	0.04	0	0	0	0	0	0	0	0	0	0	0	0	1	0	1	0.03	0.29	0	0.99	0.19	0.19	0	0.15	0.15	0.37	0.02

001	6	1	5	0	0.33	0.72	0.1	0.38	0.97	0.35	0	0.43	0.92	0.3	0.11	0.6	0.84	0.06	0	0.96	0.28	0.04	0	0	0	0	0	0	0	0	0	0	0	0	1	0	1	0.03	0.29	0	0.99	0.19	0.19	0	0.15	0.15	0.37	0.02
001	6	1	6	0	0.33	0.72	0.1	0.38	0.97	0.35	0	0.43	0.92	0.3	0.11	0.6	0.83	0.06	0	0.96	0.28	0.04	0	0	0	0	0	0	0	0	0	0	0	0	1	0	1	0.03	0.29	0	0.99	0.19	0.19	0	0.15	0.15	0.37	0.02
001	6	1	7	0	0.33	0.72	0.1	0.38	0.97	0.35	0	0.43	0.92	0.3	0.11	0.6	0.84	0.06	0	0.96	0.28	0.04	0	0	0	0	0	0	0	0	0	0	0	0	1	0	1	0.03	0.29	0	0.99	0.19	0.19	0	0.15	0.15	0.37	0.02
001	6	1	8	0	0.33	0.72	0.1	0.38	0.97	0.35	0	0.43	0.92	0.3	0.11	0.6	0.83	0.06	0	0.96	0.28	0.04	0	0	0	0	0	0	0	0	0	0	0	0	1	0	1	0.03	0.29	0	0.99	0.19	0.19	0	0.15	0.15	0.37	0.02
001	6	1	9	0	0.33	0.72	0.1	0.38	0.97	0.35	0	0.43	0.92	0.3	0.11	0.6	0.83	0.06	0	0.96	0.28	0.04	0	0	0	0	0	0	0	0	0	0	0	0	1	0	1	0.03	0.29	0	0.99	0.19	0.19	0	0.15	0.15	0.37	0.02
001	6	2	10	0	0.33	0.72	0.1	0.38	0.97	0.35	0	0.43	0.92	0.3	0.11	0.6	0.83	0.06	0	0.96	0.28	0.04	0	0	0	0	0	0	0	0	0	0	0	0	1	0	1	0.03	0.29	0	0.99	0.19	0.19	0	0.15	0.15	0.37	0.02
001	6	2	1	0	0.33	0.72	0.1	0.38	0.97	0.35	0	0.43	0.92	0.3	0.11	0.6	0.84	0.06	0	0.96	0.28	0.04	0	0	0	0	0	0	0	0	0	0	0	0	1	0	1	0.03	0.29	0	0.99	0.19	0.19	0	0.15	0.15	0.37	0.02
001	6	2	2	0	0.33	0.72	0.1	0.38	0.97	0.35	0	0.43	0.92	0.3	0.11	0.6	0.84	0.06	0	0.96	0.28	0.04	0	0	0	0	0	0	0	0	0	0	0	0	1	0	1	0.03	0.29	0	0.99	0.19	0.19	0	0.15	0.15	0.37	0.02
001	6	2	3	0	0.33	0.72	0.1	0.38	0.97	0.35	0	0.43	0.92	0.3	0.11	0.6	0.84	0.06	0	0.96	0.28	0.04	0	0	0	0	0	0	0	0	0	0	0	0	1	0	1	0.03	0.29	0	0.99	0.19	0.19	0	0.15	0.15	0.37	0.02
001	6	2	4	0	0	0.72	0.1	0.38	0.97	0.35	0	0.43	0.92	0.3	0.11	0.6	0.84	0.06	0	0.96	0.28	0.04	0	0	0	0	0	0	0	0	0	0	0	0	1	0	1	0.03	0.29	0	0.99	0.19	0	0	0.15	0.15	0.37	0.02
001	6	2	5	0	0.33	0.72	0.1	0.38	0.97	0.35	0	0.43	0.92	0.3	0.11	0.6	0.84	0.06	0	0.96	0.28	0.04	0	0	0	0	0	0	0	0	0	0	0	0	1	0	1	0.03	0.29	0	0.99	0.19	0.19	0	0.15	0.15	0.37	0.02
001	6	2	6	0	0	0.72	0.1	0.38	0.97	0.35	0	0.43	0.92	0.3	0.11	0.6	0.84	0.06	0	0.96	0.28	0.04	0	0	0	0	0	0	0	0	0	0	0	0	1	0	1	0.03	0.29	0	0.99	0.19	0	0	0.15	0.15	0.37	0.02
001	6	2	7	0	0.33	0.72	0.1	0.38	0.97	0.35	0	0.43	0.92	0.3	0.11	0.6	0.84	0.06	0	0.96	0.28	0.04	0	0	0	0	0	0	0	0	0	0	0	0	1	0	1	0.03	0.29	0	0.99	0.19	0.19	0	0.15	0.15	0.37	0.02
001	6	2	9	0	0.33	0.72	0.1	0.38	0.97	0.35	0	0.43	0.92	0.3	0.11	0.6	0.83	0.06	0	0.96	0.28	0.04	0	0	0	0	0	0	0	0	0	0	0	0	1	0	1	0.03	0.29	0	0.99	0.19	0.19	0	0.15	0.15	0.37	0.02
001	7	1	10	0	0.33	0.72	0.1	0.38	0.97	0.35	0	0.43	0.21	0.3	0.11	0.04	0.83	0.06	0	0.96	0.28	0.04	0	0	0	0	0	0	0	0	0	0	0	0	1	0	1	0	0.29	0	0.99	0.19	0.19	0	0.15	0.15	0.37	0.02
001	7	1	11	0	0	0.72	0.1	0.38	0.97	0.35	0	0.43	0.92	0.3	0.11	0.6	0.83	0.06	0	0.96	0.28	0.04	0	0	0	0	0	0	0	0	0	0	0	0	1	0	1	0.03	0.29	0	0.99	0.19	0	0	0.15	0.15	0.37	0.02
001	7	1	1	0	0.33	0.72	0.1	0.38	0.97	0.35	0	0.43	0.92	0.3	0.11	0.6	0.84	0.06	0	0.96	0.28	0.04	0	0	0	0	0	0	0	0	0	0	0	0	1	0	1	0.03	0.29	0	0.99	0.19	0.19	0	0.15	0.15	0.37	0.02
001	7	1	2	0	0.33	0.72	0.1	0.38	0.97	0.35	0	0.43	0.92	0.3	0.11	0.6	0.84	0.06	0	0.96	0.28	0.04	0	0	0	0	0	0	0	0	0	0	0	0	1	0	1	0.03	0.29	0	0.99	0.19	0.19	0	0.15	0.15	0.37	0.02
001	7	1	3	0	0.33	0.72	0.1	0.38	0.97	0.35	0	0.43	0.92	0.3	0.11	0.6	0.84	0.06	0	0.96	0.28	0.04	0	0	0	0	0	0	0	0	0	0	0	0	1	0	1	0.03	0.29	0	0.99	0.19	0.19	0	0.15	0.15	0.37	0.02
001	7	1	4	0	0.33	0.72	0.1	0.38	0.97	0.35	0	0.43	0.92	0.3	0.11	0.6	0.84	0.06	0	0.96	0.28	0.04	0	0	0	0	0	0	0	0	0	0	0	0	1	0	1	0.03	0.29	0	0.99	0.19	0.19	0	0.15	0.15	0.37	0.02
001	7	1	5	0	0.33	0.72	0.1	0.38	0.97	0.35	0	0.43	0.92	0.3	0.11	0.6	0.84	0.06	0	0.96	0.28	0.04	0	0	0	0	0	0	0	0	0	0	0	0	1	0	1	0.03	0.29	0	0.99	0.19	0.19	0	0.15	0.15	0.37	0.02
001	7	1	6	0	0.33	0.72	0.1	0.38	0.97	0.35	0	0.43	0.92	0.3	0.11	0.6	0.84	0.06	0	0.96	0.28	0.04	0	0	0	0	0	0	0	0	0	0	0	0	1	0	1	0.03	0.29	0	0.99	0.19	0.19	0	0.15	0.15	0.37	0.02
001	7	1	7	0	0.33	0.72	0.1	0.38	0.97	0.35	0	0.43	0.92	0.3	0.11	0.6	0.84	0.06	0	0.96	0.28	0.04	0	0	0	0	0	0	0	0	0	0	0	0	1	0	1	0.03	0.29	0	0.99	0.19	0.19	0	0.15	0.15	0.37	0.02
001	7	1	8	0	0.33	0.72	0.1	0.38	0.97	0.35	0	0.43	0.92	0.3	0.11	0.6	0.83	0.06	0	0.96	0.28	0.04	0	0	0	0	0	0	0	0	0	0	0	0	1	0	1	0.03	0.29	0	0.99	0.19	0.19	0	0.15	0.15	0.37	0.02
001	7	2	10	0	0.33	0.72	0.1	0.38	0.97	0.35	0	0.43	0.92	0.3	0.11	0.6	0.84	0.06	0	0.96	0.28	0.04	0	0	0	0	0	0	0	0	0	0	0	0	1	0	1	0.03	0.29	0	0.99	0.19	0.19	0	0.15	0.15	0.37	0.02
001	7	2	1	0	0.33	0.72	0.1	0.38	0.97	0.35	0	0.43	0.92	0.3	0.11	0.6	0.84	0.06	0	0.96	0.28	0.04	0	0	0	0	0	0	0	0	0	0	0	0	1	0	1	0.03	0.29	0	0.99	0.19	0.19	0	0.15	0.15	0.37	0.02
001	7	2	2	0	0.33	0.72	0.1	0.38	0.97	0.35	0	0.43	0.92	0.3	0.11	0.6	0.83	0.06	0	0.96	0.28	0.04	0	0	0	0	0	0	0	0	0	0	0	0	1	0	1	0.03	0.29	0	0.99	0.19	0.19	0	0.15	0.15	0.37	0.02
001	7	2	3	0	0.33	0.72	0.1	0.38	0.97	0.35	0	0.43	0.92	0.3	0.11	0.6	0.84	0.06	0	0.96	0.28	0.04	0	0	0	0	0	0	0	0	0	0	0	0	1	0	1	0.03	0.29	0	0.99	0.19	0.19	0	0.15	0.15	0.37	0.02
001	7	2	4	0	0.33	0.72	0.1	0.38	0.97	0.35	0	0.43	0.92	0.3	0.11	0.6	0.84	0.06	0	0.96	0.28	0.04	0	0	0	0	0	0	0	0	0	0	0	0	1	0	1	0.03	0.29	0	0.99	0.19	0.19	0	0.15	0.15	0.37	0.02
001	7	2	5	0	0.33	0.72	0.1	0.38	0.97	0.35	0	0.43	0.92	0.3	0.11	0.6	0.84	0.06	0	0.96	0.28	0.04	0	0	0	0	0	0	0	0	0	0	0	0	1	0	1	0.03	0.29	0	0.99	0.19	0.19	0	0.15	0.15	0.37	0.02
001	7	2	6	0	0.33	0.72	0.1	0.38	0.97	0.35	0	0.43	0.92	0.3	0.11	0.6	0.84	0.06	0	0.96	0.28	0.04	0	0	0	0	0	0	0	0	0	0	0	0	1	0	1	0.03	0.29	0	0.99	0.19	0.19	0	0.15	0.15	0.37	0.02
001	7	2	7	0	0.33	0.72	0.1	0.38	0.97	0.35	0	0.43	0.92	0.3	0.11	0.6	0.84	0.06	0	0.96	0.28	0.04	0	0	0	0	0	0	0	0	0	0	0	0	1	0	1	0.03	0.29	0	0.99	0.19	0.19	0	0.15	0.15	0.37	0.02
001	7	2	8	0	0.33	0.72	0.1	0.38	0.97	0.35	0	0.43	0.92	0.3	0.11	0.6	0.83	0.06	0	0.96	0.28	0.04	0	0	0	0	0	0	0	0	0	0	0	0	1	0	1	0.03	0.29	0	0.99	0.19	0.19	0	0.15	0.15	0.37	0.02
001	7	2	9	0	0.33	0.72	0.1	0.38	0.97	0.35	0	0.43	0.92	0.3	0.11	0.6	0.83	0.06	0	0.96	0.28	0.04	0	0	0	0	0	0	0	0	0	0	0	0	1	0	1	0.03	0.29	0	0.99	0.19	0.19	0	0.15	0.15	0.37	0.02
001	8	1	4	0	0.33	0.72	0.1	0.38	0.97	0.35	0	0.43	0.92	0.3	0.11	0.6	0.84	0.06	0	0.96	0.28	0.04	0	0	0	0	0	0	0	0	0	0	0	0	1	0	1	0.03	0.29	0	0.99	0.19	0.19	0	0.15	0.15	0.37	0.02
001	8	1	5	0	0.33	0.72	0.1	0.38	0.97	0.35	0	0.43	0.92	0.3	0.11	0.6	0.84	0.06	0	0.96	0.28	0.04	0	0	0	0	0	0	0	0	0	0	0	0	1	0	1	0.03	0.29	0	0.99	0.19	0.19	0	0.15	0.15	0.37	0.02
001	8	1	6	0	0.33	0.72	0.1	0.38	0.97	0.35	0	0.43	0.92	0.3	0.11	0.6	0.84	0.06	0	0.96	0.28	0.04	0	0	0	0	0	0	0	0	0	0	0	0	1	0	1	0.03	0.29	0	0.99	0.19	0.19	0	0.15	0.15	0.37	0.02
001	8	1	7	0	0.33	0.72	0.1	0.38	0.97	0.35	0	0.43	0.92	0.3	0.11	0.6	0.84	0.06	0	0.96	0.28	0.04	0	0	0	0	0	0	0	0	0	0	0	0	1	0	1	0.03	0.29	0	0.99	0.19	0.19	0	0.15	0.15	0.37	0.02
001	8	1	8	0	0.33	0.72	0.1	0.38	0.97	0.35	0	0.43	0.92	0.3	0.11	0.6	0.84	0.06	0	0.96	0.28	0.04	0	0	0	0	0	0	0	0	0	0	0	0	1	0	1	0.03	0.29	0	0.99	0.19	0.19	0	0.15	0.15	0.37	0.02
001	14	1	4	0	0.33	0.72	0.1	0.38	0.97	0.35	0	0.43	0.92	0.3	0.11	0.6	0.84	0.06	0	0.96	0.28	0.04	0	0	0	0	0	0	0	0	0	0	0	0	1	0	1	0.03	0.29	0	0.99	0.19	0.19	0	0.15	0.15	0.37	0.02
001	14	1	5	0	0.33	0.72	0.1	0.38	0.97	0.35	0	0.43	0.92	0.3	0.11	0.6	0.84	0.06	0	0.96	0.28	0.04	0	0	0	0	0	0	0	0	0	0	0	0	1	0	1	0.03	0.29	0	0.99	0.19	0.19	0	0.15	0.15	0.37	0.02
001	14	1	6	0	0.33	0.72	0.1	0.38	0.97	0.35	0	0.43	0.92	0.3	0.11	0.6	0.84	0.06	0	0.96	0.28	0.04	0	0	0	0	0	0	0	0	0	0	0	0	1	0	1	0.03	0.29	0	0.99	0.19	0.19	0	0.15	0.15	0.37	0.02
001	14	1	7	0	0.33	0.72	0.1	0.38	0.97	0.35	0	0.43	0.92	0.3	0.11	0.6	0.84	0.06	0	0.96	0.28	0.04	0	0	0	0	0	0	0	0	0	0	0	0	1	0	1	0.03	0.29	0	0.99	0.19	0.19	0	0.15	0.15	0.37	0.02
001	14	1	8	0	0.33	0.72	0.1	0.38	0.97	0.35	0	0.43	0.92	0.3	0.11	0.6	0.84	0.06	0	0.96	0.28	0.04	0	0	0	0	0	0	0	0	0	0	0	0	1	0	1	0.03	0.29	0	0.99	0.19	0.19	0	0.15	0.15	0.37	0.02
001	21	1	5	0.04	0.98	0.45	0	0.27	0.32	0.99	0	0.89	0.93	0.27	0.97	0.89	0.97	0.96	0	0.96	0.57	0.81	0.94	0.97	0.94	0.96	0.98	0.95	0.95	0.86	0.83	0.95	0.95	0	0.09	0	0	0.03	0.2	0	0.34	0	0	0.94	0.22	0.22	0.22	0.02
001	21	1	6	0	0.33	0.72	0.1	0.38	0.97	0.35	0	0.43	0.92	0.3	0.11	0.6	0.84	0.06	0	0.96	0.28	0.04	0	0	0	0	0	0	0	0	0	0	0	0	1	0	1	0.03	0.29	0	0.99	0.19	0.19	0	0.15	0.15	0.37	0.02
001	21	1	7	0	0.33	0.72	0.1	0.38	0.97	0.35	0	0.43	0.92	0.3	0.11	0.6	0.83	0.06	0	0.96	0.28	0.04	0	0	0	0	0	0	0	0	0	0	0	0	1	0	1	0.03	0.29	0	0.99	0.19	0.19	0	0.15	0.15	0.37	0.02
001	21	1	8	0	0.33	0.72	0.1	0.38	0.97	0.35	0	0.42	0.92	0.29	0.11	0.6	0.84	0.06	0	0.96	0.28	0.04	0	0	0	0	0	0	0	0	0	0	0	0	1	0	1	0.03	0.29	0	0.99	0.19	0.19	0	0.15	0.15	0.37	0.02
001	21	1	9	0	0.33	0.72	0.1	0.38	0.97	0.35	0	0.43	0.92	0.3	0.11	0.6	0.83	0.06	0	0.96	0.28	0.04	0	0	0	0	0	0	0	0	0	0	0	0	1	0	1	0.03	0.29	0	0.99	0.19	0.19	0	0.15	0.15	0.37	0.02
006	-1	1	10	0	0.33	0.88	0.1	0.38	0.97	0.35	0	0.43	0.92	0.29	0.11	0.6	0.84	0.06	0	0.96	0.28	0.04	0	0	0	0	0	0	0	0	0	0	0	0	1	0	1	0.03	0.29	0	0.99	0.19	0.19	0	0.14	0.14	0.37	0.88
006	-1	1	11	0	0.33	0.88	0.1	0.38	0.97	0.35	0	0.43	0.92	0.29	0.11	0.6	0.84	0.06	0	0.96	0.28	0.04	0	0	0	0	0	0	0	0	0	0	0	0	1	0	1	0.03	0.29	0	0.99	0.19	0.19	0	0.14	0.14	0.37	0.88
006	-1	1	12	0	0.33	0.88	0.1	0.38	0.97	0.35	0	0.43	0.92	0.29	0.11	0.6	0.84	0.06	0	0.96	0.28	0.04	0	0	0	0	0	0	0	0	0	0	0	0	1	0	1	0.03	0.29	0	0.99	0.19	0.19	0	0.14	0.14	0.37	0.88
006	-1	1	2	0	0.33	0.88	0.1	0.38	0.97	0.35	0	0.43	0.92	0.29	0.11	0.6	0.83	0.06	0	0.96	0.28	0.04	0	0	0	0	0	0	0	0	0	0	0	0	1	0	1	0.03	0.29	0	0.99	0.19	0.19	0	0.14	0.14	0.37	0.88
006	-1	1	3	0	0.33	0.88	0.1	0.38	0.97	0.35	0	0.43	0.92	0.29	0.11	0.6	0.84	0.06	0	0.96	0.28	0.04	0	0	0	0	0	0	0	0	0	0	0	0	1	0	1	0.03	0.29	0	0.99	0.19	0.19	0	0.14	0.14	0.37	0.88
006	-1	1	4	0	0	0.79	0.3	0.27	0.32	0.99	1	0.13	0.92	0.14	0.99	0.89	0.95	0.43	0	0.29	0.93	0.53	0.76	0	0	0.92	0	0.81	0.92	0.04	0	0	0	0	1	1	1	0.03	0.34	0	0.18	0	0.19	0.06	0.17	0.17	0.13	0.15
006	-1	1	5	0	0	0.88	0.1	0.38	0.97	0.35	0	0.43	0.92	0.29	0.11	0.6	0.84	0.06	0	0.96	0.28	0.04	0	0	0	0	0	0	0	0	0	0	0	0	1	0	1	0.03	0.29	0	0.99	0.19	0	0	0.14	0.14	0.37	0.88
006	-1	1	6	0	0.33	0.88	0.1	0.38	0.97	0.35	0	0.43	0.92	0.29	0.11	0.6	0.84	0.06	0	0.96	0.28	0.04	0	0	0	0	0	0	0	0	0	0	0	0	1	0	1	0.03	0.29	0	0.99	0.19	0.19	0	0.14	0.14	0.37	0.88
006	-1	1	7	0	0.33	0.88	0.1	0.38	0.97	0.35	0	0.43	0.92	0.29	0.11	0.6	0.83	0.06	0	0.96	0.28	0.04	0	0	0	0	0	0	0	0	0	0	0	0	1	0	1	0.03	0.29	0	0.99	0.19	0.19	0	0.14	0.14	0.37	0.88
006	-1	1	8	0	0.33	0.88	0.1	0.38	0.97	0.35	0	0.43	0.92	0.29	0.11	0.6	0.83	0.06	0	0.96	0.28	0.04	0	0	0	0	0	0	0	0	0	0	0	0	1	0	1	0.03	0.29	0	0.99	0.19	0.19	0	0.14	0.14	0.37	0.88
006	1	1	10E	0	0.98	0.9	0	0.27	0.32	1	0	0.1	0.85	0.1	0.22	0.58	0.96	0.96	0	0.21	0.74	0.96	0.47	0	0	0.74	0	0.94	0.95	0.05	0.82	0.24	0	0.61	1	0	1	1	0.2	0	0.21	0	0	1	1	1	0.1	1
006	1	1	1E	0	0.98	0.8	0	0.27	0.32	1	0	0.04	1	0.04	0.22	0.59	0.96	0.96	0	0.21	0.74	0.96	0.47	0	0	0.74	0	0.94	0.95	0.05	0.82	0.24	0	0.99	1	0	1	1	0.2	0.05	0.04	0	0.19	1	1	1	0.04	1
006	1	1	2E	0	0.98	0.87	0.99	0.27	0.32	1	0	0.3	1	1	0.22	0.59	0.96	0.96	1	0.21	0.74	0.96	0.47	0	0	0.74	0	0.94	0.95	0.05	0.82	0.24	0	0	1	0	1	1	0.47	0	0.3	0	0.19	1	1	1	0.39	1
006	1	1	3E	0	0.98	0.9	0.99	0.27	0.32	1	0	0.04	1	0.04	0.22	0.59	0.96	0.96	0	0.21	0.74	0.96	0.47	0	0	0.74	0	0.94	0.95	0.05	0.82	0.24	0	1	1	0	1	1	0.81	0	0.04	0	0.19	1	1	1	0.04	1
006	1	1	4E	0	0.98	0.89	0.99	0.27	0.32	1	0	0.04	1	0.04	0.22	0.59	0.96	0.96	1	0.21	0.74	0.96	0.47	0	0	0.74	0	0.94	0.95	0.05	0.82	0.24	0	0	1	0	1	1	0.69	0	0.04	0	0.19	1	1	1	0.04	1
006	1	1	5E	0	0.98	0.87	0.99	0.27	0.32	1	0	0.04	1	0.04	0.22	0.59	0.96	0.96	0	0.21	0.74	0.96	0.47	0	0	0.74	0	0.94	0.95	0.05	0.82	0.24	0	0.61	1	0	1	1	0.47	0	0.04	0.91	0.68	1	1	1	0.04	1
006	1	1	6E	0	0.98	0.89	0	0.27	0.32	1	0	0.04	1	0.04	0.22	0.59	0.96	0.96	0	0.21	0.74	0.96	0.47	0	0	0.74	0	0.94	0.95	0.05	0.82	0.24	0	0.79	1	0	1	1	0.2	0	0.04	0.87	0.65	1	1	1	0.04	1
006	1	1	7E	0	0.98	0.88	0	0.27	0.32	1	0	0.04	1	0.04	0.22	0.59	0.96	0.96	0	0.21	0.74	0.96	0.47	0	0	0.74	0	0.94	0.95	0.05	0.82	0.24	0	1	1	0	1	1	0.2	0	0.04	0	0.19	1	1	1	0.04	1
006	1	1	8E	0	0.98	0.89	0.99	0.27	0.32	1	0	0.04	1	0.04	0.22	0.59	0.96	0.96	0	0.21	0.74	0.96	0.47	0	0	0.74	0	0.94	0.95	0.05	0.82	0.24	0	1	1	0	1	1	0.47	0	0.04	0	0.19	1	1	1	0.04	1
006	1	1	9E	0	0.88	0.88	0.99	0.27	0.32	1	0	0.3	1	1	0.22	0.59	0.96	0.96	0	0.21	0.74	0.96	0.47	0	0	0.74	0	0.94	0.95	0.05	0.82	0.24	0	1	1	0	1	1	0.47	0	0.3	0.65	1	1	1	1	0.39	1
006	3	1	11E	0	0.98	0.87	0.99	0.27	0.32	1	0	0.04	1	0.04	0.22	0.59	0.96	0.96	0	0.21	0.74	0.96	0.47	0	0	0.74	0	0.94	0.95	0.05	0.82	0.24	0	0	1	0	1	1	0.47	0	0.04	0.91	0.68	1	1	1	0.04	1

006	3	1	12E	0	0.98	0.89	0	0.27	0.32	1	0	0.04	1	0.04	0.22	0.59	0.96	0.96	0	0.21	0.74	0.96	0.47	0	0	0.74	0	0.94	0.95	0.05	0.82	0.24	0	0.4	1	0	1	1	0.2	0	0.04	0	0.19	1	1	1	0.04	1
006	3	1	1E	0	0.98	0.85	0	0.27	0.32	1	0	0.04	1	0.04	0.22	0.59	0.96	0.96	1	0.21	0.74	0.96	0.47	0	0	0.74	0	0.94	0.95	0.05	0.82	0.24	0	0.82	1	0	1	1	0.2	0	0.04	0	0.19	1	1	1	0.04	1
006	3	1	3E	0	0.98	0.89	0	0.27	0.32	1	0	0.31	1	1	0.22	0.59	0.96	0.96	0	0.21	0.74	0.96	0.47	0	0	0.74	0	0.94	0.95	0.05	0.82	0.24	0	1	1	0	1	1	0.2	0	0.3	0	0.19	1	1	1	0.39	1
006	3	1	4E	0.97	0.98	0.9	0.99	0.27	0.32	1	0	0.04	1	0.04	0.22	0.59	0.96	0.96	0	0.21	0.74	0.96	0.47	0	0	0.74	0	0.94	0.95	0.05	0.82	0.24	0	0.41	1	0	1	1	0.81	0	0.04	0.65	1	1	1	1	0.04	1
006	3	1	5E	0	0.98	0.89	0.99	0.27	0.32	1	0	0.04	1	0.04	0.22	0.59	0.96	0.96	0	0.21	0.74	0.96	0.47	0	0	0.74	0	0.94	0.95	0.05	0.82	0.24	0	0	1	0	1	1	0.47	0	0.04	0	0.19	1	1	1	0.04	1
006	3	1	6E	0.97	0.98	0.87	0	0.27	0.32	1	0	0.04	1	0.04	0.22	0.59	0.96	0.96	0	0.21	0.74	0.96	0.47	0	0	0.74	0	0.94	0.95	0.05	0.82	0.24	0	0	1	0	1	1	0.4	0	0.04	0.91	0.68	1	1	1	0.04	1
006	3	1	7E	0	0.98	0.63	0	0.27	0.32	1	0	0.31	1	1	0.22	0.59	0.96	0.96	0	0.21	0.74	0.96	0.47	0	0	0.74	0	0.94	0.95	0.05	0.82	0.24	0	0	1	0	1	1	0.2	0	0.3	0.91	0.68	1	1	1	0.39	1
006	3	1	8E	0	0.98	0.87	0	0.27	0.32	1	0	0.04	1	0.04	0.22	0.59	0.96	0.96	0	0.21	0.74	0.96	0.47	0	0	0.74	0	0.94	0.95	0.05	0.82	0.24	0	0	1	0	1	1	0.2	0	0.04	0.91	0.68	1	1	1	0.04	1
006	3	1	9E	0	0.98	0.63	0	0.27	0.32	1	0	0.04	1	0.04	0.22	0.59	0.96	0.96	0	0.21	0.74	0.96	0.47	0	0	0.74	0	0.94	0.95	0.05	0.82	0.24	0	0.61	1	0	1	1	0.2	0	0.04	0.91	0.68	1	1	1	0.04	1
006	3	2	10	0	0.98	0.87	0	0.27	0.32	1	0	0.04	1	0.04	0.22	0.59	0.96	0.96	0	0.21	0.74	0.96	0.47	0	0	0.74	0	0.94	0.95	0.05	0.82	0.24	0	0.82	1	0	1	1	0.81	0	0.04	0	0	1	1	1	0.04	1
006	3	2	1	0	0.98	0.76	0	0.27	0.32	1	0	0.04	1	0.04	0.22	0.59	0.96	0.96	0	0.21	0.74	0.96	0.47	0	0	0.74	0	0.94	0.95	0.05	0.82	0.24	0	1	1	0	1	1	0.2	0	0.04	0	0.19	1	0.51	0.51	0.04	1
006	3	2	2	0	0.98	0.89	0.99	0.27	0.32	1	0	0.31	1	1	0.22	0.59	0.96	0.96	0	0.21	0.74	0.96	0.47	0	0	0.74	0	0.94	0.95	0.05	0.82	0.24	0	1	1	0	1	1	0.47	0	0.3	0	0.19	1	1	1	0.39	1
006	3	2	3	0.97	0.98	0.87	0	0.27	0.32	1	0	0.08	1	0.09	0.22	0.59	0.96	0.96	1	0.21	0.74	0.96	0.47	0	0	0.74	0	0.94	0.95	0.05	0.82	0.24	0	1	1	0	1	1	0.2	0	0.08	0.91	0.68	1	1	1	0.06	0.91
006	3	2	4	0	0.98	0.87	0	0.27	0.32	1	0	0.04	1	0.04	0.22	0.59	0.96	0.96	0	0.21	0.74	0.96	0.47	0	0	0.74	0	0.94	0.95	0.05	0.82	0.24	0	1	1	0	1	1	0.2	0	0.04	0	0.19	1	1	1	0.04	1
006	3	2	5	0	0.98	0.9	0	0.27	0.32	1	0	0.04	1	0.04	0.22	0.59	0.96	0.96	0	0.21	0.74	0.96	0.47	0	0	0.74	0	0.94	0.95	0.05	0.82	0.24	0	1	1	0	1	1	0.47	0	0.04	0	0.19	1	1	1	0.04	1
006	3	2	6	0	0.98	0.88	0.99	0.27	0.32	1	0	0.04	1	0.04	0.22	0.59	0.96	0.96	0	0.21	0.74	0.96	0.47	0	0	0.74	0	0.94	0.95	0.05	0.82	0.24	0	0.47	1	0	1	1	0.47	0	0.04	0.91	0.68	1	1	1	0.04	1
006	3	2	7	0.97	0.98	0.84	0	0.27	0.32	0.99	0	0.04	1	0.04	0.22	0.59	0.96	0.96	0	0.21	0.74	0.96	0.47	0	0	0.74	0	0.94	0.95	0.05	0.82	0.24	0	1	1	0	1	1	0.81	0	0.04	0.91	0.68	1	1	1	0.04	1
006	3	2	8	0	0.98	0.89	0.99	0.27	0.32	0.99	0	0.04	1	0.04	0.22	0.59	0.96	0.96	0.99	0.21	0.74	0.96	0.47	0	0	0.74	0	0.94	0.95	0.05	0.82	0.24	0	0	1	0	1	1	0.81	0	0.04	0	0.19	1	1	1	0.03	1
006	3	2	9	0	0.98	0.63	0	0.27	0.32	1	0	0.04	1	0.04	0.22	0.59	0.96	0.96	0	0.21	0.74	0.96	0.47	0	0	0.74	0	0.94	0.95	0.05	0.82	0.24	0	1	1	0	1	1	0.2	0	0.04	0	0	1	1	1	0.04	1
006	4	1	10E	0	0.98	0.63	0	0.27	0.32	1	0	0.31	1	1	0.22	0.59	0.96	0.96	0	0.21	0.74	0.96	0.47	0	0	0.74	0	0.94	0.95	0.05	0.82	0.24	0	0	1	0	1	1	0.2	0	0.3	0	0.19	1	1	1	0.39	1
006	4	1	11E	0	0.98	0.89	0	0.27	0.32	1	0	0.04	1	0.04	0.22	0.59	0.96	0.96	1	0.21	0.74	0.96	0.47	0	0	0.74	0	0.94	0.95	0.05	0.82	0.24	0	0.59	1	0	1	0.83	0.2	0	0.04	0.91	0.68	0	1	1	0.04	1
006	4	1	1E	0	0.98	0.9	0.99	0.27	0.32	0.99	0	0.04	1	0.04	0.22	0.59	0.96	0.96	1	0.21	0.74	0.96	0.47	0	0	0.74	0	0.94	0.95	0.05	0.82	0.24	0	0.43	1	0	1	1	0.47	0	0.04	0	0.19	1	1	1	0.04	1
006	4	1	2E	0	0.98	0.88	0	0.27	0.32	1	0	0.04	1	0.04	0.22	0.59	0.96	0.96	0	0.21	0.74	0.96	0.47	0	0	0.74	0	0.94	0.95	0.05	0.82	0.24	0	0	1	0	1	1	0.81	0	0.04	0	0.19	1	1	1	0.04	1
006	4	1	3E	0.97	0.98	0.9	0	0.27	0.32	1	0	0.04	1	0.04	0.22	0.59	0.96	0.96	0	0.21	0.74	0.96	0.47	0	0	0.74	0	0.94	0.95	0.05	0.82	0.24	0	0	1	0	1	1	0.2	0	0.04	0	0.19	1	1	1	0.04	1
006	4	1	4E	0.97	0.98	0.87	0	0.27	0.32	1	0	0.31	1	1	0.22	0.59	0.96	0.96	0	0.21	0.74	0.96	0.47	0	0	0.74	0	0.94	0.95	0.05	0.82	0.24	0	0.79	1	0	1	1	0.2	0	0.3	0.91	0.68	1	1	1	0.39	1
006	4	1	5E	0	0.98	0.87	0	0.27	0.32	1	0	0.04	1	0.04	0.22	0.59	0.96	0.96	0	0.21	0.74	0.96	0.47	0	0	0.74	0	0.94	0.95	0.05	0.82	0.24	0	0.46	1	0	1	1	0.2	0	0.04	0	0.19	1	1	1	0.04	1
006	4	1	7E	0	0.98	0.64	0.34	0.38	0.97	1	0	0.04	1	0.04	0.22	0.59	0.43	0.96	0	0.96	0.74	0.96	0.47	0	0	0.74	0	0.94	0.95	0.05	0.82	0.24	0	1	1	0	1	1	0.2	0	0.04	0.91	0.68	1	1	1	0.04	1
006	4	1	8E	0	0.98	0.89	0.99	0.27	0.32	1	0	0.31	1	1	0.22	0.59	0.96	0.96	1	0.21	0.74	0.96	0.47	0	0	0.74	0	0.94	0.95	0.05	0.82	0.24	0	0.41	1	0	1	1	0.47	0	0.3	0	0.19	1	1	1	0.39	1
006	4	1	9E	0	0.98	0.87	0.99	0.27	0.32	1	0	0.04	1	0.04	0.22	0.59	0.96	0.96	1	0.21	0.74	0.96	0.47	0	0	0.74	0	0.94	0.95	0.05	0.82	0.24	0	0.62	1	0	1	1	0.47	0	0.04	0.91	1	1	1	1	0.04	1
006	4	2	10	0	0.33	0.72	0.1	0.38	0.97	0.35	0	0.43	0.92	0.3	0.11	0.6	0.84	0.06	0	0.96	0.28	0.04	0	0	0	0	0	0	0	0	0	0	0	0	1	0	1	0.03	0.29	0	0.99	0.19	0.19	0	0.15	0.15	0.37	0.02
006	4	2	1	0	0.33	0.72	0.1	0.38	0.97	0.35	0	0.43	0.92	0.3	0.11	0.6	0.84	0.06	0	0.96	0.28	0.04	0	0	0	0	0	0	0	0	0	0	0	0	1	0	1	0.03	0.29	0	0.99	0.19	0.19	0	0.15	0.15	0.37	0.02
006	4	2	2	0	0.33	0.72	0.1	0.38	0.97	0.35	0	0.43	0.92	0.3	0.11	0.6	0.84	0.06	0	0.96	0.28	0.04	0	0	0	0	0	0	0	0	0	0	0	0	1	0	1	0.03	0.29	0	0.99	0.19	0.19	0	0.15	0.15	0.37	0.02
006	4	2	3	0	0.33	0.72	0.1	0.38	0.97	0.35	0	0.43	0.92	0.3	0.11	0.6	0.84	0.06	0	0.96	0.28	0.04	0	0	0	0	0	0	0	0	0	0	0	0	1	0	1	0.03	0.29	0	0.99	0.19	0.19	0	0.15	0.15	0.37	0.02
006	4	2	4	0	0.33	0.72	0.1	0.38	0.97	0.35	0	0.43	0.21	0.3	0.11	0.05	0.83	0.06	0	0.96	0.28	0.04	0	0	0	0	0	0	0	0	0	0	0	0	1	0	1	0.03	0.29	0	0.99	0.19	0.19	0	0.15	0.15	0.37	0.02
006	4	2	5	0	0.33	0.72	0.1	0.38	0.97	0.35	0	0.43	0.92	0.3	0.11	0.6	0.84	0.06	0	0.96	0.28	0.04	0	0	0	0	0	0	0	0	0	0	0	0	1	0	1	0.03	0.29	0	0.99	0.19	0.19	0	0.15	0.15	0.37	0.02
006	4	2	6	0	0.33	0.72	0.1	0.38	0.97	0.35	0	0.43	0.92	0.3	0.11	0.6	0.84	0.06	0	0.96	0.28	0.04	0	0	0	0	0	0	0	0	0	0	0	0	1	0	1	0.03	0.29	0	0.99	0.19	0.19	0	0.15	0.15	0.37	0.02
006	4	2	7	0	0.33	0.72	0.1	0.38	0.97	0.35	0	0.43	0.92	0.3	0.11	0.6	0.84	0.06	0	0.96	0.28	0.04	0	0	0	0	0	0	0	0	0	0	0	0	1	0	1	0.03	0.29	0	0.76	0.19	0.19	0	0.15	0.15	0.37	0.02
006	4	2	8	0	0.33	0.72	0.1	0.38	0.97	0.35	0	0.43	0.92	0.3	0.11	0.6	0.84	0.06	0	0.96	0.28	0.04	0	0	0	0	0	0	0	0	0	0	0	0	1	0	1	0.03	0.29	0	0.99	0.19	0.19	0	0.15	0.15	0.37	0.02
006	4	2	9	0	0	0.72	0.1	0.38	0.97	0.35	0	0.43	0.92	0.3	0.11	0.6	0.84	0.06	0	0.96	0.28	0.04	0	0	0	0	0	0	0	0	0	0	0	0	1	0	1	0.03	0.29	0	0.99	0.19	0	0	0.15	0.15	0.37	0.02
006	5	1	1	0	0.33	0.88	0.1	0.38	0.97	0.35	0	0.43	0.92	0.29	0.11	0.6	0.83	0.06	0	0.96	0.28	0.04	0	0	0	0	0	0	0	0	0	0	0	0	1	0	1	0.03	0.29	0	0.99	0.19	0.19	0	0.14	0.14	0.37	0.88
006	5	1	2	0	0.33	0.88	0.1	0.38	0.97	0.35	0	0.43	0.92	0.29	0.11	0.6	0.84	0.06	0	0.96	0.28	0.04	0	0	0	0	0	0	0	0	0	0	0	0	1	0	1	0.03	0.29	0	0.99	0.19	0.19	0	0.14	0.14	0.37	0.88
006	5	1	3	0	0.33	0.88	0.1	0.38	0.97	0.35	0	0.43	0.92	0.29	0.11	0.6	0.84	0.06	0	0.21	0.28	0.04	0	0	0	0	0	0	0	0	0	0	0	0	1	0	1	0.03	0.29	0	0.49	0.19	0.19	0	0.14	0.14	0.37	0.88
006	5	1	4	0	0.33	0.88	0.1	0.38	0.97	0.35	0	0.43	0.92	0.29	0.11	0.6	0.84	0.06	0	0.96	0.28	0.04	0	0	0	0	0	0	0	0	0	0	0	0	1	0	1	0.03	0.29	0	0.99	0.19	0.19	0	0.14	0.14	0.37	0.88
006	5	1	5	0	0.33	0.88	0.1	0.38	0.97	0.35	0	0.43	0.92	0.29	0.11	0.6	0.83	0.06	0	0.96	0.28	0.04	0	0	0	0	0	0	0	0	0	0	0	0	1	0	1	0.03	0.29	0	0.99	0.19	0.19	0	0.14	0.14	0.37	0.88
006	5	1	6	0	0.33	0.88	0.1	0.38	0.97	0.35	0	0.43	0.92	0.29	0.11	0.6	0.84	0.06	0	0.96	0.28	0.04	0	0	0	0	0	0	0	0	0	0	0	0	1	0	1	0.03	0.29	0	0.99	0.19	0.19	0	0.14	0.14	0.37	0.88
006	5	1	7	0	0.33	0.88	0.1	0.38	0.97	0.35	0	0.43	0.92	0.29	0.11	0.6	0.84	0.06	0	0.96	0.28	0.04	0	0	0	0	0	0	0	0	0	0	0	0	1	0	1	0.03	0.29	0	0.99	0.19	0.19	0	0.14	0.14	0.37	0.88
006	6	1	10	0	0.33	0.88	0.1	0.38	0.97	0.35	0	0.43	0.85	0.29	0.11	0.6	0.83	0.06	0	0.96	0.28	0.04	0	0	0	0	0	0	0	0	0	0	0	0	1	0	1	0.03	0.29	0	0.99	0.19	0.19	0	0.14	0.14	0.37	0.88
006	6	1	1	0	0.33	0.88	0.1	0.38	0.97	0.35	0	0.43	0.92	0.29	0.06	0.6	0.13	0.06	0	0.15	0.28	0.04	0	0	0	0	0	0	0	0	0	0	0	0	1	0	1	0.03	0.29	0	0.49	0.21	0.19	0	0.14	0.14	0.37	0.88
006	6	1	2	0	0.33	0.88	0.1	0.38	0.97	0.35	0	0.43	0.92	0.29	0.11	0.6	0.84	0.06	0	0.21	0.28	0.04	0	0	0	0	0	0	0	0	0	0	0	0	1	0	1	0.03	0.29	0	0.49	0.19	0.19	0	0.14	0.14	0.37	0.88
006	6	1	3	0	0.33	0.88	0.1	0.38	0.97	0.35	0	0.43	0.92	0.29	0.11	0.6	0.84	0.06	0	0.21	0.28	0.04	0	0	0	0	0	0	0	0	0	0	0	0	1	0	1	0.03	0.29	0	0.49	0.19	0.19	0	0.14	0.14	0.37	0.88
006	6	1	4	0	0.33	0.88	0.1	0.38	0.97	0.35	0	0.43	0.92	0.29	0.11	0.6	0.83	0.06	0	0.21	0.28	0.04	0	0	0	0	0	0	0	0	0	0	0	0	1	0	1	0.03	0.29	0	0.49	0.19	0.19	0	0.14	0.14	0.37	0.88
006	6	1	5	0	0.33	0.88	0.1	0.38	0.97	0.35	0	0.43	0.92	0.29	0.11	0.6	0.84	0.06	0	0.96	0.28	0.04	0	0	0	0	0	0	0	0	0	0	0	0	1	0	1	0.03	0.29	0	0.99	0.19	0.19	0	0.14	0.14	0.37	0.88
006	6	1	6	0	0.33	0.88	0.1	0.38	0.97	0.35	0	0.43	0.92	0.29	0.11	0.6	0.84	0.06	0	0.96	0.28	0.04	0	0	0	0	0	0	0	0	0	0	0	0	1	0	1	0.03	0.29	0	0.99	0.19	0.19	0	0.14	0.14	0.37	0.88
006	6	1	7	0	0.33	0.88	0.1	0.38	0.97	0.35	0	0.43	0.92	0.29	0.11	0.6	0.84	0.06	0	0.21	0.28	0.04	0	0	0	0	0	0	0	0	0	0	0	0	1	0	1	0.03	0.29	0	0.49	0.19	0.19	0	0.14	0.14	0.37	0.88
006	6	1	8	0	0.33	0.88	0.1	0.38	0.97	0.35	0	0.43	0.92	0.29	0.11	0.6	0.83	0.06	0	0.21	0.28	0.04	0	0	0	0	0	0	0	0	0	0	0	0	1	0	1	0.03	0.29	0	0.49	0.19	0.19	0	0.14	0.14	0.37	0.88
006	6	1	9	0	0.33	0.88	0.1	0.38	0.97	0.35	0	0.43	0.92	0.29	0.11	0.6	0.84	0.06	0	0.96	0.28	0.04	0	0	0	0	0	0	0	0	0	0	0	0	1	0	1	0.03	0.29	0	0.99	0.19	0.19	0	0.14	0.14	0.37	0.88
006	6	2	10	0	0.33	0.88	0.1	0.38	0.97	0.35	0	0.43	0.92	0.29	0.11	0.6	0.84	0.06	0	0.21	0.28	0.04	0	0	0	0	0	0	0	0	0	0	0	0	1	0	1	0.03	0.29	0	0.49	0.19	0.19	0	0.14	0.14	0.37	0.88
006	6	2	1	0	0.33	0.88	0.1	0.38	0.97	0.35	0	0.43	0.92	0.29	0.11	0.6	0.84	0.06	0	0.21	0.28	0.04	0	0	0	0	0	0	0	0	0	0	0	0	1	0	1	0.03	0.29	0	0.49	0.19	0.19	0	0.14	0.14	0.37	0.88
006	6	2	2	0	0.33	0.88	0.1	0.38	0.97	0.35	0	0.43	0.92	0.29	0.11	0.6	0.84	0.06	0	0.21	0.28	0.04	0	0	0	0	0	0	0	0	0	0	0	0	1	0	1	0.03	0.29	0	0.49	0.19	0.19	0	0.14	0.14	0.37	0.88
006	6	2	3	0	0.33	0.88	0.1	0.38	0.97	0.35	0	0.43	0.92	0.29	0.11	0.6	0.84	0.06	0	0.21	0.28	0.04	0	0	0	0	0	0	0	0	0	0	0	0	1	0	1	0.03	0.29	0	0.49	0.19	0.19	0	0.14	0.14	0.37	0.88
006	6	2	4	0	0.33	0.88	0.1	0.38	0.97	0.35	0	0.43	0.92	0.29	0.11	0.6	0.84	0.06	0	0.21	0.28	0.04	0	0	0	0	0	0	0	0	0	0	0	0	1	0	1	0.03	0.29	0	0.49	0.19	0.19	0	0.14	0.14	0.37	0.88
006	6	2	5	0	0.33	0.88	0.1	0.38	0.97	0.35	0	0.43	0.92	0.29	0.11	0.6	0.84	0.06	0	0.21	0.28	0.04	0	0	0	0	0	0	0	0	0	0	0	0	1	0	1	0.03	0.29	0	0.49	0.19	0.19	0	0.14	0.14	0.37	0.88
006	6	2	6	0	0.33	0.88	0.1	0.38	0.97	0.35	0	0.43	0.92	0.29	0.11	0.6	0.84	0.06	0	0.21	0.28	0.04	0	0	0	0	0	0	0	0	0	0	0	0	1	0	1	0.03	0.29	0	0.49	0.19	0.19	0	0.14	0.14	0.37	0.88
006	6	2	7	0	0.33	0.88	0.1	0.38	0.97	0.35	0	0.43	0.92	0.29	0.11	0.6	0.83	0.06	0	0.21	0.28	0.04	0	0	0	0	0	0	0	0	0	0	0	0	1	0	1	0.03	0.29	0	0.49	0.19	0.19	0	0.14	0.14	0.37	0.88
006	6	2	8	0	0.33	0.88	0.1	0.38	0.97	0.35	0	0.43	0.92	0.29	0.11	0.6	0.84	0.06	0	0.21	0.28	0.04	0	0	0	0	0	0	0	0	0	0	0	0	1	0	1	0.03	0.29	0	0.49	0.19	0.19	0	0.14	0.14	0.37	0.88
006	6	2	9	0	0.33	0.88	0.1	0.38	0.97	0.35	0	0.43	0.92	0.29	0.11	0.6	0.84	0.06	0	0.21	0.28	0.04	0	0	0	0	0	0	0	0	0	0	0	0	1	0	1	0.03	0.29	0	0.49	0.19	0.19	0	0.14	0.14	0.37	0.88
006	7	1	10	0	0.33	0.88	0.1	0.38	0.97	0.35	0	0.43	0.92	0.29	0.11	0.6	0.84	0.06	0	0.21	0.28	0.04	0	0	0	0	0	0	0	0	0	0	0	0	1	0	1	0.03	0.29	0	0.49	0.19	0.19	0	0.14	0.14	0.37	0.88
006	7	1	1	0	0	0.88	0.1	0.38	0.97	0.35	0	0.43	0.92	0.29	0.11	0.6	0.83	0.06	0	0.21	0.28	0.04	0	0	0	0	0	0	0	0	0	0	0	0	1	0	1	0.03	0.29	0	0.49	0.19	0	0	0.14	0.14	0.37	0.88
006	7	1	2	0	0	0.88	0.1	0.38	0.97	0.35	0	0.43	0.92	0.29	0.11	0.6	0.84	0.06	0	0.21	0.28	0.04	0	0	0	0	0	0	0	0	0	0	0	0	1	0	1	0.03	0.29	0	0.49	0.19	0	0	0.14	0.14	0.37	0.88
006	7	1	3	0	0.33	0.88	0.1	0.38	0.97	0.35	0	0.43	0.92	0.29	0.11	0.6	0.84	0.06	0	0.21	0.28	0.04	0	0	0	0	0	0	0	0	0	0	0	0	1	0	1	0.03	0.29	0	0.49	0.19	0.19	0	0.14	0.14	0.37	0.88

006	7	1 4	0	0.33	0.88	0.1	0.38	0.97	0.35	0	0.43	0.92	0.29	0.11	0.6	0.84	0.06	0	0.21	0.28	0.04	0	0	0	0	0	0	0	0	0	0	0	0	1	0	1	0.03	0.29	0	0.49	0.19	0.19	0	0.14	0.14	0.37	0.88
006	7	1 5	0	0.33	0.88	0.1	0.38	0.97	0.35	0	0.43	0.92	0.29	0.11	0.6	0.84	0.06	0	0.21	0.28	0.04	0	0	0	0	0	0	0	0	0	0	0	0	1	0	1	0.03	0.29	0	0.49	0.19	0.19	0	0.14	0.14	0.37	0.88
006	7	1 6	0	0.33	0.88	0.1	0.38	0.97	0.35	0	0.43	0.92	0.29	0.11	0.6	0.84	0.06	0	0.21	0.28	0.04	0	0	0	0	0	0	0	0	0	0	0	0	1	0	1	0.03	0.29	0	0.4	0.19	0.19	0	0.14	0.14	0.37	0.88
006	7	1 7	0	0.33	0.88	0.1	0.38	0.97	0.35	0	0.43	0.92	0.29	0.11	0.6	0.83	0.06	0	0.21	0.28	0.04	0	0	0	0	0	0	0	0	0	0	0	0	1	0	1	0.03	0.29	0	0.49	0.19	0.19	0	0.14	0.14	0.37	0.88
006	8	1 11	0	0.33	0.88	0.1	0.38	0.97	0.35	0	0.43	0.92	0.29	0.11	0.6	0.84	0.06	0	0.21	0.28	0.04	0	0	0	0	0	0	0	0	0	0	0	0	1	0	1	0.03	0.29	0	0.49	0.19	0.19	0	0.14	0.14	0.37	0.88
006	8	1 12	0	0.33	0.88	0.1	0.38	0.97	0.35	0	0.43	0.92	0.29	0.11	0.6	0.84	0.06	0	0.21	0.28	0.04	0	0	0	0	0	0	0	0	0	0	0	0	1	0	1	0.03	0.29	0	0.49	0.19	0.19	0	0.14	0.14	0.37	0.88
006	8	1 1	0	0.33	0.88	0.1	0.38	0.97	0.35	0	0.43	0.92	0.29	0.11	0.6	0.84	0.06	0	0.21	0.28	0.04	0	0	0	0	0	0	0	0	0	0	0	0	1	0	1	0.03	0.29	0	0.49	0.19	0.19	0	0.14	0.14	0.37	0.88
006	8	1 2	0	0.33	0.88	0.1	0.38	0.97	0.35	0	0.43	0.92	0.29	0.06	0.6	0.18	0.06	0	0.15	0.28	0.04	0	0	0	0	0	0	0	0	0	0	0	0	1	0	1	0.03	0.29	0	0.49	0.19	0.19	0	0.14	0.14	0.37	0.88
006	8	1 6	0	0.33	0.88	0.1	0.38	0.97	0.35	0	0.43	0.92	0.29	0.11	0.6	0.84	0.06	0	0.21	0.28	0.04	0	0	0	0	0	0	0	0	0	0	0	0	1	0	1	0.03	0.29	0	0.49	0.19	0.19	0	0.14	0.14	0.37	0.88
006	8	2 1	0	0.33	0.88	0.1	0.38	0.97	0.35	0	0.43	0.92	0.29	0.11	0.6	0.83	0.06	0	0.21	0.28	0.04	0	0	0	0	0	0	0	0	0	0	0	0	1	0	1	0.03	0.29	0	0.49	0.19	0.19	0	0.14	0.14	0.37	0.88
006	8	2 2	0	0.33	0.88	0.1	0.38	0.97	0.35	0	0.43	0.92	0.29	0.11	0.6	0.83	0.06	0	0.21	0.28	0.04	0	0	0	0	0	0	0	0	0	0	0	0	1	0	1	0.03	0.29	0	0.49	0.19	0.19	0	0.14	0.14	0.37	0.88
006	8	2 3	0	0.33	0.88	0.1	0.38	0.97	0.35	0	0.43	0.92	0.29	0.11	0.6	0.83	0.06	0	0.21	0.28	0.04	0	0	0	0	0	0	0	0	0	0	0	0	1	0	1	0.03	0.29	0	0.49	0.19	0.19	0	0.14	0.14	0.37	0.88
006	8	2 4	0	0.33	0.88	0.1	0.38	0.97	0.35	0	0.43	0.92	0.29	0.11	0.6	0.83	0.06	0	0.21	0.28	0.04	0	0	0	0	0	0	0	0	0	0	0	0	1	0	1	0	0.29	0	0.49	0.19	0.19	0	0.14	0.14	0.37	0.88
006	8	2 8	0	0	0.88	0.1	0.38	0.97	0.35	0	0.43	0.92	0.29	0.11	0.6	0.84	0.06	0	0.21	0.28	0.04	0	0	0	0	0	0	0	0	0	0	0	0	1	0	1	0.03	0.29	0	0.49	0.19	0	0	0.14	0.14	0.37	0.88
006	14	1 3	0	0.33	0.88	0.1	0.38	0.97	0.35	0	0.43	0.92	0.29	0.11	0.6	0.84	0.06	0	0.21	0.28	0.04	0	0	0	0	0	0	0	0	0	0	0	0	1	0	1	0.03	0.29	0	0.49	0.19	0.19	0	0.14	0.14	0.37	0.88
006	14	1 4	0	0.33	0.88	0.1	0.38	0.97	0.35	0	0.43	0.92	0.29	0.11	0.6	0.84	0.06	0	0.21	0.28	0.04	0	0	0	0	0	0	0	0	0	0	0	0	1	0	1	0.03	0.29	0	0.49	0.19	0.19	0	0.14	0.14	0.37	0.88
006	14	1 5	0	0.33	0.88	0.1	0.38	0.97	0.35	0	0.43	0.92	0.29	0.11	0.6	0.84	0.06	0	0.21	0.28	0.04	0	0	0	0	0	0	0	0	0	0	0	0	1	0	1	0.03	0.29	0	0.49	0.19	0.19	0	0.14	0.14	0.37	0.88
006	14	1 6	0	0.33	0.88	0.1	0.38	0.97	0.35	0	0.43	0.92	0.29	0.11	0.6	0.83	0.06	0	0.21	0.28	0.04	0	0	0	0	0	0	0	0	0	0	0	0	1	0	1	0.03	0.29	0	0.49	0.19	0.19	0	0.14	0.14	0.37	0.88
006	14	1 7	0	0.33	0.88	0.1	0.38	0.97	0.35	0	0.43	0.92	0.29	0.11	0.6	0.84	0.06	0	0.21	0.28	0.04	0	0	0	0	0	0	0	0	0	0	0	0	1	0	1	0.03	0.29	0	0.34	0.19	0.19	0	0.14	0.14	0.37	0.88
006	21	1 5	0	0.33	0.88	0.1	0.38	0.97	0.35	0	0.43	0.92	0.29	0.11	0.6	0.84	0.06	0	0.21	0.28	0.04	0	0	0	0	0	0	0	0	0	0	0	0	1	0	1	0.03	0.29	0	0.49	0.19	0.19	0	0.14	0.14	0.37	0.88
006	21	1 6	0	0	0.88	0.1	0.38	0.97	0.35	0	0.43	0.92	0.29	0.11	0.6	0.84	0.06	0	0.96	0.28	0.04	0	0	0	0	0	0	0	0	0	0	0	0	1	0	1	0.03	0.29	0	0.99	0.19	0	0	0.14	0.14	0.37	0.88
006	21	1 7	0	0.33	0.88	0.1	0.38	0.97	0.35	0	0.43	0.92	0.29	0.11	0.6	0.83	0.06	0	0.21	0.28	0.04	0	0	0	0	0	0	0	0	0	0	0	0	1	0	1	0.03	0.29	0	0.49	0.19	0.19	0	0.14	0.14	0.37	0.88
006	21	1 8	0	0.33	0.88	0.1	0.38	0.97	0.35	0	0.43	0.92	0.29	0.11	0.6	0.83	0.06	0	0.21	0.28	0.04	0	0	0	0	0	0	0	0	0	0	0	0	1	0	1	0.03	0.29	0	0.49	0.19	0.19	0	0.14	0.14	0.37	0.88
006	21	1 9	0	0.33	0.88	0.1	0.38	0.97	0.35	0	0.43	0.92	0.29	0.11	0.6	0.84	0.06	0	0.21	0.28	0.04	0	0	0	0	0	0	0	0	0	0	0	0	1	0	1	0.03	0.29	0	0.49	0.19	0.19	0	0.14	0.14	0.37	0.88
006	28	1 1	0	0.33	0.88	0.1	0.38	0.97	0.35	0	0.43	0.92	0.29	0.11	0.6	0.84	0.06	0	0.21	0.28	0.04	0	0	0	0	0	0	0	0	0	0	0	0	1	0	1	0.03	0.29	0	0.49	0.19	0.19	0	0.14	0.14	0.37	0.88
006	28	1 2	0	0.33	0.88	0	0.38	0.97	0.35	0	0.43	0.92	0.29	0.11	0.6	0.83	0.06	0	0.96	0.28	0.04	0	0	0	0	0	0	0	0	0	0	0	0	1	0	1	0.03	0.29	0	0.99	0.19	0.19	0	0.14	0.14	0.37	0.88
006	28	1 3	0	0.33	0.88	0.1	0.38	0.97	0.35	0	0.43	0.79	0.29	0.11	0.6	0.84	0.06	0	0.21	0.28	0.04	0	0	0	0	0	0	0	0	0	0	0	0	1	0	1	0.03	0.29	0	0.49	0.19	0.19	0	0.14	0.14	0.37	0.88
006	28	1 7	0	0.33	0.88	0.1	0.38	0.97	0.35	0	0.43	0.92	0.29	0.11	0.6	0.84	0.06	0	0.96	0.28	0.04	0	0	0	0	0	0	0	0	0	0	0	0	1	0	1	0.03	0.29	0	0.99	0.19	0.19	0	0.14	0.14	0.37	0.88
006	28	1 8	0	0.33	0.88	0.1	0.38	0.97	0.35	0	0.43	0.92	0.29	0.11	0.6	0.84	0.06	0	0.96	0.28	0.04	0	0	0	0	0	0	0	0	0	0	0	0	1	0	1	0	0.29	0	0.99	0.19	0.19	0	0.14	0.14	0.37	0.88
008	-1	1 6	0	0.33	0.88	0.1	0.38	0.97	0.35	0	0.43	0.92	0.3	0.11	0.6	0.83	0.06	0	0.96	0.28	0.04	0	0	0	0	0	0	0	0	0	0	0	0	1	0	1	0.03	0.29	0	0.99	0.19	0.19	0	0.15	0.15	0.37	0.88
008	-1	1 7	0	0.33	0.88	0.1	0.38	0.97	0.35	0	0.43	0.92	0.3	0.11	0.6	0.84	0.06	0	0.96	0.28	0.04	0	0	0	0	0	0	0	0	0	0	0	0	1	0	1	0.03	0.29	0	0.99	0.19	0.19	0	0.15	0.15	0.37	0.88
008	-1	1 8	0	0	0.88	0.1	0.38	0.97	0.35	0	0.43	0.92	0.3	0.11	0.6	0.84	0.06	0	0.96	0.28	0.04	0	0	0	0	0	0	0	0	0	0	0	0	1	0	1	0.03	0.29	0	0.99	0.19	0.19	0	0.15	0.15	0.37	0.88
008	-1	1 9	0	0.33	0.88	0.1	0.38	0.97	0.35	0	0.43	0.92	0.3	0.11	0.6	0.84	0.06	0	0.96	0.28	0.04	0	0	0	0	0	0	0	0	0	0	0	0	1	0	1	0.03	0.29	0	0.99	0.19	0.19	0	0.15	0.15	0.37	0.88
008	0	1 1E	0	0.98	0.89	0	0.27	0.32	1	0	0.05	1	0.15	0.22	0.59	0.96	0.96	1	0.21	0.74	0.96	0.47	0	0	0.74	0	0.94	0.95	0.05	0.82	0.24	0	0	1	0	1	0.03	0.2	0	0.04	0	0.19	0	1	1	0.06	1
008	0	1 1	0	0.98	0.89	0	0.27	0.32	1	0	0.04	1	0.04	0.22	0.59	0.96	0.96	0	0.21	0.74	0.96	0.47	0	0	0.74	0	0.94	0.95	0.05	0.82	0.24	0	0.4	1	0	1	1	0.2	0	0.04	0	0.19	1	1	1	0.04	1
008	0	1 2E	0	0.98	0.65	0	0.27	0.32	1	0	0.31	1	1	0.22	0.59	0.96	0.96	0	0.21	0.74	0.96	0.47	0	0	0.74	0	0.94	0.95	0.05	0.82	0.24	0	0.82	1	0	1	1	0.81	0	0.3	0	0	1	1	1	0.39	1
008	0	1 2	0	0.98	0.89	0	0.27	0.32	1	0	0.31	1	1	0.22	0.59	0.96	0.96	0	0.21	0.74	0.96	0.47	0	0	0.74	0	0.94	0.95	0.05	0.82	0.24	0	0	1	0	1	1	0.81	0	0.3	0.91	0.68	1	1	1	0.39	1
008	0	1 3	0	0.33	0.88	0.1	0.38	0.97	0.35	0	0.43	0.92	0.3	0.11	0.6	0.83	0.06	0	0.96	0.28	0.04	0	0	0	0	0	0	0	0	0	0	0	0	1	0	1	0.03	0.29	0	0.99	0.19	0.19	0	0.15	0.15	0.37	0.88
008	1	1 10E	0.97	0.98	0.58	0.99	0.27	0.32	1	0	0.04	1	0.04	0.22	0.59	0.96	0.96	0	0.21	0.74	0.96	0.47	0	0	0.74	0	0.94	0.95	0.05	0.82	0.24	0	0	0.09	0	0.19	1	0.81	0	0.04	0.91	0.68	1	1	1	0.04	1
008	1	1 1E	0	0.98	0.59	0.99	0.27	0.32	1	0	0.31	1	1	0.22	0.59	0.96	0.96	1	0.21	0.74	0.96	0.47	0	0	0.74	0	0.94	0.95	0.05	0.82	0.24	0	0	1	0	1	1	0.47	0	0.3	0	0	1	1	1	0.39	1
008	1	1 2E	0	0.98	0.89	0	0.27	0.32	1	0	0.04	1	0.04	0.22	0.59	0.96	0.96	1	0.21	0.74	0.96	0.47	0	0	0.74	0	0.94	0.95	0.05	0.82	0.24	0	0	1	0	1	1	0.2	0	0.04	0.65	1	1	1	1	0.04	1
008	1	1 3E	0.97	0.98	0.87	0	0.27	0.32	1	0	0.04	1	0.05	0.22	0.59	0.96	0.96	1	0.21	0.74	0.96	0.47	0	0	0.74	0	0.94	0.95	0.05	0.82	0.24	0	1	1	0	1	1	0.81	0	0.04	0.52	0.37	1	1	1	0.04	1
008	1	1 4E	0.33	0.98	0.87	0	0.27	0.32	1	0	0.04	1	0.04	0.22	0.59	0.96	0.96	0	0.21	0.74	0.96	0.47	0	0	0.74	0	0.94	0.95	0.05	0.82	0.24	0	0	1	0	1	1	0.2	0	0.04	0	0.19	1	1	1	0.04	1
008	1	1 5E	0.97	0.98	0.5	0	0.27	0.32	1	0	0.04	1	0.04	0.22	0.59	0.96	0.96	0	0.21	0.74	0.96	0.47	0	0	0.74	0	0.94	0.95	0.05	0.82	0.24	0	0	1	0	1	1	0.81	0	0.04	0	0.19	1	1	1	0.04	1
008	1	1 6E	0	0.98	0.84	0.99	0.27	0.32	1	0	0.31	1	0.99	0.22	0.59	0.08	0.96	1	0.15	0.74	0.96	0.47	0	0	0.74	0	0.94	0.95	0.05	0.82	0.24	0	0	1	0	1	1	0.81	0	0.3	0.91	0.68	1	1	1	0.39	1
008	1	1 7E	0	0.98	0.87	0	0.27	0.32	1	0	0.04	1	0.04	0.22	0.59	0.96	0.96	0	0.21	0.74	0.96	0.47	0	0	0.74	0	0.94	0.95	0.05	0.82	0.24	0	0	1	0	1	1	0.2	0	0.04	0	0.19	1	1	1	0.04	1
008	1	1 8E	0	0.98	0.9	0	0.27	0.32	1	0	0.31	1	1	0.22	0.59	0.96	0.96	1	0.21	0.74	0.96	0.47	0	0	0.74	0	0.94	0.95	0.05	0.82	0.24	0	0.84	1	0	1	1	0.2	0	0.3	0	0.19	1	1	1	0.39	1
008	1	1 9E	0.97	0.98	0.89	0	0.27	0.32	1	0	0.04	1	0.04	0.22	0.59	0.96	0.96	0	0.21	0.74	0.96	0.47	0	0	0.74	0	0.94	0.95	0.05	0.82	0.24	0	1	1	0	1	1	0.2	0	0.04	0	0.19	1	1	1	0.04	1
008	1	2 10E	0.97	0.98	0.84	0.99	0.27	0.32	1	0	0.04	1	0.04	0.22	0.59	0.96	0.96	1	0.21	0.74	0.96	0.47	0	0	0.74	0	0.94	0.95	0.05	0.82	0.24	0	0.7	1	0	1	1	0.81	0	0.04	0	0.19	1	1	1	0.04	1
008	1	2 11E	0.97	0.98	0.9	0	0.27	0.32	1	0	0.04	1	0.04	0.22	0.59	0.96	0.96	1	0.21	0.74	0.96	0.47	0	0	0.74	0	0.94	0.95	0.05	0.82	0.24	0	0.67	1	0	1	1	0.8	0	0.04	0	0.19	1	1	1	0.04	1
008	1	2 12E	0	0.98	0.59	0.99	0.27	0.32	0.99	0	0.04	1	0.04	0.22	0.59	0.96	0.96	1	0.21	0.74	0.96	0.47	0	0	0.74	0	0.94	0.95	0.05	0.82	0.24	0	0.61	1	0	1	1	0.47	0	0.04	0.91	0.68	1	1	1	0.04	1
008	1	2 2E	0.97	0.98	0.65	0.99	0.27	0.32	1	0	0.31	1	1	0.22	0.59	0.96	0.96	1	0.21	0.74	0.96	0.47	0	0	0.74	0	0.94	0.95	0.05	0.82	0.24	0	1	1	0	1	1	0.79	0	0.3	0.91	1	1	0.82	0.82	0.39	1
008	1	2 3E	0.97	0.47	0.58	0.99	0.27	0.32	1	0	0.31	1	1	0.22	0.59	0.96	0.96	1	0.21	0.74	0.96	0.47	0	0	0.74	0	0.94	0.95	0.05	0.82	0.24	0	0.62	1	0	1	1	0.5	0	0.3	0.91	1	1	1	1	0.39	1
008	1	2 4E	0	0.98	0.65	0	0.27	0.32	1	0	0.04	1	0.04	0.22	0.59	0.96	0.96	0	0.15	0.74	0.96	0.47	0	0	0.74	0	0.94	0.95	0.05	0.82	0.24	0	0.69	1	0	1	1	0.2	0	0.04	0	0.19	1	1	1	0.04	1
008	1	2 5E	0	0.98	0.89	0	0.27	0.32	1	0	0.04	1	0.04	0.22	0.59	0.96	0.96	1	0.21	0.74	0.96	0.47	0	0	0.74	0	0.94	0.95	0.05	0.82	0.24	0	0	1	0	1	1	0.47	0	0.04	0.91	0.68	1	1	1	0.04	1
008	1	2 7E	0.97	0.98	0.87	0	0.27	0.32	1	0	0.04	1	0.04	0.22	0.59	0.96	0.96	1	0.21	0.74	0.96	0.47	0	0	0.74	0	0.94	0.95	0.05	0.82	0.24	0	0	1	0	1	1	0.81	0	0.04	0.91	1	1	1	1	0.04	1
008	1	2 8E	0	0.98	0.49	0	0.27	0.32	1	0	0.04	1	0.04	0.22	0.59	0.96	0.96	1	0.21	0.74	0.96	0.47	0	0	0.74	0	0.94	0.95	0.05	0.82	0.24	0	0.61	1	0	1	1	0.8	0.05	0.04	0.65	1	1	1	1	0.04	1
008	1	2 9E	0	0.98	0.89	0.99	0.27	0.32	1	0	0.04	1	0.04	0.22	0.59	0.96	0.96	0	0.21	0.74	0.96	0.47	0	0	0.74	0	0.94	0.95	0.05	0.82	0.24	0	0	1	0	1	1	0.47	0	0.04	0.65	1	1	1	1	0.04	1
008	2	1 10E	0	0.98	0.89	0	0.27	0.32	1	0	0.04	1	0.04	0.22	0.59	0.96	0.96	0	0.21	0.74	0.96	0.47	0	0	0.74	0	0.94	0.95	0.05	0.82	0.24	0	0.59	1	0	1	1	0.2	0	0.04	0.91	0.68	1	1	1	0.04	1
008	2	1 11E	0	0.98	0.9	0	0.27	0.32	1	0	0.04	1	0.04	0.22	0.59	0.96	0.96	0	0.21	0.74	0.96	0.47	0	0	0.74	0	0.94	0.95	0.05	0.82	0.24	0	0	1	0	1	1	0.45	0	0.04	0	0	1	1	1	0.04	1
008	2	1 1E	0	0.95	0.9	0.99	0.27	0.32	1	0	0.3	1	1	0.22	0.59	0.96	0.96	0	0.21	0.74	0.96	0.47	0	0	0.74	0	0.94	0.95	0.05	0.82	0.24	0	0.46	1	0	1	1	0.47	0	0.3	0.91	1	1	1	1	0.39	1
008	2	1 3E	0	0.98	0.84	0	0.27	0.32	1	0	0.04	1	0.04	0.22	0.59	0.96	0.96	0	0.21	0.74	0.96	0.47	0	0	0.74	0	0.94	0.95	0.05	0.82	0.24	0	1	1	0	1	1	0.2	0	0.04	0.91	0.68	1	1	1	0.04	1
008	2	1 4E	0	0.98	0.64	0	0.27	0.32	1	0	0.04	1	0.04	0.22	0.59	0.96	0.96	0	0.21	0.74	0.96	0.47	0	0	0.74	0	0.94	0.95	0.05	0.82	0.24	0	0	1	0	1	1	0.2	0	0.04	0	0.19	1	1	1	0.04	1
008	2	1 5E	0	0.98	0.65	0	0.27	0.32	1	0	0.04	1	0.04	0.22	0.59	0.96	0.96	0	0.21	0.74	0.96	0.47	0	0	0.74	0	0.94	0.95	0.05	0.82	0.24	0	0	1	0	1	1	0.2	0	0.04	0.65	1	1	0.22	0.22	0.04	1
008	2	1 6E	0	0.98	0.65	0.99	0.27	0.32	1	0	0.04	1	0.04	0.22	0.59	0.96	0.96	0	0.21	0.74	0.96	0.47	0	0	0.74	0	0.94	0.95	0.05	0.82	0.24	0	0.55	1	0	1	1	0.47	0	0.04	0	0.19	1	1	1	0.04	1
008	2	1 7E	0	0.98	0.8	0.99	0.27	0.32	1	0	0.16	1	0.81	0.22	0.59	0.96	0.96	1	0.21	0.74	0.96	0.47	0	0	0.74	0	0.94	0.95	0.05	0.82	0.24	0	0.82	1	0	1	1	0.47	0	0.14	0	0.19	1	1	1	0.27	1
008	2	1 8E	0	0.98	0.89	0	0.27	0.32	1	0	0.04	1	0.04	0.22	0.59	0.96	0.96	0	0.21	0.74	0.96	0.47	0	0	0.74	0	0.94	0.95	0.05	0.82	0.24	0	0.96	1	0	1	1	0.2	0	0.04	0	0.19	1	1	1	0.04	1
008	2	1 9E	0	0.98	0.35	0.99	0.27	0.32	1	0	0.04	1	0.04	0.22	0.59	0.96	0.96	0	0.21	0.74	0.96	0.47	0	0	0.74	0	0.94	0.95	0.05	0.82	0.24	0	0.61	1	0	1	1	0.47	0	0.04	0	0.19	1	1	1	0.04	1
008	2	2 10	0	0.98	0.6	0.99	0.27	0.32	1	0	0.1	1	0.13	0.22	0.59	0.96	0.96	0.25	0.21	0.74	0.96	0.47	0	0	0.74	0	0.94	0.95	0.05	0.82	0.24	0	0	1	0	1	1	0.47	0	0.1	0.91	0.68	1	1	1	0.08	1
008	2	2 11	0	0.98	0.76	0	0.27	0.32	1	0	0.04	1	0.08	0.22	0.59	0.96	0.96	0.4	0.21	0.74	0.96	0.47	0	0	0.74	0	0.94	0.95	0.05	0.82	0.24	0	0.61	1	0	1	1	0.2	0	0.04	0	0.19	1	1	1	0.05	1

008	2	2	2	0	0.98	0.58	0	0.27	0.32	1	0	0.22	1	0.72	0.22	0.59	0.96	0.96	0	0.21	0.74	0.96	0.47	0	0	0.74	0	0.94	0.95	0.05	0.82	0.24	0	0	1	0	1	1	0.81	0	0.27	0	0.19	1	1	1	0.3	1
008	2	2	3	0	0.98	0.42	0	0.27	0.32	1	0	0.04	1	0.04	0.22	0.59	0.96	0.96	0	0.21	0.74	0.96	0.47	0	0	0.74	0	0.94	0.95	0.05	0.82	0.24	0	0	1	0	1	1	0.2	0	0.04	0.91	0.68	1	1	1	0.04	1
008	2	2	4	0	0.98	0.89	0	0.27	0.32	1	0	0.15	1	0.48	0.22	0.59	0.96	0.96	0	0.21	0.74	0.96	0.47	0	0	0.74	0	0.94	0.95	0.05	0.82	0.24	0	0	1	0	1	1	0.6	0	0.06	0.91	0.68	1	1	1	0.19	1
008	2	2	5	0.97	0.98	0.87	0.99	0.27	0.32	1	0	0.04	1	0.04	0.22	0.59	0.96	0.96	0	0.21	0.74	0.96	0.47	0	0	0.74	0	0.94	0.95	0.05	0.82	0.24	0	0	1	0	1	1	0.76	0	0.04	0	0.19	1	1	1	0.04	1
008	2	2	6	0	0.98	0.47	0.99	0.27	0.32	1	0	0.07	1	0.09	0.22	0.59	0.96	0.96	0.9	0.21	0.74	0.96	0.47	0	0	0.74	0	0.94	0.95	0.05	0.82	0.24	0	0	1	0	1	1	0.81	0	0.08	0.91	0.68	1	1	1	0.05	1
008	2	2	7	0.65	0.92	0.87	0.99	0.27	0.32	1	0	0.31	1	1	0.22	0.59	0.96	0.96	0.07	0.21	0.74	0.96	0.47	0	0	0.74	0	0.94	0.95	0.05	0.82	0.24	0	0	1	0	1	1	0.2	0	0.3	0.91	0.97	1	1	1	0.39	1
008	2	2	8	0.97	0.98	0.87	0	0.27	0.32	1	0	0.04	1	0.09	0.22	0.59	0.96	0.96	0.07	0.21	0.74	0.96	0.47	0	0	0.74	0	0.94	0.95	0.05	0.82	0.24	0	0.61	0.09	0	0.37	1	0.81	0	0.06	0	0.19	1	1	1	0.04	1
008	2	2	9	0	0.98	0.58	0.99	0.27	0.32	1	0	0.04	1	0.04	0.22	0.59	0.96	0.96	0	0.21	0.74	0.96	0.47	0	0	0.74	0	0.94	0.95	0.05	0.82	0.24	0	0	1	0	1	1	0.47	0	0.04	0	0.19	1	1	1	0.04	1
008	3	1	10E	0	0.98	0.87	0	0.27	0.32	1	0	0.04	1	0.04	0.22	0.59	0.96	0.96	0	0.21	0.74	0.96	0.47	0	0	0.74	0	0.94	0.95	0.05	0.82	0.24	0	0	1	0	1	1	0.2	0	0.04	0	0.19	1	1	1	0.04	1
008	3	1	11E	0	0.98	0.74	0	0.27	0.32	1	0	0.04	1	0.04	0.22	0.59	0.96	0.96	0	0.21	0.74	0.96	0.47	0	0	0.74	0	0.94	0.95	0.05	0.82	0.24	0	1	1	0	1	1	0.2	0	0.04	0	0.19	1	1	1	0.04	1
008	3	1	12E	0	0.98	0.89	0	0.27	0.32	1	0	0.04	1	0.04	0.22	0.59	0.96	0.96	1	0.21	0.74	0.96	0.47	0	0	0.74	0	0.94	0.95	0.05	0.82	0.24	0	1	1	0	1	1	0.2	0.05	0.04	0	0.19	1	1	1	0.04	1
008	3	1	1E	0	0.98	0.89	0.99	0.27	0.32	1	0	0.04	1	0.04	0.22	0.59	0.96	0.96	0	0.21	0.74	0.96	0.47	0	0	0.74	0	0.94	0.95	0.05	0.82	0.24	0	0	1	0	1	1	0.47	0	0.04	0	0.19	1	1	1	0.04	1
008	3	1	4E	0	0.98	0.9	0.99	0.27	0.32	1	0	0.31	1	1	0.22	0.59	0.96	0.96	0	0.21	0.74	0.96	0.47	0	0	0.74	0	0.94	0.95	0.05	0.82	0.24	0	0	1	0	1	1	0.81	0	0.3	0	0.19	1	1	1	0.39	1
008	3	1	5E	0	0.98	0.89	0	0.27	0.32	1	0	0.04	1	0.04	0.22	0.59	0.96	0.96	0	0.21	0.74	0.96	0.47	0	0	0.74	0	0.26	0.95	0.05	0.82	0.24	0	0	1	0	1	1	0.2	0	0.04	0	0.19	1	1	1	0.04	1
008	3	1	6E	0	0.98	0.89	0.99	0.27	0.32	1	0	0.04	1	0.04	0.22	0.59	0.96	0.96	0	0.21	0.74	0.96	0.47	0	0	0.74	0	0.94	0.95	0.05	0.82	0.24	0	0.61	1	0	1	1	0.47	0	0.04	0	0.19	1	1	1	0.04	1
008	3	1	7E	0	0.98	0.73	0	0.27	0.32	1	0	0.04	1	0.04	0.22	0.59	0.96	0.96	0	0.21	0.74	0.96	0.47	0	0	0.74	0	0.94	0.95	0.05	0.82	0.24	0	1	1	0	1	1	0.2	0	0.04	0.91	0.68	1	0.97	0.97	0.04	1
008	3	1	8E	0	0.98	0.63	0	0.27	0.32	1	0	0.04	1	0.04	0.22	0.59	0.96	0.96	1	0.21	0.74	0.96	0.47	0	0	0.74	0	0.94	0.95	0.05	0.82	0.24	0	0.41	1	0	1	1	0.2	0	0.04	0	0.19	1	1	1	0.04	1
008	3	1	9E	0	0.98	0.48	0	0.27	0.32	1	0	0.04	1	0.04	0.22	0.59	0.96	0.96	0	0.21	0.74	0.96	0.47	0	0	0.74	0	0.94	0.95	0.05	0.82	0.24	0	0.82	1	0	1	1	0.2	0	0.04	0	0.19	1	1	1	0.04	1
008	3	2	10E	0	0.98	0.59	0.99	0.27	0.32	1	0	0.04	1	0.04	0.22	0.59	0.96	0.96	0	0.21	0.74	0.96	0.47	0	0	0.74	0	0.94	0.95	0.05	0.82	0.24	0	0	1	0	1	1	0.47	0	0.04	0	0.19	1	1	1	0.04	1
008	3	2	11E	0	0.98	0.89	0.99	0.27	0.32	1	0	0.04	1	0.04	0.22	0.59	0.96	0.96	1	0.21	0.74	0.96	0.47	0	0	0.74	0	0.94	0.95	0.05	0.82	0.24	0	0	1	0	1	1	0.47	0	0.04	0	0.19	1	1	1	0.04	1
008	3	2	12E	0	0.98	0.59	0	0.27	0.32	1	0	0.31	1	1	0.22	0.59	0.96	0.96	0	0.21	0.74	0.96	0.47	0	0	0.74	0	0.94	0.95	0.05	0.82	0.24	0	0.61	1	0	1	1	0.2	0	0.3	0	0.19	1	1	1	0.39	1
008	3	2	3E	0	0.98	0.9	0	0.27	0.32	1	0	0.31	1	1	0.22	0.59	0.96	0.96	0	0.21	0.74	0.96	0.47	0	0	0.74	0	0.94	0.95	0.05	0.82	0.24	0	0	1	0	1	1	0.2	0	0.3	0	0	1	1	1	0.39	1
008	3	2	4E	0	0.98	0.89	0	0.27	0.32	1	0	0.04	1	0.04	0.22	0.59	0.96	0.96	0	0.21	0.74	0.96	0.47	0	0	0.74	0	0.94	0.95	0.05	0.82	0.24	0	0.82	1	0	1	1	0.2	0	0.04	0.65	1	1	1	1	0.04	1
008	3	2	5E	0.97	0.98	0.9	0	0.27	0.32	1	0	0.31	1	1	0.22	0.59	0.96	0.96	0	0.21	0.74	0.15	0.47	0	0	0.74	0	0.57	0.95	0.05	0.82	0.24	0	1	1	0	1	1	0.47	0	0.3	0	0	1	1	1	0.39	1
008	3	2	6E	0	0.98	0.89	0	0.27	0.32	1	0	0.04	1	0.04	0.22	0.59	0.96	0.96	0	0.21	0.74	0.96	0.47	0	0	0.74	0	0.94	0.95	0.05	0.82	0.24	0	0	1	0	1	1	0.47	0	0.04	0	0	1	1	1	0.04	1
008	3	2	7E	0	0.98	0.84	0	0.27	0.32	1	0	0.04	1	0.04	0.22	0.59	0.96	0.96	0	0.21	0.74	0.96	0.47	0	0	0.74	0	0.94	0.95	0.05	0.82	0.24	0	1	1	0	1	1	0.2	0.05	0.04	0	0.19	1	1	1	0.04	1
008	3	2	8E	0.97	0.98	0.89	0	0.27	0.32	1	0	0.04	1	0.04	0.22	0.59	0.96	0.96	0	0.21	0.74	0.96	0.47	0	0	0.74	0	0.94	0.95	0.05	0.82	0.24	0	0.4	1	0	1	1	0.47	0	0.04	0.65	1	1	1	1	0.04	1
008	3	2	9E	0.96	0.98	0.55	0.99	0.27	0.32	1	0	0.04	1	0.04	0.22	0.59	0.96	0.96	0	0.21	0.74	0.96	0.47	0	0	0.74	0	0.94	0.95	0.05	0.82	0.24	0	0	1	0	1	1	0.81	0	0.04	0	0.19	1	1	1	0.04	1
008	4	2	10	0	0.33	0.88	0.1	0.38	0.97	0.35	0	0.43	0.92	0.3	0.11	0.6	0.83	0.06	0	0.96	0.28	0.04	0	0	0	0	0	0	0	0	0	0	0	0	1	0	1	0.03	0.29	0	0.99	0.19	0.19	0	0.15	0.15	0.37	0.88
008	4	2	1	0	0.33	0.88	0.1	0.38	0.97	0.35	0	0.43	0.92	0.3	0.11	0.6	0.83	0.06	0	0.96	0.28	0.04	0	0	0	0	0	0	0	0	0	0	0	0	1	0	1	0.03	0.29	0	0.99	0.19	0.19	0	0.15	0.15	0.37	0.88
008	4	2	2	0	0.33	0.88	0.1	0.38	0.97	0.35	0	0.43	0.92	0.3	0.11	0.6	0.84	0.06	0	0.96	0.28	0.04	0	0	0	0	0	0	0	0	0	0	0	0	1	0	1	0.03	0.29	0	0.99	0.19	0.19	0	0.15	0.15	0.37	0.88
008	4	2	3	0	0.33	0.88	0.1	0.38	0.97	0.35	0	0.43	0.12	0.3	0.11	0.04	0.83	0.06	0	0.96	0.28	0.04	0	0	0	0	0	0	0	0	0	0	0	0.48	1	0	1	0.03	0.29	0	0.99	0.19	0	0	0.15	0.15	0.37	0.88
008	4	2	4	0	0	0.88	0.1	0.38	0.97	0.35	0	0.43	0.92	0.3	0.11	0.6	0.84	0.06	0	0.96	0.28	0.04	0	0	0	0	0	0	0	0	0	0	0	0	1	0	1	0.03	0.29	0	0.99	0.19	0.19	0	0.15	0.15	0.37	0.88
008	4	2	5	0	0.33	0.88	0.1	0.38	0.97	0.35	0	0.43	0.92	0.3	0.11	0.6	0.83	0.06	0	0.96	0.28	0.04	0	0	0	0	0	0	0	0	0	0	0	0	1	0	1	0.03	0.29	0	0.99	0.19	0.19	0	0.15	0.15	0.37	0.88
008	4	2	6	0	0.33	0.88	0.1	0.38	0.97	0.35	0	0.43	0.92	0.3	0.11	0.6	0.84	0.06	0	0.96	0.28	0.04	0	0	0	0	0	0	0	0	0	0	0	0	1	0	1	0.03	0.29	0	0.99	0.19	0.19	0	0.15	0.15	0.37	0.88
008	4	2	7	0	0.33	0.88	0.1	0.38	0.97	0.35	0	0.43	0.92	0.3	0.11	0.6	0.83	0.06	0	0.96	0.28	0.04	0	0	0	0	0	0	0	0	0	0	0	0	1	0	1	0.03	0.29	0	0.99	0.19	0.19	0	0.15	0.15	0.37	0.88
008	4	2	8	0	0.33	0.88	0.1	0.38	0.97	0.35	0	0.43	0.92	0.3	0.11	0.6	0.84	0.06	0	0.96	0.28	0.04	0	0	0	0	0	0	0	0	0	0	0	0	0.71	0	1	0.03	0.29	0	0.99	0.19	0.19	0	0.15	0.15	0.37	0.88
008	4	2	9	0	0.33	0.88	0.1	0.38	0.97	0.35	0	0.43	0.92	0.3	0.11	0.6	0.84	0.06	0	0.96	0.28	0.04	0	0	0	0	0	0	0	0	0	0	0	0	1	0	1	0.03	0.29	0	0.99	0.19	0.19	0	0.15	0.15	0.37	0.88
008	5	1	10	0	0.33	0.88	0.1	0.38	0.97	0.35	0	0.43	0.92	0.3	0.11	0.6	0.84	0.06	0	0.96	0.28	0.04	0	0	0	0	0	0	0	0	0	0	0	0	1	0	1	0.03	0.29	0	0.99	0.19	0.19	0	0.15	0.15	0.37	0.88
008	5	1	1	0	0.33	0.88	0.1	0.38	0.97	0.35	0	0.43	0.92	0.3	0.11	0.6	0.83	0.06	0	0.96	0.28	0.04	0	0	0	0	0	0	0	0	0	0	0	0	1	0	0.78	0.03	0.29	0	0.99	0.19	0.19	0	0.15	0.15	0.37	0.88
008	5	1	2	0	0.33	0.88	0.1	0.38	0.97	0.35	0	0.43	0.92	0.3	0.11	0.6	0.84	0.06	0	0.96	0.28	0.04	0	0	0	0	0	0	0	0	0	0	0	0	1	0	1	0.03	0.29	0	0.99	0.19	0.19	0	0.15	0.15	0.37	0.88
008	5	1	3	0	0.33	0.88	0.1	0.38	0.97	0.35	0	0.43	0.92	0.29	0.11	0.6	0.84	0.06	0	0.96	0.28	0.04	0	0	0	0	0	0	0	0	0	0	0	0	1	0	1	0.03	0.29	0	0.99	0.19	0.19	0	0.15	0.15	0.37	0.88
008	5	1	4	0	0.33	0.88	0.1	0.38	0.97	0.35	0	0.43	0.92	0.3	0.11	0.6	0.84	0.06	0	0.96	0.28	0.04	0	0	0	0	0	0	0	0	0	0	0	0	1	0	1	0.03	0.29	0	0.99	0.19	0.19	0	0.15	0.15	0.37	0.88
008	5	1	5	0	0.33	0.88	0.1	0.38	0.97	0.35	0	0.43	0.92	0.3	0.11	0.6	0.83	0.06	0	0.96	0.28	0.04	0	0	0	0	0	0	0	0	0	0	0	0	1	0	1	0.03	0.29	0	0.99	0.19	0	0	0.15	0.15	0.37	0.88
008	5	1	6	0	0.33	0.88	0.1	0.38	0.97	0.35	0	0.43	0.92	0.3	0.11	0.6	0.84	0.06	0	0.96	0.28	0.04	0	0	0	0	0	0	0	0	0	0	0	0	1	0	1	0.03	0.29	0	0.99	0.19	0.19	0	0.15	0.15	0.37	0.88
008	5	1	7	0	0.33	0.88	0.1	0.38	0.97	0.35	0	0.43	0.92	0.3	0.11	0.6	0.84	0.06	0	0.96	0.28	0.04	0	0	0	0	0	0	0	0	0	0	0	0	1	0	1	0.03	0.29	0	0.99	0.19	0.19	0	0.15	0.15	0.37	0.88
008	5	1	8	0	0.33	0.88	0.1	0.38	0.97	0.35	0	0.43	0.92	0.3	0.11	0.6	0.84	0.06	0	0.96	0.28	0.04	0	0	0	0	0	0	0	0	0	0	0	0	1	0	1	0.03	0.29	0	0.99	0.19	0.19	0	0.15	0.15	0.37	0.88
008	5	1	9	0	0.33	0.88	0.1	0.38	0.97	0.35	0	0.43	0.92	0.3	0.11	0.6	0.84	0.06	0	0.96	0.28	0.04	0	0	0	0	0	0	0	0	0	0	0	0	1	0	1	0.03	0.29	0	0.99	0.19	0.19	0	0.15	0.15	0.37	0.88
008	5	2	10	0	0.33	0.88	0.1	0.38	0.97	0.35	0	0.43	0.92	0.3	0.11	0.6	0.84	0.06	0	0.96	0.28	0.04	0	0	0	0	0	0	0	0	0	0	0	0	1	0	1	0.03	0.29	0	0.99	0.19	0.19	0	0.15	0.15	0.37	0.88
008	5	2	1	0	0	0.88	0.11	0.38	0.97	0.35	0	0.43	0.92	0.3	0.11	0.6	0.84	0.06	0	0.96	0.28	0.04	0	0	0	0	0	0	0	0	0	0	0	0	1	0	1	0.03	0.29	0	0.99	0.19	0.19	0	0.15	0.15	0.37	0.88
008	5	2	2	0	0.33	0.88	0.1	0.38	0.97	0.35	0	0.43	0.92	0.3	0.11	0.6	0.83	0.06	0	0.96	0.28	0.04	0	0	0	0	0	0	0	0	0	0	0	0	1	0	1	0.03	0.29	0	0.99	0.19	0.19	0	0.15	0.15	0.37	0.88
008	5	2	3	0	0.33	0.88	0.1	0.38	0.97	0.35	0	0.43	0.92	0.3	0.11	0.6	0.83	0.06	0	0.96	0.28	0.04	0	0	0	0	0	0	0	0	0	0	0	0	1	0	1	0.03	0.29	0	0.99	0.19	0.19	0	0.15	0.15	0.37	0.88
008	5	2	4	0	0.33	0.88	0.1	0.38	0.97	0.35	0	0.43	0.92	0.3	0.11	0.6	0.83	0.06	0	0.96	0.28	0.04	0	0	0	0	0	0	0	0	0	0	0	0	1	0	1	0.03	0.29	0	0.99	0.19	0.19	0	0.15	0.15	0.37	0.88
008	5	2	5	0	0.33	0.88	0.1	0.38	0.97	0.35	0	0.43	0.92	0.3	0.11	0.6	0.84	0.06	0	0.96	0.28	0.04	0	0	0	0	0	0	0	0	0	0	0	0	1	0	1	0.03	0.29	0	0.99	0.19	0.19	0	0.15	0.15	0.37	0.88
008	5	2	6	0	0.33	0.88	0.1	0.38	0.97	0.35	0	0.43	0.92	0.3	0.11	0.6	0.84	0.06	0	0.96	0.28	0.04	0	0	0	0	0	0	0	0	0	0	0	0	1	0	1	0.03	0.29	0	0.99	0.19	0.19	0	0.15	0.15	0.37	0.88
008	5	2	7	0	0.33	0.88	0.1	0.38	0.97	0.35	0	0.43	0.92	0.3	0.11	0.6	0.83	0.06	0	0.96	0.28	0.04	0	0	0	0	0	0	0	0	0	0	0	0	1	0	1	0.03	0.29	0	0.99	0.19	0.19	0	0.15	0.15	0.37	0.88
008	5	2	8	0	0.33	0.88	0.1	0.38	0.97	0.35	0	0.43	0.92	0.3	0.11	0.6	0.84	0.06	0	0.96	0.28	0.04	0	0	0	0	0	0	0	0	0	0	0	0	1	0	1	0.03	0.29	0	0.99	0.19	0.19	0	0.15	0.15	0.37	0.88
008	5	2	9	0	0.33	0.88	0.1	0.38	0.97	0.35	0	0.43	0.92	0.3	0.11	0.6	0.84	0.06	0	0.96	0.28	0.04	0	0	0	0	0	0	0	0	0	0	0	0	1	0	1	0.03	0.29	0	0.99	0.19	0.19	0	0.15	0.15	0.37	0.88
008	6	2	10	0	0.33	0.88	0.1	0.38	0.97	0.35	0	0.43	0.92	0.3	0.11	0.6	0.84	0.06	0	0.96	0.28	0.04	0	0	0	0	0	0	0	0	0	0	0	0	1	0	1	0.03	0.29	0	0.99	0.19	0.19	0	0.15	0.15	0.37	0.88
008	6	2	11	0	0.33	0.88	0.1	0.38	0.97	0.35	0	0.43	0.92	0.3	0.11	0.6	0.84	0.06	0	0.96	0.28	0.04	0	0	0	0	0	0	0	0	0	0	0	0	1	0	1	0.03	0.29	0	0.99	0.19	0.19	0	0.15	0.15	0.37	0.88
008	6	2	2	0	0.33	0.88	0.1	0.38	0.97	0.35	0	0.43	0.92	0.3	0.11	0.6	0.84	0.06	0	0.96	0.28	0.04	0	0	0	0	0	0	0	0	0	0	0	0	1	0	1	0.03	0.29	0	0.99	0.19	0.19	0	0.15	0.15	0.37	0.88
008	6	2	3	0	0.33	0.88	0.1	0.38	0.97	0.35	0	0.43	0.92	0.3	0.11	0.6	0.84	0.06	0	0.96	0.28	0.04	0	0	0	0	0	0	0	0	0	0	0	0	1	0	1	0.03	0.29	0	0.99	0.19	0.19	0	0.15	0.15	0.37	0.88
008	6	2	4	0	0.33	0.88	0.1	0.38	0.97	0.35	0	0.43	0.92	0.3	0.11	0.6	0.84	0.06	0	0.96	0.28	0.04	0	0	0	0	0	0	0	0	0	0	0	0	1	0	1	0.03	0.29	0	0.99	0.19	0.19	0	0.15	0.15	0.37	0.88
008	6	2	5	0	0	0.88	0.1	0.38	0.97	0.35	0	0.43	0.92	0.3	0.11	0.6	0.84	0.06	0	0.96	0.28	0.04	0	0	0	0	0	0	0	0	0	0	0	0	1	0	1	0.03	0.29	0	0.99	0.19	0	0	0.15	0.15	0.37	0.88
008	6	2	6	0	0.33	0.88	0.1	0.38	0.97	0.35	0	0.43	0.92	0.3	0.11	0.6	0.84	0.06	0	0.96	0.28	0.04	0	0	0	0	0	0	0	0	0	0	0	0	1	0	1	0.03	0.29	0	0.99	0.19	0.19	0	0.15	0.15	0.37	0.88
008	6	2	7	0	0.33	0.88	0.1	0.38	0.97	0.35	0	0.43	0.92	0.3	0.11	0.6	0.83	0.06	0	0.96	0.28	0.04	0	0	0	0	0	0	0	0	0	0	0	0	1	0	1	0.03	0.29	0	0.99	0.19	0.19	0	0.15	0.15	0.37	0.88
008	6	2	8	0	0	0.88	0.1	0.38	0.97	0.35	0	0.43	0.92	0.3	0.11	0.6	0.84	0.06	0	0.96	0.28	0.04	0	0	0	0	0	0	0	0	0	0	0	0	1	0	1	0.03	0.29	0	0.99	0.19	0	0	0.15	0.15	0.37	0.88
008	6	2	9	0	0.33	0.88	0.1	0.38	0.97	0.35	0	0.43	0.92	0.3	0.11	0.6	0.83	0.06	0	0.96	0.28	0.04	0	0	0	0	0	0	0	0	0	0	0	0	1	0	1	0.03	0.29	0	0.99	0.19	0.19	0	0.15	0.15	0.37	0.88
008	8	1	10	0	0.33	0.88	0.1	0.38	0.97	0.35	0	0.43	0.92	0.29	0.11	0.6	0.84	0.06	0	0.21	0.28	0.04	0	0	0	0	0	0	0	0	0	0	0	0	1	0	1	0.03	0.29	0	0.49	0.19	0.19	0	0.14	0.14	0.37	0.88
008	8	1	2	0	0.33	0.88	0.1	0.38	0.97	0.35	0	0.43	0.92	0.29	0.11	0.6	0.84	0.06	0	0.21	0.28	0.04	0	0	0	0	0	0	0	0	0	0	0	0	1	0	1	0.03	0.29	0	0.49	0.19	0.19	0	0.14	0.14	0.37	0.88

008	8	1 5	0	0	0.87	0.1	0.38	0.97	0.35	0	0.43	0.92	0.3	0.11	0.6	0.83	0.06	0	0.96	0.28	0.04	0	0	0	0	0	0	0	0	0	0	0	0	1	0	1	0.03	0.29	0	0.99	0.19	0	0	0.15	0.15	0.37	0.88
008	8	1 6	0	0.33	0.88	0.1	0.38	0.97	0.35	0	0.43	0.92	0.29	0.11	0.6	0.84	0.06	0	0.21	0.28	0.04	0	0	0	0	0	0	0	0	0	0	0	0	1	0	1	0.03	0.29	0	0.49	0.19	0.19	0	0.14	0.14	0.37	0.88
008	8	1 9	0	0.33	0.88	0.1	0.38	0.97	0.35	0	0.43	0.92	0.29	0.11	0.6	0.83	0.06	0	0.21	0.28	0.04	0	0	0	0	0	0	0	0	0	0	0	0	1	0	1	0.03	0.29	0	0.49	0.19	0.19	0	0.14	0.14	0.37	0.88
008	8	2 10	0	0.33	0.88	0.1	0.38	0.97	0.35	0	0.43	0.92	0.29	0.11	0.6	0.84	0.06	0	0.21	0.28	0.04	0	0	0	0	0	0	0	0	0	0	0	0	1	0	1	0.03	0.29	0	0.49	0.19	0.19	0	0.14	0.14	0.37	0.88
008	8	2 1	0	0	0.88	0.1	0.38	0.97	0.35	0	0.43	0.92	0.29	0.11	0.6	0.84	0.06	0	0.21	0.28	0.04	0	0	0	0	0	0	0	0	0	0	0	0	1	0	1	0.03	0.29	0	0.49	0.19	0	0	0.14	0.14	0.37	0.88
008	8	2 2	0	0.98	0.88	0.1	0.38	0.97	0.35	0	0.43	0.92	0.29	0.11	0.6	0.83	0.06	0	0.21	0.28	0.04	0	0	0	0	0	0	0	0	0	0	0	0.82	1	0	1	0.03	0.35	0	0.49	0.19	0.19	0	0.14	0.14	0.37	0.88
008	8	2 3	0	0.33	0.88	0.1	0.38	0.97	0.35	0	0.43	0.92	0.29	0.11	0.6	0.84	0.06	0	0.21	0.28	0.04	0	0	0	0	0	0	0	0	0	0	0	0	1	0	1	0.03	0.29	0	0.49	0.19	0.19	0	0.14	0.14	0.37	0.88
008	8	2 6	0	0.33	0.88	0.1	0.38	0.97	0.35	0	0.43	0.92	0.29	0.11	0.6	0.84	0.06	0	0.21	0.28	0.04	0	0	0	0	0	0	0	0	0	0	0	0	1	0	1	0.03	0.29	0	0.49	0.19	0.19	0	0.14	0.14	0.37	0.88
008	14	1 1	0	0.33	0.88	0.1	0.38	0.97	0.35	0	0.43	0.92	0.3	0.11	0.6	0.84	0.06	0	0.96	0.28	0.04	0	0	0	0	0	0	0	0	0	0	0	0	1	0	1	0.03	0.29	0	0.99	0.19	0.19	0	0.15	0.15	0.37	0.88
008	14	1 3	0	0.33	0.88	0.1	0.38	0.97	0.35	0	0.43	0.92	0.3	0.11	0.6	0.84	0.06	0	0.96	0.28	0.04	0	0	0	0	0	0	0	0	0	0	0	0	1	0	1	0.03	0.29	0	0.99	0.19	0.19	0	0.15	0.15	0.37	0.88
008	14	1 4	0	0.33	0.88	0.1	0.38	0.97	0.35	0	0.43	0.92	0.3	0.11	0.6	0.83	0.06	0	0.96	0.28	0.04	0	0	0	0	0	0	0	0	0	0	0	0	1	0	1	0.03	0.29	0	0.99	0.19	0.19	0	0.15	0.15	0.37	0.88
008	14	1 8	0	0.33	0.88	0.1	0.38	0.97	0.35	0	0.43	0.92	0.3	0.11	0.6	0.83	0.06	0	0.96	0.28	0.04	0	0	0	0	0	0	0	0	0	0	0	0	1	0	1	0.03	0.29	0	0.99	0.19	0.19	0	0.15	0.15	0.37	0.88
008	14	1 9	0	0.33	0.88	0.1	0.38	0.97	0.35	0	0.43	0.92	0.3	0.11	0.6	0.84	0.06	0	0.96	0.28	0.04	0	0	0	0	0	0	0	0	0	0	0	0	1	0	1	0	0.29	0	0.99	0.19	0.19	0	0.15	0.15	0.37	0.88
008	21	1 3	0	0	0.88	0.1	0.38	0.97	0.35	0	0.43	0.92	0.3	0.11	0.6	0.84	0.06	0	0.96	0.28	0.04	0	0	0	0	0	0	0	0	0	0	0	0	1	0	1	0.03	0.29	0	0.99	0.19	0	0	0.15	0.15	0.37	0.88
008	21	1 4	0	0	0.88	0.1	0.38	0.97	0.35	0	0.43	0.92	0.3	0.11	0.6	0.83	0.06	0	0.96	0.28	0.04	0	0	0	0	0	0	0	0	0	0	0	0	1	0	1	0.03	0.29	0	0.99	0.19	0	0	0.15	0.15	0.37	0.88
008	21	1 5	0	0.33	0.88	0.1	0.38	0.97	0.35	0	0.43	0.92	0.3	0.11	0.6	0.84	0.06	0	0.96	0.28	0.04	0	0	0	0	0	0	0	0	0	0	0	0	1	0	1	0.03	0.29	0	0.99	0.19	0.19	0	0.15	0.15	0.37	0.88
008	21	1 8	0	0.33	0.88	0.1	0.38	0.97	0.35	0	0.43	0.92	0.3	0.11	0.6	0.84	0.06	0	0.96	0.28	0.04	0	0	0	0	0	0	0	0	0	0	0	0	1	0	1	0.03	0.29	0	0.99	0.19	0.19	0	0.15	0.15	0.37	0.88
008	21	1 9	0	0	0.88	0.1	0.38	0.97	0.35	0	0.43	0.92	0.3	0.11	0.6	0.84	0.06	0	0.96	0.28	0.04	0	0	0	0	0	0	0	0	0	0	0	0	1	0	1	0.03	0.29	0	0.99	0.19	0	0	0.15	0.15	0.37	0.88
008	28	1 1	0	0	0.1	0.3	0.99	0.4	0.54	0.93	0.04	1	0.04	0.21	0.61	0.96	0.98	0	0.21	0.65	0.53	0.47	0.58	0.59	0.68	0	0.82	0.54	0.03	0	0.09	0	0	0.08	1	0	0.03	0.2	0	0.04	0	0.19	0	0.21	0.21	0.03	0.94
008	28	1 3	0	0	0.1	0.3	0.99	0.4	0.54	0.93	0.04	1	0.04	0.21	0.61	0.96	0.98	0	0.21	0.65	0.53	0.47	0.58	0.59	0.68	0	0.82	0.54	0.03	0	0.09	0	0	0.08	1	0	0.03	0.2	0	0.04	0	0.19	0	0.21	0.21	0.03	0.94
008	28	1 5	0	0	0.1	0.3	0.99	0.4	0.54	0.93	0.04	1	0.04	0.21	0.61	0.96	0.98	0	0.21	0.65	0.53	0.47	0.58	0.59	0.68	0	0.82	0.54	0.03	0	0.09	0	0	0.08	1	0	0.03	0.2	0	0.04	0	0.19	0	0.21	0.21	0.03	0.94
008	28	1 7	0	0	0.1	0.3	0.99	0.4	0.54	0.93	0.04	1	0.04	0.21	0.61	0.96	0.98	0	0.21	0.65	0.53	0.47	0.58	0.59	0.68	0	0.82	0.54	0.03	0	0.09	0	0	0.08	1	0	0.03	0.2	0	0.04	0	0.19	0	0.21	0.21	0.03	0.94
008	28	1 8	0	0	0.1	0.3	0.99	0.4	0.54	0.93	0.04	1	0.04	0.21	0.61	0.96	0.98	0	0.21	0.65	0.53	0.47	0.58	0.59	0.68	0	0.82	0.54	0.03	0	0.09	0	0	0.08	1	0	0.03	0.2	0	0.04	0	0.19	0	0.21	0.21	0.03	0.94
009	0	1 10E	0	0.77	0.89	0	0.27	0.32	1	0	0.31	1	1	0.22	0.59	0.96	0.96	1	0.21	0.74	0.96	0.47	0	0	0.74	0	0.94	0.95	0.05	0.82	0.24	0	0	1	0	1	1	0.47	0	0.3	0	0	1	1	1	0.39	1
009	0	1 1E	0	0.98	0.37	0.99	0.27	0.32	1	0	0.04	1	0.04	0.22	0.59	0.96	0.96	0.42	0.21	0.74	0.96	0.47	0	0	0.74	0	0.94	0.95	0.05	0.82	0.24	0	0.61	1	0	1	1	0.43	0	0.04	0.65	1	1	1	1	0.04	1
009	0	1 2E	0	0.98	0.9	0	0.27	0.32	1	0	0.04	1	0.04	0.22	0.59	0.96	0.96	0.45	0.21	0.74	0.96	0.47	0	0	0.74	0	0.94	0.95	0.05	0.82	0.24	0	0	1	0	1	1	0.47	0	0.04	0	0.19	1	1	1	0.04	1
009	0	1 3E	0	0.98	0.87	0	0.27	0.32	1	0	0.04	1	0.04	0.22	0.59	0.96	0.96	0	0.21	0.74	0.96	0.47	0	0	0.74	0	0.94	0.95	0.05	0.82	0.24	0	0	1	0	1	1	0.2	0	0.04	0.91	0.68	1	1	1	0.04	1
009	0	1 4E	0	0.98	0.89	0	0.27	0.32	1	0	0.04	1	0.04	0.22	0.59	0.96	0.96	0	0.21	0.74	0.96	0.47	0	0	0.74	0	0.94	0.95	0.05	0.82	0.24	0	0.58	1	0	1	1	0.6	0	0.04	0	0.19	1	1	1	0.04	1
009	0	1 5E	0	0.98	0.9	0	0.27	0.32	1	0	0.04	1	0.04	0.22	0.59	0.96	0.96	0	0.21	0.74	0.96	0.47	0	0	0.74	0	0.94	0.95	0.05	0.82	0.24	0	0	1	0	1	1	0.2	0	0.04	0.91	1	1	1	1	0.04	1
009	0	1 6E	0	0.98	0.87	0.99	0.27	0.32	1	0	0.21	1	0.49	0.22	0.59	0.96	0.96	0.94	0.21	0.74	0.96	0.47	0	0	0.74	0	0.94	0.95	0.05	0.82	0.24	0	0	1	0	1	1	0.47	0	0.26	0.91	0.68	1	1	1	0.2	1
009	0	1 7E	0	0.98	0.83	0	0.27	0.32	1	0	0.04	1	0.04	0.22	0.59	0.96	0.96	1	0.21	0.74	0.96	0.47	0	0	0.74	0	0.94	0.95	0.05	0.82	0.24	0	0.46	1	0	1	1	0.2	0	0.04	0	0.19	1	1	1	0.04	1
009	0	1 8E	0	0.98	0.9	0	0.27	0.32	1	0	0.04	1	0.04	0.22	0.59	0.96	0.96	1	0.21	0.74	0.96	0.47	0	0	0.74	0	0.94	0.95	0.05	0.82	0.24	0	0	1	0	1	1	0.2	0	0.04	0.91	0.68	1	1	1	0.04	1
009	0	1 9E	0.97	0.98	0.88	0.99	0.27	0.32	1	0	0.04	1	0.04	0.22	0.59	0.96	0.96	0	0.21	0.74	0.96	0.47	0	0	0.74	0	0.94	0.95	0.05	0.82	0.24	0	0	1	0	1	1	0.81	0	0.04	0.65	1	1	1	1	0.04	1
009	1	1 10E	0	0.98	0.62	0.98	0.27	0.32	1	0	0.04	1	0.04	0.22	0.59	0.96	0.96	1	0.21	0.74	0.96	0.47	0	0	0.74	0	0.94	0.95	0.05	0.82	0.24	0	0.61	1	0	1	1	0.2	0	0.04	0	0.19	1	1	1	0.04	1
009	1	1 11E	0.97	0.98	0.89	0.99	0.27	0.32	1	0	0.04	1	0.04	0.22	0.59	0.96	0.96	0	0.21	0.74	0.96	0.47	0	0	0.74	0	0.94	0.95	0.05	0.82	0.24	0	0.82	1	0	1	1	0.81	0	0.04	0	0.19	1	1	1	0.04	1
009	1	1 12E	0	0.98	0.88	0	0.27	0.32	1	0	0.04	1	0.04	0.22	0.59	0.96	0.96	0	0.21	0.74	0.96	0.47	0	0	0.74	0	0.94	0.95	0.05	0.82	0.24	0	0	1	0	1	1	0.2	0	0.04	0.91	0.68	1	1	1	0.04	1
009	1	1 2E	0	0.98	0.84	0	0.27	0.32	1	0	0.04	1	0.04	0.22	0.59	0.96	0.96	0	0.21	0.74	0.96	0.47	0	0	0.74	0	0.94	0.95	0.05	0.82	0.24	0	0	1	0	1	1	0.2	0	0.04	0	0.19	1	1	1	0.04	1
009	1	1 3E	0	0.98	0.89	0	0.27	0.32	1	0	0.04	1	0.04	0.22	0.59	0.96	0.96	0	0.21	0.74	0.96	0.47	0	0	0.74	0	0.94	0.95	0.05	0.82	0.24	0	0	1	0	1	1	0.2	0	0.04	0	0.19	1	1	1	0.04	1
009	1	1 4E	0	0.98	0.63	0.99	0.27	0.32	1	0	0.04	1	0.04	0.22	0.59	0.96	0.92	0	0.21	0.74	0.96	0.47	0	0	0.74	0	0.94	0.95	0.05	0.82	0.24	0	1	1	0	1	1	0.47	0	0.04	0	0.19	1	1	1	0.04	1
009	1	1 5E	0	0.98	0.82	0	0.27	0.32	1	0	0.04	1	0.04	0.22	0.59	0.96	0.96	0	0.21	0.74	0.96	0.47	0	0	0.74	0	0.94	0.95	0.05	0.82	0.24	0	1	1	0	1	1	0.2	0	0.04	0	0.19	1	1	1	0.04	1
009	1	1 6E	0	0.98	0.89	0.99	0.27	0.32	1	0	0.04	1	0.04	0.22	0.59	0.96	0.96	0	0.21	0.74	0.96	0.47	0	0	0.74	0	0.94	0.95	0.05	0.82	0.24	0	0.99	1	0	1	1	0.47	0	0.04	0	0.19	1	1	1	0.04	1
009	1	1 7E	0.97	0.98	0.62	0.99	0.27	0.32	1	0	0.31	1	1	0.22	0.59	0.96	0.96	0	0.21	0.74	0.96	0.47	0	0	0.74	0	0.94	0.95	0.05	0.82	0.24	0	0	1	0	1	1	0.81	0	0.3	0.91	0.68	1	1	1	0.39	1
009	1	1 9E	0	0.98	0.85	0	0.27	0.32	1	0	0.04	1	0.04	0.22	0.59	0.96	0.96	0	0.21	0.74	0.96	0.47	0	0	0.74	0	0.94	0.95	0.05	0.82	0.24	0	0.61	1	0	1	1	0.81	0	0.04	0	0	1	1	1	0.04	1
009	2	1 10E	0	0.98	0.85	0	0.27	0.32	1	0	0.04	1	0.04	0.22	0.59	0.96	0.96	0	0.21	0.74	0.96	0.47	0	0	0.74	0	0.94	0.95	0.05	0.82	0.24	0	0.82	1	0	1	0.03	0.2	0	0.04	0	0.19	0	1	1	0.04	1
009	2	1 1E	0	0.98	0.89	0	0.27	0.32	1	0	0.04	1	0.04	0.22	0.59	0.96	0.96	0	0.21	0.74	0.96	0.47	0	0	0.74	0	0.94	0.95	0.05	0.82	0.24	0	0	1	0	1	1	0.8	0	0.04	0.65	1	1	1	1	0.04	1
009	2	1 2E	0.97	0.98	0.87	0	0.27	0.32	1	0	0.04	1	0.04	0.22	0.59	0.96	0.96	0	0.21	0.74	0.96	0.47	0	0	0.74	0	0.94	0.95	0.05	0.82	0.24	0	0	1	0	1	1	0.81	0	0.04	0	0.19	1	1	1	0.04	1
009	2	1 3E	0	0.98	0.89	0.99	0.27	0.32	1	0	0.04	1	0.04	0.22	0.59	0.96	0.96	0	0.21	0.74	0.96	0.47	0	0	0.74	0	0.94	0.95	0.05	0.82	0.24	0	0.82	1	0	1	1	0.47	0	0.04	0	0	1	1	1	0.04	1
009	2	1 4E	0.97	0.98	0.89	0	0.27	0.32	1	0	0.04	1	0.04	0.22	0.59	0.96	0.96	0	0.21	0.74	0.96	0.47	0	0	0.74	0	0.94	0.95	0.05	0.82	0.24	0	0	1	0	1	1	0.81	0	0.04	0	0.19	1	1	1	0.04	1
009	2	1 5E	0	0.98	0.57	0	0.27	0.32	1	0	0.04	1	0.04	0.22	0.59	0.96	0.96	0	0.21	0.74	0.96	0.47	0	0	0.74	0	0.94	0.95	0.05	0.82	0.24	0	0.46	1	0	1	1	0.2	0	0.04	0.91	0.68	1	1	1	0.04	1
009	2	1 6E	0	0.98	0.89	0	0.27	0.32	1	0	0.31	1	1	0.22	0.59	0.96	0.96	1	0.21	0.74	0.96	0.47	0	0	0.74	0	0.94	0.95	0.05	0.82	0.24	0	0.82	1	0	1	1	0.2	0	0.3	0	0	1	1	1	0.39	1
009	2	1 7E	0	0.98	0.89	0	0.27	0.32	1	0	0.04	1	0.04	0.22	0.59	0.96	0.96	1	0.21	0.74	0.96	0.47	0	0	0.74	0	0.94	0.95	0.05	0.82	0.24	0	0.82	1	0	1	1	0.2	0	0.04	0	0.19	1	1	1	0.04	1
009	2	1 8E	0	0.98	0.89	0	0.27	0.32	1	0	0.06	1	0.07	0.22	0.59	0.96	0.96	0	0.21	0.74	0.96	0.47	0	0	0.74	0	0.94	0.95	0.05	0.82	0.24	0	0.41	1	0	1	1	0.81	0	0.06	0.91	0.68	1	1	1	0.04	1
009	2	1 9E	0.97	0.98	0.9	0	0.27	0.32	1	0	0.04	1	0.04	0.22	0.59	0.96	0.96	0	0.21	0.74	0.96	0.47	0	0	0.74	0	0.94	0.95	0.05	0.82	0.24	0	0	1	0	1	1	0.81	0	0.04	0	0	1	1	1	0.04	1
009	2	2 10E	0	0.98	0.9	0	0.27	0.32	1	0	0.04	1	0.04	0.22	0.59	0.96	0.96	0	0.21	0.74	0.96	0.47	0	0	0.74	0	0.94	0.95	0.05	0.82	0.24	0	0	1	0	1	1	0.2	0	0.04	0	0.19	1	1	1	0.04	1
009	2	2 1E	0.97	0.98	0.63	0.99	0.27	0.32	1	0	0.04	1	0.04	0.22	0.59	0.96	0.96	1	0.21	0.74	0.96	0.47	0	0	0.74	0	0.94	0.95	0.05	0.82	0.24	0	0.61	1	0	1	1	0.81	0	0.04	0.91	0.68	1	1	1	0.04	1
009	2	2 2E	0.97	0.79	0.9	0	0.27	0.32	1	0	0.04	1	0.04	0.22	0.59	0.96	0.96	1	0.21	0.74	0.96	0.47	0	0	0.74	0	0.94	0.95	0.05	0.82	0.24	0	1	1	0	1	1	0.2	0	0.04	0.91	1	1	1	1	0.04	1
009	2	2 3E	0.97	0.98	0.87	0.99	0.27	0.32	1	0	0.31	1	1	0.22	0.59	0.96	0.96	1	0.21	0.74	0.96	0.47	0	0	0.74	0	0.94	0.95	0.05	0.82	0.24	0	0.99	1	0	1	1	0.81	0	0.3	0.91	0.68	1	1	1	0.39	1
009	2	2 4E	0.97	0.98	0.85	0	0.27	0.32	1	0	0.04	1	0.04	0.22	0.59	0.96	0.96	1	0.21	0.74	0.96	0.47	0	0	0.74	0	0.94	0.95	0.05	0.82	0.24	0	1	1	0	1	1	0.81	0	0.04	0.91	0.68	1	1	1	0.04	1
009	2	2 5E	0.96	0.98	0.89	0	0.27	0.32	1	0	0.04	0.98	0.04	0.22	0.07	0.96	0.96	1	0.21	0.74	0.96	0.47	0	0	0.74	0	0.94	0.95	0.05	0.82	0.24	0	1	1	0	1	1	0.8	0	0.04	0.91	0.68	1	1	1	0.04	1
009	2	2 6E	0	0.98	0.89	0.99	0.13	0.14	1	0	0.04	1	0.04	0.22	0.59	0.96	0.22	1	0.21	0.74	0.96	0.47	0	0	0.74	0	0.94	0.95	0.05	0.82	0.24	0	1	1	0	1	1	0.81	0	0.04	0.91	0.68	1	1	1	0.04	1
009	2	2 7E	0	0.98	0.88	0.99	0.27	0.32	1	0	0.04	1	0.04	0.22	0.59	0.96	0.96	0	0.21	0.74	0.96	0.47	0	0	0.74	0	0.94	0.95	0.05	0.82	0.24	0	1	1	0	1	1	0.47	0	0.04	0.91	0.68	1	1	1	0.04	1
009	2	2 8E	0	0.98	0.9	0.99	0.27	0.32	1	0	0.04	1	0.04	0.22	0.59	0.96	0.96	1	0.21	0.74	0.96	0.47	0	0	0.74	0	0.94	0.95	0.05	0.82	0.24	0	0.61	1	0	1	1	0.47	0	0.04	0.91	0.68	1	1	1	0.04	1
009	2	2 9E	0.97	0.98	0.9	0.99	0.27	0.32	1	0	0.04	1	0.04	0.22	0.59	0.96	0.96	1	0.21	0.74	0.96	0.47	0	0	0.74	0	0.94	0.95	0.05	0.82	0.24	0	0	1	0	1	1	0.81	0	0.04	0	0.19	1	1	1	0.04	1
009	3	1 10E	0	0.98	0.65	0.99	0.27	0.32	1	0	0.04	1	0.04	0.22	0.59	0.96	0.96	0	0.21	0.74	0.96	0.47	0	0	0.74	0	0.94	0.95	0.05	0.82	0.24	0	0	1	0	1	1	0.81	0	0.04	0.71	1	1	1	1	0.04	1
009	3	1 11E	0.06	0.98	0.87	0.99	0.27	0.32	1	0	0.04	1	0.04	0.22	0.59	0.96	0.96	0	0.21	0.21	0.96	0.47	0	0	0.74	0	0.94	0.95	0.05	0.82	0.24	0	0	1	0	1	1	0.47	0	0.04	0	0.19	1	1	1	0.04	1
009	3	1 12E	0	0.98	0.87	0.99	0.27	0.32	1	0	0.04	1	0.04	0.22	0.59	0.96	0.96	1	0.21	0.74	0.96	0.47	0	0	0.74	0	0.94	0.95	0.05	0.82	0.24	0	1	1	0	1	1	0.47	0	0.04	0.91	0.69	1	0.82	0.82	0.04	1
009	3	1 3E	0	0.98	0.87	0	0.27	0.32	1	0	0.04	1	0.04	0.22	0.59	0.96	0.96	0	0.21	0.74	0.96	0.47	0	0	0.74	0	0.94	0.95	0.05	0.82	0.24	0	1	1	0	1	1	0.2	0	0.04	0.91	0.68	1	1	1	0.04	1
009	3	1 4E	0	0.98	0.84	0	0.27	0.32	1	0	0.27	1	0.9	0.22	0.59	0.96	0.96	0	0.21	0.74	0.96	0.47	0	0	0.74	0	0.94	0.95	0.05	0.82	0.24	0	1	1	0	1	1	0.2	0	0.28	0	0.26	1	1	1	0.35	1
009	3	1 5E	0	0.98	0.88	0	0.27	0.32	1	0	0.04	1	0.04	0.22	0.59	0.96	0.96	0	0.21	0.74	0.96	0.47	0	0	0.74	0	0.94	0.95	0.05	0.82	0.24	0	1	1	0	1	1	0.81	0	0.04	0	0.19	1	1	1	0.04	1
009	3	1 6E	0	0.39	0.86	0.99	0.27	0.32	1	0	0.04	1	0.04	0.22	0.59	0.96	0.96	0.23	0.21	0.74	0.96	0.47	0	0	0.74	0	0.94	0.95	0.05	0.82	0.24	0	0	1	0	1	1	0.47	0	0.04	0	0.19	1	1	1	0.04	1

009	3	1	7E	0	0.98	0.89	0	0.27	0.32	1	0	0.04	1	0.04	0.22	0.59	0.96	0.96	0	0.21	0.74	0.96	0.47	0	0	0.74	0	0.94	0.95	0.05	0.82	0.24	0	1	1	0	1	1	0.42	0	0.04	0	0.19	1	1	1	0.04	1
009	3	1	8E	0	0.98	0.89	0	0.27	0.32	1	0	0.04	1	0.04	0.22	0.59	0.96	0.96	0	0.21	0.74	0.96	0.47	0	0	0.74	0	0.94	0.95	0.05	0.82	0.24	0	1	1	0	1	1	0.2	0	0.04	0	0.19	1	0.87	0.87	0.04	0.02
009	3	1	9E	0	0.98	0.64	0.99	0.27	0.32	1	0	0.04	1	0.04	0.22	0.59	0.96	0.96	0	0.21	0.74	0.96	0.47	0	0	0.74	0	0.94	0.95	0.05	0.82	0.24	0	1	1	0	1	1	0.47	0	0.04	0.91	0.68	1	1	1	0.04	1
009	3	2	10E	0.97	0.98	0.9	0	0.27	0.32	1	0	0.04	1	0.04	0.22	0.59	0.96	0.96	1	0.21	0.74	0.96	0.47	0	0	0.74	0	0.94	0.95	0.05	0.82	0.24	0	0.82	1	0	1	1	0.81	0	0.04	0.65	1	1	1	1	0.04	1
009	3	2	11E	0	0.98	0.65	0	0.27	0.32	1	0	0.3	1	0.97	0.22	0.59	0.96	0.96	0	0.21	0.74	0.96	0.47	0	0	0.74	0	0.94	0.95	0.05	0.82	0.24	0	0	1	0	1	1	0.2	0	0.29	0.91	0.68	1	1	1	0.38	1
009	3	2	12E	0.97	0.98	0.88	0	0.27	0.32	1	0	0.25	1	0.93	0.22	0.59	0.96	0.96	0	0.21	0.74	0.96	0.47	0	0	0.74	0	0.94	0.95	0.05	0.82	0.24	0	1	1	0	1	1	0.47	0	0.24	0.91	0.68	1	1	1	0.35	1
009	3	2	2E	0	0.98	0.89	0.99	0.27	0.32	1	0	0.31	1	1	0.22	0.59	0.96	0.96	0	0.21	0.74	0.96	0.47	0	0	0.74	0	0.94	0.95	0.05	0.82	0.24	0	0.96	1	0	1	1	0.47	0	0.3	0	0.19	1	1	1	0.39	1
009	3	2	3E	0	0.98	0.89	0	0.27	0.32	1	0	0.04	1	0.04	0.22	0.59	0.96	0.96	1	0.21	0.74	0.96	0.47	0	0	0.74	0	0.94	0.95	0.05	0.82	0.24	0	0.82	1	0	1	1	0.2	0.05	0.04	0	0.19	1	1	1	0.04	1
009	3	2	4E	0	0.98	0.89	0.99	0.27	0.32	1	0	0.04	1	0.04	0.22	0.59	0.96	0.96	0	0.21	0.74	0.96	0.47	0	0	0.74	0	0.94	0.95	0.05	0.82	0.24	0	1	1	0	1	1	0.47	0	0.04	0	0.19	1	1	1	0.04	1
009	3	2	5E	0	0.98	0.64	0	0.27	0.32	1	0	0.04	1	0.04	0.22	0.59	0.96	0.96	0	0.21	0.74	0.96	0.47	0	0	0.74	0	0.94	0.95	0.05	0.82	0.24	0	0.46	1	0	1	1	0.2	0	0.04	0.65	1	1	1	1	0.04	1
009	3	2	6E	0	0.98	0.89	0	0.27	0.32	1	0	0.04	1	0.04	0.22	0.59	0.96	0.96	0	0.21	0.74	0.96	0.47	0	0	0.74	0	0.94	0.95	0.05	0.82	0.24	0	0.61	1	0	1	1	0.2	0	0.04	0.91	0.68	1	1	1	0.04	1
009	3	2	8E	0	0.98	0.89	0.99	0.27	0.32	1	0	0.04	1	0.04	0.22	0.59	0.96	0.96	0	0.21	0.74	0.96	0.47	0	0	0.74	0	0.94	0.95	0.05	0.82	0.24	0	0.41	1	0	1	1	0.81	0	0.04	0.91	0.68	1	1	1	0.04	1
009	3	2	9E	0.97	0.98	0.89	0.99	0.27	0.32	1	0	0.04	1	0.04	0.22	0.59	0.96	0.96	0	0.21	0.74	0.96	0.47	0	0	0.74	0	0.94	0.95	0.05	0.82	0.24	0	1	1	0	1	1	0.81	0	0.04	0.91	0.68	1	1	1	0.04	1
009	4	1	10E	0	0.98	0.87	0	0.27	0.32	1	0	0.04	1	0.04	0.22	0.59	0.96	0.96	0	0.21	0.74	0.96	0.47	0	0	0.74	0	0.94	0.95	0.05	0.82	0.24	0	0.82	1	0	1	1	0.2	0	0.04	0.65	1	1	1	1	0.04	1
009	4	1	1E	0	0.98	0.89	0	0.27	0.32	1	0	0.04	1	0.04	0.22	0.59	0.96	0.96	1	0.21	0.74	0.96	0.47	0	0	0.74	0	0.94	0.95	0.05	0.82	0.24	0	0	1	0	1	1	0.2	0	0.04	0	0.19	1	1	1	0.04	1
009	4	1	2E	0	0	0.45	0	0.27	0.32	1	0	0.04	1	0.04	0.22	0.59	0.96	0.96	0	0.21	0.74	0.96	0.47	0	0	0.74	0	0.94	0.95	0.05	0.82	0.24	0	0	1	0	1	1	0.2	0	0.04	0	0.19	1	1	1	0.04	0.61
009	4	1	3E	0	0.98	0.63	0	0.27	0.32	1	0	0.04	1	0.04	0.22	0.59	0.96	0.96	1	0.21	0.74	0.96	0.47	0	0	0.74	0	0.94	0.95	0.05	0.82	0.24	0	0.96	1	0	1	1	0.2	0	0.04	0.91	0.68	1	1	1	0.04	1
009	4	1	4E	0	0.98	0.9	0	0.27	0.32	1	0	0.04	1	0.04	0.22	0.59	0.96	0.96	0	0.21	0.74	0.96	0.47	0	0	0.74	0	0.94	0.95	0.05	0.82	0.24	0	0.82	1	0	1	1	0.2	0	0.04	0	0	1	1	1	0.04	1
009	4	1	5E	0	0.98	0.9	0.99	0.27	0.32	1	0	0.04	1	0.04	0.22	0.59	0.96	0.96	0	0.21	0.74	0.96	0.47	0	0	0.74	0	0.94	0.95	0.05	0.82	0.24	0	0	1	0	1	1	0.47	0	0.04	0	0.19	1	1	1	0.04	1
009	4	1	6E	0	0.98	0.9	0	0.27	0.32	1	0	0.04	1	0.05	0.22	0.59	0.96	0.96	0	0.21	0.74	0.96	0.47	0	0	0.74	0	0.94	0.95	0.05	0.82	0.24	0	0	1	0	1	1	0.2	0	0.04	0	0.19	1	1	1	0.04	0.02
009	4	1	7E	0	0.98	0.89	0	0.27	0.32	1	0	0.04	1	0.04	0.22	0.59	0.96	0.96	0	0.21	0.74	0.96	0.47	0	0	0.74	0	0.94	0.95	0.05	0.82	0.24	0	0.61	1	0	1	1	0.2	0	0.04	0	0.19	1	1	1	0.04	1
009	4	1	8E	0	0.98	0.89	0.99	0.27	0.32	1	0	0.04	1	0.04	0.22	0.59	0.96	0.96	0	0.21	0.74	0.96	0.47	0	0	0.74	0	0.94	0.95	0.05	0.82	0.24	0	0.61	1	0	1	1	0.81	0	0.04	0	0.19	1	1	1	0.04	1
009	6	2	10	0	0.33	0.88	0.1	0.38	0.97	0.35	0	0.43	0.92	0.3	0.11	0.6	0.84	0.06	0	0.96	0.28	0.04	0	0	0	0	0	0	0	0	0	0	0	0	1	0	1	0.03	0.29	0	0.99	0.19	0.19	0	0.15	0.15	0.37	0.88
009	6	2	11	0	0.33	0.88	0.1	0.38	0.97	0.35	0	0.43	0.92	0.3	0.11	0.6	0.84	0.06	0	0.96	0.28	0.04	0	0	0	0	0	0	0	0	0	0	0	0	1	0	1	0.03	0.29	0	0.99	0.19	0.19	0	0.15	0.15	0.37	0.88
009	6	2	12	0	0.33	0.88	0.1	0.38	0.97	0.35	0	0.43	0.92	0.3	0.11	0.6	0.84	0.06	0	0.96	0.28	0.04	0	0	0	0	0	0	0	0	0	0	0	0	1	0	1	0.03	0.29	0	0.99	0.19	0.19	0	0.15	0.15	0.37	0.88
009	6	2	3	0	0.33	0.88	0.1	0.38	0.97	0.35	0	0.43	0.92	0.3	0.11	0.6	0.84	0.06	0	0.96	0.28	0.04	0	0	0	0	0	0	0	0	0	0	0	0	1	0	1	0.03	0.29	0	0.99	0.19	0.19	0	0.15	0.15	0.37	0.88
009	6	2	4	0	0.33	0.88	0.1	0.38	0.97	0.35	0	0.43	0.92	0.3	0.11	0.6	0.84	0.06	0	0.96	0.28	0.04	0	0	0	0	0	0	0	0	0	0	0	0	1	0	1	0.03	0.29	0	0.99	0.19	0.19	0	0.15	0.15	0.37	0.88
009	6	2	5	0	0.33	0.88	0.1	0.38	0.97	0.35	0	0.43	0.92	0.3	0.11	0.6	0.84	0.06	0	0.96	0.28	0.04	0	0	0	0	0	0	0	0	0	0	0	0	1	0	1	0.03	0.29	0	0.99	0.19	0.19	0	0.15	0.15	0.37	0.88
009	6	2	6	0	0.33	0.88	0.1	0.38	0.97	0.35	0	0.43	0.92	0.3	0.11	0.6	0.83	0.06	0	0.96	0.28	0.04	0	0	0	0	0	0	0	0	0	0	0	0	1	0	1	0.03	0.29	0	0.99	0.19	0.19	0	0.15	0.15	0.37	0.88
009	6	2	7	0	0.33	0.88	0.1	0.38	0.97	0.35	0	0.43	0.92	0.3	0.11	0.6	0.84	0.06	0	0.96	0.28	0.04	0	0	0	0	0	0	0	0	0	0	0	0	1	0	1	0.03	0.29	0	0.67	0.19	0.19	0	0.15	0.15	0.37	0.88
009	6	2	8	0	0.33	0.88	0.1	0.38	0.97	0.35	0	0.43	0.92	0.3	0.11	0.6	0.83	0.06	0	0.96	0.28	0.04	0	0	0	0	0	0	0	0	0	0	0	0	1	0	1	0.03	0.29	0	0.99	0.19	0.19	0	0.15	0.15	0.37	0.88
009	6	2	9	0	0.33	0.88	0.1	0.38	0.97	0.35	0	0.43	0.92	0.3	0.11	0.6	0.84	0.06	0	0.96	0.28	0.04	0	0	0	0	0	0	0	0	0	0	0	0	1	0	1	0.03	0.29	0	0.99	0.19	0.19	0	0.15	0.15	0.37	0.88
009	28	1	3	0	0	0.1	0.3	0.99	0.4	0.54	0.93	0.04	1	0.04	0.21	0.61	0.96	0.98	0	0.21	0.65	0.53	0.47	0.58	0.59	0.68	0	0.82	0.54	0.03	0	0.09	0	0	0.08	1	0	0.03	0.2	0	0.04	0	0.19	0	0.21	0.21	0.03	0.94
009	28	1	4	0	0	0.1	0.3	0.99	0.4	0.54	0.93	0.04	1	0.04	0.21	0.61	0.96	0.98	0	0.21	0.65	0.53	0.47	0.58	0.59	0.68	0	0.82	0.54	0.03	0	0.09	0	0	0.08	1	0	0.03	0.2	0	0.04	0	0.19	0	0.21	0.21	0.03	0.94
009	28	1	5	0	0	0.1	0.3	0.99	0.4	0.54	0.93	0.04	1	0.04	0.21	0.61	0.96	0.98	0	0.21	0.65	0.53	0.47	0.58	0.59	0.68	0	0.82	0.54	0.03	0	0.09	0	0	0.08	1	0	0.03	0.2	0	0.04	0	0.19	0	0.21	0.21	0.03	0.94
009	28	1	6	0	0	0.1	0.3	0.99	0.4	0.54	0.93	0.04	1	0.04	0.21	0.61	0.96	0.98	0	0.21	0.65	0.53	0.47	0.58	0.59	0.68	0	0.82	0.54	0.03	0	0.09	0	0	0.08	1	0	0.03	0.2	0	0.04	0	0.19	0	0.21	0.21	0.03	0.94
009	28	1	7	0	0	0.1	0.3	0.99	0.4	0.54	0.93	0.04	1	0.04	0.21	0.61	0.96	0.98	0	0.21	0.65	0.53	0.47	0	0	0.68	0	0.82	0.54	0.03	0	0.09	0	0	0.08	1	0	0.03	0.2	0	0.04	0	0.19	0	0.21	0.21	0.03	0.94
015	-1	1	10	0	0	0.69	0	0.89	0.96	0.34	0	0.22	0.94	0.4	0.24	0.6	0.83	0.07	0	0.21	0.26	0.05	0	0	0	0	0	0	0	0	0	0	0	0	1	0	1	0.03	0.2	0	0.28	0	0	0.61	0.14	0.14	1	0.89
015	-1	1	12	0	0.33	0.69	0	0.89	0.97	0.34	0	0.22	0.94	0.4	0.24	0.6	0.83	0.07	0	0.21	0.26	0.05	0	0	0	0	0	0	0	0	0	0	0	0	1	0	1	0.03	0.2	0	0.28	0	0.19	0.61	0.14	0.14	1	0.89
015	-1	1	1	0	0.33	0.68	0	0.89	0.96	0.34	0	0.22	0.94	0.4	0.24	0.6	0.83	0.07	0	0.21	0.26	0.05	0	0	0	0	0	0	0	0	0	0	0	0	1	0	1	0.03	0.2	0	0.28	0	0.19	0.61	0.14	0.14	1	0.89
015	-1	1	2	0	0.33	0.69	0	0.89	0.97	0.34	0	0.22	0.94	0.24	0.24	0.6	0.83	0.07	0	0.21	0.26	0.05	0	0	0	0	0	0	0	0	0	0	0	0	1	0	1	0.03	0.2	0	0.25	0	0.19	0.61	0.14	0.14	0.64	0.89
015	-1	1	3	0	0.33	0.69	0	0.89	0.96	0.34	0	0.22	0.94	0.4	0.24	0.6	0.83	0.07	0	0.21	0.26	0.05	0	0	0	0	0	0	0	0	0	0	0	0	1	0	1	0.03	0.2	0	0.28	0	0.19	0.61	0.14	0.14	1	0.89
015	-1	1	4	0	0	0.68	0	0.89	0.97	0.34	0	0.22	0.94	0.4	0.23	0.6	0.83	0.07	0	0.21	0.26	0.05	0	0	0	0	0	0	0	0	0	0	0	0	1	0	1	0.03	0.2	0	0.28	0	0.19	0.61	0.14	0.14	1	0.89
015	-1	1	6	0	0.33	0.69	0	0.89	0.96	0.34	0	0.22	0.94	0.4	0.23	0.6	0.83	0.07	0	0.21	0.26	0.05	0	0	0	0	0	0	0	0	0	0	0	0	1	0	1	0.03	0.2	0	0.28	0	0.19	0.61	0.14	0.14	1	0.89
015	-1	1	7	0	0.33	0.69	0	0.89	0.97	0.34	0	0.22	0.94	0.4	0.24	0.6	0.83	0.07	0	0.21	0.26	0.05	0	0	0	0	0	0	0	0	0	0	0	0	1	0	1	0.03	0.2	0	0.28	0	0.19	0.61	0.14	0.14	1	0.89
015	-1	1	8	0	0.33	0.69	0	0.89	0.97	0.34	0	0.22	0.94	0.4	0.24	0.6	0.83	0.07	0	0.21	0.26	0.05	0	0	0	0	0	0	0	0	0	0	0	0	1	0	1	0.03	0.2	0	0.28	0	0.19	0.61	0.14	0.14	1	0.89
015	-1	1	9	0	0.33	0.68	0	0.89	0.97	0.34	0	0.22	0.94	0.4	0.23	0.6	0.83	0.07	0	0.21	0.26	0.05	0	0	0	0	0	0	0	0	0	0	0	0	1	0	1	0.03	0.2	0	0.28	0	0.19	0.61	0.14	0.14	1	0.89
015	0	1	10	0	0	0.68	0	0.89	0.97	0.34	0	0.22	0.94	0.4	0.24	0.6	0.83	0.07	0	0.21	0.26	0.05	0	0	0	0	0	0	0	0	0	0	0	0	1	0	1	0.03	0.2	0	0.28	0	0.19	0.61	0.14	0.14	1	0.89
015	0	1	11	0	0.33	0.69	0	0.89	0.97	0.34	0	0.22	0.94	0.4	0.24	0.6	0.83	0.07	0	0.21	0.26	0.05	0	0	0	0	0	0	0	0	0	0	0	0	1	0	1	0.03	0.2	0	0.28	0	0.19	0.61	0.14	0.14	1	0.89
015	0	1	12	0	0.33	0.69	0	0.89	0.97	0.34	0	0.22	0.94	0.4	0.23	0.6	0.83	0.07	0	0.21	0.26	0.05	0	0	0	0	0	0	0	0	0	0	0	0	1	0	1	0.03	0.2	0	0.28	0	0.19	0.61	0.14	0.14	1	0.89
015	0	1	2	0	0.33	0.69	0	0.89	0.97	0.34	0	0.31	0.94	0.4	0.24	0.6	0.83	0.07	0	0.21	0.26	0.05	0	0	0	0	0	0	0	0	0	0	0	0	1	0	1	0.03	0.2	0	0.28	0	0.19	0.61	0.14	0.14	1	0.89
015	0	1	3	0	0	0.69	0	0.89	0.96	0.34	0	0.31	0.94	0.4	0.24	0.6	0.83	0.07	0	0.21	0.26	0.05	0	0	0	0	0	0	0	0	0	0	0	0	1	0	1	0.03	0.2	0	0.28	0	0	0.61	0.14	0.14	1	0.89
015	0	1	4	0	0	0.69	0	0.89	0.97	0.34	0	0.22	0.94	0.4	0.24	0.6	0.83	0.07	0	0.21	0.26	0.05	0	0	0	0	0	0	0	0	0	0	0	0	1	0	1	0.03	0.2	0	0.28	0	0	0.61	0.14	0.14	1	0.89
015	0	1	5	0	0	0.69	0	0.89	0.97	0.34	0	0.22	0.94	0.4	0.24	0.51	0.83	0.07	0	0.21	0.26	0.05	0	0	0	0	0	0	0	0	0	0	0	0	1	0	1	0.03	0.2	0	0.28	0	0	0.61	0.14	0.14	1	0.89
015	0	1	7	0	0	0.69	0	0.89	0.97	0.34	0	0.22	0.94	0.4	0.23	0.6	0.83	0.07	0	0.21	0.26	0.05	0	0	0	0	0	0	0	0	0	0	0	0	1	0	1	0.03	0.2	0	0.28	0	0	0.61	0.14	0.14	1	0.89
015	0	1	8	0	0	0.69	0	0.89	0.97	0.34	0	0.22	0.94	0.4	0.24	0.6	0.83	0.07	0	0.21	0.26	0.05	0	0	0	0	0	0	0	0	0	0	0	0	1	0	1	0.03	0.2	0	0.28	0	0	0.61	0.14	0.14	1	0.89
015	0	1	9	0	0	0.69	0	0.89	0.97	0.34	0	0.22	0.94	0.4	0.24	0.6	0.83	0.07	0	0.21	0.26	0.05	0	0	0	0	0	0	0	0	0	0	0	0	1	0	1	0.03	0.2	0	0.28	0	0	0.61	0.14	0.14	1	0.89
015	0	2	10E	0	0.98	0.88	0.99	0.27	0.32	1	0	0.06	1	0.12	0.22	0.59	0.96	0.96	1	0.21	0.74	0.96	0.47	0	0	0.74	0	0.94	0.95	0.05	0.82	0.24	0	0.97	1	0	1	1	0.2	0	0.06	0.91	0.68	1	1	1	0.08	1
015	0	2	1E	0	0.98	0.63	0	0.27	0.32	1	0	0.04	1	0.04	0.22	0.59	0.96	0.96	1	0.21	0.74	0.96	0.47	0	0	0.74	0	0.94	0.95	0.05	0.82	0.24	0	0	1	0	1	1	0.81	0	0.04	0	0.19	1	1	1	0.04	1
015	0	2	2E	0.97	0.98	0.89	0	0.27	0.32	1	0	0.04	1	0.04	0.22	0.59	0.96	0.96	0	0.21	0.74	0.96	0.47	0	0	0.74	0	0.94	0.95	0.05	0.82	0.24	0	1	1	0	1	1	0.2	0	0.04	0.91	1	1	1	1	0.04	1
015	0	2	3E	0	0.98	0.58	0	0.27	0.32	1	0	0.04	1	0.04	0.22	0.59	0.96	0.96	1	0.21	0.74	0.96	0.47	0	0	0.74	0	0.94	0.95	0.05	0.82	0.24	0	0.61	1	0	1	1	0.2	0	0.04	0.91	0.68	1	1	1	0.04	1
015	0	2	4E	0	0.98	0.64	0.99	0.27	0.32	1	0	0.04	1	0.04	0.22	0.59	0.96	0.96	1	0.21	0.74	0.96	0.47	0	0	0.74	0	0.94	0.95	0.05	0.82	0.24	0	1	1	0	1	1	0.47	0	0.04	0.91	0.68	1	1	1	0.04	1
015	0	2	5E	0	0.98	0.87	0.99	0.27	0.32	1	0	0.31	1	1	0.22	0.59	0.96	0.96	0	0.21	0.74	0.96	0.47	0	0	0.74	0	0.94	0.95	0.05	0.82	0.24	0	0	1	0	1	1	0.81	0	0.3	0.91	0.68	1	1	1	0.39	1
015	0	2	6E	0	0.98	0.6	0	0.27	0.32	1	0	0.04	1	0.04	0.22	0.59	0.96	0.96	0	0.21	0.74	0.96	0.47	0	0	0.74	0	0.94	0.95	0.05	0.82	0.24	0	1	1	0	1	1	0.2	0	0.04	0.88	0.66	1	1	1	0.04	1
015	0	2	7E	0	0.98	0.88	0.1	0.27	0.32	1	0	0.04	1	0.04	0.22	0.59	0.96	0.96	0	0.21	0.74	0.96	0.47	0	0	0.74	0	0.94	0.95	0.05	0.82	0.24	0	0.61	1	0	1	1	0.2	0	0.04	0	0.19	1	1	1	0.04	1
015	0	2	8E	0	0.98	0.52	0.99	0.27	0.32	1	0	0.04	1	0.04	0.22	0.59	0.96	0.96	0	0.21	0.74	0.96	0.47	0	0	0.74	0	0.94	0.95	0.05	0.82	0.24	0	0.61	1	0	1	1	0.47	0	0.04	0	0.19	1	1	1	0.04	1
015	0	2	9E	0	0.98	0.89	0	0.27	0.32	1	0	0.04	1	0.04	0.22	0.59	0.96	0.96	0	0.21	0.74	0.96	0.47	0	0	0.74	0	0.94	0.95	0.05	0.82	0.24	0	0	1	0	1	1	0.2	0	0.04	0	0.19	1	1	1	0.04	1
015	2	1	10E	0	0.98	0.85	0	0.27	0.32	1	0	0.04	1	0.04	0.22	0.59	0.96	0.96	0	0.21	0.74	0.96	0.47	0	0	0.74	0	0.94	0.95	0.05	0.82	0.24	0	0.82	1	0	1	1	0.2	0	0.04	0	0.19	1	1	1	0.04	1
015	2	1	1E	0	0.98	0.85	0	0.27	0.32	1	0	0.04	1	0.04	0.22	0.59	0.96	0.96	0	0.21	0.74	0.96	0.47	0	0	0.74	0	0.94	0.95	0.05	0.82	0.24	0	0.82	1	0	1	1	0.2	0	0.04	0	0.19	1	1	1	0.04	1
015	2	1	2E	0	0.98	0.78	0.99	0.27	0.32	1	0	0.04	1	0.04	0.22	0.59	0.96	0.96	0	0.21	0.74	0.96	0.47	0	0	0.74	0	0.94	0.95	0.05	0.82	0.24	0	1	1	0	1	1	0.47	0	0.04	0	0.19	1	1	1	0.04	1

015	2	1	3E	0	0.98	0.9	0	0.27	0.32	1	0	0.04	1	0.04	0.22	0.59	0.96	0.96	1	0.21	0.74	0.96	0.47	0	0	0.74	0	0.94	0.95	0.05	0.82	0.24	0	0	1	0	1	1	0.2	0	0.04	0	0.19	1	1	1	0.04	1
015	2	1	4E	0	0.98	0.87	0	0.27	0.32	1	0	0.04	1	0.04	0.22	0.59	0.96	0.96	0	0.21	0.74	0.96	0.47	0	0	0.74	0	0.94	0.95	0.05	0.82	0.24	0	0.82	1	0	1	1	0.2	0	0.04	0	0.19	1	1	1	0.04	1
015	2	1	5E	0	0.98	0.87	0	0.27	0.32	1	0	0.31	1	1	0.22	0.59	0.96	0.96	0	0.21	0.74	0.96	0.47	0	0	0.74	0	0.94	0.95	0.05	0.82	0.24	0	0	1	0	1	1	0.2	0	0.3	0	0.19	1	1	1	0.39	1
015	2	1	6E	0	0.98	0.9	0	0.27	0.32	1	0	0.04	1	0.04	0.22	0.59	0.96	0.96	0	0.21	0.74	0.96	0.47	0	0	0.74	0	0.94	0.95	0.05	0.82	0.24	0	0.82	1	0	1	1	0.2	0	0.04	0	0.19	1	1	1	0.04	1
015	2	1	7E	0.97	0.98	0.67	0	0.27	0.32	1	0	0.04	1	0.04	0.22	0.59	0.96	0.96	1	0.21	0.74	0.96	0.47	0	0	0.74	0	0.94	0.95	0.05	0.82	0.24	0	0	1	0	1	1	0.81	0	0.04	0	0.19	1	1	1	0.04	1
015	2	1	8E	0	0.98	0.6	0	0.27	0.32	1	0	0.04	1	0.04	0.22	0.59	0.96	0.96	0	0.21	0.74	0.96	0.47	0	0	0.74	0	0.94	0.95	0.05	0.82	0.24	0	1	1	0	1	1	0.2	0	0.04	0.91	0.68	1	1	1	0.04	1
015	2	1	9E	0	0.98	0.89	0	0.27	0.32	1	0	0.04	1	0.04	0.22	0.59	0.96	0.96	1	0.21	0.74	0.96	0.47	0	0	0.74	0	0.94	0.95	0.05	0.82	0.24	0	0	1	0	1	1	0.81	0	0.04	0	0	1	1	1	0.04	0.02
015	2	2	10E	0.97	0.98	0.61	0.99	0.27	0.32	1	0	0.04	1	0.04	0.22	0.59	0.96	0.96	0	0.21	0.74	0.96	0.47	0	0	0.74	0	0.94	0.95	0.05	0.82	0.24	0	0.82	1	0	1	1	0.81	0	0.04	0.91	0.68	1	1	1	0.04	1
015	2	2	12E	0.97	0.98	0.87	0.99	0.27	0.32	1	0	0.04	1	0.04	0.22	0.59	0.96	0.96	0	0.21	0.74	0.96	0.47	0	0	0.74	0	0.94	0.95	0.05	0.82	0.24	0	0.61	1	0	1	1	0.47	0	0.04	0.91	0.68	1	1	1	0.04	1
015	2	2	1E	0.97	0.98	0.5	0	0.27	0.32	1	0	0.04	1	0.04	0.22	0.59	0.96	0.96	0	0.21	0.74	0.96	0.47	0	0	0.74	0	0.94	0.95	0.05	0.82	0.24	0	0.61	1	0	1	1	0.2	0	0.04	0	0.19	1	1	1	0.04	1
015	2	2	2E	0	0.98	0.83	0	0.27	0.32	1	0	0.31	1	1	0.22	0.59	0.96	0.96	1	0.21	0.74	0.96	0.47	0	0	0.74	0	0.94	0.95	0.05	0.82	0.24	0	1	1	0	1	1	0.2	0	0.3	0.91	0.68	1	1	1	0.39	1
015	2	2	3E	0.97	0.98	0.63	0	0.27	0.32	1	0	0.04	1	0.04	0.22	0.59	0.96	0.96	0	0.21	0.74	0.96	0.47	0	0	0.74	0	0.94	0.95	0.05	0.82	0.24	0	1	1	0	1	1	0.81	0	0.04	0.56	0.86	1	1	1	0.04	1
015	2	2	5E	0.97	0.98	0.9	0	0.27	0.32	1	0	0.04	1	0.04	0.22	0.59	0.96	0.96	1	0.21	0.74	0.96	0.47	0	0	0.74	0	0.94	0.95	0.05	0.82	0.24	0	1	1	0	1	1	0.81	0	0.04	0.91	1	1	1	1	0.04	1
015	2	2	6E	0	0.98	0.65	0.99	0.27	0.32	1	0	0.31	1	1	0.22	0.59	0.96	0.96	0	0.21	0.74	0.96	0.47	0	0	0.74	0	0.94	0.95	0.05	0.82	0.24	0	1	1	0	1	1	0.47	0	0.3	0.65	1	1	1	1	0.39	1
015	2	2	7E	0.97	0.98	0.87	0.99	0.27	0.32	1	0	0.04	1	0.04	0.22	0.59	0.96	0.96	1	0.21	0.74	0.96	0.47	0	0	0.74	0	0.94	0.95	0.05	0.82	0.24	0	1	1	0	1	1	0.81	0	0.04	0.65	1	1	1	1	0.04	1
015	2	2	8E	0	0.98	0.63	0	0.27	0.32	1	0	0.04	1	0.04	0.22	0.59	0.96	0.96	0	0.21	0.74	0.96	0.47	0	0	0.74	0	0.94	0.95	0.05	0.82	0.24	0	0.41	1	0	1	1	0.81	0	0.04	0	0	1	1	1	0.04	1
015	2	2	9E	0	0.98	0.9	0	0.27	0.32	1	0	0.04	1	0.04	0.22	0.59	0.96	0.96	0	0.21	0.74	0.96	0.47	0	0	0.74	0	0.94	0.95	0.05	0.82	0.24	0	0	1	0	1	1	0.81	0	0.04	0	0.19	1	1	1	0.04	1
015	3	1	10	0	0.98	0.6	0.99	0.27	0.32	1	0	0.31	1	1	0.22	0.59	0.96	0.96	0	0.21	0.74	0.96	0.47	0	0	0.74	0	0.94	0.95	0.05	0.82	0.24	0	0	1	0	1	1	0.47	0	0.3	0.65	1	1	1	1	0.39	1
015	3	1	11	0	0.98	0.35	0	0.27	0.32	1	0	0.04	1	0.04	0.22	0.59	0.96	0.96	0	0.21	0.74	0.96	0.47	0	0	0.74	0	0.94	0.95	0.05	0.82	0.24	0	1	1	0	1	1	0.2	0	0.04	0	0.19	1	1	1	0.04	1
015	3	1	12	0	0.98	0.78	0.99	0.27	0.32	1	0	0.04	1	0.04	0.22	0.59	0.96	0.96	0	0.21	0.74	0.96	0.47	0	0	0.74	0	0.94	0.95	0.05	0.82	0.24	0	0.82	1	0	1	1	0.53	0	0.04	0	0.19	1	1	1	0.04	1
015	3	1	2	0	0.98	0.8	0	0.27	0.32	1	0	0.04	1	0.04	0.22	0.59	0.96	0.96	1	0.21	0.74	0.96	0.47	0	0	0.74	0	0.94	0.95	0.05	0.82	0.24	0	0	1	0	1	1	0.2	0	0.04	0	0.19	1	0.99	0.99	0.04	1
015	3	1	3	0	0.98	0.7	0.99	0.27	0.32	1	0	0.1	1	0.12	0.22	0.59	0.96	0.96	1	0.21	0.74	0.96	0.47	0	0	0.74	0	0.94	0.95	0.05	0.82	0.24	0	0	1	0	1	1	0.81	0	0.1	0.91	0.68	1	1	1	0.08	1
015	3	1	4	0	0.98	0.86	0	0.27	0.32	1	0	0.04	1	0.1	0.22	0.59	0.96	0.96	0	0.21	0.74	0.96	0.47	0	0	0.74	0	0.94	0.95	0.05	0.82	0.24	0	0.61	1	0	1	1	0.81	0	0.04	0.91	0.69	1	1	1	0.04	1
015	3	1	5	0	0.98	0.89	0	0.27	0.32	1	0	0.31	1	1	0.22	0.59	0.96	0.96	0	0.21	0.74	0.96	0.47	0	0	0.74	0	0.94	0.95	0.05	0.82	0.24	0	0.61	1	0	1	1	0.81	0	0.3	0.91	0.68	1	1	1	0.39	1
015	3	1	6	0.97	0.98	0.6	0	0.27	0.32	1	0	0.04	1	0.04	0.22	0.59	0.96	0.96	0	0.21	0.74	0.96	0.47	0	0	0.74	0	0.94	0.95	0.05	0.82	0.24	0	0	1	0	1	1	0.2	0	0.04	0.65	1	1	1	1	0.04	1
015	3	1	7	0	0.98	0.89	0	0.27	0.32	1	0	0.04	1	0.04	0.22	0.59	0.96	0.96	0	0.21	0.74	0.96	0.47	0	0	0.74	0	0.94	0.95	0.05	0.82	0.24	0	0	1	0	1	1	0.2	0	0.04	0.91	0.68	1	1	1	0.04	1
015	3	1	9	0	0.98	0.76	0.1	0.27	0.32	1	0	0.04	1	0.04	0.22	0.59	0.96	0.96	0	0.21	0.74	0.96	0.47	0	0	0.74	0	0.94	0.95	0.05	0.82	0.24	0	0	1	0	1	1	0.81	0	0.04	0	0.19	1	1	1	0.04	1
015	3	2	10E	0.97	0.98	0.83	0.99	0.27	0.32	1	0	0.04	1	0.04	0.22	0.59	0.96	0.96	1	0.21	0.74	0.96	0.47	0	0	0.74	0	0.94	0.95	0.05	0.82	0.24	0	1	1	0	1	1	0.81	0	0.04	0	0	1	1	1	0.04	1
015	3	2	11E	0.97	0.98	0.89	0	0.27	0.32	1	0	0.04	1	0.04	0.22	0.59	0.96	0.96	0	0.21	0.74	0.96	0.47	0	0	0.74	0	0.94	0.95	0.05	0.82	0.24	0	0.96	1	0	1	1	0.81	0	0.04	0	0	1	1	1	0.04	1
015	3	2	2E	0.97	0.98	0.9	0	0.27	0.32	1	0	0.04	1	0.04	0.22	0.59	0.96	0.96	0	0.21	0.74	0.96	0.47	0	0	0.74	0	0.94	0.95	0.05	0.82	0.24	0	0.61	1	0	1	1	0.81	0	0.04	0.91	0.68	1	1	1	0.04	1
015	3	2	3E	0.97	0.98	0.59	0.99	0.27	0.32	1	0	0.04	1	0.04	0.22	0.59	0.96	0.96	0	0.21	0.74	0.96	0.47	0	0	0.74	0	0.94	0.95	0.05	0.82	0.24	0	0.72	1	0	1	1	0.81	0	0.04	0	0.19	1	1	1	0.04	1
015	3	2	4E	0.97	0.98	0.89	0	0.27	0.32	1	0	0.04	1	0.04	0.22	0.59	0.96	0.96	0	0.21	0.74	0.96	0.47	0	0	0.74	0	0.94	0.95	0.05	0.82	0.24	0	1	1	0	1	1	0.81	0	0.04	0	0.19	1	1	1	0.04	1
015	3	2	5E	0	0.98	0.87	0.99	0.27	0.32	1	0	0.04	1	0.04	0.22	0.59	0.96	0.96	1	0.21	0.74	0.96	0.47	0	0	0.74	0	0.94	0.95	0.05	0.82	0.24	0	1	1	0	1	1	0.47	0	0.04	0	0	1	1	1	0.04	1
015	3	2	6E	0	0.98	0.89	0.99	0.27	0.32	1	0	0.04	1	0.04	0.22	0.59	0.96	0.96	0.36	0.21	0.74	0.96	0.47	0	0	0.74	0	0.94	0.95	0.05	0.82	0.24	0	0.61	1	0	1	1	0.2	0	0.04	0.91	0.68	1	1	1	0.04	1
015	3	2	7E	0	0.98	0.89	0.99	0.27	0.32	1	0	0.04	1	0.04	0.22	0.59	0.96	0.96	0	0.21	0.74	0.96	0.47	0	0	0.74	0	0.94	0.95	0.05	0.82	0.24	0	0.82	1	0	1	0.03	0.81	0	0.04	0.65	1	0	1	1	0.04	1
015	3	2	8E	0	0.98	0.89	0	0.27	0.32	1	0	0.04	1	0.04	0.22	0.59	0.96	0.96	1	0.21	0.74	0.96	0.47	0	0	0.74	0	0.94	0.95	0.05	0.82	0.24	0	0.61	1	0	1	1	0.2	0	0.04	0	0	1	1	1	0.04	1
015	3	2	9E	0.2	0.98	0.87	0.99	0.27	0.32	1	0	0.25	1	0.85	0.22	0.59	0.96	0.96	0	0.21	0.74	0.96	0.47	0	0	0.74	0	0.94	0.95	0.05	0.82	0.24	0	0.82	1	0	1	1	0.27	0	0.28	0.91	0.68	1	1	1	0.34	1
015	4	1	10E	0.97	0.98	0.65	0.99	0.27	0.32	1	0	0.04	1	0.04	0.22	0.59	0.96	0.96	0.22	0.21	0.74	0.96	0.47	0	0	0.74	0	0.94	0.95	0.05	0.82	0.24	0	0.41	1	0	1	1	0.81	0	0.04	0.91	1	1	1	1	0.04	1
015	4	1	1E	0	0.98	0.9	0	0.27	0.32	1	0	0.04	1	0.04	0.22	0.59	0.96	0.96	1	0.21	0.74	0.96	0.47	0	0	0.74	0	0.94	0.95	0.05	0.82	0.24	0	0	1	0	1	1	0.2	0	0.04	0	0	1	1	1	0.04	1
015	4	1	2E	0	0.98	0.87	0	0.27	0.32	1	0	0.04	1	0.04	0.22	0.59	0.96	0.96	0	0.21	0.74	0.96	0.47	0	0	0.74	0	0.94	0.95	0.05	0.82	0.24	0	0.61	1	0	1	1	0.2	0	0.04	0.91	0.68	1	1	1	0.04	1
015	4	1	3E	0.92	0.98	0.76	0.66	0.27	0.32	1	0	0.04	1	0.09	0.22	0.59	0.96	0.96	0	0.21	0.74	0.96	0.47	0	0	0.74	0	0.94	0.95	0.05	0.82	0.24	0	0.61	1	0	1	1	0.2	0	0.04	0.91	0.68	1	1	1	0.04	1
015	4	1	4E	0	0.98	0.87	0.99	0.27	0.32	1	0	0.04	1	0.04	0.22	0.59	0.96	0.96	0	0.21	0.74	0.96	0.47	0	0	0.74	0	0.94	0.95	0.05	0.82	0.24	0	0	1	0	1	1	0.47	0	0.04	0	0.19	1	1	1	0.04	1
015	4	1	5E	0.97	0.98	0.87	0.99	0.27	0.32	1	0	0.31	1	1	0.27	0.59	0.96	0.96	0	0.21	0.74	0.96	0.47	0	0	0.74	0	0.94	0.95	0.05	0.82	0.24	0	0.61	1	0	1	1	0.47	0	0.3	0.65	1	1	1	1	0.39	1
015	4	1	7E	0	0.98	0.63	0	0.27	0.32	1	0	0.04	1	0.04	0.22	0.59	0.96	0.96	1	0.21	0.74	0.96	0.47	0	0	0.74	0	0.94	0.95	0.05	0.82	0.24	0	0.41	1	0	1	1	0.81	0	0.04	0	0	1	1	1	0.04	1
015	4	1	8E	0	0.98	0.88	0	0.27	0.32	1	0	0.04	1	0.04	0.22	0.59	0.96	0.96	0	0.21	0.74	0.96	0.47	0	0	0.74	0	0.94	0.95	0.05	0.82	0.24	0	0	1	0	1	1	0.81	0	0.04	0	0.19	1	1	1	0.04	1
015	4	1	9E	0.97	0.98	0.9	0.99	0.27	0.32	1	0	0.04	1	0.04	0.22	0.59	0.96	0.96	1	0.21	0.74	0.96	0.47	0	0	0.74	0	0.94	0.95	0.05	0.82	0.24	0	0.61	1	0	1	1	0.81	0	0.04	0	0.19	1	1	1	0.04	1
016	0	1	10	0	0	0.87	0.18	0.89	0.96	0.35	0.84	0.41	0.94	0.4	0.11	0.6	0.83	0.06	0	0.96	0.23	0.04	0	0	0	0	0	0	0	0	0	0	0	0	1	0	1	0.03	0.26	0	0.99	0	0.19	0.95	0.15	0.15	1	0.88
016	0	1	11	0	0	0.87	0.18	0.89	0.96	0.35	0.84	0.41	0.94	0.4	0.11	0.6	0.83	0.06	0	0.96	0.23	0.04	0	0	0	0	0	0	0	0	0	0	0	0	1	0	1	0.03	0.26	0	0.99	0	0.19	0.95	0.15	0.15	1	0.88
016	0	1	12	0	0	0.87	0.18	0.89	0.96	0.35	0.84	0.41	0.94	0.4	0.11	0.6	0.83	0.06	0	0.96	0.23	0.04	0	0	0	0	0	0	0	0	0	0	0	0	1	0	1	0.03	0.26	0	0.99	0	0.19	0.95	0.15	0.15	1	0.88
016	0	1	2	0	0	0.73	0.18	0.89	0.96	0.35	0.84	0.41	0.94	0.4	0.11	0.6	0.83	0.06	0	0.96	0.23	0.04	0	0	0	0	0	0	0	0	0	0	0	0	1	0	1	0.03	0.26	0	0.28	0	0.19	0.95	0.15	0.15	1	0.88
016	0	1	3	0	0	0.87	0.18	0.89	0.96	0.35	0.84	0.41	0.94	0.4	0.11	0.6	0.83	0.06	0	0.96	0.23	0.04	0	0	0	0	0	0	0	0	0	0	0	0	1	0	1	0.03	0.26	0	0.99	0	0.19	0.95	0.15	0.15	1	0.88
016	0	1	4	0	0	0.72	0.1	0.38	0.97	0.35	0	0.43	0.92	0.3	0.11	0.6	0.84	0.06	0	0.96	0.28	0.04	0	0	0	0	0	0	0	0	0	0	0	0	1	0	1	0.03	0.29	0	0.99	0.19	0	0	0.15	0.15	0.37	0.88
016	0	1	5	0	0	0.87	0.18	0.89	0.96	0.35	0.84	0.41	0.94	0.4	0.11	0.6	0.83	0.06	0	0.96	0.23	0.04	0	0	0	0	0	0	0	0	0	0	0	0	1	0	1	0.03	0.26	0	0.99	0	0.19	0.95	0.15	0.15	1	0.88
016	0	1	6	0	0	0.87	0.18	0.89	0.96	0.35	0.84	0.41	0.94	0.4	0.11	0.6	0.83	0.06	0	0.96	0.23	0.04	0	0	0	0	0	0	0	0	0	0	0	0	1	0	1	0.03	0.26	0	0.99	0	0.19	0.94	0.15	0.15	1	0.88
016	0	1	8	0	0	0.87	0.18	0.89	0.96	0.35	0.84	0.41	0.94	0.4	0.11	0.6	0.83	0.06	0	0.96	0.23	0.04	0	0	0	0	0	0	0	0	0	0	0	0	1	0	1	0.03	0.26	0	0.99	0	0	0.95	0.15	0.15	1	0.88
016	0	1	9	0	0	0.74	0.18	0.89	0.96	0.35	0.84	0.41	0.94	0.4	0.11	0.6	0.83	0.06	0	0.96	0.23	0.04	0	0	0	0	0	0	0	0	0	0	0	0	1	0	1	0.03	0.26	0	0.99	0	0.19	0.95	0.15	0.15	1	0.88
016	1	1	10	0	0.33	0.87	0.1	0.38	0.97	0.35	0	0.43	0.92	0.3	0.11	0.6	0.84	0.06	0	0.96	0.28	0.04	0	0	0	0	0	0	0	0	0	0	0	0	1	0	1	0.03	0.29	0	0.99	0.19	0.19	0	0.15	0.15	0.37	0.88
016	1	1	1	0	0.33	0.88	0.1	0.38	0.97	0.35	0	0.43	0.92	0.3	0.11	0.6	0.84	0.06	0	0.96	0.28	0.04	0	0	0	0	0	0	0	0	0	0	0	0	1	0	1	0.03	0.29	0	0.99	0.19	0.19	0	0.15	0.15	0.37	0.88
016	1	1	2	0	0	0.87	0.1	0.38	0.97	0.35	0	0.43	0.92	0.3	0.11	0.6	0.84	0.06	0	0.96	0.28	0.04	0	0	0	0	0	0	0	0	0	0	0	0	1	0	1	0.03	0.29	0	0.99	0.19	0	0	0.15	0.15	0.37	0.88
016	1	1	3	0	0	0.51	0.1	0.38	0.97	0.35	0	0.43	0.92	0.3	0.11	0.6	0.84	0.06	0	0.96	0.28	0.04	0	0	0	0	0	0	0	0	0	0	0	0	0.65	0	1	0.03	0.29	0	0.99	0.19	0	0	0.15	0.15	0.37	0.88
016	1	1	4	0	0.33	0.72	0.1	0.38	0.97	0.35	0	0.43	0.92	0.3	0.11	0.6	0.84	0.06	0	0.96	0.28	0.04	0	0	0	0	0	0	0	0	0	0	0	0	0.07	0	0	0.03	0.29	0	0.99	0.19	0.19	0	0.15	0.15	0.37	0.88
016	1	1	5	0	0.33	0.74	0.1	0.38	0.97	0.35	0	0.43	0.92	0.3	0.11	0.6	0.84	0.06	0	0.96	0.28	0.04	0	0	0	0	0	0	0	0	0	0	0	0	1	0	1	0.03	0.29	0	0.99	0.19	0.19	0	0.15	0.15	0.37	0.67
016	1	1	6	0	0.33	0.87	0.1	0.38	0.97	0.35	0	0.43	0.92	0.3	0.11	0.6	0.84	0.06	0	0.96	0.28	0.04	0	0	0	0	0	0	0	0	0	0	0	0	1	0	1	0.03	0.29	0	0.99	0.19	0.19	0	0.15	0.15	0.37	0.88
016	1	1	7	0	0.33	0.72	0.1	0.89	0.98	0.34	0	0.31	0.94	0.4	0.11	0.6	0.82	0.07	0	0.96	0.35	0.05	0	0	0	0	0	0	0	0	0	0	0	0	1	0	1	0.03	0.2	0.99	0.86	0	0.19	0.93	0.15	0.15	1	0.88
016	1	1	8	0	0.33	0.72	0.1	0.38	0.97	0.35	0	0.43	0.92	0.3	0.11	0.6	0.84	0.06	0	0.96	0.28	0.04	0	0	0	0	0	0	0	0	0	0	0	0	1	0	1	0.03	0.29	0	0.99	0.19	0.19	0	0.15	0.15	0.37	0.88
016	1	1	9	0	0	0.87	0.18	0.89	0.96	0.35	0.84	0.41	0.94	0.4	0.11	0.6	0.83	0.06	0	0.96	0.23	0.04	0	0	0	0	0	0	0	0	0	0	0	0	1	0	1	0.03	0.26	0	0.99	0	0.19	0.95	0.15	0.15	1	0.88
016	2	1	10	0	0.33	0.72	0.1	0.38	0.97	0.35	0	0.43	0.92	0.3	0.11	0.6	0.84	0.06	0	0.96	0.28	0.04	0	0	0	0	0	0	0	0	0	0	0	0	1	0	1	0.03	0.29	0	0.99	0.19	0.19	0	0.15	0.15	0.37	0.88
016	2	1	1E	0.97	0.98	0.87	0.99	0.27	0.32	1	0	0.04	1	0.04	0.22	0.59	0.96	0.96	0	0.21	0.74	0.96	0.47	0	0	0.74	0	0.94	0.95	0.05	0.82	0.24	0	0.84	1	0	1	1	0.81	0	0.04	0.91	0.68	1	1	1	0.04	1
016	2	1	1	0	0	0.72	0.1	0.38	0.97	0.35	0	0.43	0.92	0.3	0.11	0.6	0.83	0.06	0	0.96	0.28	0.04	0	0	0	0	0	0	0	0	0	0	0	0	1	0	1	0.03	0.29	0	0.99	0.19	0.19	0	0.15	0.15	0.37	0.88
016	2	1	2E	0.97	0.98	0.86	0.99	0.27	0.32	1	0	0.31	1	1	0.22	0.59	0.96	0.96	1	0.21	0.74	0.96	0.47	0	0	0.74	0	0.94	0.95	0.05	0.82	0.24	0	1	1	0	1	1	0.47	0	0.3	0.91	0.68	1	0.22	0.22	0.39	1

016	2	1	2	0	0	0.87	0.18	0.89	0.96	0.35	0.84	0.41	0.94	0.4	0.11	0.6	0.83	0.06	0	0.96	0.23	0.04	0	0	0	0	0	0	0	0	0	0	0	0	1	0	1	0.03	0.26	0	0.99	0	0.19	0.95	0.15	0.15	1	0.88
016	2	1	3E	0	0.98	0.87	0.99	0.27	0.32	1	0	0.31	1	1	0.22	0.59	0.96	0.96	1	0.21	0.74	0.96	0.47	0	0	0.74	0	0.94	0.95	0.05	0.82	0.24	0	0.82	1	0	1	1	0.81	0	0.3	0	0	1	1	1	0.39	1
016	2	1	3	0	0	0.87	0.18	0.89	0.96	0.35	0.84	0.41	0.94	0.4	0.11	0.6	0.83	0.06	0	0.96	0.23	0.04	0	0	0	0	0	0	0	0	0	0	0	0	1	0	1	0.03	0.26	0	0.99	0	0	0.95	0.15	0.15	1	0.88
016	2	1	4E	0	0.33	0.87	0.1	0.38	0.97	0.35	0	0.43	0.92	0.3	0.11	0.6	0.84	0.06	0	0.96	0.28	0.04	0	0	0	0	0	0	0	0	0	0	0	0	1	0	1	0.03	0.29	0	0.99	0.19	0.19	0	0.15	0.15	0.37	0.88
016	2	1	4	0	0	0.87	0.1	0.38	0.97	0.35	0	0.43	0.92	0.3	0.11	0.6	0.83	0.06	0	0.96	0.28	0.04	0	0	0	0	0	0	0	0	0	0	0	0	1	0	1	0.03	0.29	0	0.99	0.19	0	0	0.15	0.15	0.37	0.88
016	2	1	5E	0.97	0.98	0.88	0.99	0.38	0.97	1	0	0.31	1	1	0.22	0.59	0.96	0.96	1	0.96	0.74	0.96	0.47	0	0	0.74	0	0.94	0.95	0.05	0.82	0.24	0	1	1	0	1	1	0.81	0	0.99	0.91	1	1	1	1	0.39	1
016	2	1	5	0	0	0.87	0.18	0.89	0.96	0.35	0.84	0.41	0.93	0.4	0.11	0.6	0.83	0.06	0	0.96	0	0	0	0	0	0	0	0	0	0	0	0	0	0	1	0	1	0.03	0.26	0	0.99	0	0	0.95	0.15	0.15	1	0.88
016	2	1	6E	0	0	0.87	0.18	0.89	0.96	0.35	0.84	0.41	0.94	0.4	0.11	0.6	0.83	0.06	0	0.96	0.23	0.04	0	0	0	0	0	0	0	0	0	0	0	0	1	0	1	0.03	0.26	0	0.99	0	0.19	0.95	0.15	0.15	1	0.88
016	2	1	6	0	0	0.87	0.18	0.89	0.96	0.35	0.84	0.41	0.94	0.4	0.11	0.6	0.83	0.06	0	0.96	0.23	0.04	0	0	0	0	0	0	0	0	0	0	0	0	1	0	1	0.03	0.26	0	0.99	0	0	0.94	0.15	0.15	1	0.88
016	2	1	7E	0	0.91	0.87	0	0.27	0.32	1	0	0.06	1	0.07	0.22	0.59	0.96	0.96	0.32	0.21	0.74	0.96	0.47	0	0	0.74	0	0.94	0.95	0.05	0.82	0.24	0	1	1	0	1	1	0.2	0	0.06	0	0.19	1	1	1	0.04	1
016	2	1	7	0	0.33	0.88	0.1	0.38	0.97	0.35	0	0.43	0.92	0.3	0.11	0.6	0.84	0.06	0	0.96	0.28	0.04	0	0	0	0	0	0	0	0	0	0	0	0	1	0	1	0.03	0.29	0	0.99	0.19	0.19	0	0.15	0.15	0.37	0.88
016	2	1	8E	0.97	0.98	0.72	0.99	0.89	0.95	0.55	0.84	0.41	0.94	1	0.22	0.6	0.84	0.96	1	0.96	0.74	0.96	0.47	0	0	0.74	0	0.94	0.95	0.05	0.82	0.24	0	1	1	0	1	1	0.81	0	0.99	0.91	0.68	0.97	1	1	1	1
016	2	1	8	0	0	0.87	0.18	0.89	0.96	0.35	0.84	0.41	0.94	0.4	0.11	0.6	0.83	0.06	0	0.96	0.23	0.04	0	0	0	0	0	0	0	0	0	0	0	0	1	0	1	0.03	0.26	0	0.99	0	0.19	0.95	0.15	0.15	1	0.88
016	2	1	9	0	0	0.87	0.18	0.89	0.96	0.35	0.84	0.41	0.94	0.4	0.11	0.6	0.83	0.06	0	0.96	0.23	0.04	0	0	0	0	0	0	0	0	0	0	0	0.82	1	0	1	0.03	0.26	0	0.99	0	0.19	0.95	0.15	0.15	1	0.88
016	3	1	10	0	0	0.87	0.18	0.89	0.96	0.35	0.84	0.41	0.94	0.4	0.11	0.6	0.83	0.06	0	0.96	0.23	0.04	0	0	0	0	0	0	0	0	0	0	0	0	1	0	1	0.03	0.26	0	0.99	0	0.19	0.95	0.15	0.15	1	0.88
016	3	1	1	0	0	0.87	0.18	0.89	0.96	0.35	0.84	0.41	0.94	0.4	0.11	0.6	0.83	0.06	0	0.96	0.23	0.04	0	0	0	0	0	0	0	0	0	0	0	0	1	0	1	0.03	0.26	0	0.99	0	0.19	0.95	0.15	0.15	1	0.88
016	3	1	2	0	0	0.87	0.18	0.89	0.96	0.35	0.84	0.43	0.87	0.4	0.11	0.6	0.84	0.06	0	0.96	0.28	0.04	0	0	0	0	0	0	0	0	0	0	0	0	1	0	1	0.03	0.29	0	0.99	0	0	0.95	0.15	0.15	1	0.88
016	3	1	3	0	0.33	0.75	0.18	0.89	0.97	0.35	0.84	0.43	0.93	0.4	0.11	0.6	0.83	0.06	0	0.96	0.28	0.04	0	0	0	0	0	0	0	0	0	0	0	0	1	0	1	0.03	0.29	0	0.99	0	0	0.95	0.15	0.15	1	0.88
016	3	1	4	0	0	0.87	0.18	0.89	0.96	0.35	0.84	0.41	0.94	0.4	0.1	0.6	0.43	0.06	0	0.96	0.23	0.04	0	0	0	0	0	0	0	0	0	0	0	0	1	0	1	0.03	0.26	0	0.99	0	0.19	0.95	0.15	0.15	1	0.88
016	3	1	5	0	0	0.87	0.18	0.89	0.96	0.35	0.84	0.41	0.94	0.4	0.11	0.6	0.83	0.06	0	0.96	0.23	0.04	0	0	0	0	0	0	0	0	0	0	0	0	1	0	1	0.03	0.26	0	0.99	0	0.19	0.95	0.15	0.15	1	0.88
016	3	1	6	0	0	0.87	0.18	0.89	0.96	0.35	0.84	0.29	0.94	0.4	0.11	0.6	0.83	0.06	0	0.96	0.23	0.04	0	0	0	0	0	0	0	0	0	0	0	0	1	0	1	0.03	0.2	0	0.99	0	0.19	0	0.15	0.15	1	0.88
016	3	1	7	0	0	0.73	0.18	0.89	0.96	0.35	0.84	0.41	0.94	0.4	0.11	0.6	0.83	0.06	0	0.96	0.23	0.04	0	0	0	0	0	0	0	0	0	0	0	0	1	0	1	0.03	0.26	0	0.99	0	0.19	0.95	0.15	0.15	1	0.88
016	3	1	8	0	0	0.87	0.18	0.89	0.96	0.35	0.84	0.41	0.94	0.4	0.11	0.6	0.83	0.06	0	0.96	0.23	0.04	0	0	0	0	0	0	0	0	0	0	0	0	1	0	1	0.03	0.26	0	0.99	0	0.19	0.95	0.15	0.15	1	0.88
016	3	1	9	0	0	0.73	0.18	0.89	0.96	0.35	0.84	0.41	0.94	0.4	0.11	0.6	0.83	0.07	0	0.96	0.23	0.04	0	0	0	0	0	0	0	0	0	0	0	0	1	0	1	0.03	0.26	0	0.99	0	0.19	0.95	0.15	0.15	1	0.88
016	3	2	10	0	0	0.87	0.18	0.89	0.96	0.35	0.84	0.41	0.94	0.4	0.11	0.6	0.83	0.06	0	0.96	0.23	0.04	0	0	0	0	0	0	0	0	0	0	0	0	1	0	1	0.03	0.26	0	0.99	0	0.19	0.95	0.15	0.15	1	0.88
016	3	2	1	0	0	0.87	0.18	0.89	0.96	0.35	0.84	0.41	0.94	0.4	0.11	0.6	0.83	0.06	0	0.96	0.23	0.04	0	0	0	0	0	0	0	0	0	0	0	0	1	0	1	0.03	0.26	0	0.99	0	0.19	0.95	0.15	0.15	1	0.88
016	3	2	2	0	0.33	0.72	0.1	0.38	0.97	0.35	0	0.43	0.92	0.3	0.11	0.6	0.84	0.06	0	0.96	0.28	0.04	0	0	0	0	0	0	0	0	0	0	0	0	1	0	1	0.03	0.29	0	0.99	0.19	0.19	0	0.15	0.15	0.37	0.88
016	3	2	3	0	0	0.87	0.18	0.89	0.96	0.35	0.84	0.41	0.94	0.4	0.11	0.6	0.83	0.06	0	0.96	0.23	0.04	0	0	0	0	0	0	0	0	0	0	0	0	1	0	1	0.03	0.26	0	0.99	0	0.19	0.95	0.15	0.15	1	0.88
016	3	2	4	0	0.33	0.72	0.1	0.38	0.97	0.35	0	0.43	0.92	0.3	0.11	0.6	0.84	0.06	0	0.96	0.28	0.04	0	0	0	0	0	0	0	0	0	0	0	0	1	0	1	0	0.29	0	0.99	0.19	0.19	0	0.15	0.15	0.37	0.88
016	3	2	5	0	0	0.75	0.18	0.89	0.96	0.35	0.84	0.41	0.94	0.4	0.11	0.6	0.83	0.06	0	0.96	0.23	0.04	0	0	0	0	0	0	0	0	0	0	0	0	1	0	1	0.03	0.26	0	0.99	0	0.19	0.94	0.15	0.15	1	0.88
016	3	2	6	0	0.98	0.77	0.18	0.89	0.96	0.35	0.84	0.43	0.94	0.4	0.11	0.6	0.84	0.06	0	0.96	0.23	0.04	0	0	0	0	0	0	0	0	0	0	0	0	1	0	1	0.03	0.29	0	0.99	0	0	0.95	0.15	0.15	1	0.88
016	3	2	7	0	0.33	0.72	0.1	0.38	0.97	0.35	0	0.43	0.92	0.3	0.06	0.6	0.09	0.06	0	0.96	0.28	0.04	0	0	0	0	0	0	0	0	0	0	0	0	1	0	1	0.03	0.29	0	0.99	0	0.19	0	0.15	0.15	0.37	0.88
016	3	2	8	0	0.33	0.72	0.1	0.38	0.97	0.35	0	0.43	0.92	0.3	0.11	0.6	0.84	0.06	0	0.96	0.28	0.04	0	0	0	0	0	0	0	0	0	0	0	0	1	0	1	0.03	0.29	0	0.99	0.19	0.19	0	0.15	0.15	0.37	0.88
016	3	2	9	0	0	0.87	0.18	0.89	0.96	0.35	0.84	0.41	0.94	0.4	0.11	0.6	0.83	0.06	0	0.96	0.23	0.04	0	0	0	0	0	0	0	0	0	0	0	0	1	0	1	0.03	0.26	0	0.99	0	0.19	0.92	0.15	0.15	1	0.88
016	4	1	10	0	0	0.87	0.18	0.89	0.96	0.35	0.84	0.41	0.94	0.4	0.11	0.6	0.83	0.06	0	0.96	0.23	0.04	0	0	0	0	0	0	0	0	0	0	0	0	1	0	1	0.03	0.26	0	0.99	0	0.19	0.95	0.15	0.15	1	0.88
016	4	1	1	0	0	0.72	0.18	0.89	0.96	0.35	0.84	0.41	0.94	0.4	0.11	0.6	0.83	0.06	0	0.96	0.23	0.04	0	0	0	0	0	0	0	0	0	0	0	0	1	0	1	0.03	0.26	0	0.99	0	0.19	0.95	0.15	0.15	1	0.88
016	4	1	2	0	0.33	0.87	0.12	0.38	0.97	0.35	0	0.43	0.92	0.3	0.11	0.6	0.84	0.06	0	0.96	0.28	0.04	0	0	0	0	0	0	0	0	0	0	0	0	1	0	1	0.03	0.29	0	0.99	0.19	0	0.16	0.15	0.15	0.37	0.88
016	4	1	3	0	0.33	0.87	0.1	0.38	0.97	0.35	0	0.43	0.92	0.3	0.11	0.6	0.84	0.06	0	0.96	0.28	0.04	0	0	0	0	0	0	0	0	0	0	0	0	1	0	1	0.03	0.29	0	0.99	0.19	0.19	0	0.15	0.15	0.37	0.88
016	4	1	4	0	0	0.87	0.18	0.89	0.96	0.35	0.84	0.41	0.94	0.4	0.11	0.6	0.83	0.06	0	0.96	0.23	0.04	0	0	0	0	0	0	0	0	0	0	0	0	1	0	1	0.03	0.26	0	0.99	0	0.19	0.95	0.15	0.15	1	0.88
016	4	1	5	0.04	0.33	0.82	0.18	0.38	0.97	0.35	0.84	0.43	0.92	0.3	0.11	0.6	0.84	0.06	0	0.96	0.28	0.04	0	0	0	0	0	0	0	0	0	0	0	0	1	0	1	0.03	0.29	0	0.99	0.19	0	0	0.15	0.15	0.37	0.88
016	4	1	6	0	0	0.87	0.1	0.38	0.97	0.35	0	0.43	0.92	0.3	0.11	0.6	0.84	0.06	0	0.96	0.28	0.04	0	0	0	0	0	0	0	0	0	0	0	0	1	0	1	0.03	0.29	0	0.99	0.19	0	0	0.15	0.15	0.37	0.88
016	4	1	7	0	0.33	0.87	0.18	0.89	0.97	0.35	0.84	0.43	0.94	0.4	0.11	0.6	0.84	0.06	0	0.96	0.27	0.04	0	0	0	0	0	0	0	0	0	0	0	0	1	0	1	0.03	0.29	0	0.99	0.19	0	0.95	0.15	0.15	1	0.88
016	4	1	8	0	0.33	0.87	0.1	0.38	0.97	0.35	0	0.43	0.92	0.3	0.11	0.6	0.84	0.06	0	0.96	0.28	0.04	0	0	0	0	0	0	0	0	0	0	0	0	1	0	1	0.03	0.29	0	0.99	0.19	0	0.47	0.15	0.15	0.37	0.88
016	4	1	9	0	0	0.88	0.18	0.89	0.76	0.35	0.84	0.43	0.94	0.4	0.04	0.6	0.31	0.06	0	0.96	0.23	0.04	0	0	0	0	0	0	0	0	0	0	0	0	1	0	1	0.03	0.29	0	0.99	0	0	0.95	0.15	0.15	1	0.88
016	4	2	10	0	0	0.87	0.18	0.89	0.96	0.35	0.84	0.41	0.94	0.4	0.11	0.6	0.83	0.06	0	0.96	0.23	0.04	0	0	0	0	0	0	0	0	0	0	0	0	1	0	1	0.03	0.26	0	0.99	0	0.19	0.94	0.15	0.15	1	0.88
016	4	2	1	0	0	0.72	0.1	0.38	0.97	0.35	0	0.43	0.92	0.3	0.11	0.6	0.84	0.06	0	0.96	0.28	0.04	0	0	0	0	0	0	0	0	0	0	0	0	1	0	1	0.03	0.29	0	0.99	0.19	0	0	0.15	0.15	0.37	0.88
016	4	2	2	0	0	0.72	0.1	0.38	0.97	0.35	0	0.43	0.92	0.3	0.11	0.6	0.84	0.06	0	0.96	0.28	0.04	0	0	0	0	0	0	0	0	0	0	0	0	1	0	1	0.03	0.29	0	0.99	0.19	0	0	0.15	0.15	0.37	0.88
016	4	2	3	0	0	0.87	0.1	0.38	0.97	0.35	0	0.43	0.92	0.3	0.11	0.6	0.84	0.06	0	0.96	0.28	0.04	0	0	0	0	0	0	0	0	0	0	0	0	1	0	1	0.03	0.29	0	0.99	0.19	0.19	0	0.15	0.15	0.37	0.88
016	4	2	4	0	0.33	0.87	0.1	0.38	0.97	0.35	0	0.43	0.92	0.3	0.11	0.6	0.84	0.06	0	0.96	0.28	0.04	0	0	0	0	0	0	0	0	0	0	0	0	1	0	1	0.03	0.29	0	0.99	0.19	0.19	0	0.15	0.15	0.37	0.88
016	4	2	5	0	0.33	0.88	0.1	0.38	0.97	0.35	0	0.43	0.92	0.3	0.11	0.6	0.84	0.06	0	0.96	0.28	0.04	0	0	0	0	0	0	0	0	0	0	0	0	1	0	1	0.03	0.29	0	0.99	0.19	0.19	0	0.15	0.15	0.37	0.88
016	4	2	6	0	0	0.87	0.18	0.89	0.96	0.35	0.84	0.41	0.94	0.4	0.11	0.6	0.83	0.06	0	0.96	0.23	0.04	0	0	0	0	0	0	0	0	0	0	0	0	1	0	1	0.03	0.26	0	0.99	0	0.19	0.95	0.15	0.15	1	0.88
016	4	2	7	0	0	0.75	0.18	0.89	0.95	0.35	0.84	0.41	0.94	0.4	0.11	0.6	0.83	0.06	0	0.96	0.23	0.04	0	0	0	0	0	0	0	0	0	0	0	0	1	0	1	0.03	0.26	0	0.99	0	0	0.95	0.15	0.15	1	0.88
016	4	2	8	0	0	0.72	0.1	0.38	0.97	0.34	0	0.43	0.92	0.3	0.11	0.6	0.83	0.06	0	0.96	0.28	0.04	0	0	0	0	0	0	0	0	0	0	0	0	1	0	1	0.03	0.29	0	0.99	0.19	0	0	0.15	0.15	0.37	0.88
016	4	2	9	0	0.33	0.87	0.1	0.38	0.97	0.35	0	0.43	0.92	0.3	0.11	0.6	0.84	0.06	0	0.96	0.28	0.04	0	0	0	0	0	0	0	0	0	0	0	0	1	0	1	0.03	0.29	0	0.99	0.19	0.19	0	0.15	0.15	0.37	0.88
016	5	1	10	0	0.33	0.88	0.1	0.38	0.97	0.35	0	0.43	0.92	0.3	0.11	0.6	0.84	0.06	0	0.96	0.28	0.04	0	0	0	0	0	0	0	0	0	0	0	0	1	0	1	0.03	0.29	0	0.99	0.19	0.19	0	0.15	0.15	0.37	0.88
016	5	1	1	0	0.33	0.87	0.1	0.38	0.97	0.35	0	0.43	0.92	0.3	0.11	0.6	0.84	0.06	0	0.96	0.28	0.04	0	0	0	0	0	0	0	0	0	0	0	0	1	0	1	0.03	0.29	0	0.99	0.19	0.19	0	0.15	0.15	0.37	0.88
016	5	1	2	0	0.33	0.88	0.1	0.38	0.97	0.35	0	0.43	0.92	0.3	0.11	0.6	0.84	0.06	0	0.96	0.28	0.04	0	0	0	0	0	0	0	0	0	0	0	0	1	0	1	0.03	0.29	0	0.99	0.19	0.19	0	0.15	0.15	0.37	0.88
016	5	1	3	0	0.33	0.72	0.1	0.38	0.97	0.35	0	0.43	0.92	0.3	0.11	0.6	0.83	0.06	0	0.96	0.28	0.04	0	0	0	0	0	0	0	0	0	0	0	0	1	0	1	0.03	0.29	0	0.99	0.19	0.19	0	0.15	0.15	0.37	0.88
016	5	1	4	0	0	0.81	0.1	0.38	0.97	0.35	0	0.37	0.92	0.29	0.11	0.6	0.84	0.06	0	0.96	0.28	0.04	0	0	0	0	0	0	0	0	0	0	0	0	1	0	1	0.03	0.29	0	0.99	0.19	0	0	0.15	0.15	0.32	0.88
016	5	1	5	0	0.33	0.87	0.1	0.38	0.97	0.35	0	0.43	0.92	0.3	0.11	0.6	0.83	0.06	0	0.96	0.28	0.04	0	0	0	0	0	0	0	0	0	0	0	0	1	0	1	0.03	0.29	0	0.99	0.19	0.19	0	0.15	0.15	0.37	0.88
016	5	1	6	0	0.33	0.72	0.1	0.38	0.97	0.35	0	0.43	0.92	0.3	0.11	0.6	0.83	0.06	0	0.96	0.28	0.04	0	0	0	0	0	0	0	0	0	0	0	0	1	0	1	0.03	0.29	0	0.99	0.19	0.19	0	0.15	0.15	0.37	0.88
016	5	1	7	0	0.33	0.87	0.1	0.38	0.97	0.35	0	0.43	0.92	0.3	0.11	0.6	0.84	0.06	0	0.96	0.28	0.04	0	0	0	0	0	0	0	0	0	0	0	0	1	0	1	0.03	0.29	0	0.99	0.19	0.19	0	0.15	0.15	0.37	0.88
016	5	1	8	0	0.33	0.87	0.1	0.38	0.97	0.35	0	0.43	0.92	0.3	0.11	0.6	0.84	0.06	0	0.96	0.28	0.04	0	0	0	0	0	0	0	0	0	0	0	0	1	0	1	0.03	0.29	0	0.99	0.19	0.19	0	0.15	0.15	0.37	0.88
016	5	1	9	0	0.33	0.72	0.1	0.38	0.97	0.35	0	0.43	0.92	0.3	0.11	0.6	0.84	0.06	0	0.96	0.28	0.04	0	0	0	0	0	0	0	0	0	0	0	0	1	0	1	0.03	0.29	0	0.99	0.19	0.19	0	0.15	0.15	0.37	0.88
016	5	2	10	0	0	0.72	0.1	0.38	0.97	0.35	0	0.43	0.92	0.3	0.11	0.6	0.83	0.06	0	0.96	0.28	0.04	0	0	0	0	0	0	0	0	0	0	0	0	1	0	1	0.03	0.29	0	0.99	0.19	0	0	0.15	0.15	0.37	0.88
016	5	2	1	0	0.33	0.88	0.1	0.38	0.97	0.35	0	0.43	0.92	0.3	0.11	0.6	0.84	0.06	0	0.96	0.28	0.04	0	0	0	0	0	0	0	0	0	0	0	0	1	0	1	0.03	0.29	0	0.99	0.19	0.19	0	0.15	0.15	0.37	0.88
016	5	2	2	0	0.33	0.88	0.1	0.38	0.97	0.35	0	0.43	0.92	0.3	0.11	0.6	0.84	0.06	0	0.96	0.28	0.04	0	0	0	0	0	0	0	0	0	0	0	0	1	0	1	0.03	0.29	0	0.99	0.19	0.19	0	0.15	0.15	0.37	0.88
016	5	2	3	0	0.33	0.87	0.1	0.38	0.97	0.35	0	0.43	0.92	0.3	0.11	0.6	0.84	0.06	0	0.96	0.28	0.04	0	0	0	0	0	0	0	0	0	0	0	0	1	0	1	0.03	0.29	0	0.99	0.19	0.19	0	0.15	0.15	0.37	0.88
016	5	2	4	0	0	0.88	0.1	0.38	0.97	0.35	0	0.43	0.92	0.3	0.11	0.6	0.83	0.06	0	0.96	0.28	0.04	0	0	0	0	0	0	0	0	0	0	0	0	1	0	1	0.03	0.29	0	0.99	0.19	0.19	0	0.15	0.15	0.37	0.88
016	5	2	5	0	0	0.87	0.1	0.38	0.97	0.35	0	0.43	0.92	0.3	0.11	0.6	0.83	0.06	0	0.96	0.28	0.04	0	0	0	0	0	0	0	0	0	0	0	0	1	0	1	0.03	0.29	0	0.99	0.19	0	0	0.15	0.15	0.37	0.88

016	5	2 6	0	0	0.88	0.1	0.38	0.97	0.35	0	0.43	0.92	0.3	0.11	0.6	0.84	0.06	0	0.96	0.28	0.04	0	0	0	0	0	0	0	0	0	0	0	0	1	0	1	0.03	0.29	0	0.99	0.19	0.19	0	0.15	0.15	0.37	0.88
016	5	2 7	0	0.33	0.87	0.1	0.38	0.97	0.35	0	0.43	0.92	0.3	0.11	0.6	0.84	0.06	0	0.96	0.28	0.04	0	0	0	0	0	0	0	0	0	0	0	0	1	0	1	0.03	0.29	0	0.99	0.19	0.19	0	0.15	0.15	0.37	0.88
016	5	2 8	0	0.33	0.87	0.1	0.38	0.97	0.35	0	0.43	0.92	0.3	0.11	0.6	0.84	0.06	0	0.96	0.28	0.04	0	0	0	0	0	0	0	0	0	0	0	0	1	0	1	0.03	0.29	0	0.99	0.19	0.19	0	0.15	0.15	0.37	0.88
016	5	2 9	0	0	0.87	0.1	0.38	0.97	0.35	0	0.43	0.92	0.3	0.11	0.6	0.84	0.06	0	0.96	0.28	0.04	0	0	0	0	0	0	0	0	0	0	0	0	1	0	1	0.03	0.29	0	0.99	0.19	0	0	0.15	0.15	0.37	0.88
016	6	1 10	0	0	0.87	0.1	0.38	0.97	0.35	0	0.43	0.92	0.3	0.11	0.6	0.84	0.06	0	0.96	0.28	0.04	0	0	0	0	0	0	0	0	0	0	0	0	1	0	1	0.03	0.29	0	0.99	0.19	0.19	0	0.15	0.15	0.37	0.88
016	6	1 1	0	0	0.88	0.1	0.38	0.97	0.35	0	0.43	0.92	0.3	0.11	0.6	0.84	0.06	0	0.96	0.28	0.04	0	0	0	0	0	0	0	0	0	0	0	0	1	0	1	0.03	0.29	0	0.99	0.19	0	0	0.15	0.15	0.37	0.88
016	6	1 2	0	0	0.87	0.1	0.38	0.97	0.35	0	0.43	0.92	0.3	0.11	0.6	0.84	0.06	0	0.96	0.28	0.04	0	0	0	0	0	0	0	0	0	0	0	0	1	0	1	0.03	0.29	0	0.94	0.19	0	0	0.15	0.15	0.37	0.88
016	6	1 3	0	0	0.72	0.1	0.38	0.97	0.35	0	0.43	0.92	0.3	0.11	0.6	0.84	0.06	0	0.96	0.28	0.04	0	0	0	0	0	0	0	0	0	0	0	0	1	0	1	0.03	0.29	0	0.99	0.19	0	0	0.15	0.15	0.37	0.88
016	6	1 4	0	0	0.87	0.1	0.38	0.97	0.35	0	0.43	0.92	0.3	0.11	0.6	0.84	0.06	0	0.96	0.28	0.04	0	0	0	0	0	0	0	0	0	0	0	0	1	0	1	0.03	0.29	0	0.99	0.19	0.19	0	0.15	0.15	0.37	0.88
016	6	1 5	0	0	0.87	0.1	0.38	0.97	0.35	0	0.43	0.92	0.3	0.11	0.6	0.83	0.06	0	0.96	0.28	0.04	0	0	0	0	0	0	0	0	0	0	0	0	1	0	1	0.03	0.29	0	0.99	0.19	0	0	0.15	0.15	0.37	0.88
016	6	1 6	0	0	0.72	0.1	0.38	0.97	0.35	0	0.43	0.92	0.3	0.11	0.6	0.84	0.06	0	0.96	0.28	0.04	0	0	0	0	0	0	0	0	0	0	0	0	1	0	1	0.03	0.29	0	0.99	0.19	0	0	0.15	0.15	0.37	0.88
016	6	1 7	0	0.33	0.87	0.1	0.38	0.97	0.35	0	0.43	0.92	0.3	0.11	0.6	0.84	0.06	0	0.96	0.28	0.04	0	0	0	0	0	0	0	0	0	0	0	0	1	0	1	0.03	0.29	0	0.99	0.19	0.19	0	0.15	0.15	0.37	0.88
016	6	1 8	0	0	0.69	0.1	0.38	0.97	0.35	0	0.43	0.92	0.3	0.11	0.6	0.84	0.06	0	0.96	0.28	0.04	0	0	0	0	0	0	0	0	0	0	0	0	1	0	1	0.03	0.29	0	0.99	0.19	0	0	0.15	0.15	0.37	0.88
016	6	1 9	0	0	0.87	0.1	0.38	0.97	0.35	0	0.43	0.92	0.3	0.11	0.6	0.84	0.06	0	0.96	0.28	0.04	0	0	0	0	0	0	0	0	0	0	0	0	1	0	1	0.03	0.29	0	0.99	0.19	0	0	0.15	0.15	0.37	0.88
016	6	2 10	0	0.33	0.83	0.1	0.38	0.97	0.35	0	0.43	0.92	0.3	0.11	0.6	0.84	0.06	0	0.96	0.28	0.04	0	0	0	0	0	0	0	0	0	0	0	0	1	0	1	0.03	0.29	0	0.99	0.19	0.19	0	0.15	0.15	0.37	0.88
016	6	2 1	0	0.33	0.57	0.1	0.38	0.97	0.35	0	0.43	0.92	0.3	0.11	0.6	0.84	0.06	0	0.96	0.28	0.04	0	0	0	0	0	0	0	0	0	0	0	0	1	0	1	0.03	0.29	0	0.99	0.19	0.19	0	0.15	0.15	0.37	0.88
016	6	2 2	0	0	0.87	0.1	0.38	0.97	0.35	0	0.43	0.92	0.3	0.11	0.6	0.83	0.06	0	0.96	0.28	0.04	0	0	0	0	0	0	0	0	0	0	0	0	1	0	1	0.03	0.29	0	0.99	0.19	0	0	0.15	0.15	0.37	0.88
016	6	2 3	0	0.33	0.87	0.1	0.38	0.97	0.35	0	0.43	0.92	0.3	0.11	0.6	0.84	0.06	0	0.96	0.28	0.04	0	0	0	0	0	0	0	0	0	0	0	0	1	0	1	0.03	0.29	0	0.99	0.19	0.19	0	0.15	0.15	0.37	0.88
016	6	2 4	0	0.33	0.87	0.1	0.38	0.97	0.35	0	0.43	0.92	0.3	0.11	0.6	0.83	0.06	0	0.96	0.28	0.04	0	0	0	0	0	0	0	0	0	0	0	0	1	0	1	0.03	0.29	0	0.99	0.19	0.19	0	0.15	0.15	0.37	0.88
016	6	2 5	0	0.33	0.87	0.1	0.38	0.97	0.35	0	0.43	0.92	0.3	0.11	0.6	0.84	0.06	0	0.96	0.28	0.04	0	0	0	0	0	0	0	0	0	0	0	0	1	0	1	0.03	0.29	0	0.99	0.19	0.19	0	0.15	0.15	0.37	0.88
016	6	2 6	0	0.33	0.72	0.1	0.38	0.97	0.35	0	0.43	0.92	0.3	0.11	0.6	0.84	0.06	0	0.96	0.28	0.04	0	0	0	0	0	0	0	0	0	0	0	0	1	0	1	0.03	0.29	0	0.99	0.19	0.19	0	0.15	0.15	0.37	0.88
016	6	2 7	0	0	0.72	0.1	0.38	0.97	0.35	0	0.43	0.92	0.3	0.11	0.6	0.84	0.06	0	0.96	0.28	0.04	0	0	0	0	0	0	0	0	0	0	0	0	1	0	1	0.03	0.29	0	0.99	0.19	0	0	0.15	0.15	0.37	0.88
016	6	2 8	0	0	0.72	0.1	0.38	0.97	0.35	0	0.43	0.92	0.3	0.11	0.6	0.84	0.06	0	0.96	0.28	0.04	0	0	0	0	0	0	0	0	0	0	0	0	1	0	1	0.03	0.29	0	0.99	0.19	0	0	0.15	0.15	0.37	0.88
016	6	2 9	0	0.33	0.72	0.1	0.38	0.97	0.35	0	0.43	0.92	0.3	0.11	0.6	0.84	0.06	0	0.96	0.28	0.04	0	0	0	0	0	0	0	0	0	0	0	0	1	0	1	0.03	0.29	0	0.99	0.19	0.19	0	0.15	0.15	0.37	0.88
016	7	1 10	0	0	0.88	0.1	0.38	0.97	0.35	0	0.43	0.92	0.3	0.11	0.6	0.84	0.06	0	0.96	0.28	0.04	0	0	0	0	0	0	0	0	0	0	0	0	1	0	1	0.03	0.29	0	0.99	0.19	0	0	0.15	0.15	0.37	0.88
016	7	1 1	0	0.33	0.84	0.1	0.38	0.97	0.35	0	0.43	0.92	0.3	0.11	0.6	0.84	0.06	0	0.96	0.28	0.04	0	0	0	0	0	0	0	0	0	0	0	0	1	0	1	0.03	0.29	0	0.99	0.19	0.19	0	0.15	0.15	0.37	0.88
016	7	1 2	0	0.33	0.88	0.1	0.38	0.97	0.35	0	0.43	0.92	0.3	0.11	0.6	0.84	0.06	0	0.96	0.28	0.04	0	0	0	0	0	0	0	0	0	0	0	0	1	0	1	0.03	0.29	0	0.99	0.19	0.19	0	0.15	0.15	0.37	0.88
016	7	1 3	0	0.33	0.88	0.1	0.38	0.97	0.35	0	0.43	0.92	0.3	0.11	0.6	0.84	0.06	0	0.96	0.28	0.04	0	0	0	0	0	0	0	0	0	0	0	0	1	0	1	0.03	0.29	0	0.99	0.19	0.19	0	0.15	0.15	0.37	0.88
016	7	1 4	0	0.33	0.72	0.1	0.38	0.97	0.35	0	0.43	0.92	0.3	0.11	0.6	0.84	0.06	0	0.96	0.28	0.04	0	0	0	0	0	0	0	0	0	0	0	0	1	0	1	0.03	0.29	0	0.99	0.19	0.19	0	0.15	0.15	0.37	0.88
016	7	1 5	0	0	0.87	0.1	0.38	0.97	0.35	0	0.43	0.92	0.3	0.11	0.6	0.84	0.06	0	0.96	0.28	0.04	0	0	0	0	0	0	0	0	0	0	0	0	1	0	1	0.03	0.29	0	0.99	0.19	0	0	0.15	0.15	0.37	0.88
016	7	1 6	0	0.33	0.87	0.1	0.38	0.97	0.35	0	0.43	0.92	0.3	0.11	0.6	0.83	0.06	0	0.96	0.28	0.04	0	0	0	0	0	0	0	0	0	0	0	0	1	0	1	0.03	0.29	0	0.99	0.19	0.19	0	0.15	0.15	0.37	0.88
016	7	1 7	0	0.33	0.88	0.1	0.38	0.97	0.35	0	0.43	0.92	0.3	0.11	0.6	0.84	0.06	0	0.96	0.28	0.04	0	0	0	0	0	0	0	0	0	0	0	0	1	0	1	0.03	0.29	0	0.99	0.19	0.19	0	0.15	0.15	0.37	0.88
016	7	1 8	0	0.33	0.72	0.1	0.38	0.97	0.35	0	0.43	0.92	0.3	0.11	0.6	0.84	0.06	0	0.96	0.28	0.04	0	0	0	0	0	0	0	0	0	0	0	0	1	0	1	0.03	0.29	0	0.99	0.19	0.19	0	0.15	0.15	0.37	0.88
016	7	1 9	0	0	0.87	0.1	0.38	0.97	0.35	0	0.43	0.92	0.3	0.11	0.6	0.84	0.06	0	0.96	0.28	0.04	0	0	0	0	0	0	0	0	0	0	0	0	1	0	1	0.03	0.29	0	0.99	0.19	0	0	0.14	0.14	0.37	0.88
016	7	2 11	0	0	0.87	0.1	0.38	0.97	0.35	0	0.43	0.92	0.3	0.11	0.6	0.84	0.06	0	0.96	0.28	0.04	0	0	0	0	0	0	0	0	0	0	0	0	1	0	1	0	0.29	0	0.99	0.19	0	0	0.15	0.15	0.37	0.88
016	7	2 1	0	0.33	0.87	0.1	0.38	0.97	0.35	0	0.43	0.92	0.3	0.11	0.6	0.84	0.06	0	0.96	0.28	0.04	0	0	0	0	0	0	0	0	0	0	0	0	1	0	1	0.03	0.29	0	0.99	0.19	0.19	0	0.15	0.15	0.37	0.88
016	7	2 2	0	0.33	0.88	0.1	0.38	0.97	0.35	0	0.43	0.92	0.3	0.11	0.6	0.84	0.06	0	0.96	0.28	0.04	0	0	0	0	0	0	0	0	0	0	0	0	1	0	1	0.03	0.29	0	0.99	0.19	0.19	0	0.15	0.15	0.37	0.88
016	7	2 3	0	0.33	0.72	0.1	0.38	0.97	0.35	0	0.43	0.92	0.3	0.11	0.6	0.83	0.06	0	0.96	0.28	0.04	0	0	0	0	0	0	0	0	0	0	0	0	1	0	1	0.03	0.29	0	0.99	0.19	0.19	0	0.15	0.15	0.37	0.88
016	7	2 4	0	0.33	0.88	0.1	0.38	0.97	0.35	0	0.43	0.92	0.3	0.11	0.6	0.84	0.06	0	0.96	0.28	0.04	0	0	0	0	0	0	0	0	0	0	0	0	1	0	1	0.03	0.29	0	0.99	0.19	0.19	0	0.15	0.15	0.37	0.88
016	7	2 5	0	0.33	0.87	0.1	0.38	0.97	0.35	0	0.43	0.92	0.3	0.11	0.6	0.84	0.06	0	0.96	0.28	0.04	0	0	0	0	0	0	0	0	0	0	0	0	1	0	1	0.03	0.29	0	0.99	0.19	0.19	0	0.15	0.15	0.37	0.88
016	7	2 6	0	0.33	0.87	0.1	0.38	0.97	0.35	0	0.43	0.92	0.3	0.11	0.6	0.84	0.06	0	0.96	0.28	0.04	0	0	0	0	0	0	0	0	0	0	0	0	1	0	1	0.03	0.29	0	0.99	0.19	0.19	0	0.15	0.15	0.37	0.88
016	7	2 7	0	0.33	0.88	0.1	0.38	0.97	0.35	0	0.43	0.92	0.3	0.11	0.6	0.83	0.06	0	0.96	0.28	0.04	0	0	0	0	0	0	0	0	0	0	0	0	1	0	1	0.03	0.29	0	0.99	0.19	0.19	0	0.15	0.15	0.37	0.88
016	7	2 8	0	0.33	0.87	0.1	0.38	0.97	0.35	0	0.43	0.92	0.3	0.11	0.6	0.84	0.06	0	0.96	0.28	0.04	0	0	0	0	0	0	0	0	0	0	0	0	1	0	1	0.03	0.29	0	0.99	0.19	0.19	0	0.15	0.15	0.37	0.88
016	7	2 9	0	0.33	0.88	0.1	0.38	0.97	0.35	0	0.43	0.92	0.3	0.11	0.6	0.84	0.06	0	0.96	0.28	0.04	0	0	0	0	0	0	0	0	0	0	0	0	1	0	1	0.03	0.29	0	0.99	0.19	0.19	0	0.15	0.15	0.37	0.88
016	21	1 3	0	0	0.72	0.1	0.38	0.97	0.35	0	0.43	0.92	0.3	0.11	0.6	0.84	0.06	0	0.96	0.28	0.04	0	0	0	0	0	0	0	0	0	0	0	0	1	0	1	0.03	0.29	0	0.99	0.19	0.19	0	0.15	0.15	0.37	0.88
016	21	1 4	0	0.33	0.87	0.1	0.38	0.97	0.35	0	0.43	0.92	0.3	0.11	0.6	0.84	0.06	0	0.96	0.28	0.04	0	0	0	0	0	0	0	0	0	0	0	0	1	0	1	0	0.29	0	0.99	0.19	0.19	0	0.15	0.15	0.37	0.88
016	21	1 5	0	0.33	0.87	0.1	0.38	0.97	0.35	0	0.43	0.92	0.3	0.11	0.6	0.84	0.06	0	0.96	0.28	0.04	0	0	0	0	0	0	0	0	0	0	0	0	1	0	1	0.03	0.29	0	0.99	0.19	0.19	0	0.15	0.15	0.37	0.88
016	21	1 6	0	0	0.86	0.1	0.38	0.97	0.35	0	0.43	0.92	0.3	0.11	0.6	0.83	0.06	0	0.96	0.28	0.04	0	0	0	0	0	0	0	0	0	0	0	0	1	0	1	0.03	0.29	0	0.99	0.19	0	0	0.15	0.15	0.37	0.88
016	21	1 7	0	0.33	0.73	0.1	0.38	0.97	0.35	0	0.43	0.92	0.3	0.11	0.6	0.84	0.06	0	0.96	0.28	0.04	0	0	0	0	0	0	0	0	0	0	0	0	1	0	1	0.03	0.29	0	0.99	0.19	0.19	0	0.15	0.15	0.37	0.88
016	28	1 2	0	0	0.72	0.1	0.38	0.97	0.35	0	0.43	0.92	0.3	0.11	0.6	0.84	0.06	0	0.96	0.28	0.04	0	0	0	0	0	0	0	0	0	0	0	0	1	0	1	0.03	0.29	0	0.99	0.19	0	0	0.15	0.15	0.37	0.88
016	28	1 4	0	0.33	0.72	0.1	0.38	0.97	0.35	0	0.43	0.92	0.3	0.11	0.6	0.84	0.06	0	0.96	0.28	0.04	0	0	0	0	0	0	0	0	0	0	0	0	1	0	1	0.03	0.29	0	0.99	0.19	0.19	0	0.15	0.15	0.37	0.88
016	28	1 5	0	0	0.87	0.1	0.38	0.97	0.35	0	0.43	0.92	0.3	0.11	0.6	0.84	0.06	0	0.96	0.28	0.04	0	0	0	0	0	0	0	0	0	0	0	0	1	0	1	0.03	0.29	0	0.99	0.19	0	0	0.15	0.15	0.37	0.88
016	28	1 8	0	0	0.72	0.1	0.38	0.97	0.35	0	0.43	0.92	0.3	0.11	0.6	0.84	0.06	0	0.96	0.28	0.04	0	0	0	0	0	0	0	0	0	0	0	0	1	0	1	0.03	0.29	0	0.99	0.19	0	0	0.15	0.15	0.37	0.88
016	28	1 9	0	0.33	0.88	0.1	0.38	0.97	0.35	0	0.43	0.92	0.3	0.11	0.6	0.84	0.06	0	0.64	0.28	0.04	0	0	0	0	0	0	0	0	0	0	0	0	1	0	1	0.03	0.29	0	0.99	0.19	0.19	0	0.15	0.15	0.37	0.88
