# Supplementary material for: Responses of the Human Gut Escherichia coli Population to Pathogen and Antibiotic Disturbances
Source: mSystems. 2018 Jul 24;3(4):e00047-18. doi: 10.1128/mSystems.00047-18 (PMC6060285; doi:10.1128/mSystems.00047-18)
Supplement: TABLE S7 [file sys004182251st7.pdf]

Table S7: Proportions of resistance genes relative to number of genomes in each subject during each trial phase.

		Prechallenge				Challenge						Postchallenge				
**Name**	**Resistance description**	**001**	**006**	**008**	**015**	**001**	**006**	**008**	**009**	**015**	**016**	**001**	**006**	**008**	**009**	**016**
APH(3'')-Ib	antibiotic inactivation enzyme; determinant of aminoglycoside resistance	0.1	0	0	0	0.054	0	0	0	0	0	0.27	0.01	0	0	0
APH(6)-Id	antibiotic inactivation enzyme; determinant of aminoglycoside resistance	0.2	0	0	0	0.054	0	0	0	0	0	0.27	0.01	0	0	0
aadA5	antibiotic inactivation enzyme; determinant of aminoglycoside resistance	0.3	0	1	0	0.022	0	0.05	0	0	0	0.09	0.01	0.46	0.1	0
kdpE	determinant of aminoglycoside resistance; protein(s) and two-component regulatory system modulating antibiotic efflux	1.3	0.9	1	1.2	0.924	0.95	1.14	0.98	1	0.85	1.09	1.01	1.02	1	1.088
LRA-18-lik	antibiotic inactivation enzyme; determinant of beta-lactam resistance; LRA-18-like	1	1.4	1	1	0.88	1	1.03	1.05	1.01	0.87	1	1.03	1	1	1
SHV-75-lik	antibiotic inactivation enzyme; determinant of beta-lactam resistance; SHV-75-like	0	0.1	0	0	0.13	0	0.95	0	0	0	0.05	0.04	0.15	0	0
TEM-1	antibiotic inactivation enzyme; determinant of beta-lactam resistance	0.1	0.8	0.25	0	0.25	0	0.02	0	0	0	0.2	0.47	0.57	0	0
TEM-171	antibiotic inactivation enzyme; determinant of beta-lactam resistance	0	0	0	0	0	0	0	0.02	0.01	0	0	0	0	0	0
dfrA17	antibiotic target replacement protein; determinant of diaminopyrimidine resistance	0.3	0	0	0	0.033	0	0	0	0	0	0.15	0.01	0	0	0
parC	Escherichia coli parC conferring resistance to fluoroquinolone; S80I;gene involved in self-resistance to antibiotic; antibiotic resistant gene variant or mutant; determinant of fluoroquinolone resistance	1	0.9	1	0	0.804	0	0.02	0	0	0.32	0.97	1	0.92	0.4	0.963
mfd	antibiotic target protection protein; determinant of fluoroquinolone resistance	1	0.9	1	1	0.88	0.95	0.98	1	1	0.87	1	1	1	1	1
gyrA	Escherichia coli gyrA conferring resistance to fluoroquinolones; D87N, S83L, D87N, A85T, D87N, S95T, D94N, D95N, D95N, S101L	1	0.9	1	0	0.837	0	0.02	0		0.31	0.99	0.97	0.92	0.4	0.963
parE	Salmonella serovars parE conferring resistance to fluoroquinolones	0	0	0	0	0	0	0	0	0	0	0	0	0	0	0.013
GlpT	Escherichia coli GlpT with mutation conferring resistance to fosfomycin; E448K;	1	0.9	1	1	0.837	0	0.02	0	0.14	0.79	1	0.97	1	0.6	1
UhpT	Escherichia coli UhpT with mutation conferring resistance to fosfomycin; E350Q	1	0.9	1	0	0.848	0	0.02	0	0	0.81	0.99	0.99	0.92	0.4	1
mphA	antibiotic inactivation enzyme; determinant of macrolide resistance	0.3	0	1	0	0.022	0	0.02	0	0	0	0.12	0.01	0.4	0	0
Mrx	determinant of macrolide resistance; antibiotic inactivation enzyme	0.4	0	1	0	0.054	0	0.02	0	0	0	0.2	0.01	0.4	0	0
bacA	determinant of resistance to peptide antibiotics; gene conferring antibiotic resistance via molecular bypass	1	0.9	1	1	0.859	0.95	1	1	1	0.87	0.99	1	1	1	0.988
catI	determinant of phenicol resistance; antibiotic inactivation enzyme	0	0	0	0	0	0	0	0.02	0.01	0	0	0	0	0	0
arnA	determinant of polymyxin resistance; gene altering cell wall charge	1	0.9	1	1	0.88	0.95	0.98	1	1	0.88	1.04	1.03	1.06	1	1
PmrC	determinant of polymyxin resistance; gene altering cell wall charge	1	1.4	1	1	0.88	0.95	1	1	1	0.94	1	1.03	1	1	1
PmrE	determinant of polymyxin resistance; gene altering cell wall charge	1	1.8	2	1	0.88	0.95	1.02	0.98	1	0.85	1.04	1.2	1.83	1.4	1.038
PmrF	determinant of polymyxin resistance; gene altering cell wall charge	1	0.9	1	1	0.88	0.95	0.98	1	1	0.88	1.04	1.03	1.06	1	1
leuO	protein(s) and two-component regulatory system modulating antibiotic efflux; determinant of sulfonamide resistance	1	0.9	1	1	0.88	0.95	1	1	1	0.87	1	1	1	1	1
sul1	antibiotic target replacement protein; determinant of sulfonamide resistance	0.4	0	1	0	0.033	0	0.02	0	0	0	0.14	0.01	0.4	0	0
sul2	antibiotic target replacement protein; determinant of sulfonamide resistance	0.1	0	0	0	0.011	0	0	0	0	0	0.18	0.01	0	0	0
