# Supplementary material for: Responses of the Human Gut Escherichia coli Population to Pathogen and Antibiotic Disturbances
Source: mSystems. 2018 Jul 24;3(4):e00047-18. doi: 10.1128/mSystems.00047-18 (PMC6060285; doi:10.1128/mSystems.00047-18)
Supplement: TABLE S8 [file sys004182251st8.pdf]

Table S8. Genes not in common in Day 30 isolates from subject 008 and 009

Function	008_D28_1	008_D28_3	008_D28_5	008_D28_7	008_D28_8	009_D28_3	009_D28_4	009_D28_5	009_D28_6	009_D28_7
putative integrase	1	0.38	0.51	0.78	0.99	0.23	0.35	0.17	0.99	0.99
tRNA-Tyr-GTA	1	0.72	1	0.71	0.68	0.71	0.71	0.45	1	0.71
conserved hypothetical, putative RHS repeat protein	1	0.97	0.97	0.97	0.97	0.8	0.97	0.97	0.97	0.7
hypothetical	1	0.68	0.68	1	0.68	0.68	0.68	0.68	0.68	1
NADH pyrophosphatase (EC 3.6.1.22)	1	1	1	1	1	1	1	0.42	1	1
tRNA-Gly-GCC	1	0.53	0.53	0.53	0.52	0.54	0.82	0.53	0.53	0.53
RNA polymerase sigma factor RpoS	1	1	1	1	0.9	1	1	0.64	0.8	1
hypothetical	1	1	1	1	1	1	1	1	1	0.33
PTS system, galactitol-specific IIB component, putative	1	1	1	1	1	1	1	1	1	0.2
DNA gyrase inhibitory protein	1	1	1	1	1	1	1	1	1	0.11
Histidinol-phosphate aminotransferase (EC 2.6.1.9)	1	1	1	1	1	1	1	1	1	0.06
Mobile element protein	1	1	1	1	0.98	0.96	0.97	0.83	1	1
Citrate lyase beta chain (EC 4.1.3.6)	1	1	1	1	1	0.82	1	0.99	1	1
Magnesium and cobalt transport protein CorA	1	1	1	1	1	1	1	1	0.81	0.81
putative DNA-binding protein	1	0.79	0.79	0.79	0.79	0.79	0.79	0.79	0.79	0.79
Mobile element protein	1	1	1	1	1	1	1	1	1	0.69
Phage integrase	1	1	1	1	1	1	1	1	1	0.43
FIG00638424: hypothetical protein	1	1	1	1	1	1	1	1	1	0
adhesin	1	1	1	1	1	1	1	1	1	0.08
Cytochrome b561 homolog 1	1	1	1	1	1	1	1	1	1	0.42
Outer membrane porin protein NmpC precursor	1	1	1	1	1	1	1	1	1	0.54
Flagellar motor switch protein FliM	1	1	1	1	1	1	1	1	1	0
Flagellum-specific ATP synthase FliI	1	1	1	1	1	1	1	1	1	0.48
Gifsy-2 prophage protein	1	1	1	1	1	1	1	1	1	0
Flagellar biosynthesis protein FliC	1	1	1	1	1	1	1	1	1	0.11
Excinuclease ABC subunit C	1	1	1	1	1	1	1	1	1	0.05
FIG00510289: hypothetical protein	1	1	1	1	1	1	1	1	1	0
Alpha,alpha-trehalose-phosphate synthase [UDP-forming] (EC 2.4.1.15)	1	1	1	1	1	1	1	1	1	0
Flagellar transcriptional activator FlhD	1	1	1	1	1	1	1	1	1	0
Flagellar transcriptional activator FlhC	1	1	0.99	1	1	1	1	1	1	0
Chemotaxis response regulator protein-glutamate methylesterase CheB (EC 3.1.1.61)	1	1	1	1	1	1	1	1	1	0.09
Chemotaxis regulator - transmits chemoreceptor signals to flagelllar motor components CheY	1	1	1	1	1	1	1	1	1	0.29
Cytoplasmic copper homeostasis protein CutC	1	1	1	1	1	1	1	1	1	0
Cytochrome c-type protein TorY	1	1	1	1	1	1	1	1	1	0.28
FIG000859: hypothetical protein YebC	1	1	1	1	1	1	1	1	1	0
Cell wall endopeptidase, family M23/M37	1	1	1	1	1	1	1	1	1	0.19
FIG01200701: possible membrane protein	1	1	1	1	1	1	1	1	1	0
FIG00639796: hypothetical protein	1	1	1	1	1	1	1	1	1	0.26
FIG00638105: hypothetical protein	1	1	1	1	1	1	1	1	1	0
Cold shock protein CspC	1	1	1	1	1	1	1	1	1	0.85
Hypothetical nudix hydrolase YeaB	1	1	1	1	1	1	1	1	1	0
MltA-interacting protein MipA	1	1	1	1	1	1	1	1	1	0.35
NAD-dependent glyceraldehyde-3-phosphate dehydrogenase (EC 1.2.1.12)	1	1	1	1	1	1	1	1	1	0.42
Uncharacterized sugar kinase YdjH	1	1	1	1	1	1	1	1	1	0.15
Anthranilate synthase, aminase component (EC 4.1.3.27)	1	1	1	1	0.7	1	1	1	1	1
Putative sulfite oxidase subunit YedY	1	1	1	1	1	1	1	1	1	0
Colanic acid capsular biosynthesis activation accesory protein RcsA, co-regulator with RcsB	1	1	1	1	1	1	1	1	1	0.09
Nicotinamidase (EC 3.5.1.19)	1	1	1	1	1	1	1	1	1	0.89
Uncharacterized protein YeaC	1	1	1	1	1	1	1	1	1	0
Aldose 1-epimerase family protein YeaD	1	1	1	1	1	1	1	1	1	0
Inner membrane protein YeaI	1	1	1	1	1	1	1	1	1	0.33
FIG074102: hypothetical protein	1	1	1	1	1	1	1	1	1	0
Choline-glycine betaine transporter	1	1	1	1	1	1	1	1	1	0.24
Long-chain-fatty-acid--CoA ligase (EC 6.2.1.3)	1	1	1	1	1	1	1	1	1	0.16
RidA/YER057c/UK114 superfamily, group 2, YoaB-like protein	1	1	1	1	1	1	1	1	1	0.21
FIG00638134: hypothetical protein	1	1	1	1	1	1	1	1	1	0
Putative transport protein	1	1	1	1	1	1	1	1	1	0.18
Ribosomal RNA small subunit methyltransferase F (EC 2.1.1.-)	1	1	1	1	1	1	1	1	1	0.09
FIG004088: inner membrane protein YebE	1	1	1	1	1	1	1	1	1	0
FIG00637868: hypothetical protein	1	1	1	1	1	1	1	1	1	0.18
Flagellar protein FlhE	1	1	1	1	1	1	1	1	1	0
Flagellar biosynthesis protein FlhA	1	1	1	1	1	1	1	1	1	0.41
Chemotaxis response - phosphatase CheZ	1	1	1	1	1	1	1	1	1	0
Positive regulator of CheA protein activity (CheW)	1	1	1	1	1	1	1	1	1	0
Ferritin-like protein 2	1	1	1	1	1	1	1	1	1	0
UPF0033 protein YedF	1	1	1	1	1	1	1	1	1	0.27
Flagellar hook-basal body complex protein FliE	1	1	1	1	1	1	1	1	1	0.22
Flagellar assembly protein FliH	1	1	1	1	1	1	1	1	1	0.15
Pyruvate dehydrogenase E1 component (EC 1.2.4.1)	1	1	1	1	1	1	1	1	0.73	1
Biotin operon repressor / Biotin-protein ligase (EC 6.3.4.15)	1	1	1	1	1	1	1	0	1	1
LysR-family transcriptional regulator YdhB	1	1	0.84	1	1	1	1	0.84	0.97	1
Hexose phosphate uptake regulatory protein UhpC	1	1	1	1	1	0.71	1	1	1	1
core protein	1	1	0.97	1	1	1	0.77	1	1	0.74
core protein	0.92	1	1	0.95	0.98	0.95	0.56	1	0.9	1
Multimodular transpeptidase-transglycosylase (EC 2.4.1.129) (EC 3.4.-.-)	1	1	1	1	1	1	0.84	1	1	1
Hydroxymethylpyrimidine phosphate synthase ThiC (EC 4.1.99.17)	1	1	1	1	1	1	1	0.77	1	1
Mobile element protein	0.85	1	0.59	0.52	0.7	0.63	0.9	0.91	0.71	0.91
PTS system, galactitol-specific IIB component (EC 2.7.1.69)	1	1	1	1	1	1	1	1	1	0.27
Transcriptional repressor of the fructose operon, DeoR family	1	1	1	1	1	1	1	1	1	0.27
PTS system, galactitol-specific IIA component (EC 2.7.1.69)	1	1	1	1	1	1	1	1	1	0.22
FIG00644922: hypothetical protein	1	1	1	1	1	1	1	1	1	0.12
FIG00638167: hypothetical protein	1	1	1	1	1	1	1	1	1	0
hypothetical protein	0.84	0.84	1	0.84	0.84	0.84	0.84	0.84	0.84	0.84
UDP-glucose 6-dehydrogenase (EC 1.1.1.22)	1	1	1	1	1	1	1	1	1	0.38
Histidinol-phosphatase (EC 3.1.3.15) / Imidazoleglycerol-phosphate dehydratase (EC 4.2.1.19)	1	1	1	1	1	1	1	1	1	0.11
ATP phosphoribosyltransferase (EC 2.4.2.17)	1	1	1	1	1	1	1	1	1	0
PTS system, galactitol-specific IIC component (EC 2.7.1.69)	1	1	1	1	1	1	1	1	1	0.29
hypothetical protein	1	1	1	1	1	1	1	1	1	0
Translation elongation factor Tu	0.81	0.81	1	0.81	0.81	0.81	0.81	0.81	0.81	0.81
Mobile element protein	0.65	0.65	1	0.65	0.65	0.65	0.65	0.65	0.65	0.65

Preprotein translocase subunit SecE (TC 3.A.5.1.1)	1	1	1	1	1	1	1	0	1	1
Nickel transport ATP-binding protein NikE (TC 3.A.1.5.3)	1	1	1	1	1	0.99	1	0.71	1	1
metal-dependent phosphohydrolase	1	1	1	1	1	1	1	1	1	0
Flagellar biosynthesis protein FliL	1	1	1	1	1	1	1	1	1	0
Cytoplasmic alpha-amylase (EC 3.2.1.1)	1	1	1	1	1	1	1	1	1	0.04
Flagellar biosynthesis protein FliT	1	1	1	1	1	1	1	1	1	0
Methyl-accepting chemotaxis protein I (serine chemoreceptor protein)	1	1	1	1	1	1	1	1	1	0.56
Flagellar biosynthesis protein FlhB	1	1	1	1	1	1	1	1	1	0.29
conserved hypothetical	1	1	1	1	1	1	1	1	1	0
Dihydroneopterin triphosphate pyrophosphohydrolase type 2 (nudB)	1	1	1	1	1	1	1	1	1	0.09
Holliday junction DNA helicase RuvB	1	1	1	1	1	1	1	1	1	0.1
Zinc ABC transporter, inner membrane permease protein ZnuB	1	1	1	1	1	1	1	1	1	0.07
Zinc ABC transporter, ATP-binding protein ZnuC	1	1	1	1	1	1	1	1	1	0.2
Copper resistance protein CopC	1	1	1	1	1	1	1	1	1	0.29
Transcriptional regulator KdgR, KDG operon repressor	1	1	1	1	1	1	1	1	1	0.25
PTS system, mannose-specific IID component	1	1	1	1	1	1	1	1	1	0.3
Uncharacterized protein YeaK	1	1	1	1	1	1	1	1	1	0
Putative transport protein YdjK, MFS superfamily	1	1	1	1	1	1	1	1	1	0.14
core protein	0.89	0.94	1	0.98	0.91	0.8	0.85	1	0.99	0.99
conserved hypothetical	0	0	0	1	0	0	0	0	0	0
putative plasmid replication protein	0	0	0	1	0	0.46	0	0	0	0
putative transposase	0	0	0.97	1	0	0	0	0	0	0
Hypothetical oxidoreductase YdjG (EC 1.-.-.-)	1	1	1	1	1	1	1	1	1	0.21
Peptide methionine sulfoxide reductase MsrB (EC 1.8.4.12)	1	1	1	1	1	1	1	1	1	0
Serine protein kinase (prkA protein), P-loop containing	1	1	1	1	1	1	1	1	1	0
FIG074102: hypothetical protein	1	1	1	1	1	1	1	1	1	0
Ribonuclease D (EC 3.1.26.3)	1	1	1	1	1	1	1	1	1	0
FIG00639292: hypothetical protein	1	1	1	1	1	1	1	1	1	0.15
L-serine dehydratase, beta subunit (EC 4.3.1.17) / L-serine dehydratase, alpha subunit (EC 4.3.1.17)	1	1	1	1	1	1	1	1	1	0.78
Rtn protein	1	1	1	1	1	1	1	1	1	0.21
PTS system, mannose-specific IIA component (EC 2.7.1.69) / PTS system, mannose-specific IIB component (EC 2.7.1.69)	1	1	1	1	1	1	1	1	1	0.16
Protease II (EC 3.4.21.83)	1	1	1	1	1	1	1	1	1	0
DNA damage-inducible gene in SOS regulon, dependent on cyclic AMP and H-NS	1	1	1	1	1	1	1	1	1	0
Pyruvate kinase (EC 2.7.1.40)	1	1	1	1	1	1	1	1	1	0.31
Lipid A biosynthesis (KDO) 2-(lauroyl)-lipid IVA acyltransferase (EC 2.3.1.-)	1	1	1	1	1	1	1	1	1	0.18
Holliday junction DNA helicase RuvA	1	1	1	1	0.99	1	1	1	1	0.08
Trimethylamine-N-oxide reductase (EC 1.6.6.9)	1	1	1	1	1	1	1	1	1	0.63
Flagellar motor rotation protein MotB	1	1	1	1	1	1	1	1	1	0.24
Trehalose-6-phosphate phosphatase (EC 3.1.3.12)	1	1	1	1	1	1	1	1	1	0
L-arabinose transport system permease protein (TC 3.A.1.2.2)	1	1	1	1	1	1	1	1	1	0.32
FIG00638267: hypothetical protein	1	1	1	1	0.98	1	1	1	1	0
FIG00638619: hypothetical protein	1	1	1	1	1	1	1	1	1	0
FIG00637942: hypothetical protein	1	1	1	1	1	1	1	1	1	0.6
Flagellar motor switch protein FliG	1	1	1	1	1	1	1	1	1	0.24
Flagellar motor switch protein FliN	1	1	1	1	1	1	1	1	1	0.25
Flagellar biosynthesis protein FliP	1	1	1	1	1	1	1	1	1	0.44
Flagellar biosynthesis protein FliQ	1	1	1	1	1	1	0.97	1	1	0.38
FIG00638997: hypothetical protein	1	1	1	1	1	1	1	1	1	0.09
5-Hydroxyisourate Hydrolase (HIUase) (EC 3.5.2.17)	1	1	1	1	1	1	1	1	1	0
Candidate zinc-binding lipoprotein ZinT	1	1	1	1	1	1	1	1	1	0
LSU ribosomal protein L7/L12 (P1/P2)	1	1	1	1	1	1	1	0.16	1	1
Beta-galactosidase (EC 3.2.1.23)	1	1	0.74	1	1	1	1	1	1	1
Putative deaminase YahJ	0.79	0.78	0.78	1	0.78	0.8	0.81	0.78	0.78	0.78
6-phospho-3-hexuloisomerase	1	1	1	1	1	1	1	1	1	0.12
Ribulose-phosphate 3-epimerase (EC 5.1.3.1)	1	1	1	1	1	1	1	1	1	0.37
PTS system, galactitol-specific IIC component (EC 2.7.1.69)	1	1	1	1	1	1	1	1	1	0.3
PTS system, galactitol-specific IIA component (EC 2.7.1.69)	1	1	1	1	1	1	1	1	1	0.38
Ribose 5-phosphate isomerase B (EC 5.3.1.6)	0.99	1	1	1	1	1	1	1	1	0.24
Nicotinate-nucleotide--dimethylbenzimidazole phosphoribosyltransferase (EC 2.4.2.21)	1	1	1	1	1	1	1	1	1	0
Hypothetical transcriptional regulator yeeY	1	0.99	1	1	1	0.99	1	0.99	1	0.3
Nucleoside-diphosphate-sugar epimerases	1	1	1	1	1	1	1	1	1	0.06
Imidazole glycerol phosphate synthase cyclase subunit (EC 4.1.3.-)	0.7	1	1	1	1	1	1	0.7	1	0
regulator of length of O-antigen component of lipopolysaccharide chains	1	1	1	1	1	1	1	1	1	0.18
FIG01069516: hypothetical protein	1	1	1	1	1	1	1	1	1	0.11
Shikimate transporter	1	1	1	1	1	1	1	1	1	0.4
Magnesium and cobalt transport protein CorA	0.8	0.8	0.8	1	0.8	0.8	0.8	0.8	0.8	0.8
Aspartokinase (EC 2.7.2.4) / Homoserine dehydrogenase (EC 1.1.1.3)	0.81	1	1	1	1	1	1	1	1	1
FIG074102: hypothetical protein	0.73	0.73	0.73	1	0.73	0.97	0.73	0.73	0.73	0.73
hypothetical	0	0	0	1	0	0.8	0	0	0	0
Putative two component system histidine kinase YedV	1	1	1	1	1	1	1	1	1	0.17
Permease of the drug/metabolite transporter (DMT) superfamily	1	1	1	1	1	0.99	1	1	1	0.13
FIG00640199: hypothetical protein	1	1	1	1	1	1	1	1	1	0
Flagellar hook-associated protein FliD	1	1	1	1	1	1	1	1	1	0.18
Cystine ABC transporter, ATP-binding protein	0.99	1	1	1	1	1	1	1	1	0.52
L-arabinose transport ATP-binding protein AraG (TC 3.A.1.2.2)	1	1	1	1	1	1	1	1	1	0.41
Chemotaxis protein methyltransferase CheR (EC 2.1.1.80)	1	1	1	1	1	1	1	1	1	0
Protein yecM	1	1	1	1	1	1	1	1	1	0
tRNA (uridine-5-oxyacetic acid methyl ester) 34 synthase	1	1	1	1	1	1	1	1	1	0
4-hydroxy-2-oxoglutarate aldolase (EC 4.1.3.16) @ 2-dehydro-3-deoxyphosphogluconate aldolase (EC 4.1.2.14)	1	1	1	1	1	1	1	1	1	0.17
Phosphoribosylglycinamide formyltransferase 2 (EC 2.1.2.-)	1	1	1	1	1	1	1	1	1	0.09
FIG00510464: hypothetical protein	1	1	1	1	1	1	1	1	1	0
Starvation lipoprotein Slp paralog	1	1	1	1	1	1	1	1	1	0
Flavodoxin reductases (ferredoxin-NADPH reductases) family 1; Vanillate O-demethylase oxidoreductase (EC 1.14.13.-)	1	1	1	1	1	1	1	0.99	1	0.61
FIG00638567: hypothetical protein	1	1	1	1	1	1	1	1	1	0.13
LSU ribosomal protein L11p (L12e)	1	1	1	1	1	1	1	0	1	1
Sulfur carrier protein adenylyltransferase ThiF	1	1	1	1	1	1	1	0.62	1	1
Uroporphyrinogen III decarboxylase (EC 4.1.1.37)	1	1	1	1	1	1	1	0.82	1	1
conserved hypothetical	1	1	1	1	1	1	0	1	1	1
tRNA(Ile)-lysidine synthetase (EC 6.3.4.19)	1	1	1	1	1	1	0.99	0.82	1	1
Mobile element protein	0.99	1	1	1	1	1	0.99	0.99	1	0.06
Integrase	0.92	0.98	0.92	0.92	1	0.92	0.51	0.81	0.92	0.13
Alkanesulfonate utilization operon LysR-family regulator Cbl	1	1	1	1	1	1	1	1	1	0.39
FIG007491: hypothetical protein YeeN	1	1	1	1	1	1	1	1	1	0
AMP nucleosidase (EC 3.2.2.4)	1	1	1	1	1	1	1	1	1	0.04
Invasin	1	1	1	1	1	1	1	1	1	0
TldD protein, part of TldE/TldD proteolytic complex	0.86	0.86	0.86	0.86	1	0.86	0.86	0.86	0.86	0.86
Mobile element protein	0.78	0.78	0.78	0.78	1	0.78	0.78	0.78	0.78	0.77
Cobalamin synthase (EC 2.7.8.26)	1	1	1	1	1	1	1	1	1	0

L,D-transpeptidase YbiS	1	1	1	1	1	1	1	1	1	0.63
hypothetical protein	0	0	0	0	0	1	0	0	0	0
putative replication protein	0	0	0	0	0	1	0	0	0	0
IncI1 plasmid conjugative transfer DNA primase	0	0	0	0	0	1	0	0	0	0
hypothetical	0	0	0	0.75	0	1	0	0	0	0
putative inititator RepB protein	0	0	0	0	0	1	0	0	0	0
hypothetical	0	0	0	0	0	1	0	0	0	0
replication protein	0	0	0	0	0	1	0	0	0	0
hypothetical protein	0	0	0	0	0	1	0	0	0	0
hypothetical	0	0	0	0	0	1	0	0	0	0
putative ribokinase operon repressor	0.89	0.89	0.89	0.89	0.89	1	0.89	0.89	0.89	0.86
putative DNA binding protein	0.68	1	1	1	1	1	0.98	0.68	1	1
conserved hypothetical, putative conjugal transfer protein	0	0	0	0	0	1	0	0	0	0
Entericidin B precursor	1	1	1	1	0.72	1	1	1	1	1
FIG01047791: hypothetical protein	0.76	0.76	0.76	0.76	0.76	1	0.76	0.76	0.76	0.76
FIG00639198: hypothetical protein	0.82	0.76	0.96	0.76	0.53	1	0.45	0.45	0.85	1
Glutamate decarboxylase (EC 4.1.1.15)	0.88	0.88	0.88	0.87	0.87	1	0.88	0.88	0.88	0.87
Imidazole glycerol phosphate synthase amidotransferase subunit (EC 2.4.2.-)	1	1	1	1	1	1	1	1	1	0.08
Regulator of sigma D	1	1	1	1	1	1	1	0.13	1	1
2-iminoacetate synthase (ThiH) (EC 4.1.99.19)	1	1	1	1	1	1	1	0.04	1	1
LSU ribosomal protein L1p (L10Ae)	1	1	1	1	1	1	1	0	1	1
hypothetical protein	1	1	0.99	0.99	0.99	1	1	1	0.99	0
FIG001196: Membrane protein YedZ	1	1	1	1	1	1	1	1	1	0.28
Copper-sensing two-component system response regulator CusR	1	1	1	1	1	1	1	1	1	0.45
Very-short-patch mismatch repair endonuclease (G-T specific)	1	1	1	1	1	1	1	1	1	0
Putative inner membrane protein	1	1	0.99	1	1	1	1	1	1	0
FIG00638072: hypothetical protein	1	1	1	1	1	1	1	1	1	0
putative cytoplasmic protein	1	1	1	1	1	1	1	1	1	0
Flagellar biosynthesis protein FliR	1	1	1	1	1	1	1	1	1	0.27
Flagellar hook-length control protein FliK	1	1	1	1	1	1	1	1	1	0
Flagellar biosynthesis protein FliZ	1	1	1	1	1	1	1	1	1	0
N-3-oxohexanoyl-L-homoserine lactone quorum-sensing transcriptional activator @ N-3-oxooctanoyl-L-homoserine lactone quorum-sensing transcriptional	1	1	1	1	1	1	1	1	1	0.09
FIG00634007: hypothetical protein	1	1	1	1	1	1	1	1	1	0
Tyrosine-specific transport protein	1	1	1	1	1	1	1	1	1	0.3
Exodeoxyribonuclease X (EC 3.1.11.-)	1	1	1	1	1	1	1	1	1	0.12
Paraquat-inducible protein A	1	1	1	1	1	1	1	1	1	0.19
Probable protease HtpX (EC 3.4.24.-)	1	1	1	0.99	1	1	1	1	1	0.08
FIG00638740: hypothetical protein	1	1	1	1	1	1	1	1	1	0.16
Putative membrane protein	1	1	1	1	1	1	1	1	1	0
PTS system, mannose-specific IIC component	1	1	1	1	1	1	1	1	1	0.24
TsaB protein, required for threonylcarbamoyladenosine (t(6)A) formation in tRNA	1	1	1	1	1	1	0.99	1	1	0.12
Putative dioxygenase, alpha subunit (EC 1.-.-.-); Choline monooxygenase, alpha subunit (EC 1.14.15.7)	1	1	1	1	1	1	1	1	1	0.1
Positive regulator of Tartrate dehydrogenase/decarboxylase/D-malic enzyme	1	1	1	1	1	1	1	1	1	0.46
Transglycosylase associated protein	1	1	1	1	1	1	1	1	1	0.72
FIG00638318: hypothetical protein	1	1	1	1	1	1	1	1	1	0.17
Putative oxidoreductase YdjL	1	1	1	1	1	1	1	1	1	0.22
Putative transport protein YdjK, MFS superfamily	1	1	1	1	1	1	1	1	1	0.13
Hypothetical zinc-type alcohol dehydrogenase-like protein YdjJ	1	1	1	1	1	1	1	1	1	0.22
Diaminopimelate decarboxylase (EC 4.1.1.20)	0	0	0	0.53	0	1	0	0	0	0
Uncharacterized protein YgeA of aspartate/glutamate/hydantoin racemase family	1	1	1	1	0.29	1	1	1	1	1
UDP-N-acetylenolpyruvoylglucosamine reductase (EC 1.1.1.158)	1	1	1	1	1	1	1	0.83	1	1
hypothetical protein	0.79	1	0.99	0.96	0.79	0.79	1	1	1	0.77
core protein	0.72	0.72	0.72	0.72	0.72	0.72	1	0.72	0.72	0.67
FIG00638308: hypothetical protein	1	1	1	1	1	1	1	1	1	0.15
DinG family ATP-dependent helicase YoaA	1	1	1	1	1	1	1	1	1	0.16
Ribosomal RNA large subunit methyltransferase A (EC 2.1.1.51)	1	1	1	1	1	1	1	1	1	0.07
Copper resistance protein D	1	1	1	1	1	1	1	1	1	0.14
Phosphogluconate dehydratase (EC 4.2.1.12)	1	1	1	1	1	1	1	1	1	0.16
Crossover junction endodeoxyribonuclease RuvC (EC 3.1.22.4)	1	1	1	1	1	1	1	1	0.99	0
Nicotinamidase/isochorismatase family protein	1	1	1	1	1	1	1	1	1	0.17
FIG003003: hypothetical protein	1	1	1	1	1	1	1	1	1	0
Arginyl-tRNA synthetase (EC 6.1.1.19)	1	1	1	1	1	1	1	1	1	0
Methyl-accepting chemotaxis protein IV (dipeptide chemoreceptor protein)	1	1	1	1	1	1	1	1	1	0.43
CDP-diacylglycerol--glycerol-3-phosphate 3-phosphatidyltransferase (EC 2.7.8.5)	1	1	1	1	1	1	1	1	1	0.09
Cystine ABC transporter, permease protein	1	1	1	1	1	1	1	1	1	0.33
Flagellar biosynthesis protein FliS	1	1	1	1	1	1	1	1	1	0
DsrB protein	1	1	1	1	1	1	1	1	1	0
Putative mannosyl-3-phosphoglycerate phosphatase (EC 3.1.3.70)	1	1	1	1	1	0.99	1	1	1	0
Chaperone protein hchA	1	1	1	1	1	1	1	1	1	0.06
Phosphoribosylformimino-5-aminoimidazole carboxamide ribotide isomerase (EC 5.3.1.16)	1	1	1	1	1	1	1	1	1	0.07
Exodeoxyribonuclease I (EC 3.1.11.1)	1	1	1	1	1	1	1	1	1	0
UPF0265 protein YeeX	1	1	1	1	1	1	1	1	1	0
3-oxoacyl-[acyl-carrier protein] reductase (EC 1.1.1.100)	1	1	1	1	1	1	1	1	1	0.3
FIG01047791: hypothetical protein	1	1	1	1	1	0.75	1	1	1	1
Type I restriction-modification system, specificity subunit S (EC 3.1.21.3)	1	0.84	1	1	1	1	1	1	1	1
LSU ribosomal protein L10p (P0)	1	1	1	1	1	1	1	0	1	1
Phosphoribosylamine--glycine ligase (EC 6.3.4.13)	1	1	1	1	1	1	1	0.04	1	1
hypothetical	1	1	1	1	1	1	1	1	1	0
Enterobactin synthetase component F, serine activating enzyme (EC 2.7.7.-)	1	1	1	1	1	0.53	1	1	1	1
BigB	1	0.72	0.86	1	1	0.84	1	1	1	0.86
HigA protein (antitoxin to HigB)	1	1	1	1	1	1	1	0.73	1	1
hypothetical protein	0.86	0.86	0.86	0.86	0.86	0.86	0.86	1	0.86	0.86
core protein	0.72	0.72	0.72	0.72	0.72	0.72	0.69	1	0.72	0.72
Putrescine importer	1	1	1	1	1	1	1	1	1	0.65
D-alanyl-D-alanine carboxypeptidase (EC 3.4.16.4)	1	1	1	1	1	1	1	1	1	0.47
Putative inner membrane protein	1	1	1	1	1	1	1	1	1	0.06
Adenosylcobinamide-phosphate guanylyltransferase (EC 2.7.7.62)	1	1	1	1	1	1	1	1	1	0
Putative aldolase YdjI	1	1	1	1	1	1	1	1	1	0.3
UPF0229 protein YeaH	1	1	1	1	1	1	1	1	1	0
Putative two-component response regulator and GGDEF family protein YeaJ	1	1	1	1	1	1	1	1	1	0.09
Putative inner membrane protein	1	1	1	1	1	1	1	1	1	0.13
Transcriptional regulator, AraC family	1	1	1	1	1	1	1	1	1	0.07
Para-aminobenzoate synthase, aminase component (EC 2.6.1.85)	1	1	1	1	1	1	1	1	1	0.19
Tail-specific protease precursor (EC 3.4.21.102)	1	1	1	1	1	1	1	1	1	0
ProQ: influences osmotic activation of compatible solute ProP	1	1	1	1	1	1	1	1	1	0.03
Free methionine-(R)-sulfoxide reductase, contains GAF domain	1	1	1	1	1	1	1	1	1	0.07
FIG00638765: hypothetical protein	1	1	1	1	1	1	1	1	1	0
FIG00923183: possible membrane protein YebY	1	1	1	1	1	1	1	1	1	0.2

Glucose-6-phosphate 1-dehydrogenase (EC 1.1.1.49)	1	1	1	1	1	1	1	1	1	0
hypothetical protein	1	1	1	1	1	1	1	1	1	0
Phosphogluconate repressor HexR, RpiR family	1	1	1	1	1	1	1	1	1	0.17
Zinc ABC transporter, periplasmic-binding protein ZnuA	1	1	1	1	1	1	1	1	1	0.06
Aspartyl-tRNA synthetase (EC 6.1.1.12)	1	1	1	1	1	1	1	1	1	0.08
tRNA (5-methoxyuridine) 34 synthase	1	1	1	1	1	1	1	1	1	0.06
Flagellar motor rotation protein MotA	1	1	1	1	1	1	1	1	1	0.36
Universal stress protein C	1	1	1	1	1	1	1	1	1	0.36
L-arabinose-binding periplasmic protein precursor AraF (TC 3.A.1.2.2)	1	1	1	1	1	1	1	1	1	0.08
D-cysteine desulfhydrase (EC 4.4.1.15)	1	1	1	1	1	1	1	1	1	0.07
Cystine ABC transporter, periplasmic cystine-binding protein FliY	1	1	1	1	1	1	1	1	1	0.24
RNA polymerase sigma factor for flagellar operon	1	1	1	1	1	1	1	1	1	0.27
Putative transport system permease protein	1	1	1	1	1	1	1	1	1	0.04
Flagellar M-ring protein FliF	1	1	1	1	1	1	1	1	1	0.19
Flagellar protein FliJ	1	1	1	1	1	1	1	1	1	0.11
FIG00638388: hypothetical protein	1	1	1	1	1	1	1	1	1	0
Type III secretion inner membrane protein (YscS,homologous to flagellar export components)	1	1	1	1	1	0.71	1	1	1	1
Unsaturated fatty acid biosythesis repressor FabR, TetR family	0.99	0.88	1	0.99	0.99	1	0.99	1	1	1
adherence and invasion outermembrane protein (Inv,enhances Peyer's patches colonization)	1	1	1	1	1	1	1	1	1	0.18
Nitrogen assimilation regulatory protein Nac	1	1	1	1	1	1	1	1	1	0.17
Translation elongation factor Tu	0.46	0.46	0.46	0.46	0.46	0.46	0.46	1	0.46	0.46
Ribulokinase (EC 2.7.1.16)	1	0.8	1	1	1	1	1	1	1	1
2,3-dihydro-2,3-dihydroxybenzoate dehydrogenase (EC 1.3.1.28) of siderophore biosynthesis	1	1	1	1	1	0.66	1	1	1	1
Diaminohydroxyphosphoribosylaminopyrimidine deaminase (EC 3.5.4.26) / 5-amino-6-(5-phosphoribosylamino)uracil reductase (EC 1.1.1.193)	1	1	1	1	0.99	0.61	1	1	1	0.99
Phage tail fiber protein	1	1	1	1	0.63	1	1	1	1	1
tRNA-Ser-GGA	0.84	0.85	0.98	0.99	0.99	1	0.99	1	0.84	0.79
tRNA-Tyr-GTA	0.77	0.76	0.77	0.77	0.72	0.77	0.77	1	0.83	0.77
conserved hypothetical	0	0	0	0	0	0	0	0	1	0
Response regulator of zinc sigma-54-dependent two-component system	1	1	1	1	1	1	1	0.46	1	1
Zinc resistance-associated protein	1	1	1	1	1	1	1	0	1	1
FIG00638261: hypothetical protein	1	1	1	1	1	1	1	0	1	1
DNA-directed RNA polymerase beta subunit (EC 2.7.7.6)	1	1	1	1	1	1	1	0	1	1
mobilization protein MobC	0	0	0	0	0	0	0	0	1	0
Translation elongation factor Tu	1	1	0.99	1	0.97	1	1	0.8	1	1
DNA polymerase II (EC 2.7.7.7)	1	0.8	1	1	1	1	1	0.79	1	1
Phosphoribosyl-AMP cyclohydrolase (EC 3.5.4.19) / Phosphoribosyl-ATP pyrophosphatase (EC 3.6.1.31)	1	1	1	1	1	1	1	1	1	0
Histidinol dehydrogenase (EC 1.1.1.23)	1	1	1	1	1	1	1	1	1	0
Putative transport system permease protein	1	1	1	1	1	1	1	1	1	0.05
hypothetical protein	1	1	1	1	1	1	1	1	1	0
core protein	1	1	1	1	1	1	1	1	1	0.64
Putative HTH-type transcriptional regulator YdjF	1	1	1	1	1	1	1	1	1	0.25
Putative oxidoreductase YeaE, aldo/keto reductase family	1	1	1	1	1	1	1	1	1	0.19
FIG00638254: hypothetical protein	1	1	1	1	1	1	1	1	1	0
FIG00638802: hypothetical protein	1	1	1	1	1	1	1	1	1	0.17
Putative transport protein	1	1	1	1	1	1	1	1	1	0.19
Tartrate dehydrogenase (EC 1.1.1.93) / Tartrate decarboxylase (EC 4.1.1.73) / D-malic enzyme (EC 1.1.1.83)	0.99	1	1	1	1	1	1	1	1	0.26
Magnesium and cobalt efflux protein CorC	1	1	1	1	1	1	1	1	1	0.45
Paraquat-inducible protein B	1	1	1	1	1	1	1	1	1	0.1
Ren protein	1	1	1	1	1	1	1	1	1	0.39
DNA polymerase III theta subunit (EC 2.7.7.7)	1	1	1	1	1	1	1	1	1	0
Putative amidohydrolase	1	1	1	1	1	1	1	1	1	0
FIG01046209: inner membrane protein	1	1	1	1	1	1	1	1	1	0
Signal transduction histidine kinase CheA (EC 2.7.3.-)	1	1	1	1	1	1	1	1	1	0.04
Ferritin-like protein 2	1	1	1	1	1	1	1	1	1	0
BarA-associated response regulator UvrY (= GacA = SirA)	1	1	1	1	1	1	1	1	1	0.29
hypothetical	1	1	1	1	1	1	1	1	1	0
hypothetical	1	1	1	1	1	1	1	1	1	0
hypothetical	1	1	1	1	1	1	1	1	1	0
Flagellar biosynthesis protein FliQ	1	1	1	1	1	1	1	1	1	0
DNA-cytosine methyltransferase (EC 2.1.1.37)	1	1	1	1	1	1	1	1	1	0.09
Uncharacterized protein YehA precursor	1	1	1	1	1	1	0.62	1	1	1
GTPase (EC 3.6.1.-)	1	1	1	1	1	0.98	0.82	1	1	1
RNA polymerase sigma factor RpoS	0.99	0.99	0.99	0.99	0.63	0.99	0.99	0.99	1	0.99
conserved hypothetical	1	1	1	0.54	0.53	1	1	1	1	0.54
conserved hypothetical	1	0.55	0.55	0.55	0.55	0.8	0.55	0.55	0.83	1
IMP cyclohydrolase (EC 3.5.4.10) / Phosphoribosylaminoimidazolecarboxamide formyltransferase (EC 2.1.2.3)	1	1	1	1	1	1	1	0.88	1	1
Sensor protein of zinc sigma-54-dependent two-component system	1	1	1	1	1	1	1	0.19	1	1
Thiazole biosynthesis protein ThiG	1	1	1	1	1	0.99	1	0.6	1	1
DNA-directed RNA polymerase beta' subunit (EC 2.7.7.6)	1	1	1	1	1	1	1	0	1	1
Transcription antitermination protein NusG	1	1	1	1	1	1	1	0	1	1
Pantothenate kinase (EC 2.7.1.33)	1	1	1	1	1	1	1	0.1	1	1
putative RHS element protein RhsA	0.87	0.87	0.87	0.87	0.87	0.87	0.88	0.87	0.87	1
Small Subunit Ribosomal RNA; ssuRNA; SSU rRNA	1	1	1	0.99	1	0.88	1	1	1	1
Putative periplasmic protein YibQ, distant homology with nucleoside diphosphatase and polysaccharide deacetylase	1	1	0.62	1	1	0.65	1	1	1	1
